# A palladium catalyzed asymmetric desymmetrization approach to enantioenriched 1,3-disubstituted isoindolines†

**DOI:** 10.1039/d3sc03496h

**Published:** 2023-09-26

**Authors:** Dattatraya H. Dethe, Vimlesh Kumar, Manmohan Shukla

**Affiliations:** a Department of Chemistry, Indian Institute of Technology Kanpur Kanpur - 208016 India ddethe@iitk.ac.in

## Abstract

Herein, we report the first palladium/MPAA catalyzed enantioselective C–H activation/[4 + 1] annulation of diarylmethyltriflamide and olefins to construct chiral *cis*-1,3-disubstituted isoindoline derivatives. The use of a readily accessible mono-N-protected amino acid as a chiral ligand improves the efficiency and enantioselectivity of the catalytic transformation. The developed method provides access to both enantiomers of a product using either d or l-phenylalanine derivative as a chiral ligand facilitating the synthesis of both optically active 1,3-disubstituted isoindoline derivatives.

## Abstract 2

Herein, we report the first palladium/MPAA catalyzed enantioselective C–H activation/[4 + 1] annulation of diarylmethyltriflamide and olefins to construct chiral *cis*-1,3-disubstituted isoindoline derivatives.

## Graphical abstract

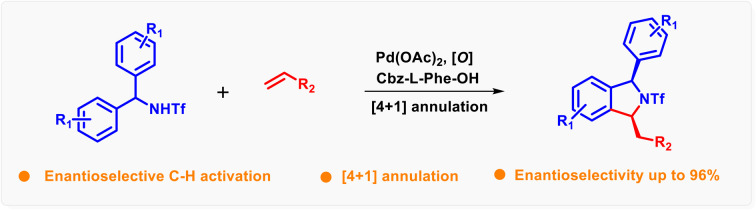

The development of synthetic methodologies that provide direct access to enantiopure molecules and intermediates expedites the biological investigations in the pharmaceutical industry and drug discovery. Various catalytic strategies have been developed to access such optically active compounds of great biological importance under asymmetric synthesis. In the past two decades, numerous potential synthetic methodologies have been developed by using a transition metal catalysed C–H bond functionalization strategy and their applications in synthetic chemistry are well-documented.^1^ A major ongoing challenge is the development of enantioselective versions of such reactions, especially the ones involved in the creation of asymmetry during the C–H activation step.^2^ Because of the inherent advantages of atom and step economy, an enantioselective C–H functionalization method offers a unique opportunity to produce chiral molecules of great synthetic interest.^3^ Transition metal-catalysed asymmetric functionalization of inert C–H bonds has attracted a lot of attention from synthetic chemists for the development of such novel reactions either by stereoselective C–H activation followed by inter- or intramolecular reactions, or by using prochiral starting materials to accomplish enantioselective desymmetrization.^4^ Despite the significant advancement in this area, there is still room to develop new and potential asymmetric C–H functionalization strategies for constructing new chiral scaffolds.^5^ In 2008, the Yu group reported the first palladium catalyzed enantioselective C–H activation by utilizing a mono-N-protected amino acid as a chiral ligand.^6^ Thereafter various Pd-catalysed asymmetric C–H functionalization reactions using a mono-N-protected amino acid as a chiral ligand have been reported.^7^ Mono-N-protected amino acids (MPAAs) are being successfully used as bifunctional ligands in various Pd-catalysed asymmetric C–H functionalization reactions.^7^ Mechanistic studies indicate that both *N*-acyl and carboxylate groups of MPAAs coordinate to the Pd and modulate the reactivity and stereoselectivity of the *in situ* forming catalyst for C–H activation.^8^ The *N*-acyl group of this ligand may act as an internal base to drive the C–H activation step *via* a concerted metalation–deprotonation (CMD) mechanism and the rigid MPAA chelation helps in proposing a realistic stereo-model to anticipate the stereo-chemical outcome of enantioselective processes.^8^ Various reactions have been developed for the construction of complex cyclic scaffolds from simpler acyclic starting materials by using transition metal catalyzed C–H activation followed by tandem annulation.^9^ In this context, Yu's group was the first to report a palladium catalyzed enantioselective C–H iodination reaction, which provides direct access of chiral diarylmethylamines (Scheme 1a).^10^ In 2019 Gulías's group reported palladium catalyzed desymmetrization of diarylmethyltriflamides by reaction with allenes to construct chiral tetrahydroisoquinolines (Scheme 1b).^11^ Later on, You and coworkers reported the first (*R*)-CpmRh catalyzed enantioselective [4 + 1] annulation reaction of benzamides and alkenes to afford a variety of isoindolinones (Scheme 1c).^12^ Optically pure isoindolines are constituents of many pharmaceuticals and diverse groups of bioactive natural products.^13^ Particularly, 1,3-disubstituted isoindoline derivatives possess antitumor activity in human melanoma cells when treated along with anticancer medicines. They also inhibit enzymes such as prolyl dipeptidases DPP8 and DPP9 (Fig. 1).^14^ Due to their potential biological importance, considerable efforts have been devoted to their efficient synthesis. However, there are only a few synthetic methodologies developed to access racemic and optically active 1,3-disubstituted isoindolines.^15^ There is still a significant need for developing efficient and versatile strategies for enantioselective and diastereoselective synthesis of 1,3-disubstituted isoindolines. We envisioned that enantioenriched 1,3-disubstituted isoindolines might be constructed by an asymmetric [4 + 1] annulation reaction enabled by Pd-catalyzed desymmetrization of diarylmethyltriflamides with vinyl ketones. In this regard, chemo- and regio-selectivity are notably challenging, as in the absence of *ortho* substitution a mixture of mono- and di-C–H activation could be obtained.^10^ Also, the use of vinyl ketones as a coupling partner is challenging, as a mixture of the olefination product and the corresponding conjugated addition product with poor enantio- and diastereoselectivities could be obtained.^7*b*^ Inspired by previous studies, here, we report our study on the use of a MPAA as a chiral ligand (L9) to enable the unprecedented palladium catalyzed desymmetrization of diarylmethyltriflamides using vinyl ketones to form isoindolines. High enantioselectivities, diastereoselectivities and yields are obtained for the [4 + 1] annulation process and the products of the reaction are privileged scaffolds, which we believe will be of significant interest to practitioners of medicinal chemistry.

**Scheme 1:**
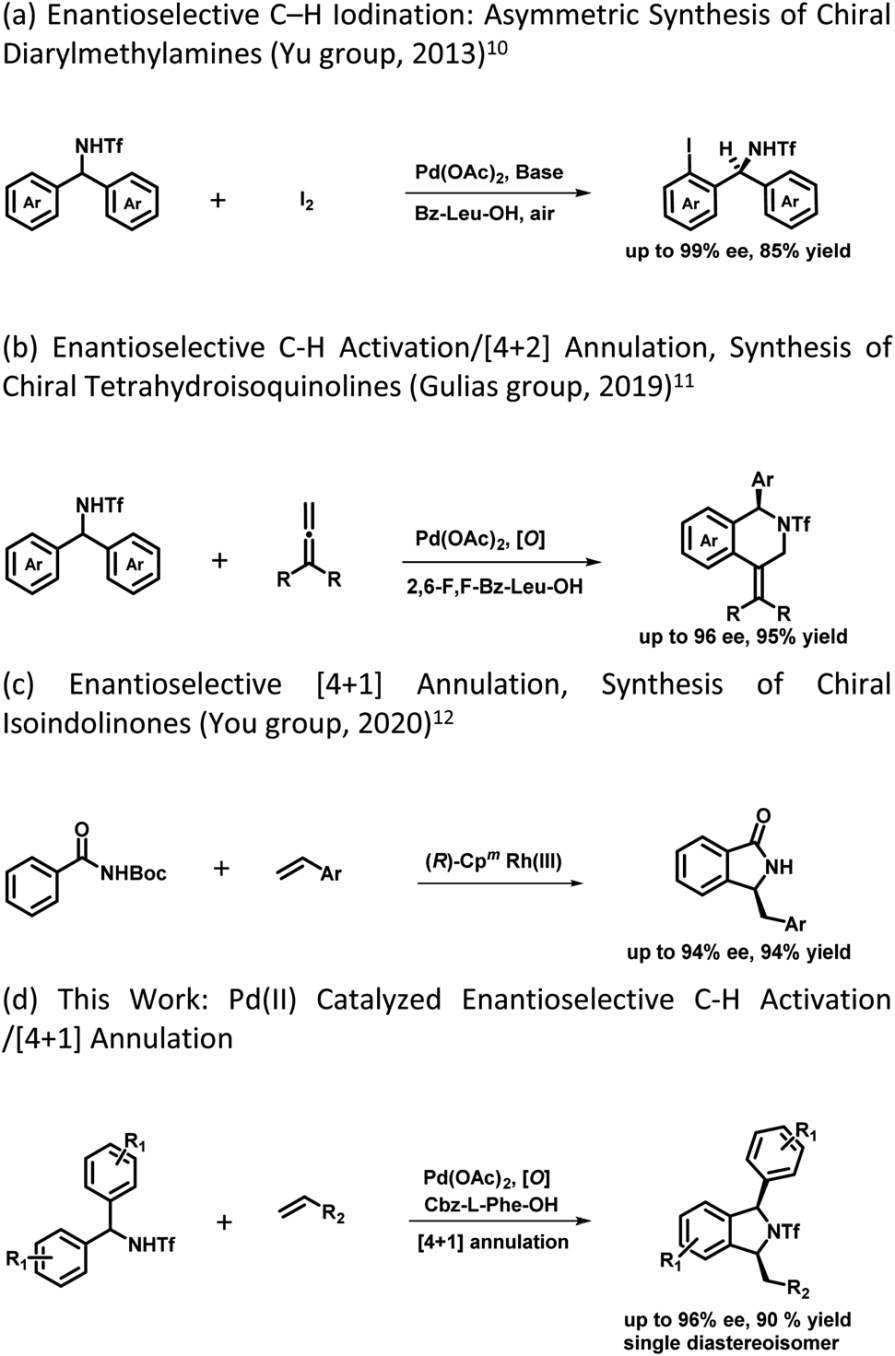
Prior report and this work on desymmetrization and annulation.

**Fig. 1:**
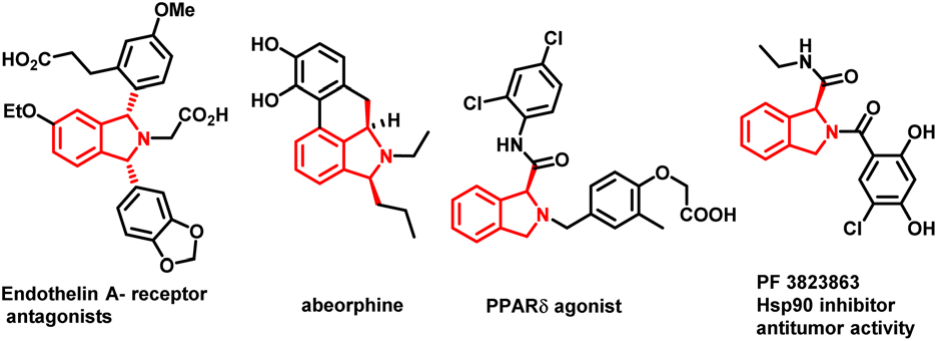
Optically pure biologically active molecule containing the 1,3 disubstituted isoindoline skeleton.

To check the feasibility of our method, we performed a reaction between triflyl-protected diarylmethylamine 1a and methyl vinyl ketone 2a in the presence of Pd(OAc)_2_ (5 mol%), Cs_2_CO_3_ (1.5 equiv.), and Cu(OAc)_2_·H_2_O (2 equiv.) in toluene at 90 °C for 24 h (Scheme 2). The reaction of triflamide directed C–H activation followed by concomitant [4 + 1] annulation afforded the corresponding (+/−)-1,3-disubstituted isoindoline derivative 3a in only 45% yield. To our delight, a significant influence on the reactivity and enantioselectivity was observed when a mono-N-protected amino acid (L1–L20) was used as a chiral ligand with the above reaction conditions. We initially tested the Boc-l-leu-OH ligand using the same reaction conditions that afforded the desired product 3a with 72% yield and a 75 : 25 enantiomeric ratio (er). It confirmed that the MPAA not only acted as a chiral ligand but also showed a strong impact on increasing the reactivity of the palladium catalyst. The temperature effects were examined under the same reaction conditions and we observed 72% yield with an 80 : 20 er at 60 °C. It is important to mention that further variation in the temperature led to noteworthy attenuation in the reaction rate as well as the yield. We also tested l-leucine with different protecting groups such as Boc, Cbz, 2-fluoro-Bz, and 2,6-difluoro-Bz (L1–L4) at 60 °C and found no significant changes in the reaction output except that a mediocre change in the enantiomeric ratio was observed in the case of Boc/Cbz-l-Leu-OH. With this, we tested reaction with some Boc & Cbz N-protected structurally diverse MPAAs (L5–L14). Among them, Cbz-l-Phe-OH (L9) afforded desired product 3a with 85% yield and an excellent 95 : 5 enantiomeric ratio. Subsequently, Boc protected di- and tri-peptides (L15–L17), cinchonine, NOBINAc and L20 were employed for the transformation but none of them improved the reaction yield and enantioselectivity. To further improve the reaction efficacy and enantioselectivity, we tested the reaction using different palladium catalysts, bases and solvents (Table 1). Catalysts such as Pd(TFA)_2_ and Pd(CH_3_CN)_2_Cl_2_ were found to be as effective as Pd(OAc)_2_ but [(PEt_3_)_2_PdCl_2_] showed no catalytic activity (entry 1–4). Although bases such as K_2_CO_3_, Ag_2_CO_3_ and Na_2_CO_3_ yielded the product 3a, there was a decrease in either the yield or the enantiomeric ratio (er: 60 : 40–94 : 6) (entries 5–7). Similarly, polar solvents including DMF and dioxane failed to offer desired transformation; instead the starting material was recovered from the reaction. The reaction in 1,2-dichloroethane afforded the 1,3-disubstituted isoindoline derivative in 70% yield and a 91 : 9 enantiomeric ratio (entry 8–10). Increase in the yield of the product 3a was observed when the reaction was carried out using 10 mol% Pd(OAc)_2_ (entry 11). However, no reaction was observed in the absence of either Cs_2_CO_3_ or Cu(OAc)_2_, which confirmed that the base and oxidant were necessary for this transformation (entries 12 and 13). On the basis of a previous report,^11^ to further enhance the ee and yield of the reaction we also performed reaction under the optimized reaction conditions with the addition of 15 equiv. of DMSO. We observed only a trace amount of desired product and the starting material was recovered (entry 14). Even complete replacement of toluene with DMSO didn't improve the result (entry 15). So far, we found the reaction conditions for (entry 11) to be the best to explore and investigate the substrate scope of this transformation.^16^

**Scheme 2:**
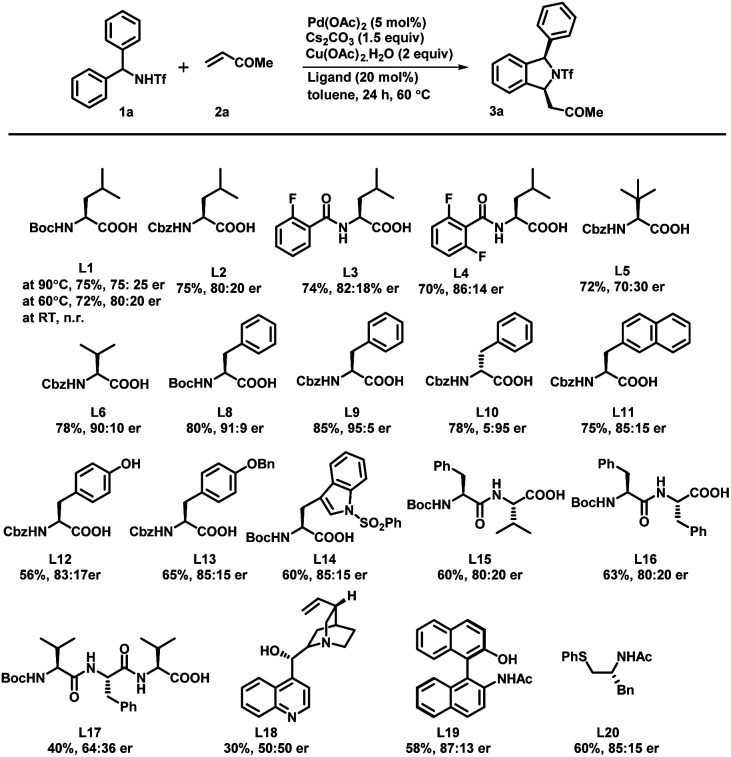
Screening of ligands. ^*a*^Reaction conditions: 1a (0.2 mmol), 2a (0.3 mmol), Pd(OAc)_2_ (5 mol%), ligand (20 mol%), Cs_2_CO_3_ (1.5 equiv.) and Cu(OAc)_2_·H_2_O (2.0 equiv.) at 60 °C in toluene (3.0 mL) under argon. ^*b*^Isolated yields are of the product. ^*c*^Determined by HPLC analysis on a chiral stationary phase.

**Table 1:** Optimization table of reaction conditions^a^

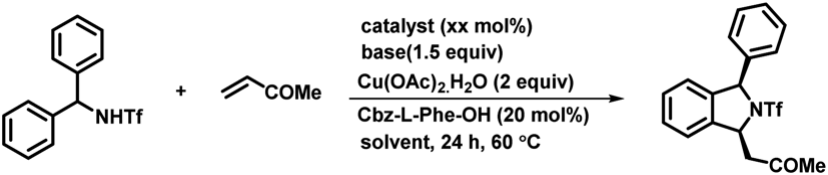					
Entry	Catalyst	Base	Solvent	Yield^b^	er^c^
1	Pd(OAc)_2_	Cs_2_CO_3_	Toluene	85%	95 5
2	Pd(TFA)_2_	Cs_2_CO_3_	Toluene	80%	92 8
3	Pd(CH_3_CN)_2_Cl_2_	Cs_2_CO_3_	Toluene	70%	93 7
4	Pd(PEt_3_)_2_Cl_2_	Cs_2_CO_3_	Toluene	—	—
5	Pd(OAc)_2_	K_2_CO_3_	Toluene	70%	94 6
6	Pd(OAc)_2_	Ag_2_CO_3_	Toluene	68%	60 40
7	Pd(OAc)_2_	Na_2_CO_3_	Toluene	78%	85 15
8	Pd(OAc)_2_	Cs_2_CO_3_	DMF	—	—
9	Pd(OAc)_2_	Cs_2_CO_3_	Dioxane	—	—
10	Pd(OAc)_2_	Cs_2_CO_3_	1,2-DCE	70%	91 9
11^d^	Pd(OAc)_2_	Cs_2_CO_3_	Toluene	90%	95 5
12	Pd(OAc)_2_	—	Toluene	Trace	—
13^e^	Pd(OAc)_2_	Cs_2_CO_3_	Toluene	—	—
14^d^^,^^f^	Pd(OAc)_2_	Cs_2_CO_3_	Toluene	Trace	—
15^d^	Pd(OAc)_2_	Cs_2_CO_3_	DMSO	Trace	—

a Reaction conditions: 1a (0.2 mmol), 2a (0.3 mmol), Pd(OAc)_2_ (5 mol%), ligand (20 mol%), base (1.5 eq.) and Cu(OAc)_2_·H_2_O (2.0 equiv.) at 60 °C in toluene (3.0 mL) under argon.

b Isolated yields are of the product.

c Determined by HPLC analysis on a chiral stationary phase.

d Reaction conducted in the presence of 10 mol% Pd(OAc)_2_.

e Reaction conducted in the absence of Cu(OAc)_2_·H_2_O.

f 15 equiv. of DMSO added.

Under optimized reaction conditions, we investigated the scope of various triflyl-protected diraylmethylamines (1a–1n) with methyl vinyl ketone 2a (Scheme 3). Typically, various substituents at different positions of the aromatic ring were well tolerated without imposing any of their steric or electronic effects. Diarylmethyltriflamide (1a–1e) bearing substituents at the *para*-position such as methyl, ethyl, electron-withdrawing fluoro, and chloro groups successfully underwent an enantioselective C–H activation/[4 + 1] annulation reaction with methyl vinyl ketone to provide the corresponding isoindoline derivatives (3a–3e) with excellent yield (84–90%) and enantiomeric ratios (up to 95 : 5 er). Electron rich substituents at the *para* position such as OMe on diarylmethyltriflamide also showed viability for the transformation in affording the desired product (3f) with good yield (88%) and enantiomeric ratio (94 : 6). Similarly, *meta*-substituents Me and OCF_3_ on diarylmethyltriflamide afforded desired products with a regioselective manner under the influence of the steric effect. In the case of electron rich substrates, the reaction occurred smoothly at the less sterically hindered site providing products 3g with a moderate er (87 : 13) and good yields whereas reaction also occurred at the less hindered site in the case of the electron withdrawing group –OCF_3_ containing substrate 1h afforded product 3h with an excellent er (95 : 5) and moderate yield (75%). Furthermore, sterically hindered and strong electron donating substituents at the *para* position (*tert*-butyl and phenyl) provided desired products 3i and 3j in good yields (81–85%) and an excellent enantiomeric ratio (96 : 4). Gratifyingly, *N*-(di(naphthalen-2-yl)methyl)-1,1,1-trifluoromethanesulfonamide successfully underwent an enantioselective C–H activation/[4 + 1] annulation reaction with methyl vinyl ketone to produce 3k with a good er (92 : 8) and yield. Similar reactivity and enantioselectivity were observed when reactions were performed with disubstituted substrates, which afforded 3l, 3m and 3n with moderate to good yield and good to excellent er. Furthermore, various activated olefins were examined under standard reaction conditions by performing the reaction with compound 1a (Scheme 4). It is noteworthy that different vinyl ketone derivatives, including ethyl and phenyl, successfully reacted with substrate 1a and afforded required products (4b and 4d) with high enantioselectivity and yields. Remarkably, the use of propyl vinyl ketone resulted in the formation of product 4c with good yield and an excellent enantiomeric ratio 98 : 2. It was delightful and interesting to observe that acrylonitrile underwent smooth reaction with 1a, 1b and 1k to afford the corresponding cyclized products 4e, 4f and 4g respectively in moderate yield and good to excellent er. Similarly, acrylamide 2f also reacted efficiently with 1a, 1i, 1d and 1b producing cyclized products 4i, 4j, 4k and 4l with (68–72%) yield and 91 : 9 er. Furthermore, we also performed reaction with various activated olefins such as acrylates, vinyl acetates, vinylsulfones, styrene and vinyl ethers under the optimized reaction conditions but unfortunately these olefinic partners didn't couple to afford the desired product and only the starting material was recovered.

**Scheme 3:**
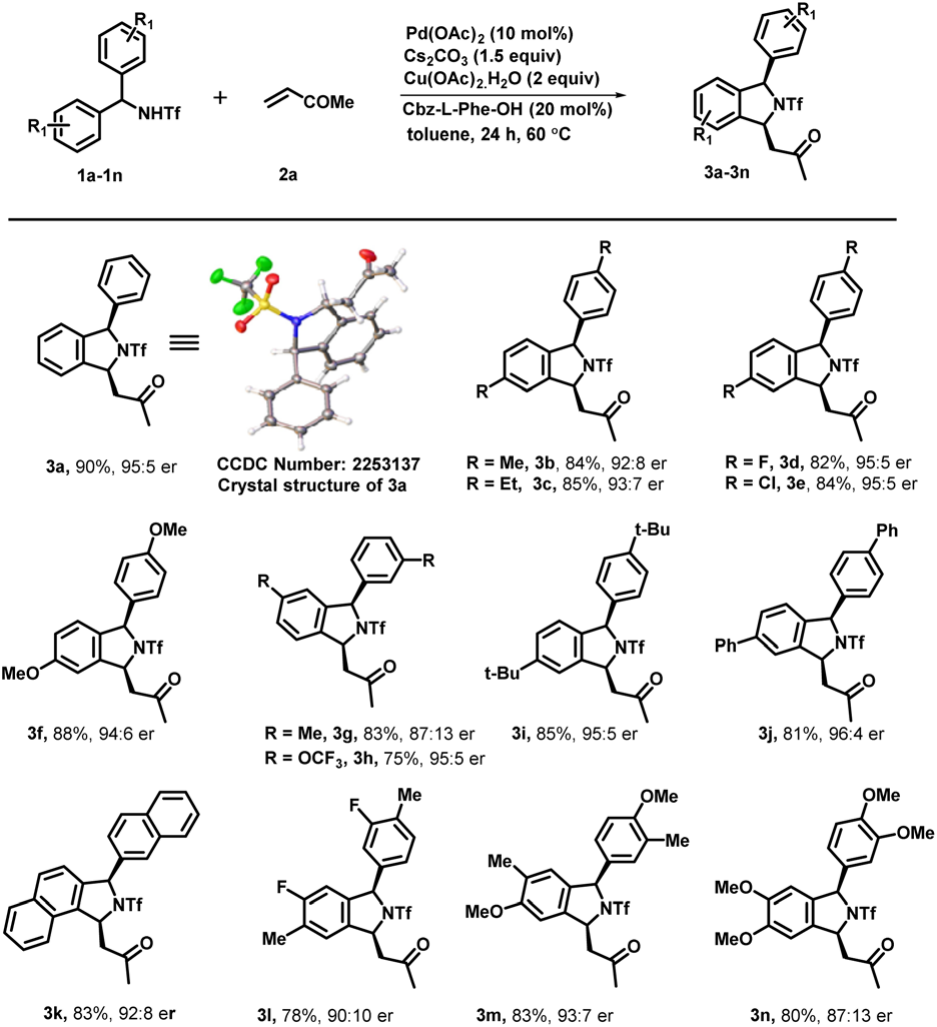
Scope of diarylmethyltriflamide for the enantioselective synthesis of 1,3-disubstituted isoindoline. ^*a*^Reaction conditions: 1 (0.2 mmol), 2a (0.3 mmol), Pd(OAc)_2_ (10 mol%), Cbz-l-Phe-OH (20 mol%), Cs_2_CO_3_ (1.5 equiv.) and Cu(OAc)_2_·H_2_O (2.0 equiv.) at 60 °C in toluene (3.0 mL) under argon. ^*b*^Isolated yields are of the product. ^*c*^Determined by HPLC analysis on a chiral stationary phase.

**Scheme 4:**
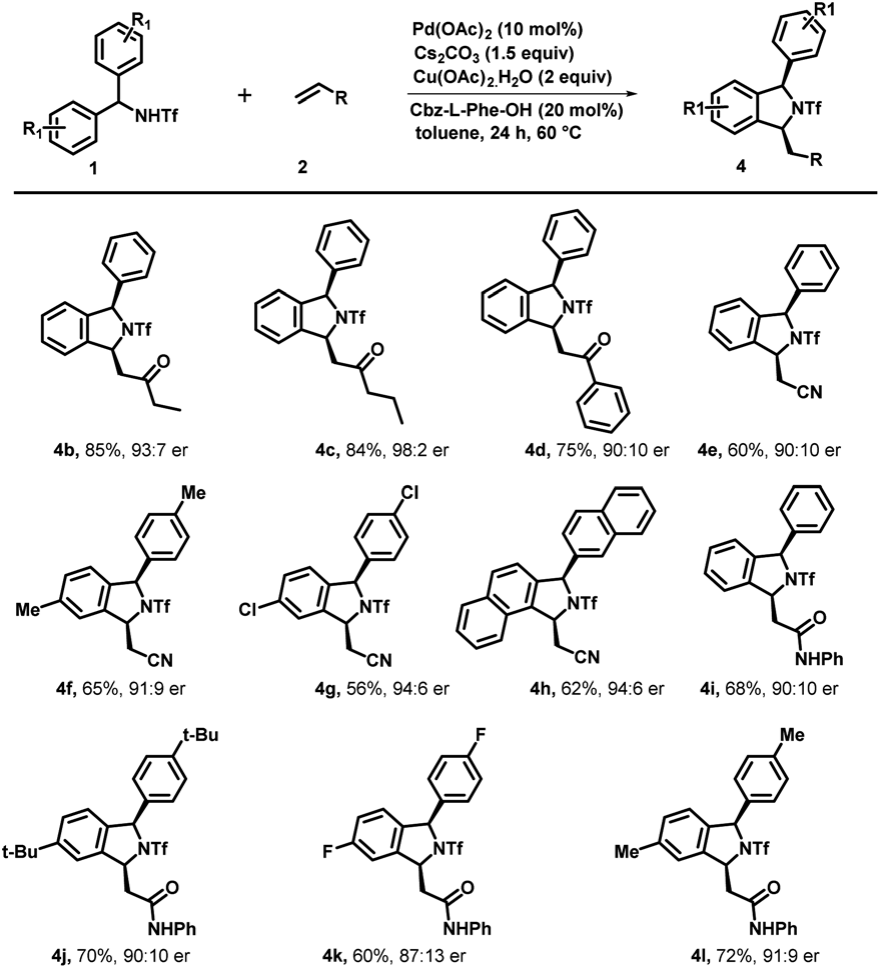
Scope of Olefins. ^*a*^Reaction conditions: 1a (0.2 mmol), 2 (0.3 mmol), Pd(OAc)_2_ (10 mol%), Cbz-l-Phe-OH (40 mol%), Cs_2_CO_3_ (1.5 equiv.) and Cu(OAc)_2_·H_2_O (2.0 equiv.) at 60 °C in toluene (3.0 mL) under argon. ^*b*^Isolated yields are of the product. ^*c*^Determined by HPLC analysis on a chiral stationary phase. [Note] 2f is used (0.2 mmol).

To demonstrate the synthetic utility of our developed enantioselective transformation, we scaled up the reaction by employing diarylmethyltriflamide 1a and methyl vinyl ketone 2a on both the enantiomers of cbz-protected phenylalanine. The corresponding enantioenriched isoindoline derivative 3a was obtained in 85% yield and 94 : 6 er and *e*nt-3a was obtained in 78% yield and 4 : 96 er (Scheme 5a). In order to understand the mechanism of this reaction, we performed some mechanistic studies. We performed a deuterium-labeling experiment with diarylmethyltriflamide 1a and D_2_O under the optimized reaction conditions. The product 1a-*D*_4_ was obtained in 80% yield with 33% deuterium incorporation at four *ortho*-positions of both rings (Scheme 5b). This deuterium scrambling at the *ortho*-position indicates that the C–H activation step was reversible. Furthermore, we examined the reaction of *N*-methyltriflyl-protected diarylmethylamine with methyl vinyl ketone to check whether the NH proton is abstracted *in situ* and then C–H activation occurs. Interestingly, no reaction was observed (Scheme 5c). This result suggests that the NH proton is necessary for this reaction. Furthermore, the five-membered palladacycle intermediate was detected in high-resolution mass spectrometry (HRMS), confirming metalation following C–H bond activation (Scheme 5d).

**Scheme 5:**
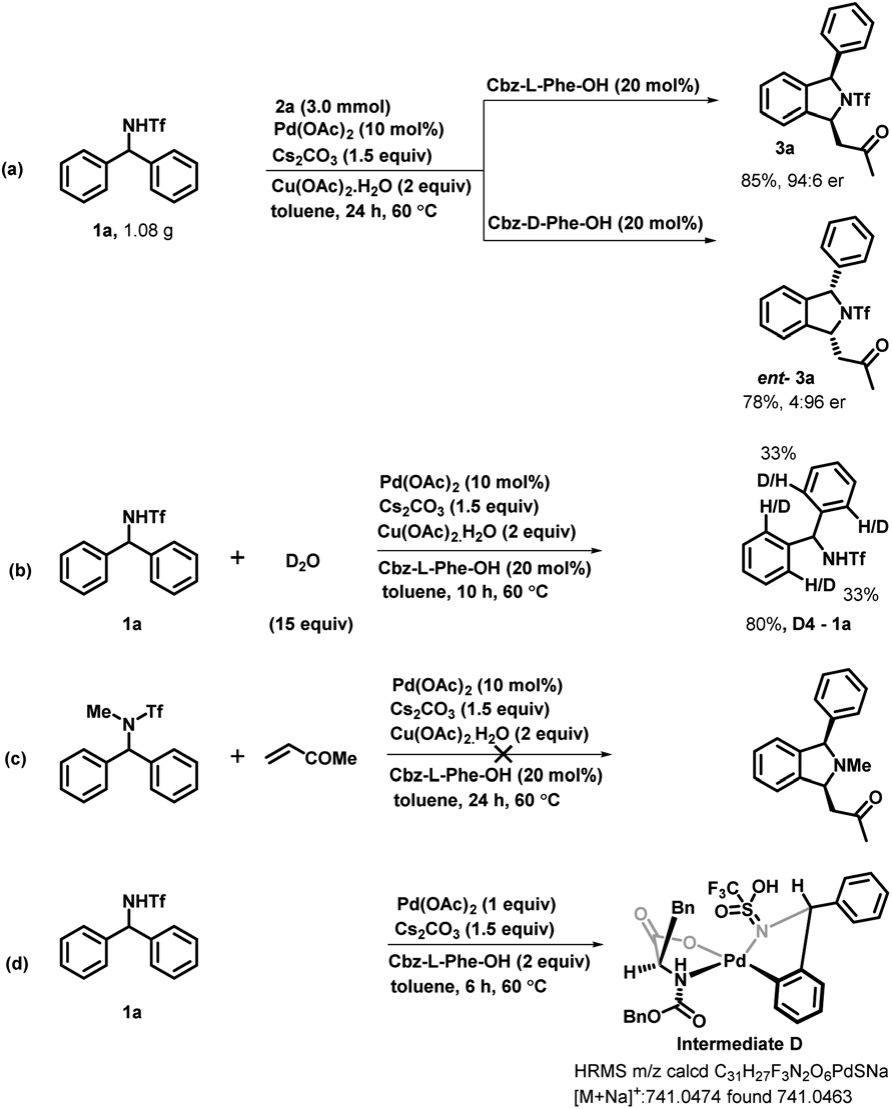
(a–d) Mechanistic studies and gram scale synthesis.

On the basis of previous reports,^2*b*,8,10,11,12,17*a*–*c*^ a plausible mechanism for enantioselective synthesis of *cis*-1,3-disubstituted isoindoline derivatives is depicted in . Initially palladium acetate would react with the MPAA to produce an active species A, which would then react with substrate 1a in the presence of a base affording intermediate B. Enantioselective C–H activation would occur through a proposed transition state C. We propose this transition state on the basis of a previous report^17*a*^ and detection of intermediate D and then the absolute configuration was determined from X-ray crystal structure analysis of product 3a. In this transition state, the N-protecting group is pushed below the Pd coordination plane due to steric repulsion of the amino acid side chain (benzyl group). The substrate needs to coordinate in such a way that the activating C–H points downward to the carbonyl oxygen of the N-protecting group, as this acts as an internal base for proton abstraction. The sp^2^ C–H bond mandates a downward orientation for the prochiral carbon in the coplanar *ortho* position and an upward orientation for the aryl ring. To avoid producing an eclipsed conformation with the reacting upward phenyl group, the downward phenyl group adopts a sterically less hindered axial position in this structure, which ultimately results in an (*R*)-configured product. After enantioselective C–H activation it forms intermediate D, which is confirmed by high-resolution mass spectrometry (HRMS) of 5-membered palladacycle intermediate D. The coordination of olefins followed by alkene migratory 1,2-addition affords a seven-membered palladacycle E, which undergoes *syn* β-H elimination resulting in the formation of intermediate F. Subsequently second migratory insertion of this metal hydride species leads to thermodynamically stable 6-membered palladacycle intermediate G. Finally, intermediate G undergoes reductive elimination to deliver the cyclized product and Pd(0). This Pd(0) could be re-oxidized in the presence of Cu(OAc)_2_ and convert into active species A for a new catalytic cycle (Scheme 6).

**Scheme 6:**
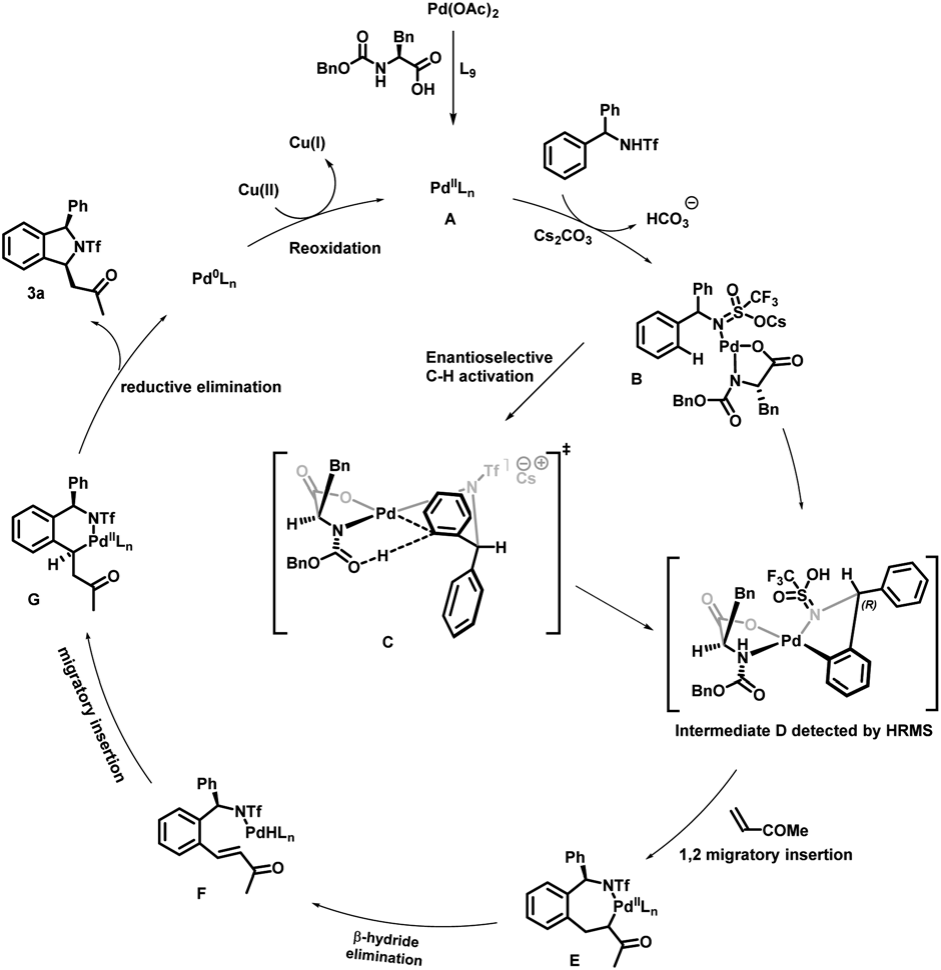
Proposed catalytic cycle.

## Conclusions

In summary, we have developed a triflamide directed palladium(ii) catalysed enantioselective C–H activation/[4 + 1] annulation reaction between diraylmethyltriflamide and activated olefins with the assistance of MPAAs. The developed method provides efficient access to chiral *cis*-1,3-disubstituted isoindoline derivatives. Mild reaction conditions, excellent regio-, diastereo-, and enantioselectivity, broad substrate scope and functional group tolerance proved the versatility of this method.

## Data availability

Further details of the experimental procedure, ^1^H, ^13^C, NMR spectra, HPLC analyses and X-ray crystallographic data for 3a are available in the ESI.†

## Author contributions

D. H. D. directed the project and wrote the manuscript. V. K. wrote the manuscript, prepared the ESI† and conducted most of the synthetic experiments. M. S. has done some of the synthetic experiment.

## Conflicts of interest

The authors declare no competing financial interest.

## Supplementary Material

SC-014-D3SC03496H-s001

SC-014-D3SC03496H-s002
