# Supplementary material for: A palladium catalyzed asymmetric desymmetrization approach to enantioenriched 1,3-disubstituted isoindolines
Source: Chem Sci. 2023 Sep 26;14(40):11267–72. doi: 10.1039/d3sc03496h (PMC10583692; doi:10.1039/d3sc03496h)
Supplement: SC-014-D3SC03496H-s001 [file SC-014-D3SC03496H-s001.pdf]

Electronic Supplementary Material (ESI) for Chemical Science.
This journal is © The Royal Society of Chemistry 2023

Supplementary Information for

Palladium Catalyzed Asymmetric Desymmetrization Approach to Enantioenriched 1,3-disubstituted Isoindolines

Dattatraya H. Dethe*, Vimlesh kumar and Manmohan Shukla

Department of Chemistry, Indian Institute of Technology-Kanpur, Kanpur - 208016, India.
Tel: + 91-512-2596537, fax: + 91-512-2597436.

Contents

1. General information……………………………………………………3

2. Experimental procedure for the preparation of starting material

2.1 General procedure for preparation of Diarylmethyltriflamides……………4

2.2 General procedure for the synthesis of Diarylmethylamine………………4-5

2.3 General procedure for preparation of substrates substituted with F, and Cl…5

2.4 Examples with spectral data……………………………………………….5-6

3. Experimental procedure and characterization of products

3.1 General procedure for Pd(II)-Catalysed racemic synthesis of 1,3 disubstituted isoindoline……………………………………………………………………….6

3.2 General procedure for Pd(II)-Catalysed enantioselective synthesis of 1,3 disubstituted isoindoline…………………………………………………………6

3.3 Gram scale synthesis of (+) enantiomer of 1,3 disubstituted isoindoline…….7

3.4 Gram scale synthesis of (-) enantiomer of 1,3 disubstituted isoindoline…..7-8

3.5 Deuterium labelling experiment……………………………………………8-9

3.6 Detection of intermediate D……………………………………………….10

3.7 Examples with spectral data………………………………………………10-14

4. NMR spectra of starting material 1……………………………………………..15-22

5. NMR spectra of compounds 3 and 4…………………………………………….23-47

6. HPLC spectra of compounds 3 and 4……………………………………………48-72

7. Crystallographic Data of 3a……………………………………………………..73-79

1. General Information

General Aspects: Experiments involving moisture and air-sensitive components were performed in oven-dried glassware. Commercial solvents and reagents were used without further purification unless otherwise noted. Yields refer to chromatographically pure compounds unless otherwise stated. Reactions were monitored by thin-layer chromatography (TLC) carried out on 0.25 mm Merck silica gel plates (60F-254) using UV light as a visualizing agent and a p-anisaldehyde or ninhydrin stain, and heat as developing agents. Merck silica gel (particle size 100-200 and 230-400 mesh) was used for flash column chromatography. Neat compounds were used for record IR spectra. NMR spectra were recorded on either a Bruker Avance 400 (^{1}H, 400 MHz; ^{13}C, 100 MHz), Bruker Avance 500 (^{1}H, 500 MHz; ^{13}C, 125 MHz), or JEOL DELTA (ECX) 500 (^{1}H, 500 MHz; ^{13}C, 125 MHz). Mass spectrometric data were obtained using Agilent-Premier-APCI-MS instruments and IR data recorded from PerkinElmer, FT-IR spectrometer. Optical rotations were measured using a Polarimeter (AUTOPOL II) at 28 °C. The following abbreviations were used to explain the multiplicities: s = singlet, d = doublet, t = triplet, q = quartet, spt= septet, dd = doublet of doublet, ddd = doublet of a doublet of a doublet, dt = doublet of a triplet, td = triplet of a doublet, m = multiplet, br = broad.

Experimental procedure for preparation of starting material:

2.1 General procedure for preparation of Diarylmethyltriflamides.[3]

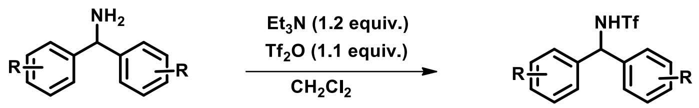

To a stirred solution of diarylmethylamine (5 mmol, 1.0 equiv.) in dichloromethane (20 mL) was added triethylamine (6 mmol, 1.2 equiv) at -78 °C under nitrogen. After stirring for 5 min at -78 °C, trifluoromethanesulfonic anhydride (5.5 mmol, 1.1 equiv.) was added dropwise and the mixture was stirred for 1h at the same temperature before being quenched by water (20 mL). The organic layer was separated and the aqueous layer was extracted with dichloromethane (10 mL × 2). The combined organic phase was washed with brine (20 mL) and then dried over Na_2SO_4. Evaporation and column chromatography on silica gel (ethyl acetate/hexane= 1:20 as eluant) afforded corresponding trifluoromethanesulfonamide.

2.2 General procedure for preparation of diarylmethylamines. [1]

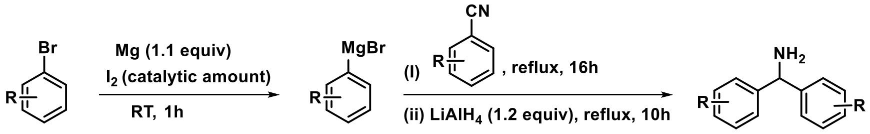

To a stirred solution of Mg (1.1 equiv), I_2 (catalytic amount) in anhydrous THF (20 ml) was added arylbromide (1.0 equiv.) dropwise. The resulting solution was stirred for another 1h at room temperature. Then this solution was added dropwise into corresponding arylnitrile in THF (10 ml) at room temperature. The resulting mixture was heated to reflux for 12h and then allowed to cool to room temperature and then to 0 °C. To this mixture was transferred a suspension of $LiAlH_4$ (20 mmol) in THF (20 mL) via cannula. The ice bath was then removed, and the reaction mixture was heated to reflux, which was maintained for 12h. Upon completion, the mixture was cooled to room temperature, and carefully quenched by slow addition of water (5 ml), The resulting slurry was filtered through a celite pad and washed with DCM until no amine was left. The combined organic layer was washed with sat. aq. NaCl and concentrated under reduced pressure to give the crude amine, which could be used directly in the next step without purification. And the corresponding trifluoromethane sulfonamides **1a, 1c**, **1h**, **1j**, **1k**, **1l**, **1m** and **1n** could be synthesized using the same protocol shown above.

2.3 General procedure for preparation of biarylamine substituted with F and Cl.[1]

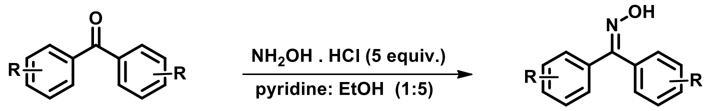

To a 25 ml round bottom flask has added the ketone (1.5 mmol), hydroxylamine hydrochloride (7.5 mmol, 0.52 g), pyridine (1 ml), and EtOH (5 ml). The resulting solution was heated in an oil bath to reflux for 6h. After completion of the reaction, the solvent was removed in vacuo and the residue was partitioned between EtOAc and H_2O (1:1). The aqueous layer was extracted with EtOAc twice, and the combined organic layer was washed with brine and dried over Na_2SO_4. Evaporation afforded the crude oxime, which could be used directly into the next step without purification.

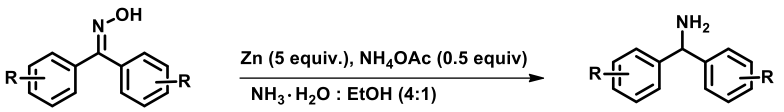

To a stirred suspension of oxime (3 mmol) in EtOH (4 ml) and ammonia solution (ammonium hydroxide solution) (16 ml) was added NH_4OAc (1.5 mmol, 0.12 g), followed by portion-wise addition of zinc powder (15.0 mmol, 0.98 g). The mixture was heated to 50 °C in an oil bath for 1h and then refluxed for 10h. The mixture was cooled to room temperature, diluted with 20 ml of EtOAc, stirred for 30 min, and filtered through filter paper. The filtrate was transferred to a separation funnel. The organic layer was collected, and the aqueous layer was extracted with EtOAc (2 × 20 ml). The combined organic layer was dried over Na_2SO_4 and concentrated under reduced pressure to give the crude amine. And the corresponding trifluoromethanesulfonamides **1d** and **1e** could be synthesized using the same protocol shown above.

Note- Compounds **1a, 1b, 1f, 1g** and **1i** are reported in literature[2,3].

References:

1. Zhang, Y.; Lu, Z.; Desai, A.; Wulff, W. D. *Org. Lett.* 2008, **10**, 5429.

2. Chu, L.; Wang, X.-C.; Moore, C. E.; Rheingold, A. L.; Yu, J.-Q. *J. Am. Chem. Soc.* 2013, **135**, 16344.

3. Vidal, X.; Mascareñas, J. L.; Gulías, M. *J. Am. Chem. Soc.* 2019, **141**, 1862.

2.4 Examples with data.

Compound 1c

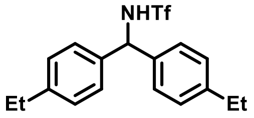

Compound **1c** was obtained as a pale yellow liquid (1.28 g, 3.47 mmol, 83%) ^{1}H NMR (400 MHz, $CDCl_3$) δ 7.24 – 7.02 (m, 8H), 5.81 (s, 1H), 5.41 (brs, 1H) 2.64 (q, *J* = 7.6 Hz, 4H), 1.22 (t, *J* = 7.6 Hz, 6H). ^{13}C NMR (125 MHz, $CDCl_3$) δ 144.46, 137.18, 128.48, 127.20, 119.57 (q, *J* = 321.25 Hz), 62.24, 28.55, 15.46. HRMS (APCI-TOF) m/z calcd. for $C_{18}H_{20}F_3NO_2S$ $[M]^+$ 371.1167; found 371.1166. IR (neat): v_{max}/cm^{-1} 3300, 2958, 2921, 2852, 1604, 1464, 1370, 1390, 1224, 1182, 1054, 603.

Compound 1d

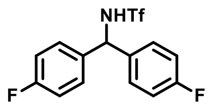

Compound **1d** was obtained as a colorless liquid (1.29 g, 3.66 mmol, 80%) ^{1}H NMR (400 MHz, $CDCl_3$) δ 7.21 (dd, *J* = 7.7, 5.4 Hz, 4H), 7.10 – 6.93 (m, 4H), 6.09 (brs, 1H), 5.83 (s, 1H). ^{13}C NMR (100 MHz, $CDCl_3$) δ 162.61 (d, *J* = 249.47 Hz), 135.35 (d, *J* = 3.1 Hz), 128.99 (d, *J* = 8.2 Hz), 119.46 (q, *J* = 321.26 Hz), 116.10 (d, *J* = 21.3 Hz), 61.28. HRMS (APCI-TOF) m/z calcd. for $C_{14}H_{10}F_5NO_2S$ $[M]^+$ 351.0352; found 351.0345. IR (neat): v_{max}/cm^{-1} 3298, 2925, 2854, 1895, 1606, 1510, 1375, 1231, 1199, 1047, 834, 616.

Compound 1h

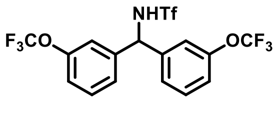

Compound **1h** was obtained as a pale yellow liquid (1.02 g, 2.13 mmol, 75%) ^{1}H NMR (400 MHz, $CDCl_3$) δ 7.42 (t, *J* = 8.0 Hz, 2H), 7.24 – 7.19 (m, 2H), 7.17 (d, *J* = 7.8 Hz, 2H), 7.08 (s, 2H), 5.91 (brs, 1H), 5.87 (s, 1H). ^{13}C NMR (100 MHz, $CDCl_3$) δ 149.79, 141.11, 130.79, 125.58, 121.18, 120.40 (q, *J* = 321.25 Hz),119.39 (q, *J* = 355.25 Hz), 119.88, 61.27. HRMS (APCI-TOF) m/z calcd. for $C_{16}H_{10}F_9NO_4S$ $[M]^+$ 483.0187; found 483.0195. IR (neat): v_{max}/cm^{-1} 3296, 3075, 2927, 1610, 1490, 1453, 1377, 1264, 1145, 610.

Compound 1j

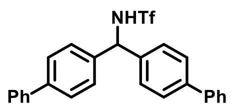

Compound **1j** was obtained as a white solid (1.0 g, 2.15 mmol, 72%) ^{1}H NMR (400 MHz, $CDCl_3$) δ 7.60 (ddd, *J* = 9.4, 7.2, 1.1 Hz, 8H), 7.44 (dd, *J* = 10.8, 4.5 Hz, 4H), 7.39 – 7.33 (m, 6H), 5.96 (d, *J* = 8.7 Hz, 1H), 5.72 (d, *J* = 8.7 Hz, 1H). ^{13}C NMR (100 MHz, $CDCl_3$) δ 141.46, 140.25, 138.55, 128.96, 127.81, 127.73, 127.18, 119.59 (q, *J* = 321.25 Hz), HRMS (APCI-TOF) m/z calcd. for $C_{26}H_{20}F_3NO_2S$ $[M]^+$ 467.1167; found 467.1164. IR (neat): v_{max}/cm^{-1} 3308, 3031, 2925, 1600, 1487, 1374, 1230, 1144, 1039, 1007, 764.

Compound 1k

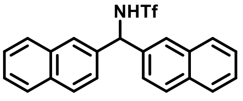

Compound **1k** was obtained as a white solid (996mg, 2.40 mmol, 68%) ^{1}H NMR (500 MHz, $CDCl_3$) δ 7.86 – 7.79 (m, 6H), 7.76 (s, 2H), 7.54 – 7.48 (m, 4H), 7.35 (dd, *J* = 8.6, 1.7 Hz, 2H), 6.20 (d, *J* = 8.9 Hz, 1H), 5.83 (d, *J* = 8.7 Hz, 1H). ^{13}C NMR (100 MHz, $CDCl_3$) δ 136.88, 133.22, 133.09, 129.19, 128.28, 127.83, 126.85, 126.45, 124.95, 119.61(q, *J* = 321.25 Hz), 62.82. HRMS (APCI-TOF) m/z calcd. for $C_{22}H_{16}F_3NO_2S$ $[M]^+$ 415.0854; found 415.0856. IR (neat): v_{max}/cm^{-1} 3306, 3058, 2927, 2250, 1601, 1508, 1428, 1374, 1198, 1230, 1198, 1046, 609.

Compound 1l

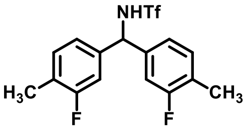

Compound **1l** was obtained as a white solid (1.1g, 2.96 mmol, 74%) ^{1}H NMR (400MHz, $CDCl_3$) δ 7.02 (dd, *J* = 8.4, 2.4 Hz, 2H), 6.97 (d, *J* = 2.1 Hz, 2H), 6.78 (d, *J* = 8.4 Hz, 2H), 5.71 (d, *J* = 6.4 Hz, 1H), 5.29 (s, 1H), 3.81 (s, 6H), 2.17 (s, 6H). ^{13}C NMR (125 MHz, $CDCl_3$) δ 161.45 (d, *J* = 247.12 Hz), 138.96 (d, *J* = 6.3 Hz), 132.16 (d, *J* = 6.3 Hz), 125.42(d, *J* = 16.38 Hz), 122.49, 119.11 (q, *J* = 321.26 Hz) 113.95 (d, *J* = 21.3 Hz), 61.28, 14.34.HRMS (APCI-TOF) m/z calcd. for $C_{16}H_{14}F_5NO_2S$ $[M]^+$ 379.0665; found 379.0668. IR (neat): v_{max}/cm^{-1} 3300, 2957, 2928, 1991, 1627, 1584, 1510, 1454, 1230, 1198, 1052, 969, 633.

Compound 1m

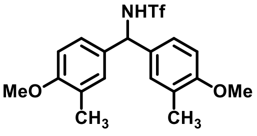

Compound **1m** was obtained as a pale yellow liquid (1.01g, 2.50 mmol, 34%) ^{1}H NMR (400 MHz, $CDCl_3$) δ 7.01 (dd, *J* = 8.4, 2.2 Hz, 2H), 6.96 (d, *J* = 1.9 Hz, 2H), 6.77 (d, *J* = 8.4 Hz, 2H), 5.70 (s, 1H), 5.32 (s, 1H), 3.81 (s, 6H), 2.17 (s, 6H). ^{13}C NMR (100 MHz, $CDCl_3$) δ 157.61, 131.73, 129.46, 127.33, 125.62, 109.93, 61.83, 55.43, 16.41. HRMS (APCI-TOF) m/z calcd. for $C_{18}H_{19}F_3NO_4S$ $[M-H]^+$ 402.0992; found 402.1008. IR (neat): v_{max}/cm^{-1} 343, 2954, 2854, 2836, 2060, 1609, 1503, 1464, 1375, 1253, 1131, 1035, 620.

Compound 1n

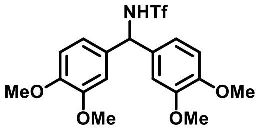

Compound **1n** was obtained as a pale yellow liquid (400mg, 0.924 mmol, 28%) ^{1}H NMR (400 MHz, $CDCl_3$) δ 6.82 (d, *J* = 8.3 Hz, 2H), 6.78 (d, *J* = 2.0 Hz, 1H), 6.75 (d, *J* = 1.9 Hz, 1H), 6.72 (d, *J* = 1.9 Hz, 2H), 5.76 (d, *J* = 8.6 Hz, 1H), 5.66 – 5.52 (m, 1H), 3.85 (s, 6H), 3.79 (s, 6H). ^{13}C NMR (100 MHz, $CDCl_3$) δ 149.32, 149.04, 132.26, 119.59 (q, *J* = 321.26 Hz), 119.65, 111.24, 110.49, 62.08, 56.01, 55.98. HRMS (APCI-TOF) m/z calcd. for $C_{18}H_{21}F_3NO_5S$ $[M+H]^+$ 420.1093; found 420.1099. IR (neat): v_{max}/cm^{-1} 3456, 2957, 294, 2055, 1639, 1516, 1375, 1228, 1191, 1026, 606.

3. Experimental procedure and characterization of products

3.1 General procedure for Pd(II)-Catalysed Racemic Synthesis of 1,3 disubstituted isoindoline

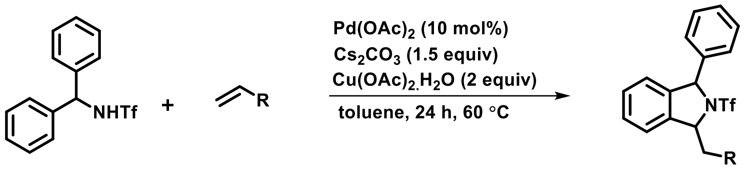

A 5 mL vial was charged with $Pd(OAc)_2$ (4.5 mg, 0.20 mmol, 10 mol%), $Cu(OAc)_2 \cdot H_2O$ (79.6 mg, 0.40 mmol, 2.0 equiv), Cs_2CO_3 (97.5 mg, 0.3 mmol, 1.5 equiv), toluene (3.0 mL) and then the reaction mixture was stirred at room temperature for 10 min under a nitrogen atmosphere. Then diarylmethyltriflamide **1a** (0.2 mmol, 1.0 equiv) and activated olefin **2** (0.3mmol, 1.5 equiv) were added into the solution in sequence. The vial was sealed under nitrogen and heated to 60 °C (using an oil bath) with stirring for 24h. After cooling down, the reaction mixture was diluted with ethyl acetate and concentrated to give the crude compound which was directly purified by column chromatography.

3.2 General procedure for Pd(II)-Catalysed Enantioselective Synthesis of 1,3 disubstituted isoindoline

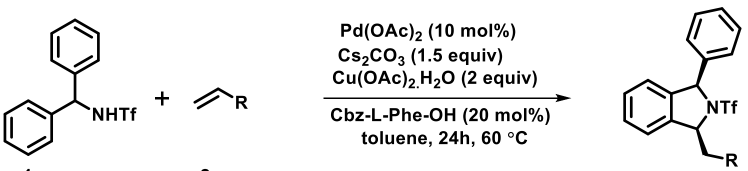

1a 2a 3a

A 5 mL vial was charged with $Pd(OAc)_2$ (4.5 mg, 0.020 mmol, 10 mol%), $Cu(OAc)_2 \cdot H_2O$ (79.6 mg, 0.40 mmol, 2.0 equiv), Cs_2CO_3 (97.5 mg, 0.3 mmol, 1.5 equiv.), Cbz-L-Phe-OH (12 mg, 0.04 mmol, 0.20 equiv), toluene (2.0 mL), and then the reaction mixture was stirred at room temperature for 10 min under a nitrogen atmosphere. Then diarylmethyltriflamide **1a** (0.20 mmol, 1.0 equiv) and activated olefin **2** (0.3mmol, 1.5 equiv) were added into the solution in sequence. The vial was sealed under nitrogen and heated to 60 °C (using an oil bath) with stirring for 24h. After cooling down, the reaction mixture

was diluted with ethyl acetate and concentrated to give the crude compound which was directly purified by column chromatography.

3.3 Gram scale reaction for synthesis of (+) enantiomer of 1,3 disubstituted isoindoline

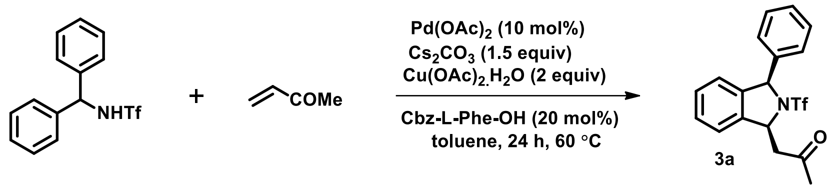

1a, 1.0 g **2a** **3a, 85%, 94:6 er**

A 50 mL screw-cap vial was charged with $Pd(OAc)_2$ (72 mg, 0.32 mmol, 10 mol%), $Cu(OAc)_2{\cdot}H_2O$ (1.27 g, 6.4 mmol, 2.0 equiv), Cs_2CO_3 (1.04 g, 4.80 mmol 1.5 equiv), Cbz-L-Phe-OH (127mg, 0.4 mmol, 20 mol%), toluene (20 mL) and then the reaction mixture was stirred at room temperature for 30 min under a nitrogen atmosphere. Then diarylmethyltriflamides **1a** (3.2 mmol, 1.0 equiv) and methyl vinyl ketone **2a** (4.8 mmol, 1.5 equiv) were added into the solution in sequence. The vial was sealed under nitrogen and heated to 60 °C (using an oil bath) with stirring for 24h. After cooling down, the reaction mixture was diluted with ethyl acetate and concentrated to give the crude compound which was directly purified by column chromatography to afford desired product **3a** (1.04 g, 2.7 mmol, 85 %). HPLC analysis (Chiralpak IC-3; *n*-Hexane/*i*-PrOH =95/5, 0.9 ml/min, 254 nm): t_r (major) = 5.2 min, t_r (minor) = 5.85 min, 94:6 er.

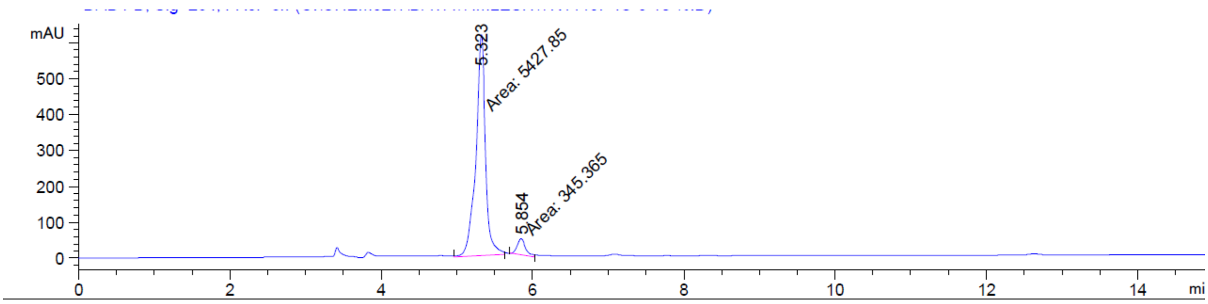

Peak #	RetTime [min]	Type	Width [min]	Area [mAU*s]	Height [mAU]	Area %
1	5.323	MM	0.1467	5427.85205	616.64771	94.0178
2	5.854	MM	0.1297	345.36548	44.36584	5.9822

3.4 Gram scale synthesis of (-) enantiomer of 1,3 disubstituted isoindoline

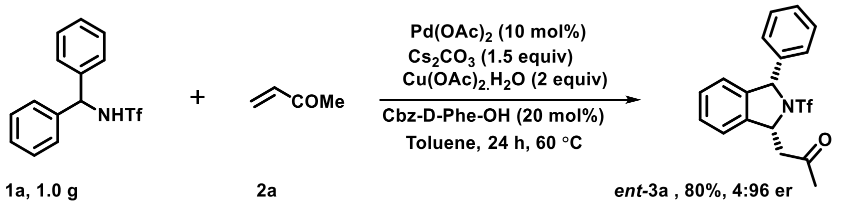

1a, 1.0 g **2a** ***ent*-3a , 80%, 4:96 er**

A 50 mL screw-cap vial was charged with $Pd(OAc)_2$ (72 mg, 0.32 mmol, 10 mol%), $Cu(OAc)_2{\cdot}H_2O$ (1.27 g, 6.4 mmol, 2.1 equiv), Cs_2CO_3 (1.04 g, 4.80 mmol 1.5 equiv), Cbz-D-Phe-OH (127mg, 0.4 mmol, 20 mol%), toluene (20 mL) and then the reaction mixture was stirred at room temperature for 30 min under a nitrogen atmosphere. Then diarylmethyltriflamides **1a** (3.2 mmol, 1.0 equiv) and methyl vinyl ketone **2a** (4.8 mmol, 1.5 equiv) were added into the solution in sequence. The vial was sealed under nitrogen and heated to 60 °C (using an oil bath) with stirring for 24h. After cooling down, the reaction

mixture was diluted with ethyl acetate and concentrated to give the crude compound which was directly purified by column chromatography to afford desired product ***ent*-3a** (980mg, 2.56 mmol, 80%). HPLC analysis (Chiralpak IC-3; *n*-Hexane/*i*-PrOH = 95/5, 0.9 ml/min, 254 nm): t_r (major) = 7.51 min, t_r (minor) = 6.36 min, 4:96 er.

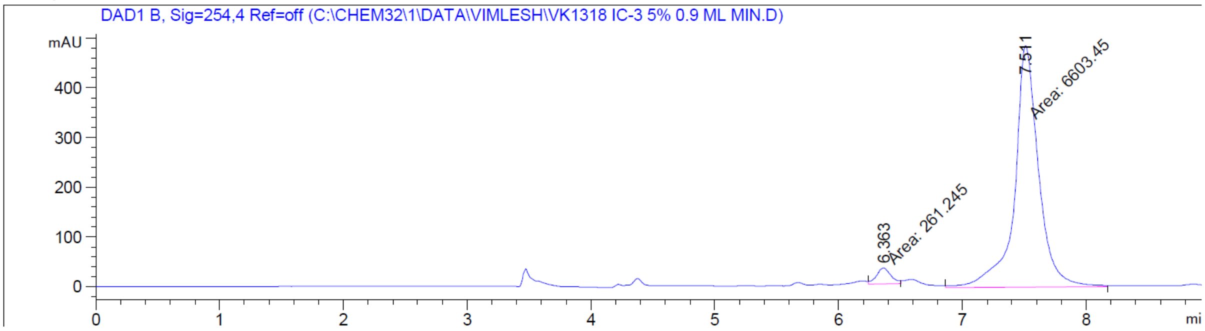

Peak #	RetTime [min]	Type	Width [min]	Area [mAU*s]	Height [mAU]	Area %
1	6.363	MM	0.1357	261.24463	32.08454	3.8056
2	7.511	MM	0.2263	6603.45459	486.28693	96.1944

3.5 Deuterium labelling experiment.

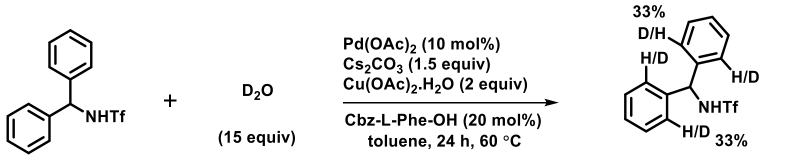

D4 - 1a

A 10 mL screw-cap vial was charged with $Pd(OAc)_2$ (4.5 mg, 0.20mmol, 10 mol%), $Cu(OAc)_2{\cdot}H_2O$ (79.6 mg, 0.40 mmol, 2.0 equiv), Cs_2CO_3 (97.5 mg, 0.3mmol, 1.5 equiv.), Cbz-L-Phe-OH (12mg, 0.04mmol, 0.20 equiv), toluene (2.0 mL) and then the reaction mixture was stirred at room temperature for 10 min under a nitrogen atmosphere. Then diarylmethyltriflamides **1a** (0.20 mmol, 1.0 equiv) and D_2O (3.0 mmol, 15 equiv) were added into the solution in sequence. The vial was sealed under nitrogen atmosphere and heated to 60 °C (using an oil bath) with stirring for 10h. After cooling down, the reaction mixture was diluted with ethyl acetate and concentrated to give the crude compound which was directly purified by column chromatography to afford desired product **D4-1a in** 78% (49 mg, 0.156mmol, 78%).

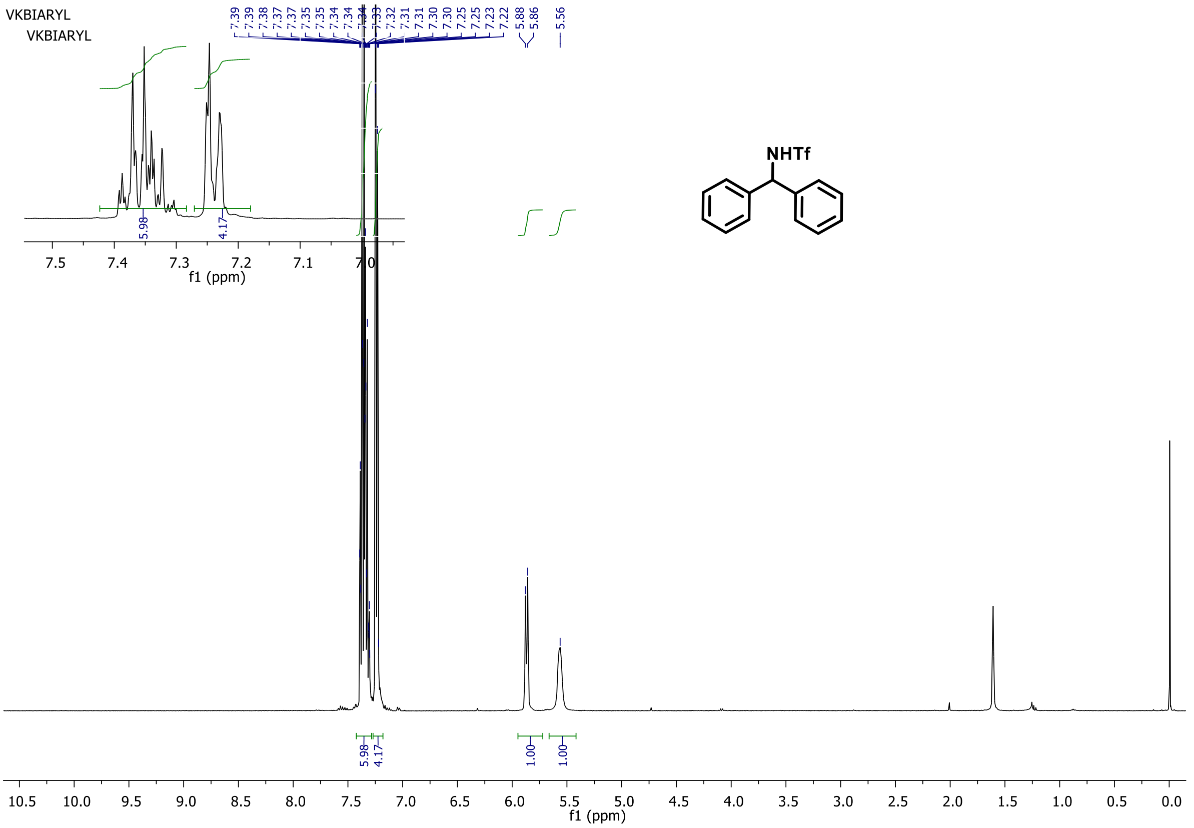
VKBIARYL
VKBIARYL
7.39 7.39 7.38 7.37 7.37 7.35 7.35 7.34 7.34 7.33 7.33 7.32 7.31 7.30 7.30 7.25 7.25 7.23 7.22
5.88 5.86
5.56
NHTf
5.98 4.17
1.00 1.00
f1 (ppm)

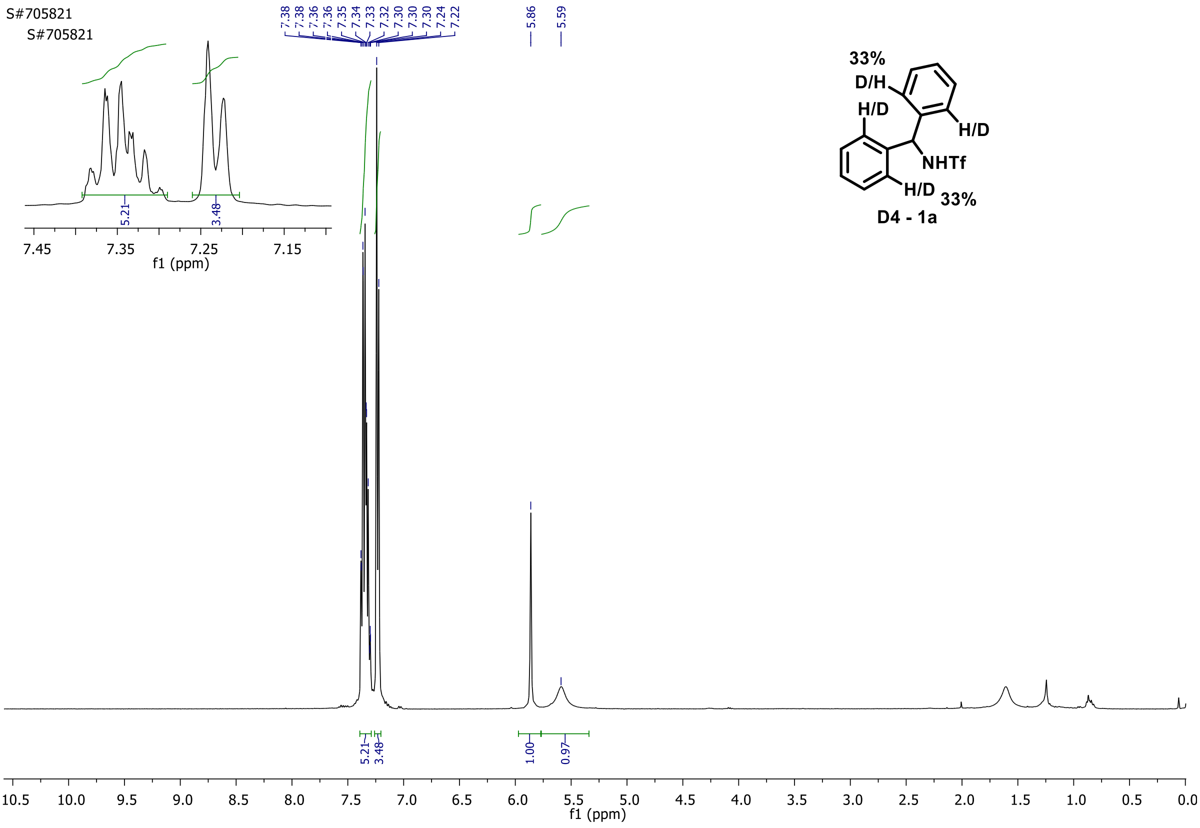
S#705821
S#705821
7.38 7.38 7.36 7.35 7.34 7.33 7.32 7.30 7.30 7.24 7.22
5.86
5.59
33%
D/H
H/D
H/D
NHTf
H/D 33%
D4 - 1a
5.21 3.48
1.00 0.97
f1 (ppm)

3.6 Detection of intermediate D.

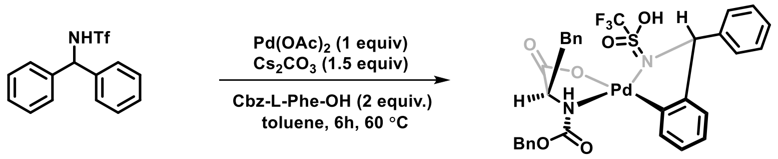

HRMS m/z calcd $C_{31}H_{27}F_{3}N_{2}O_{6}PdSNa$ $[M+Na]^{+}$:741.0474 found 741.0463

A screw-cap 10 mL vial was charged with $Pd(OAc)_2$ (45 mg, 0.20 mmol, 1.0 equiv), Cs_2CO_3 (97.5 mg, 0.30 mmol, 1.5 equiv.), Cbz-L-Phe-OH (120 mg, 0.40 mmol, 2.0 equiv), toluene (2.0 mL) and then the reaction mixture was stirred at room temperature for 10 min under a nitrogen atmosphere. Then diarylmethyltriflamide **1a** (0.20 mmol, 1.0 equiv) was added into the solution. The vial was sealed under a nitrogen and heated to 60 °C (using an oil bath) with stirring for 6h. After cooling down, the reaction mixture was filtered through a celite pad and concentrated to give the crude compound which was directly submitted for HRMS.

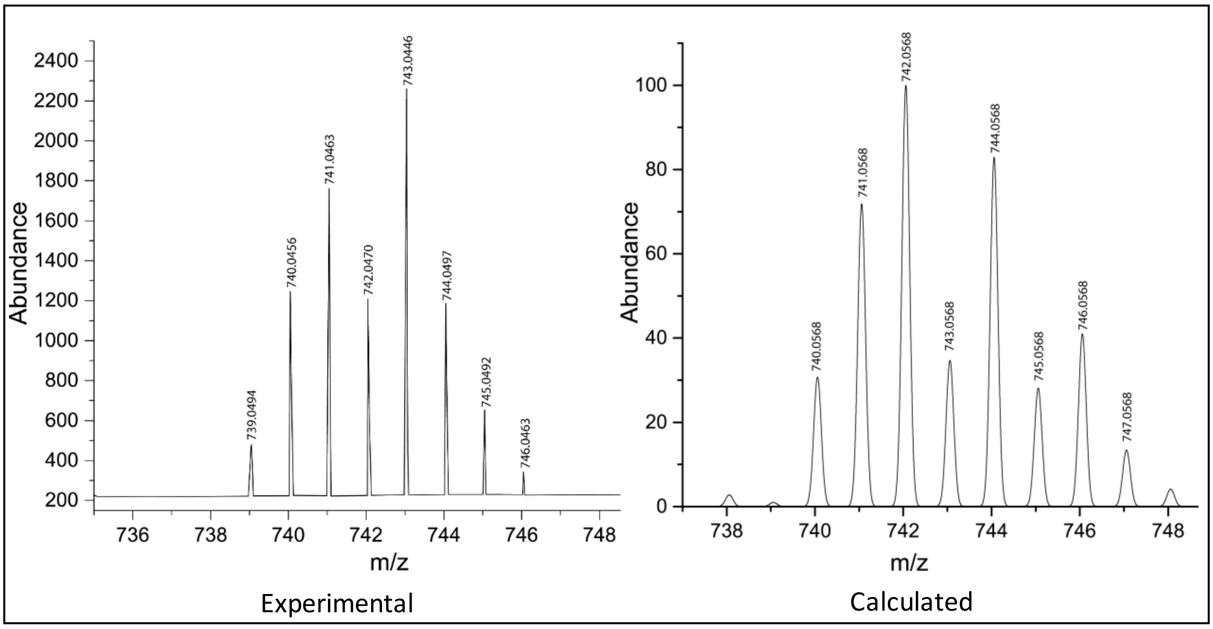

3.7 Examples with data

Compound 3a Following the general procedure, Compound **3a** was obtained as a white crystalline solid (68.9 mg, 0.18 mmol, 90%) Melting point = 119 °C. ^{1}H NMR (500 MHz, $CDCl_3$) δ 7.41 – 7.26 (m, 8H), 7.05 (d, *J* = 7.1 Hz, 2H), 6.17 (s, 1H), 5.85 (d, *J* = 8.8 Hz, 1H), 3.43 (dd, *J* = 17.8, 2.1 Hz, 1H), 3.04 (dd, *J* = 17.9, 9.7 Hz, 1H), 2.21 (s, 3H). ^{13}C NMR (125 MHz, $CDCl_3$) δ 205.13, 140.67, 138.87, 138.34, 129.11, 129.03, 128.92, 128.74, 127.77, 123.77, 123.43, 70.07, 62.45, 52.89, 30.59. HRMS (APCI-TOF) m/z calcd. for $C_{18}H_{17}F_{3}NO_{3}S$ $[M+H]^{+}$ 384.0881; found 384.0882. IR (neat): v_{max}/cm^{-1} 3035, 2957, 2922, 2851, 1717, 1392, 1364, 1226, 1189, 1056, 599. HPLC analysis (Chiralpak IC-3; *n*-Hexane/*i*-PrOH = 95/5, 0.9 ml/min, 254 nm): t_r (major) = 6.21 min, t_r(minor) = 7.02 min, 95:5 er. $[\alpha]_D^{30}$ = +15.03 (c = 0.13 in $CHCl_3$).

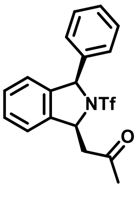

Compound 3b Following the general procedure, Compound **3b** was obtained as a colorless liquid (69 mg, 0.17 mmol, 84%) ^{1}H NMR (500 MHz, $CDCl_3$) δ 7.18 – 7.06 (m, 6H), 6.91 (d, *J* = 7.8 Hz, 1H), 6.08 (s, 1H), 5.76 (d, *J* = 8.6 Hz, 1H), 3.38 (dd, *J* = 17.8, 2.2 Hz, 1H), 3.00 (dd, *J* = 17.9, 9.7 Hz, 1H), 2.34 (d, *J* = 2.3 Hz, 6H), 2.20 (s, 3H). ^{13}C NMR (126 MHz, $CDCl_3$) δ 205.24, 139.14, 139.09, 138.54, 137.99, 135.65, 129.94, 129.53, 127.74, 123.67, 123.45, 69.70, 62.23, 53.07, 30.59, 21.45, 21.23. HRMS (APCI-TOF) m/z calcd. for $C_{20}H_{21}F_{3}NO_{3}S$ $[M+H]^{+}$412.1194; found 412.1184. IR (neat): v_{max}/cm^{-1} 3054, 2956, 2869, 1718, 1616, 1592, 1459, 1392, 1056, 863. HPLC analysis (Chiralpak IC-3; *n*-Hexane/*i*-PrOH = 95/5, 0.9 ml/min, 254 nm): t_r (major) = 5.67 min, t_r(minor) = 7.42 min, 92:8 er. $[\alpha]_D^{30}$ = +13.33 (c = 0.15 in $CHCl_3$).

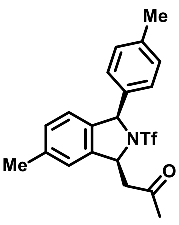

Compound 3c Following the general procedure, Compound **3c** was obtained as a colorless liquid (73 mg, 0.17 mmol, 85%) ^{1}H NMR (500 MHz, $CDCl_3$) δ 7.20 – 7.15 (m, 5H), 7.13 (d, *J* = 7.9 Hz, 1H), 6.95 (d, *J* = 7.9 Hz, 1H), 6.09 (s, 1H), 5.78 (d, *J* = 8.8 Hz, 1H), 3.40 (dd, *J* = 17.8, 2.3 Hz, 1H), 3.01 (dd, *J* = 17.8, 9.6 Hz, 1H), 2.67 – 2.62 (m, 4H), 2.21 (s, 3H), 1.22 (td, *J* = 7.6, 5.2 Hz, 6H). ^{13}C NMR (125 MHz, $CDCl_3$) δ 205.28, 145.54, 144.74, 139.07, 138.19, 135.88, 128.79, 128.31, 127.74, 123.54, 122.49, 69.76, 62.30, 53.08, 30.64, 28.84, 28.58, 15.64, 15.38. HRMS (APCI-TOF) m/z calcd. for $C_{22}H_{25}F_3NO_3S$[M+H]$^+$ 440.1507; found 440.1518. IR (neat): v_{max}/cm^{-1} 3052, 2960, 2854, 1716, 1617, 1590, 1460, 1390, 1060, 897. HPLC analysis (Chiralpak IC-3; *n*-Hexane/*i*-PrOH = 90/10, 0.9 ml/min, 254 nm): t_r (major) = 4.61 min, t_r(minor) = 5.82 min, 93:7 er. $[\alpha]_D^{30}$ = +10.00 (c = 0.10 in $CHCl_3$).

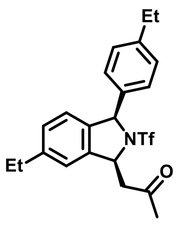

Compound 3d Following the general procedure, Compound **3d** was obtained as a yellow liquid (69 mg, 0.16 mmol, 82 %) ^{1}H NMR (400 MHz, $CDCl_3$) δ 7.40 – 7.16 (m, 2H), 7.13 – 7.07 (m, 1H), 7.07 – 6.87 (m, 4H), 6.09 (s, 1H), 5.75 (d, *J* = 9.4 Hz, 1H), 3.39 (dd, *J* = 18.1, 1.9 Hz, 1H), 2.98 (dd, *J* = 18.2, 9.9 Hz, 1H), 2.20 (s, 3H). ^{13}C NMR (126 MHz, $CDCl_3$) δ 204.92, 164.29, 163.91, 162.32, 161.93, 140.96, 136.27, 133.68, 129.79, 129.11, 129.04, 125.29, 125.22, 116.83, 116.65, 116.04, 115.87, 111.04, 110.84, 68.77, 62.09, 52.50, 30.41. HRMS (APCI-TOF) m/z calcd. for $C_{18}H_{15}F_5NO_3S$ [M+H]$^+$ 420.0693; found 420.0686. IR (neat): v_{max}/cm^{-1} 3033, 2924, 2854, 1716, 1605, 1510, 1491, 1392, 1055, 866. HPLC analysis (Chiralpak IC-3; *n*-Hexane/*i*-PrOH = 98/2, 0.9 ml/min, 254 nm): t_r (major) = 8.27 min, t_r(minor) = 7.72 min, 95:5 er. $[\alpha]_D^{30}$ = +12.00 (c = 0.08 in $CHCl_3$).

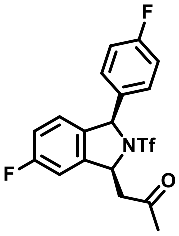

Compound 3e Following the general procedure, Compound **3e** was obtained as a colorless liquid (75 mg, 0.17 mmol, 84 %) ^{1}H NMR (500 MHz, $CDCl_3$) δ 7.40 (s,1H), 7.36 – 7.32 (m, 2H), 7.29 (dd, *J* = 8.2, 1.8 Hz, 1H), 7.21 – 7.15 (m, 2H), 6.95 (d, *J* = 8.1 Hz, 1H), 6.07 (s, 1H), 5.76 (d, *J* = 9.1 Hz, 1H), 3.40 (d, *J* = 17.8 Hz, 1H), 2.98 (dd, *J* = 18.2, 9.9 Hz, 1H), 2.22 (s, 3H). ^{13}C NMR (125 MHz, $CDCl_3$) δ 204.89, 140.71, 138.63, 137.73, 135.48, 135.06, 134.76, 129.64, 129.42, 129.27, 128.62, 124.88, 124.02, 68.84, 62.03, 61.28, 52.52, 30.44. HRMS(APCI-TOF) m/z calcd. for $C_{18}H_{15}Cl_2F_3NO_3S$ [M+H]$^+$ 452.0102; found 452.0108. IR (neat): v_{max}/cm^{-1} ,2957, 2926, 2854, 1716, 1604, 1492, 1393, 1227, 1154, 1055, 862. HPLC analysis (Chiralpak IC-3; *n*-Hexane/*i*-PrOH = 99/1, 0.5 ml/min, 254 nm): t_r (major) = 15.57 min, t_r(minor) = 17.33 min, 95:5 er. $[\alpha]_D^{30}$ = +4.0 (c = 0.25 in $CHCl_3$).

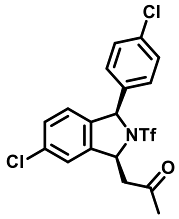

Compound 3f Following the general procedure, Compound **3f** was obtained as a colorless liquid (78 mg, 0.18 mmol, 88 %) ^{1}H NMR (400 MHz, $CDCl_3$) δ 7.15 (dd, *J* = 9.1, 2.4 Hz, 2H), 6.97 – 6.79 (m, 5H), 6.05 (s, 1H), 5.74 (t, *J* = 13.4 Hz, 1H), 3.79 (s, 3H), 3.78 (s, 3H), 3.35 (dd, *J* = 18.0, 2.7 Hz, 1H), 2.97 (dd, *J* = 18.0, 9.7 Hz, 1H), 2.19 (s, 3H). ^{13}C NMR (125 MHz, $CDCl_3$) δ 205.31, 160.49, 159.84, 140.48, 132.30, 130.44, 129.30, 124.63, 115.99, 114.14, 107.72, 69.22, 62.18, 55.62, 55.37, 53.03, 30.57. HRMS (APCI-TOF) m/z calcd. for $C_{20}H_{21}F_3NO_3S$ [M+H]$^+$ 444.1093; found 444.1091. IR (neat): v_{max}/cm^{-1} 3006, 2950, 2848, 1717, 1616, 1575, 1492, 1392, 1230, 872. HPLC analysis (Chiralpak IC; *n*-Hexane/*i*-PrOH = 90/10, 0.9 ml/min, 254 nm): t_r (major) = 6.34 min, t_r(minor) = 7.54 min, 94:6 er. $[\alpha]_D^{30}$ = +13.33 (c = 0.15 in $CHCl_3$).

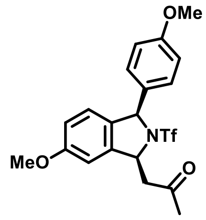

Compound 3g Following the general procedure, Compound **3g** was obtained as a pale yellow liquid (68 mg, 0.16 mmol, 83 %) ^{1}H NMR (400 MHz, $CDCl_3$) δ 7.23 (dt, *J* = 7.9, 4.4 Hz, 2H), 7.14 – 7.09 (m, 3H), 7.00 (d, *J* = 7.6 Hz, 1H), 6.81 (s, 1H), 6.04 (s, 1H), 5.77 (d, *J* = 9.0 Hz, 1H), 3.42 (dd, *J* = 17.7, 2.3 Hz, 1H), 2.99 (dd, *J* = 17.8, 9.8 Hz, 1H), 2.35 (s, 3H), 2.28 (s, 3H), 2.20 (s, 3H). ^{13}C NMR (100 MHz, $CDCl_3$) δ 205.26, 140.76, 139.07, 138.58, 135.92, 129.99, 129.44, 128.76, 128.49, 124.65, 124.60, 124.04, 123.12, 70.09, 62.30, 53.08, 30.65, 21.58, 21.32.HRMS (APCI-TOF) m/z calcd. for $C_{20}H_{20}F_3NO_3SNa$ [M+Na]$^+$ 434.1014; found 434.1020. IR (neat): v_{max}/cm^{-1} 2956, 2923 2868, 1716, 1608, 1496, 1392, 1226, 1057, 882. HPLC analysis (Chiralpak IC-3; *n*-Hexane/*i*-PrOH = 95/5, 0.9 ml/min, 254 nm): t_r (major) = 5.96 min, t_r(minor) = 6.63 min, 87:13 er. $[\alpha]_D^{30}$ = +20.0 (c = 0.10 in $CHCl_3$).

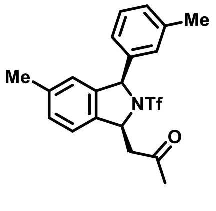

Compound 3h Following the general procedure, Compound **3h** was obtained as a colorless liquid (82 mg, 0.15 mmol, 75 %) ^{1}H NMR (500 MHz, $CDCl_3$) δ 7.49 – 7.39 (m, 2H), 7.25 – 7.17 (m, 3H), 7.07 (s, 1H), 6.89 (s, 1H), 6.15 (s, 1H), 5.80 (d, *J* = 9.6 Hz, 1H), 3.42 (d, *J* = 18.5 Hz, 1H), 2.97 (dd, *J* = 18.1, 10.0 Hz, 1H), 2.21 (s, 3H). ^{13}C NMR (125 MHz, $CDCl_3$) δ 204.85, 149.93, 149.68, 141.99, 141.96, 139.50, 137.49, 137.44, 132.36, 130.64, 126.19, 125.46, 122.40, 121.52, 120.26, 119.30, 117.37, 116.34, 68.87, 62.06, 52.67. HRMS (APCI-TOF) m/z calcd. for $C_{20}H_{15}F_9NO_5S$ [M+H]$^+$ 552.0527; found 552.0526. IR (neat): v_{max}/cm^{-1} 2955, 2924, 2869, 2852, 1737, 1600, 1491, 1461, 1377, 1191, 832. HPLC analysis (Chiralpak OD-H; *n*-Hexane/*i*-PrOH = 99/1, 0.5 ml/min, 254 nm): t_r (major) = 8.86 min, t_r(minor) = 9.28 min, 95:5 er. $[\alpha]_D^{30}$ = +62.50 (c = 0.016 in $CHCl_3$).

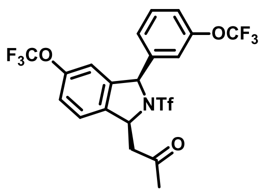

Compound 3i Following the general procedure, Compound **3i** was obtained as a colorless liquid (84 mg, 0.17 mmol, 85 %) ^{1}H NMR (500 MHz, $CDCl_3$) δ 7.38 – 7.34 (m, 2H), 7.34 – 7.30 (m, 2H), 7.19 (d, *J* = 8.4 Hz, 2H), 6.98 (d, *J* = 8.3 Hz, 1H), 6.10 (s, 1H), 5.79 (d, *J* = 8.8 Hz, 1H), 3.41 (d, *J* = 17.7 Hz, 1H), 3.01 (dd, *J* = 17.6, 9.5 Hz, 1H), 2.21 (s, 3H), 1.30 (s, 9H), 1.29 (s, 9H). ^{13}C NMR (126 MHz, $CDCl_3$) δ 205.36, 152.52, 151.52, 138.74, 135.59, 127.29, 126.31, 125.74, 123.19, 119.85, 69.69, 62.51, 53.20, 34.97, 34.67, 31.44, 31.36, 30.75. HRMS (APCI-TOF) m/z calcd. for $C_{26}H_{33}F_3NO_3S$ [M+H]$^+$ 496.2133; found 496.2129. IR (neat): v_{max}/cm^{-1} 2964, 2906, 2870, 1719, 1615, 1498, 1406, 1193, 1054, 869, 616. HPLC analysis (Chiralpak IC; *n*-Hexane/*i*-PrOH = 95/5, 0.9 ml/min, 254 nm): t_r (major) = 4.27 min, t_r(minor) = 6.37 min, 95:5 er. $[\alpha]_D^{30}$ = +13.33(c = 0.075 in $CHCl_3$).

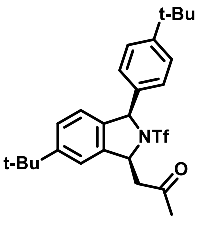

Compound 3j Following the general procedure, Compound **3j** was obtained as a colorless liquid (86.6 mg, 0.16 mmol, 81 %) ^{1}H NMR (500 MHz, $CDCl_3$) δ 7.65 – 7.60 (m, 4H), 7.60 – 7.56 (m, 4H), 7.46 (t, *J* = 7.6 Hz, 4H), 7.42 – 7.35 (m, 4H), 7.16 (t, *J* = 11.2 Hz, 1H), 6.26 (s, 1H), 5.94 (d, *J* = 9.0 Hz, 1H), 3.52 (d, *J* = 18.6 Hz, 1H), 3.14 (dd, *J* = 18.0, 9.7 Hz, 1H), 2.24 (s, 3H). ^{13}C NMR (126 MHz, $CDCl_3$) δ 205.23, 142.61, 141.74, 140.43, 140.17, 139.75, 139.61, 137.28, 129.04, 128.95, 128.24, 127.96, 127.71, 127.32, 127.22, 124.14, 122.09, 69.66, 62.44, 53.12, 30.61. HRMS (ESI-MS) m/z calcd. for $C_{30}H_{24}F_3NNaO_3S$ [M+Na]$^+$ 558.1327; found 558.1312. IR (neat): v_{max}/cm^{-1} 3441, 3032, 2924, 2857, 1716, 1642, 1483, 1391, 1155, 1057, 762. HPLC analysis (Chiralpak IC-3; *n*-Hexane/*i*-PrOH = 98/2, 0.9 ml/min, 254 nm): t_r (major) = 10.97 min, t_r(minor) = 17.52 min, 95:5 er. $[\alpha]_D^{30}$= +9.20 (c = 0.108 in $CHCl_3$).

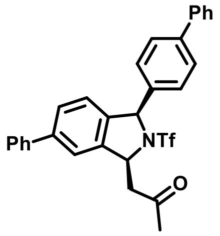

Compound 3k Following the general procedure, Compound **3k** was obtained as a pale yellow liquid (80 mg, 0.16 mmol, 83 %) ^{1}H NMR (400 MHz, $CDCl_3$) δ 7.91 (s, 1H), 7.90 – 7.78 (m, 5H), 7.73 (d, *J* = 7.9 Hz, 1H), 7.56 (s, 1H), 7.55 – 7.42 (m, 4H), 7.38 (d, *J* = 8.5 Hz, 1H), 6.49 (s, 1H), 6.04 (d, *J* = 9.2 Hz, 1H), 3.50 (dd, *J* = 18.0, 2.6 Hz, 1H), 3.16 (dd, *J* = 18.0, 9.9 Hz, 1H), 2.23 (s, 3H). ^{13}C NMR (100 MHz, $CDCl_3$) δ 205.31, 138.12, 137.56, 136.84, 133.71, 133.50, 133.32, 133.15, 129.12, 128.42, 128.36, 128.06, 127.84, 127.50, 126.82, 126.75, 125.01, 123.13, 122.77, 69.66, 61.97, 53.19, 30.64. HRMS (APCI-TOF) m/z calcd. for $C_{26}H_{21}F_3NO_3S$ [M+H]$^+$ 484.1194; found 484.1189. IR (neat): v_{max}/cm^{-1} 2955, 2924, 2869, 2853, 1716, 1602, 1493, 1461, 1392, 1222 1159,967, 859. HPLC analysis (Chiralpak IC-3; *n*-Hexane/*i*-PrOH = 98/2, 0.5 ml/min, 254 nm): t_r (major) = 21.21 min, t_r(minor) = 24.57 min, 92:8 er. $[\alpha]_D^{30}$ = +12.04 (c = 0.083 in $CHCl_3$).

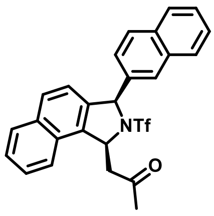

Compound 3l Following the general procedure, Compound **3l** was obtained as a pale yellow liquid (70 mg, 0.15 mmol, 78 %) ^{1}H NMR (400 MHz, $CDCl_3$) δ 7.16 (q, *J* = 7.4 Hz, 2H), 7.00 (dd, *J* = 7.8, 1.4 Hz, 1H), 6.91 (d, *J* = 10.4 Hz, 1H), 6.70 (d, *J* = 7.7 Hz, 1H), 6.07 (s, 1H), 5.95 (t, *J* = 5.0 Hz, 1H), 3.22 (dd, *J* = 16.8, 4.3 Hz,1H), 3.11 (dd, *J* = 16.8, 5.9 Hz,1H), 2.27 (d, *J* = 1.8 Hz,1H), 2.25 (d, *J* = 1.6 Hz,1H), 2.21 (s, 3H). ^{13}C NMR (100 MHz, $CDCl_3$) δ 203.00, 162.58, 160.11, 156.70, 154.21, 144.34, 139.82, 139.75, 138.26, 132.94, 132.90, 131.87, 131.82, 125.85, 125.68, 125.50, 125.15, 123.49, 123.46, 123.15, 119.06, 119.02, 114.66, 114.42, 110.28, 110.03, 69.73, 60.73, 50.76, 30.42, 14.44, 14.21.HRMS (ESI-MS) m/z calcd.for $C_{20}H_{17}NO_3F_5S$ [M-H]$^-$ 446.0855; found 446.0875. IR (neat): v_{max}/cm^{-1} 2956, 2923, 2851, 1719, 1493, 1394, 1226, 1191, 1066, 646. HPLC analysis (Chiralpak IC-3; *n*-Hexane/*i*-PrOH = 97/3, 0.5 ml/min, 254 nm): t_r (major) = 9.78 min, t_r(minor) = 11.42 min, 90:10 er. $[\alpha]_D^{30}$ = -15.10 (c = 0.066 in $CHCl_3$).

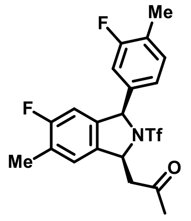

Compound 3m Following the general procedure, Compound **3m** was obtained as a colorless liquid (78 mg, 0.16 mmol, 83%) ^{1}H NMR (500 MHz, $CDCl_3$) δ 7.02 (dd, *J* = 8.3, 2.1 Hz, 1H), 6.98 (s, 1H), 6.77 (dd, *J* = 14.4, 6.0 Hz, 3H), 5.98 (s, 1H), 5.70 (brs, 1H), 3.81 (s, 3H), 3.81 (s, 3H), 3.40 (d, *J* = 18.0 Hz, 1H), 2.99 (dd, *J* = 18.0, 9.8 Hz, 1H), 2.20 (s, 3H), 2.18 (s, 3H), 2.12 (s, 3H). ^{13}C NMR (100 MHz, $CDCl_3$) δ 205.81, 158.55, 157.95, 137.44, 130.17, 129.86, 128.27, 127.05, 126.63, 125.20, 109.74, 104.19, 69.62, 62.32, 55.57, 55.39, 53.39, 30.65, 16.56, 16.51.HRMS (ESI-MS) m/z calcd. for $C_{22}H_{24}F_3NO_5SNa$ [M+Na]$^+$ 494.1225; found 494.1237. IR (neat): v_{max}/cm^{-1} 3423, 2955, 2922, 2850, 1717, 1610,1503, 1465, 1390, 1205, 1187, 1034, 607. HPLC analysis (Chiralpak IC-3; *n*-Hexane/*i*-PrOH = 98/2, 0.5 ml/min, 254 nm): t_r (major) = 13.84 min, t_r(minor) = 20.33 min, 93:7 er. $[\alpha]_D^{30}$ = +30.30 (c = 0.033 in $CHCl_3$).

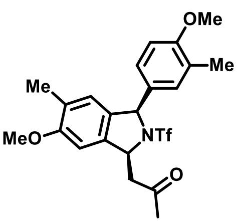

Compound 3n Following the general procedure, Compound **3n** was obtained as a colorless liquid (80 mg, 0.16 mmol, 80 %) ^{1}H NMR (400 MHz, $CDCl_3$) δ 6.91 – 6.82 (m, 2H), 6.80 (d, *J* = 8.4 Hz, 1H), 6.67 (dd, *J* = 8.3, 1.4 Hz, 1H), 6.46 (s, 1H), 6.02 (s, 1H), 5.65 (d, *J* = 8.9 Hz, 1H), 3.86 (s, 6H), 3.84 (s, 3H), 3.76 (s, 3H), 3.33 (dd, *J* = 17.9, 2.7 Hz, 1H), 2.92 (dd, *J* = 17.9, 9.7 Hz, 1H), 2.17 (s, 3H). ^{13}C NMR (126 MHz, $CDCl_3$) δ 205.72, 150.20, 149.50, 149.20, 133.17, 129.88, 120.52, 111.86, 111.11, 105.87, 105.82, 69.93, 62.44, 56.22, 56.14, 56.02, 53.53, 53.15, 30.66. HRMS (APCI-TOF) m/z calcd. for $C_{22}H_{24}F_3NO_7SNa$ $[M+Na]^+$ 526.1123; found 526.1119. IR (neat): v_{max}/cm^{-1} 2956, 2923, 2851, 1715, 1465, 1389, 1224, 1190, 1026, 611. HPLC analysis (Chiralpak ID; *n*-Hexane/*i*-PrOH = 95/5, 0.9 ml/min, 254 nm): t_r (major) = 30.66 min, t_r(minor) = 32.99 min, 87:13 er. $[\alpha]_D^{30}$ = +15.10 (c = 0.066 in $CHCl_3$).

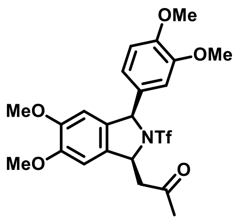

Compound 4b Following the general procedure, Compound **4b** was obtained as a colorless liquid (67.5 mg, 0.17 mmol, 85 %) ^{1}H NMR (500 MHz, $CDCl_3$) δ 7.39 – 7.27 (m, 8H), 7.05 (d, *J* = 7.1 Hz, 1H), 6.17 (s, 1H), 5.87 (d, *J* = 8.7 Hz, 1H), 3.39 (dd, *J* = 17.5, 2.7 Hz, 1H), 3.00 (dd, *J* = 17.5, 9.6 Hz, 1H), 2.52 (dq, *J* = 17.7, 7.3 Hz, 1H), 2.40 (dq, *J* = 17.7, 7.3 Hz, 1H), 1.10 (t, *J* = 7.3 Hz, 3H). ^{13}C NMR (125 MHz, $CDCl_3$) δ 207.90, 140.68, 138.92, 138.31, 129.06, 128.97, 128.88, 128.70, 127.75, 123.74, 123.37, 70.05, 62.60, 51.64, 36.63, 7.59. HRMS (ESI-MS) m/z calcd. for $C_{19}H_{17}NO_3F_3S$ $[M-H]^-$396.0887; found 396.0912. IR (neat): v_{max}/cm^{-1} 3036, 2980, 2941, 2856, 1715, 1602, 1496, 1460, 1392, 1153, 1051, 912. HPLC analysis (Chiralpak IC-3; *n*-Hexane/*i*-PrOH = 98/2, 0.9 ml/min, 254 nm): t_r (major) = 6.76 min, t_r(minor) = 7.42 min, 93:7 er. $[\alpha]_D^{30}$ = +4.0 (c = 0.25 in $CHCl_3$).

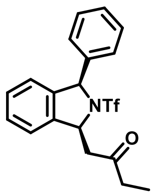

Compound 4c Following the general procedure, Compound **4c** was obtained as a colorless liquid (69 mg, 0.16 mmol, 84 %) ^{1}H NMR (400 MHz, $CDCl_3$) δ 7.41 – 7.27 (m, 6H), 7.27 – 7.24 (m, 2H), 7.03 (d, *J* = 6.9 Hz, 1H), 6.14 (s, 1H), 5.85 (d, *J* = 8.9 Hz, 1H), 3.37 (dd, *J* = 17.7, 2.6 Hz, 1H), 2.97 (dd, *J* = 17.7, 9.8 Hz, 1H), 2.46 (dt, *J* = 16.4, 7.4 Hz, 1H), 2.41 – 2.30 (m, 1H), 1.69 – 1.57 (m, 2H), 1.54 (s, 3H), 0.92 (t, *J* = 7.4 Hz, 3H). ^{13}C NMR (126 MHz, $CDCl_3$) δ 207.56, 140.73, 139.01, 138.35, 129.08, 128.97, 128.91, 128.72, 127.77, 123.75, 123.44, 70.05, 62.53, 52.06, 45.34, 17.19, 13.73. HRMS (APCI-TOF) m/z calcd. for $C_{20}H_{21}F_3NO_3S$ $[M+H]^+$ 412.1194; found 412.1197. IR (neat): v_{max}/cm^{-1} 2961 2925, 2855, 1714, 1575, 1483, 1460, 1392, 1186, 1054, 897. HPLC analysis (Chiralpak IC-3; *n*-Hexane/*i*-PrOH = 98/2, 0.9 ml/min, 254 nm): t_r (major) = 6.08 min, t_r(minor) = 6.37 min, 98:2 er. $[\alpha]_D^{30}$ = +16.60 (c = 0.183 in $CHCl_3$).

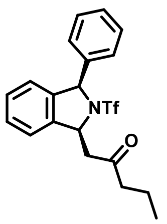

Compound 4d Following the general procedure, Compound **4d** was obtained as a yellow liquid (66.7mg, 0.15 mmol, 75 %) ^{1}H NMR (500 MHz, $CDCl_3$) δ 7.99 – 7.89 (m, 2H), 7.61 – 7.54 (m, 1H), 7.47 (t, *J* = 7.8 Hz, 2H), 7.44 – 7.35 (m, 4H), 7.31 (ddd, *J* = 7.1, 4.2, 1.9 Hz, 4H), 7.06 (d, *J* = 4.4 Hz, 1H), 6.21 (s, 1H), 6.06 (d, *J* = 8. 8 Hz, 1H), 3.94 (dd, *J* = 17.3, 2.6 Hz, 1H), 3.53 (dd, *J* = 17.3, 10.0 Hz, 1H). ^{13}C NMR (125 MHz, $CDCl_3$) δ 196.78, 140.72, 138.95, 138.37, 136.40, 133.82, 129.07, 128.97, 128.91, 128.82, 128.27, 127.99, 127.32, 123.83, 123.79, 70.07, 63.16, 48.37. HRMS (APCI-TOF) m/z calcd. for $C_{23}H_{19}F_3NO_3S$ $[M+H]^+$ 446.1038; found 446.1078. IR (neat): v_{max}/cm^{-1} 3437, 2955, 2924, 2855, 1682, 1636, 1460, 1392, 1225, 1118, 1060, 733. HPLC analysis (Chiralpak AD-H; *n*-Hexane/*i*-PrOH = 99/1, 0.5 ml/min, 254 nm): t_r (major) = 15.40 min, t_r(minor) = 13.55 min, 90:10 er. $[\alpha]_D^{30}$ = +30.00 (c = 0.033 in $CHCl_3$).

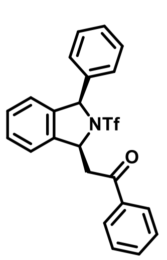

Compound 4e Following the general procedure, Compound **4e** was obtained as a pale yellow liquid (43.9 mg, 0.12 mmol, 60 %) ^{1}H NMR (500 MHz, $CDCl_3$) δ 7.55 (s, 1H), 7.47 (t, *J* = 7.5 Hz, 1H), 7.41 (t, *J* = 7.6 Hz, 1H), 7.35 (t, *J* = 6.2 Hz, 5H), 7.05 (d, *J* = 4.8 Hz, 1H), 6.21 (s, 1H), 5.52 (s, 1H), 3.24 (d, *J* = 14.3 Hz, 1H), 3.02 (s, 1H). ^{13}C NMR (100 MHz, $CDCl_3$) δ 138.86, 135.07, 130.17, 129.49, 129.26, 128.94, 128.78, 124.34, 122.77, 116.03, 70.73, 62.36, 26.39. ^{13}C NMR (100 MHz, $CDCl_3$) δ 139.25, 138.89, 135.07, 130.17, 129.51, 129.26, 128.95, 124.34, 122.79, 116.25, 70.83, 62.48, 26.14. HRMS (APCI-TOF) m/z calcd. for $C_{17}H_{14}F_3N_2O_2S$ $[M+H]^+$ 367.0728; found 367.0735. IR (neat): v_{max}/cm^{-1} 3037, 2955, 2923, 2851, 2253, 1960, 1461, 1392, 1226, 1194, 1056, 612. HPLC analysis (Chiralpak IC-3; *n*-Hexane/*i*-PrOH = 90/10, 0.9 ml/min, 254 nm): t_r (major) = 14.74 min, t_r(minor) = 6.86 min, 90:10 er. $[\alpha]_D^{30}$ = +7.51 (c = 0.133 in $CHCl_3$).

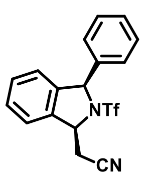

Compound 4f Following the general procedure, Compound **4f** was obtained as a pale yellow liquid (52.2 mg, 0.14 mmol, 65 %) ^{1}H NMR (400 MHz, $CDCl_3$) δ 7.34 (s, 1H), 7.22 (t, *J* = 7.2 Hz, 3H), 7.16 (d, *J* = 7.9 Hz, 2H), 6.93 (d, *J* = 7.2 Hz, 1H), 6.15 (s, 1H), 5.45 (s, 1H), 3.21 (dd, *J* = 16.8, 2.5 Hz, 1H), 2.99 (dd, *J* = 16.6, 7.3 Hz, 1H), 2.43 (s, 3H), 2.34 (s, 3H). ^{13}C NMR (100 MHz, $CDCl_3$) δ 139.66, 139.12, 136.56, 136.13, 135.32, 131.11, 129.56, 128.65, 124.02, 123.06, 116.23, 70.46, 62.27, 26.34, 21.51, 21.26. HRMS (ESI-MS) m/z calcd. for $C_{19}H_{17}F_3N_2O_2SNa$ $[M+Na]^+$ 417.0861; found 417.0851. IR (neat): v_{max}/cm^{-1} 3030, 2925, 2854, 2253, 1914, 1515, 1458, 1392, 1225, 1194, 1044, 823, 643. HPLC analysis (Chiralpak AD-H; *n*-Hexane/*i*-PrOH = 95/5, 0.9 ml/min, 254 nm): t_r (major) = 6.44 min, t_r(minor) = 7.46 min, 91:9 er. $[\alpha]_D^{30}$ = +3.0 (c = 0.33 in $CHCl_3$).

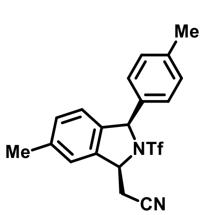

Compound 4g Following the general procedure, Compound **4g** was obtained as colorless liquid (47.9 mg, 0.11 mmol, 56 %) ^{1}H NMR (400 MHz, $CDCl_3$) δ 7.47 (s, 1H), 7.40 (dd, J = 8.3, 1.6 Hz, 1H), 7.37 – 7.25 (m, 4H), 6.95 (d, J = 7.9 Hz, 1H), 6.14 (s, 1H), 5.48 (s, 1H), 3.18 (dd, J = 17.1, 3.2 Hz, 1H), 3.08 (dd, J = 17.1, 6.4 Hz, 1H).^{13}C NMR (125 MHz, $CDCl_3$) δ 136.94, 136.65, 135.96, 135.68, 130.85, 130.39, 129.27, 125.55, 122.99, 115.68, 69.70, 62.13, 25.88. HRMS (ESI-MS) m/z calcd. for $C_{19}H_{17}F_3N_2O_2SNa$ $[M+Na]^+$ 456.9768; found 456.9777. IR (neat): v_{max}/cm^{-1} 3037, 2926, 2855, 2253, 1915, 1493, 1393, 1226, 1212. 1149, 1088, 626. HPLC analysis (Chiralpak AD-H; *n*-Hexane/*i*-PrOH = 95/5, 0.5 ml/min, 254 nm): t_r (major) = 22.60 min, t_r(minor) = 20.89 min, 94:6 er. $[\alpha]_D^{30}$ = +10.0 (c = 0.10 in $CHCl_3$).

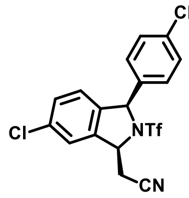

Compound 4h Following the general procedure, Compound **4h** was obtained as colorless liquid (57.8 mg, 0.12 mmol, 62 %) ^{1}H NMR (400 MHz, $CDCl_3$) δ 8.06 (s, 3H), 7.95 (d, J = 8.1 Hz, 3H), 7.85 (dd, J = 19.1, 11.7 Hz, 11H), 7.73 (d, J = 7.9 Hz, 3H), 7.61 – 7.44 (m, 13H), 7.42 (d, J = 8.6 Hz, 3H), 6.52 (s, 3H), 5.70 (s, 3H), 3.34 (dd, J = 16.9, 3.5 Hz, 3H), 3.14 (s, 3H). ^{13}C NMR (100 MHz, $CDCl_3$) δ 134.01, 133.58, 133.53, 133.03, 129.20, 128.48, 128.41, 128.18, 127.84, 127.39, 127.23, 127.02, 126.75, 125.43, 123.77, 122.34, 116.15, 70.41, 61.91, 26.69. HRMS (ESI-MS) m/z calcd. for $C_{25}H_{17}F_3N_2O_2SNa$ $[M+Na]^+$ 489.0861; found 489.0843. IR (neat): v_{max}/cm^{-1} 3058, 2956, 2925, 2852, 2254, 1957, 1508, 1390, 1221, 1152, 1055, 649. HPLC analysis (Chiralpak AD-H; *n*-Hexane/*i*-PrOH = 98/2, 0.5 ml/min, 254 nm): t_r (major) = 58.05 min, t_r(minor) = 52.65 min, 94:6 er. $[\alpha]_D^{30}$ = +40.0 (c = 0.05 in $CHCl_3$).

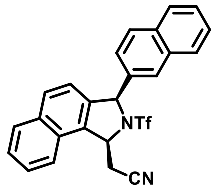

Compound 4i Following the general procedure, Compound **4i** was obtained as a colorless liquid (62.5 mg, 0.13 mmol, 68 %) ^{1}H NMR (500 MHz, $CDCl_3$) δ 7.49 (t, J = 8.1 Hz, 3H), 7.36 – 7.26 (m, 6H), 7.12 (t, J = 7.5 Hz, 1H), 7.06 (d, J = 7.2 Hz, 1H), 6.19 (s, 1H), 5.84 (d, J = 6.6 Hz, 1H), 3.30 (dd, J = 14.7, 3.7 Hz, 1H), 2.85 (dd, J = 14.8, 9.0 Hz, 1H). ^{13}C NMR (125 MHz, $CDCl_3$) δ 166.90, 140.49, 138.14, 137.43, 129.16, 128.90, 128.80, 127.93, 124.80, 123.85, 123.59, 120.10, 70.25, 63.93, 46.38. HRMS (APCI-TOF) m/z calcd. for $C_{23}H_{20}F_3N_2O_3S$ $[M+H]^+$ 461.1147; found 461.1157. IR (neat): v_{max}/cm^{-1} 2955, 2955, 2921, 2850, 1660, 1600, 1543, 1497, 1393, 1225, 1189, 1055, 696. HPLC analysis (Chiralpak IC-3; *n*-Hexane/*i*-PrOH = 90/10, 0.9 ml/min, 254 nm): t_r (major) = 9.62 min, t_r(minor) = 8.93 min, 90:10 er. $[\alpha]_D^{30}$ = +86.90 (c = 0.023 in $CHCl_3$).

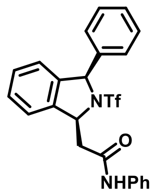

Compound 4j Following the general procedure, Compound **4j** was obtained as a colorless liquid (80 mg, 0.14 mmol, 70 %) ^{1}H NMR (500 MHz, $CDCl_3$) δ 7.48 (t, J = 9.0 Hz, 2H), 7.45 (s, 1H), 7.38 – 7.27 (m, 5H), 7.21 (d, J = 8.3 Hz, 2H), 7.16 (brs, 1H), 7.12 (t, J = 7.4 Hz, 1H), 7.02 (d, J = 8.2 Hz, 1H), 6.15 (s, 1H), 5.79 (s, 1H), 3.30 (dd, J = 14.4, 4.2 Hz, 1H), 2.80 (dd, J = 14.3, 8.9 Hz, 1H), 1.29 (s, 9H), 1.24 (s, 9H). ^{13}C NMR (125 MHz, $CDCl_3$) δ 166.98, 157.49, 152.62, 151.62, 150.03, 137.99, 137.63, 137.47, 137.29, 135.48, 129.13, 127.41, 126.50, 125.76, 124.74, 123.30, 120.00, 69.82, 64.07, 47.02, 35.00, 34.67, 31.40, 31.35. HRMS (APCI-TOF) m/z calcd. for $C_{31}H_{36}F_3N_2O_3S$ $[M+H]^+$ 573.2399; found 573.2405 IR (neat): v_{max}/cm^{-1} 2959, 2925, 2868, 1660, 1601, 1544, 1498, 1378, 1225, 1188, 1059, 691. HPLC analysis (Chiralpak AD-H; *n*-Hexane/*i*-PrOH = 95/5, 0.9 ml/min, 254 nm): t_r (major) = 8.02 min, t_r(minor) = 6.28 min, 90:10 er. $[\alpha]_D^{30}$= +40.00 (c = 0.05 in $CHCl_3$).

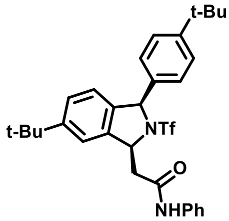

Compound 4k Following the general procedure, Compound **4k** was obtained as a yellow liquid (59.5 mg, 0.12 mmol, 60 %) ^{1}H NMR (400 MHz, $CDCl_3$) δ 7.45 (d, J = 8.1 Hz, 2H), 7.31 (dd, J = 15.3, 7.2 Hz, 2H), 7.27 – 7.18 (m, 4H), 7.13 (t, J = 7.3 Hz, 1H), 7.03 (dd, J = 9.6, 7.5 Hz, 1H), 6.94 (dd, J = 16.3, 8.0 Hz, 3H), 6.12 (s, 1H), 5.77 (d, J = 8.0 Hz, 1H), 3.27 (dd, J = 14.9, 3.0 Hz, 1H), 2.88 (dd, J = 15.1, 9.1 Hz, 1H). ^{13}C NMR (100 MHz, $CDCl_3$) δ 166.52, 164.59, 164.18, 162.09, 161.69, 140.17, 137.20, 136.08, 133.56, 130.06, 129.98, 129.19, 125.35, 125.26, 124.98, 120.11, 117.12, 116.89, 115.99, 115.77, 111.23, 110.98, 69.14, 63.53, 45.60. HRMS(ESI-MS) m/z calcd. for $C_{23}H_{17}F_3N_2O_3SNa$ $[M+Na]^+$519.0773; found 519.0778. IR (neat): v_{max}/cm^{-1} 2919, 2956, 2851, 1660, 1602, 1547, 1510, 1444, 1394, 1225, 1192, 1055, 617. HPLC analysis (Chiralpak IC-3; *n*-Hexane/*i*-PrOH = 95/5, 0.9 ml/min, 254 nm): t_r (major) = 12.13 min, t_r(minor) = 10.69, 86:14 er. $[\alpha]_D^{30}$= +12.00 (c = 0.25 in $CHCl_3$).

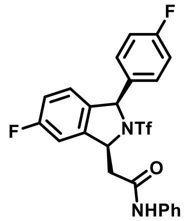

Compound 4l Following the general procedure, Compound **4l** was obtained as a colorless liquid (70.3 mg, 0.14 mmol, 72 %) ^{1}H NMR (400 MHz, $CDCl_3$) δ 7.46 (d, J = 8.0 Hz, 2H), 7.31 (dd, J = 14.8, 7.1 Hz, 3H), 7.11 (dt, J = 23.0, 8.5 Hz, 6H), 6.92 (d, J = 7.8 Hz, 1H), 6.11 (s, 1H), 5.75 (d, J = 7.1 Hz, 1H), 3.24 (dd, J = 14.7, 3.4 Hz, 1H), 2.83 (dd, J = 14.8, 8.7 Hz, 1H), 2.33 (s, 3H), 2.30 (s, 3H).^{13}C NMR (100 MHz, $CDCl_3$) δ 167.01, 139.24, 138.62, 138.37, 137.78, 137.44, 135.47, 130.13, 129.52, 129.12, 127.89, 124.74, 123.79, 123.53, 120.13, 69.91, 63.77, 46.54, 21.49, 21.23. HRMS(ESI-MS) m/z calcd. for $C_{25}H_{23}F_3N_2O_3SNa$ $[M+Na]^+$511.1279; found 511.1260 IR (neat): v_{max}/cm^{-1} 2955, 2923, 2851, 1655, 1600, 1548, 1443, 1378, 1225, 1188, 1055, 652. HPLC analysis (Chiralpak AD-H; *n*-Hexane/*i*-PrOH = 95/5, 0.9 ml/min, 254 nm): t_r (major) = 12.47 min, t_r(minor) = 10.62 min, 91:9 er. $[\alpha]_D^{30}$= +7.69 (c = 0.13 in $CHCl_3$).

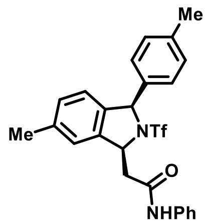

4. NMR spectra of Diarylmethyltriflamides 1

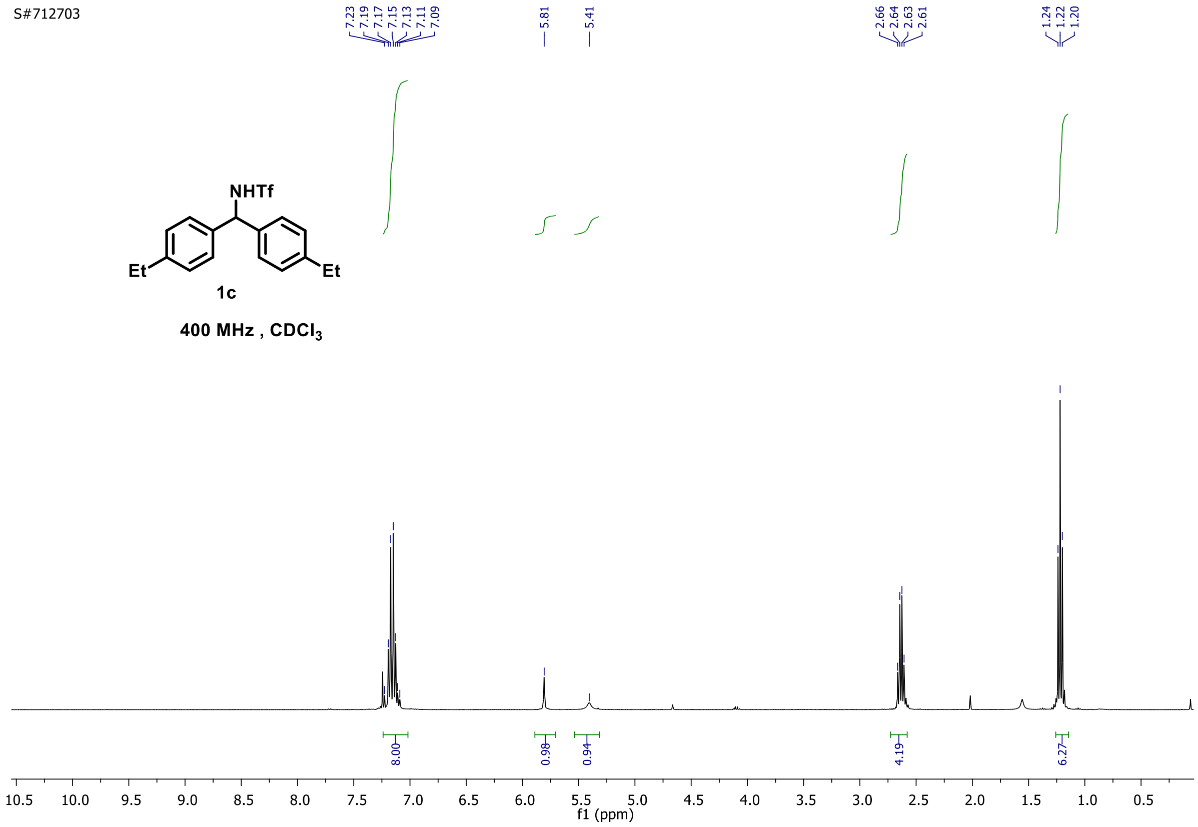

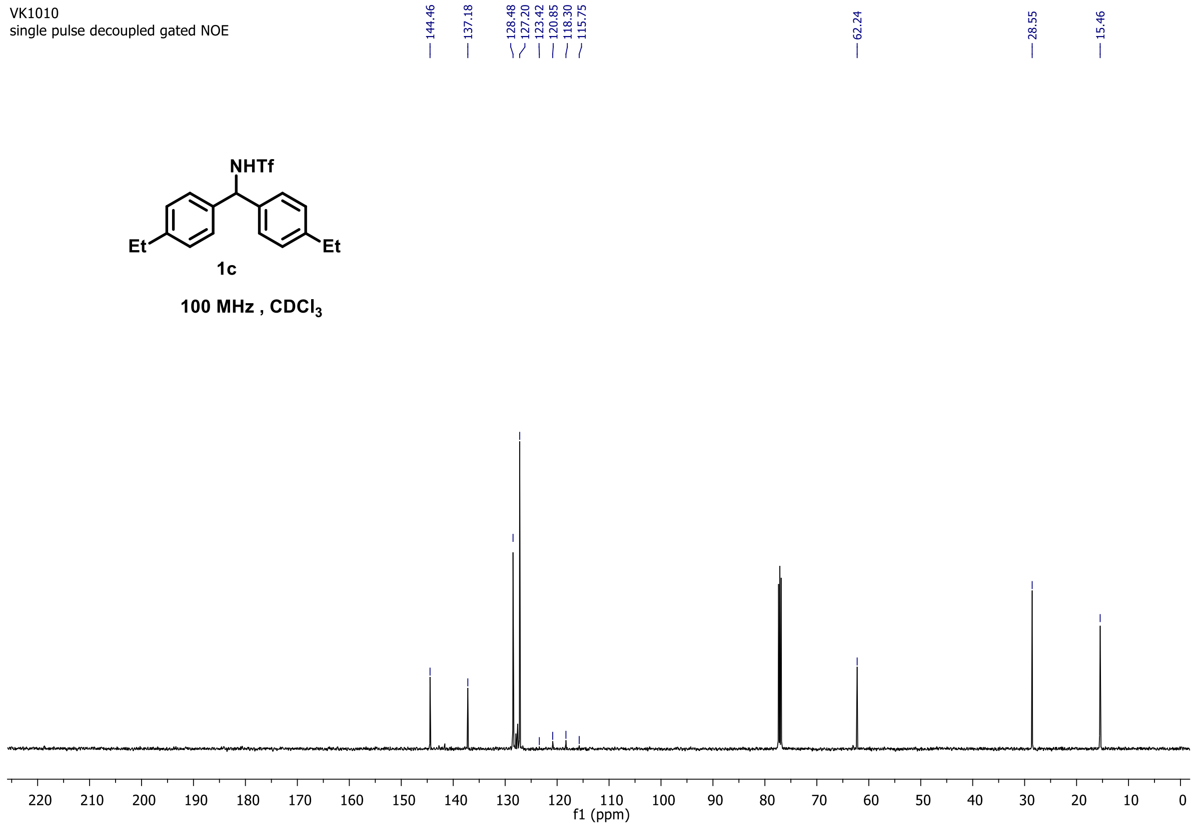

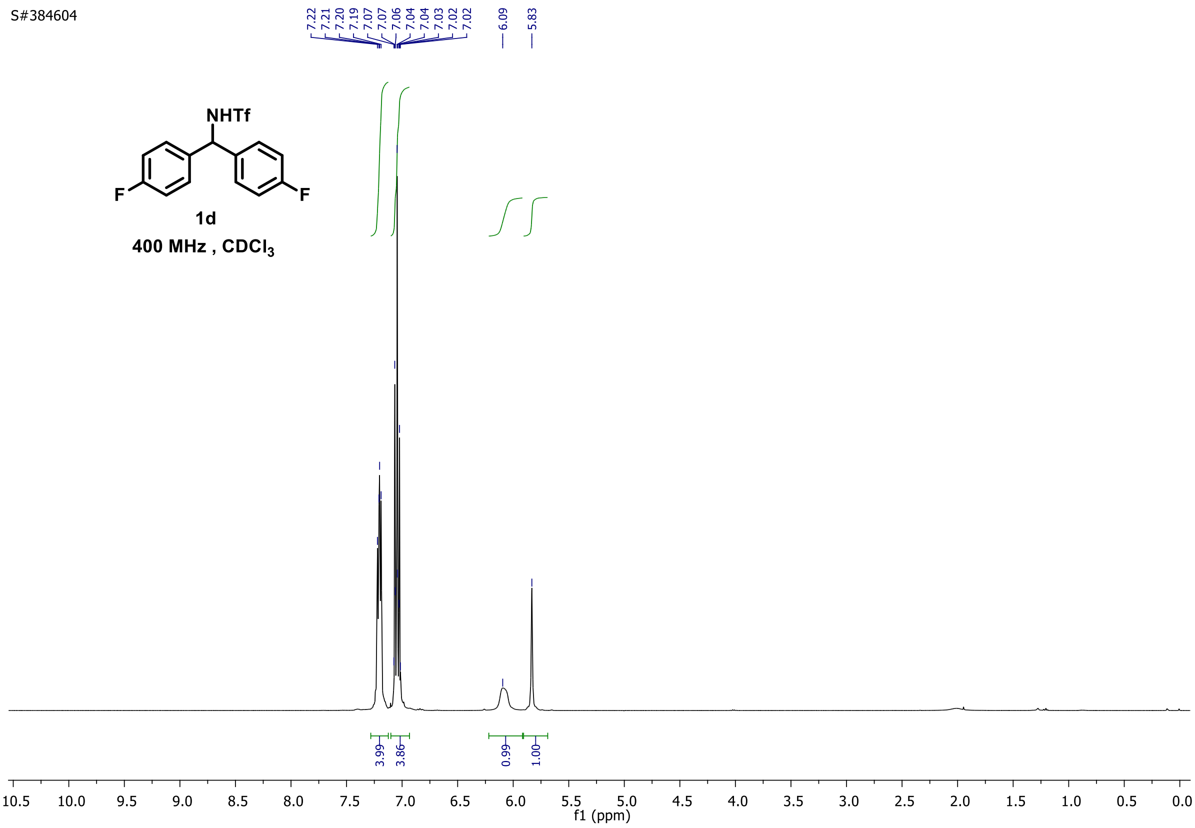
S#384604
7.22
7.21
7.20
7.19
7.07
7.07
7.06
7.04
7.04
7.03
7.02
7.02
6.09
5.83
NHTf
F
F
1d
400 MHz , CDCl3
3.99
3.86
0.99
1.00
10.5 10.0 9.5 9.0 8.5 8.0 7.5 7.0 6.5 6.0 5.5 5.0 4.5 4.0 3.5 3.0 2.5 2.0 1.5 1.0 0.5 0.0
f1 (ppm)

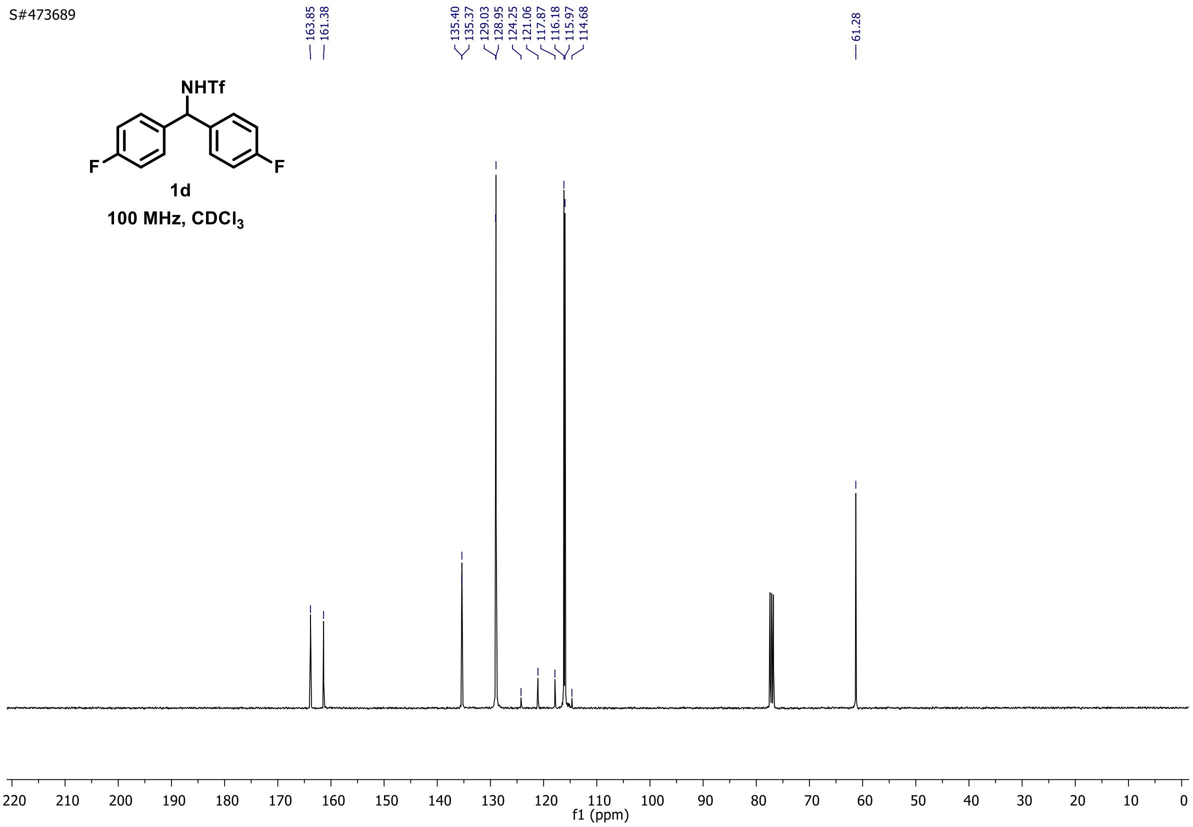
S#473689
163.85
161.38
135.40
135.37
129.03
128.95
124.25
121.06
117.87
116.18
115.97
114.68
61.28
NHTf
F
F
1d
100 MHz, CDCl3
220 210 200 190 180 170 160 150 140 130 120 110 100 90 80 70 60 50 40 30 20 10 0
f1 (ppm)

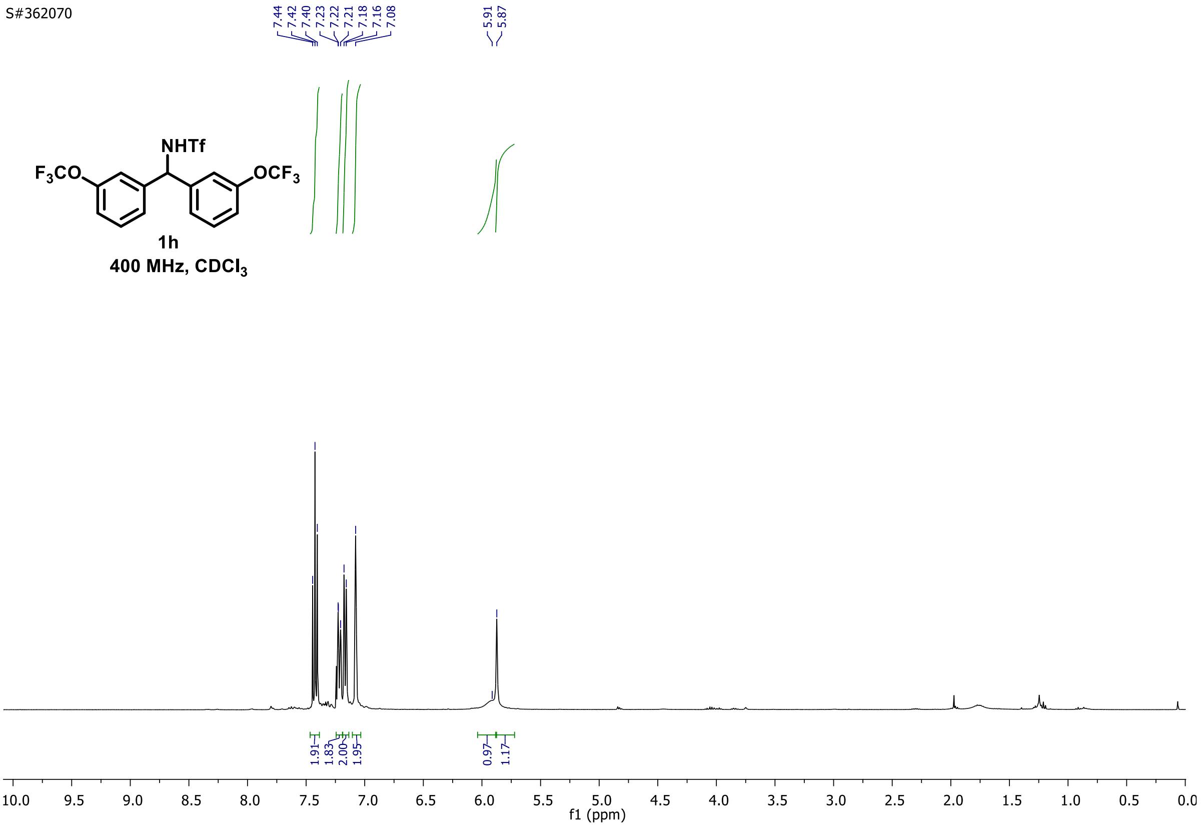
S#362070
7.44
7.42
7.40
7.23
7.22
7.21
7.18
7.16
7.08
5.91
5.87
NHTf
F3CO
OCF3
1h
400 MHz, CDCl3
1.91
1.83
2.00
1.95
0.97
1.17
10.0 9.5 9.0 8.5 8.0 7.5 7.0 6.5 6.0 5.5 5.0 4.5 4.0 3.5 3.0 2.5 2.0 1.5 1.0 0.5 0.0
f1 (ppm)

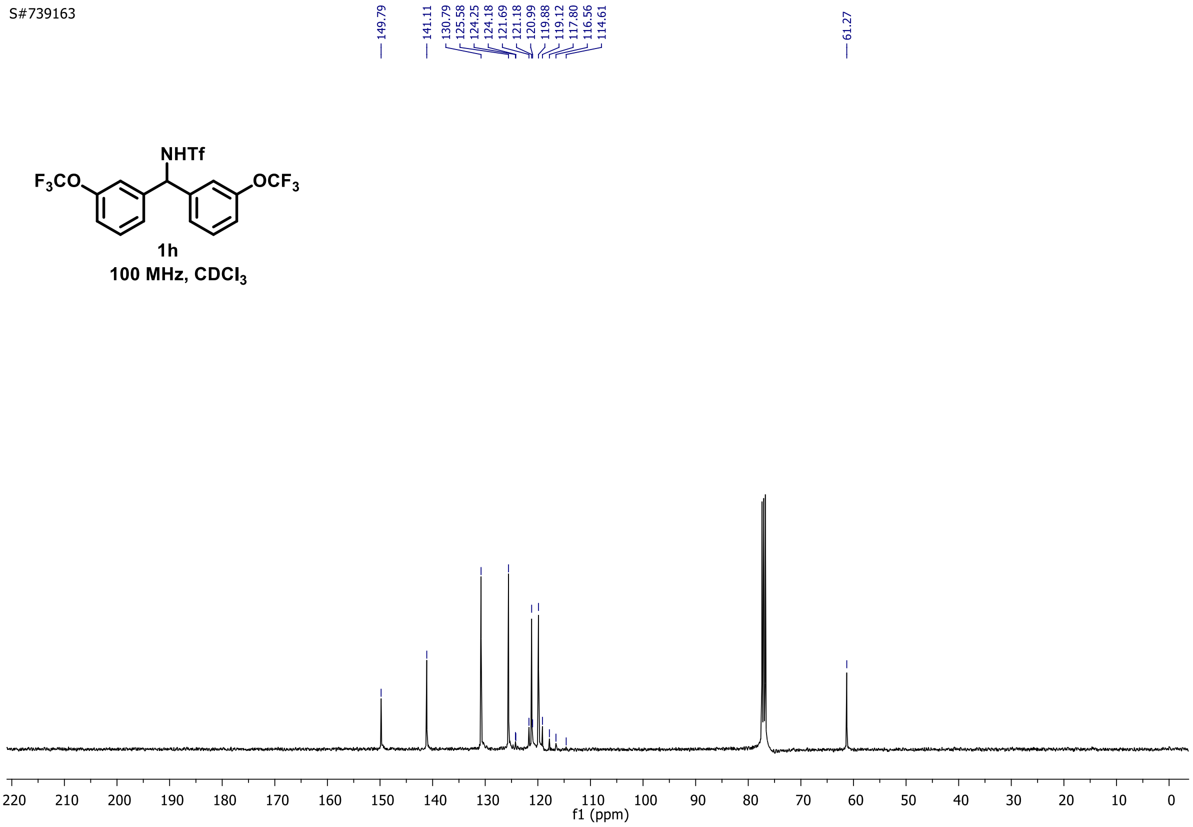
S#739163
149.79
141.11
130.79
125.58
124.25
124.18
121.69
121.18
120.99
119.88
119.12
117.80
116.56
114.61
61.27
NHTf
F3CO
OCF3
1h
100 MHz, CDCl3
220 210 200 190 180 170 160 150 140 130 120 110 100 90 80 70 60 50 40 30 20 10 0
f1 (ppm)

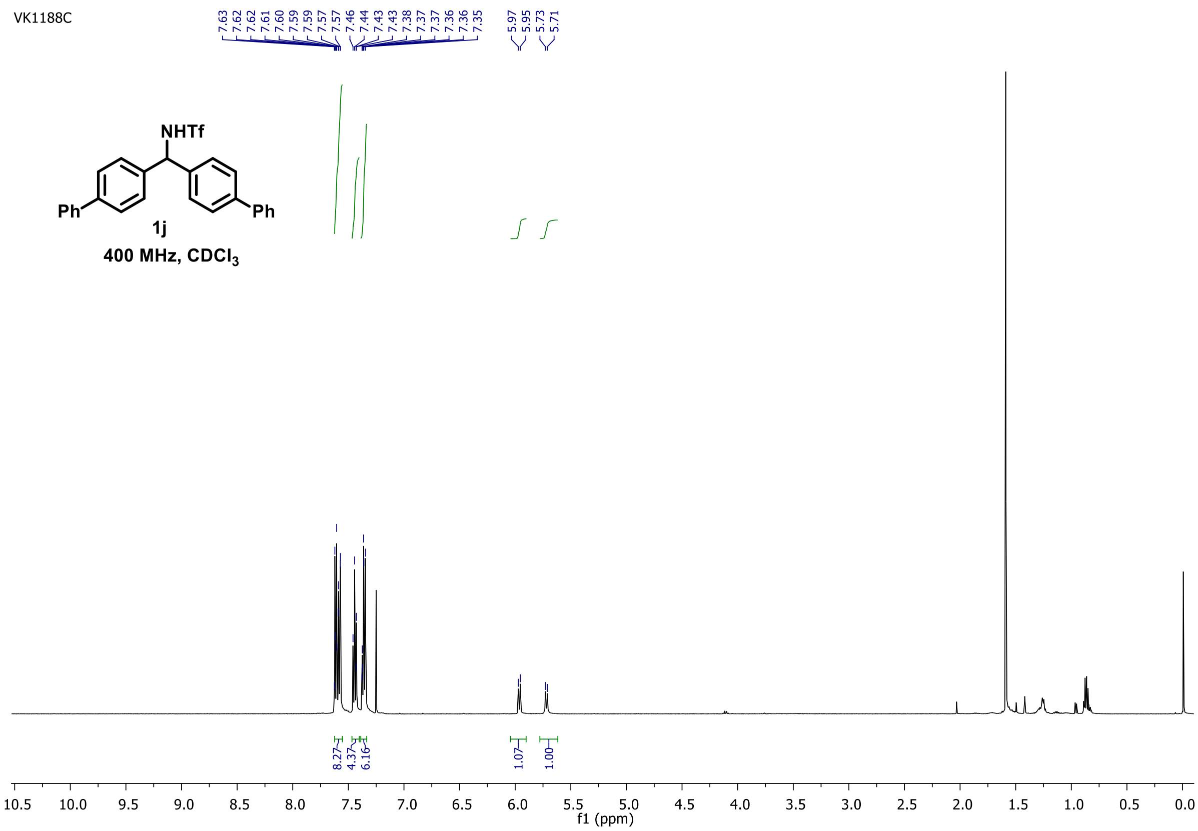
VK1188C
7.63
7.62
7.62
7.61
7.60
7.59
7.59
7.57
7.57
7.46
7.44
7.43
7.43
7.38
7.37
7.37
7.36
7.36
7.35
5.97
5.95
5.73
5.71
NHTf
Ph
Ph
1j
400 MHz, CDCl3
8.27
4.37
6.16
1.07
1.00
10.5 10.0 9.5 9.0 8.5 8.0 7.5 7.0 6.5 6.0 5.5 5.0 4.5 4.0 3.5 3.0 2.5 2.0 1.5 1.0 0.5 0.0
f1 (ppm)

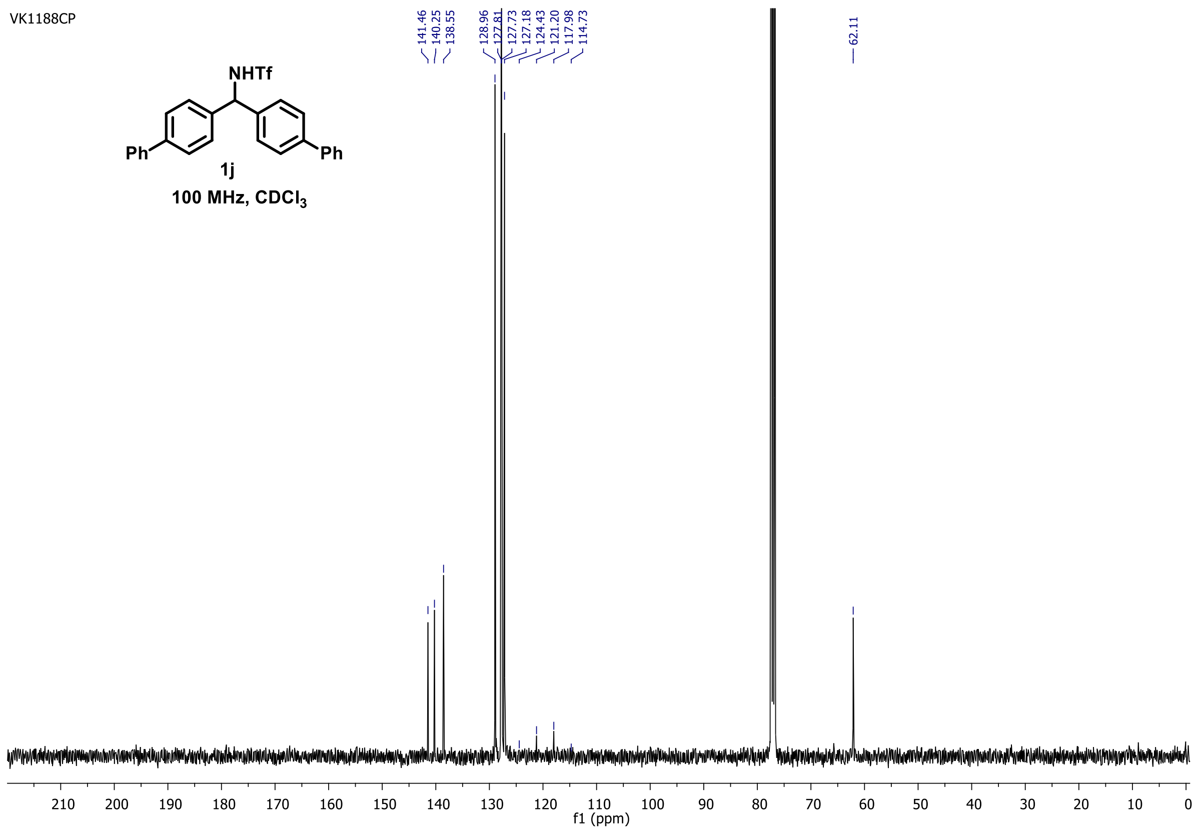
VK1188CP
141.46
140.25
138.55
128.96
127.81
127.73
127.18
124.43
121.20
117.98
114.73
62.11
NHTf
Ph
Ph
1j
100 MHz, CDCl3
210 200 190 180 170 160 150 140 130 120 110 100 90 80 70 60 50 40 30 20 10 0
f1 (ppm)

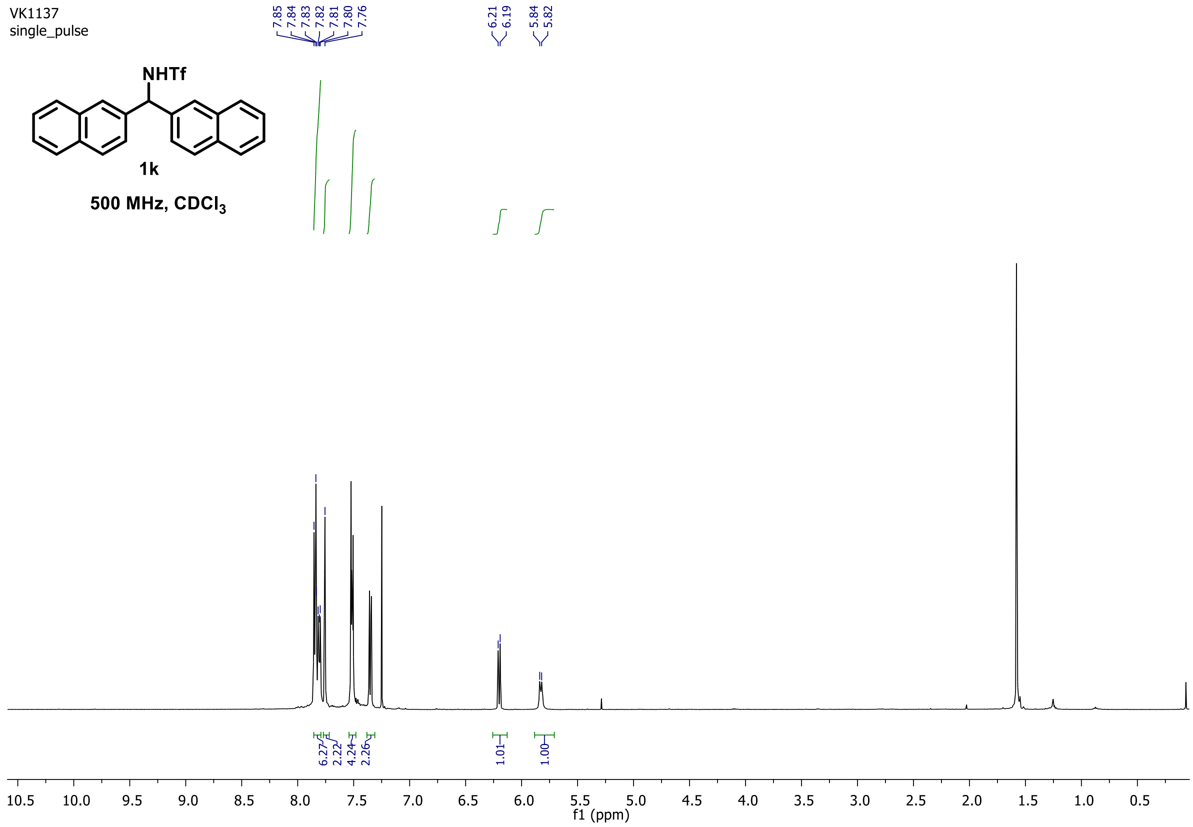
VK1137
single_pulse
NHTf
1k
500 MHz, CDCl3
7.85
7.84
7.83
7.82
7.81
7.80
7.76
6.21
6.19
5.84
5.82
6.27
2.22
4.24
2.26
1.01
1.00
10.5 10.0 9.5 9.0 8.5 8.0 7.5 7.0 6.5 6.0 5.5 5.0 4.5 4.0 3.5 3.0 2.5 2.0 1.5 1.0 0.5
f1 (ppm)

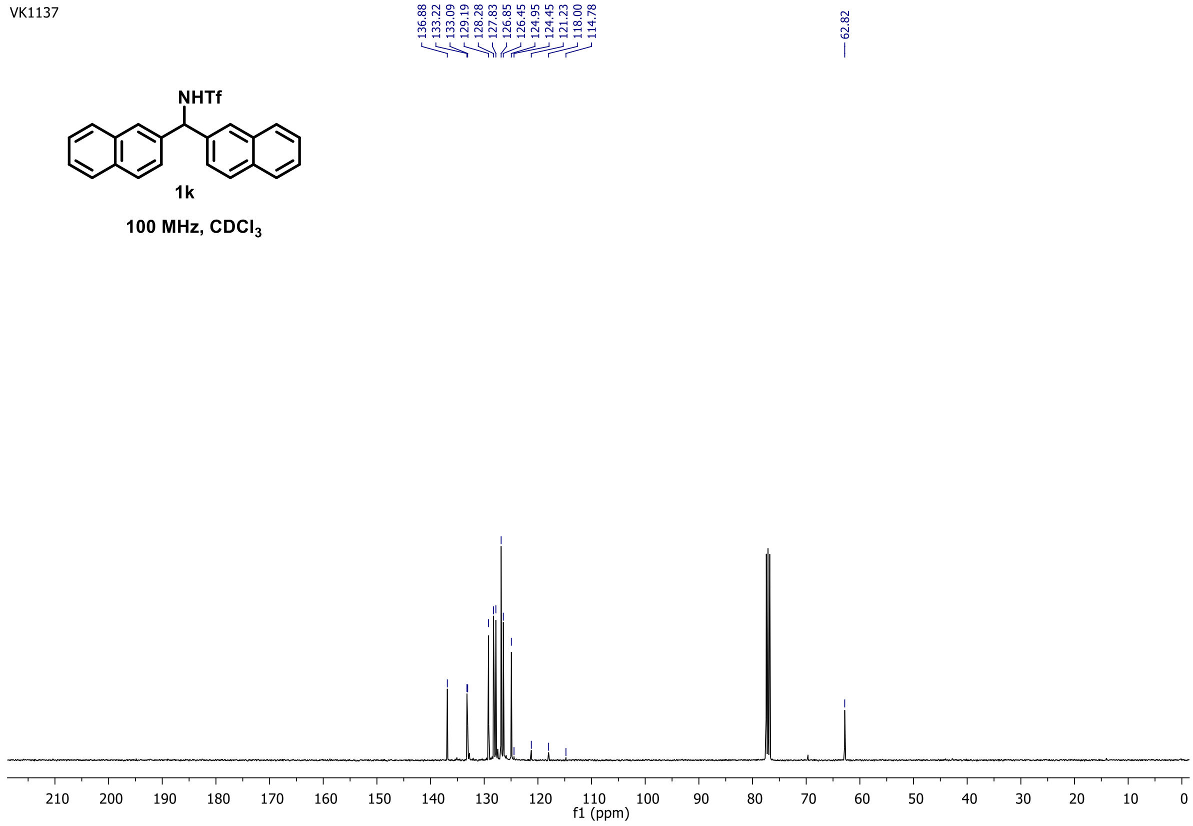
VK1137
136.88
133.22
133.09
129.19
128.28
127.83
126.85
126.45
124.95
124.45
121.23
118.00
114.78
62.82
NHTf
1k
100 MHz, CDCl3
210 200 190 180 170 160 150 140 130 120 110 100 90 80 70 60 50 40 30 20 10 0
f1 (ppm)

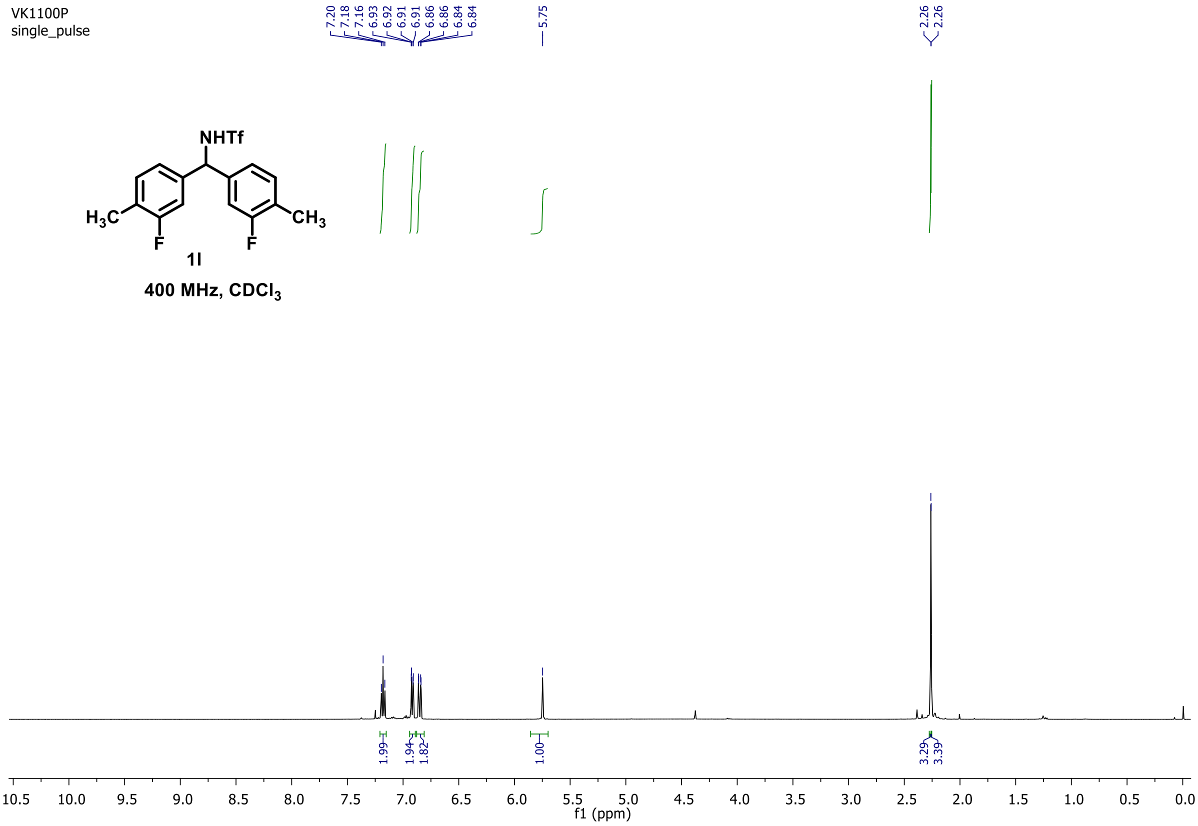
VK1100P
single_pulse
7.20 7.18 7.16 6.93 6.92 6.91 6.91 6.86 6.86 6.84 6.84
5.75
2.26 2.26
NHTf
H3C
CH3
F
F
1l
400 MHz, CDCl3
1.99
1.94
1.82
1.00
3.29
3.39
10.5 10.0 9.5 9.0 8.5 8.0 7.5 7.0 6.5 6.0 5.5 5.0 4.5 4.0 3.5 3.0 2.5 2.0 1.5 1.0 0.5 0.0
f1 (ppm)

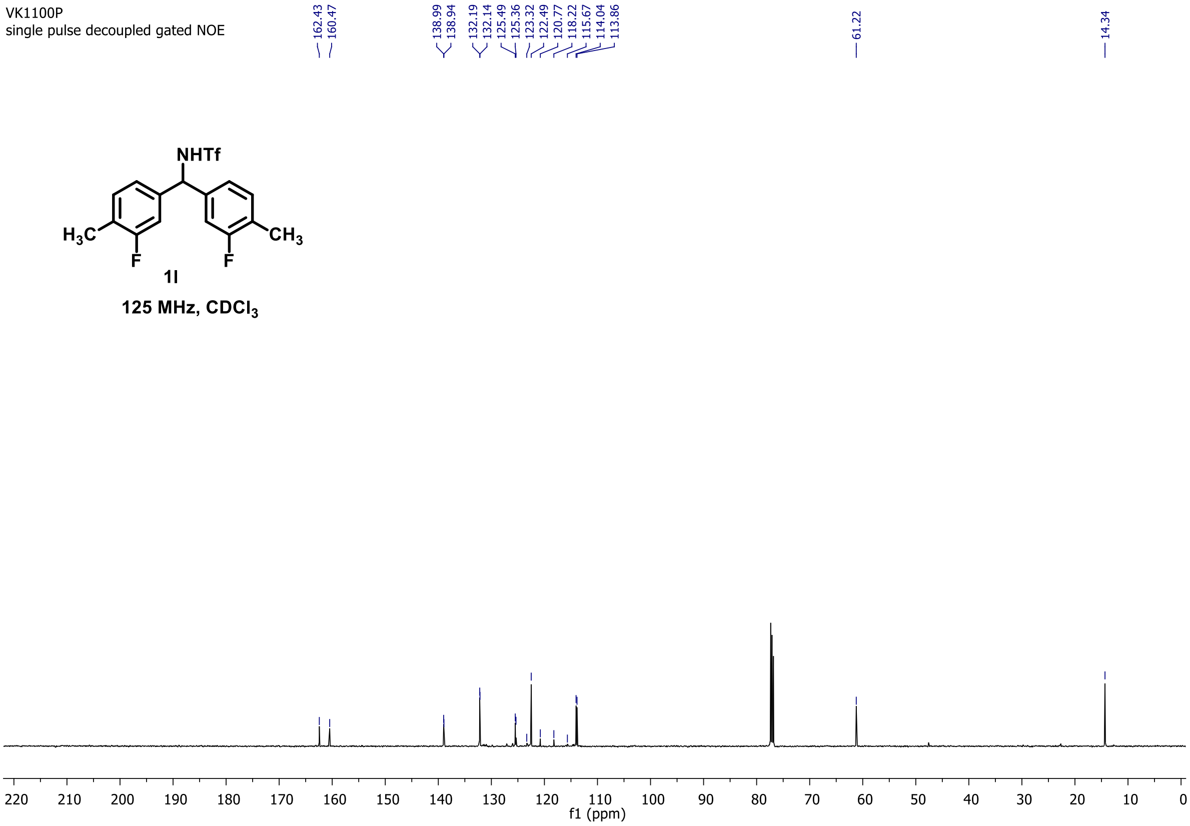
VK1100P
single pulse decoupled gated NOE
162.43 160.47
138.99 138.94 132.19 132.14 125.49 125.36 123.32 122.49 120.77 118.22 115.67 114.04 113.86
61.22
14.34
NHTf
H3C
CH3
F
F
1l
125 MHz, CDCl3
220 210 200 190 180 170 160 150 140 130 120 110 100 90 80 70 60 50 40 30 20 10 0
f1 (ppm)

VK1236PP

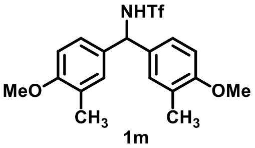

1m

400 MHz, CDCl$_3$

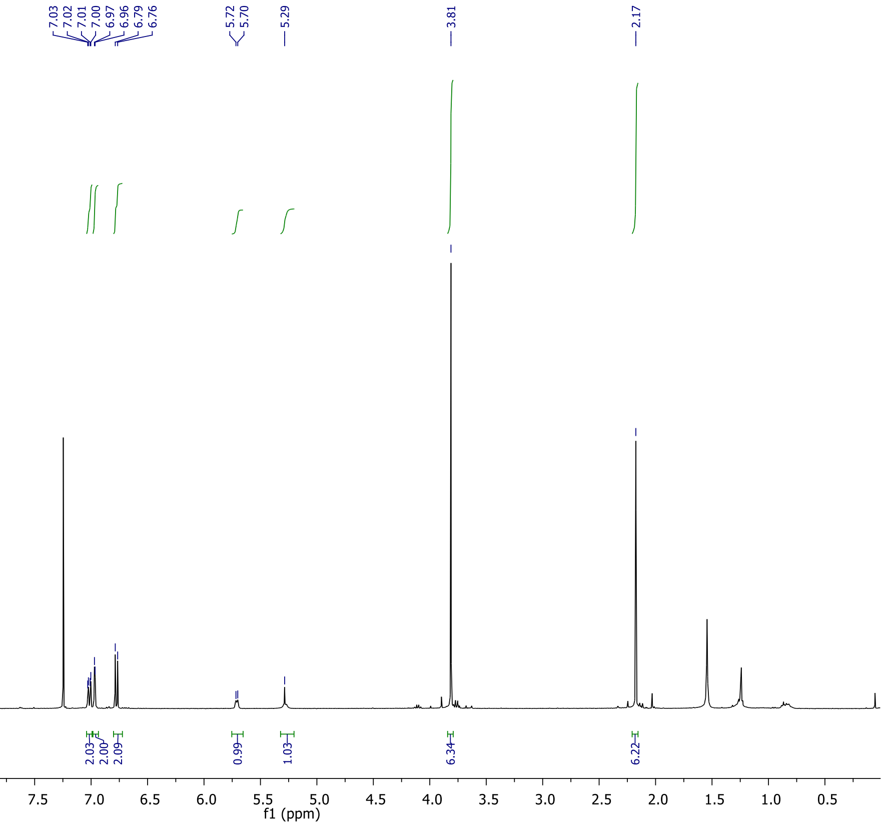

S#613877

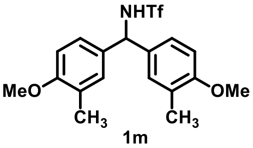

1m

100 MHz, CDCl$_3$

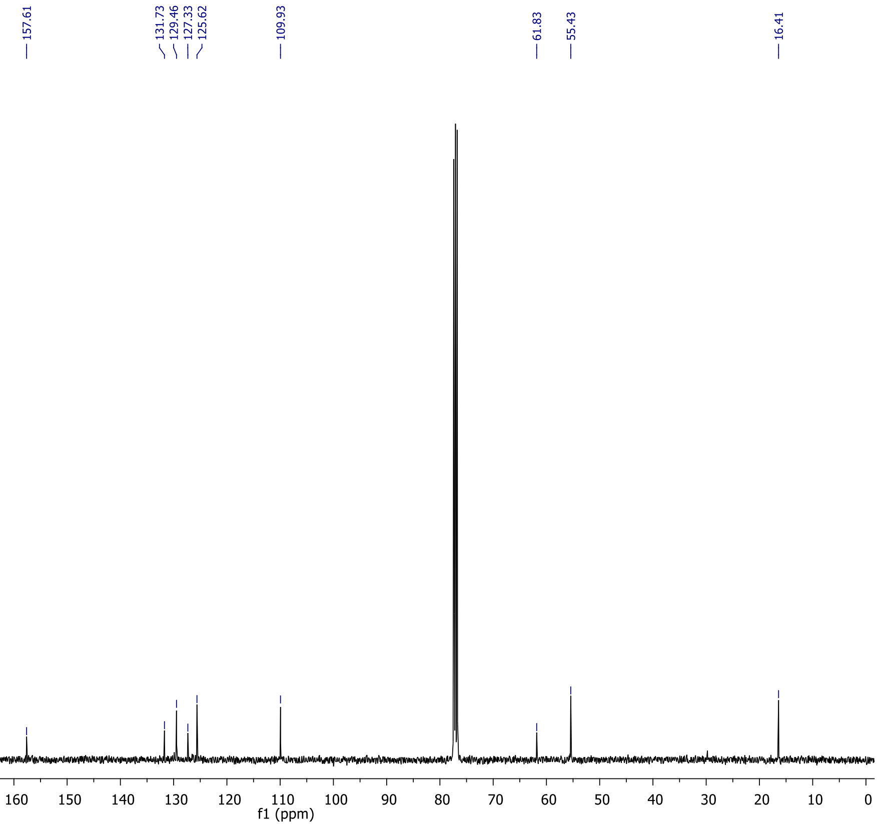

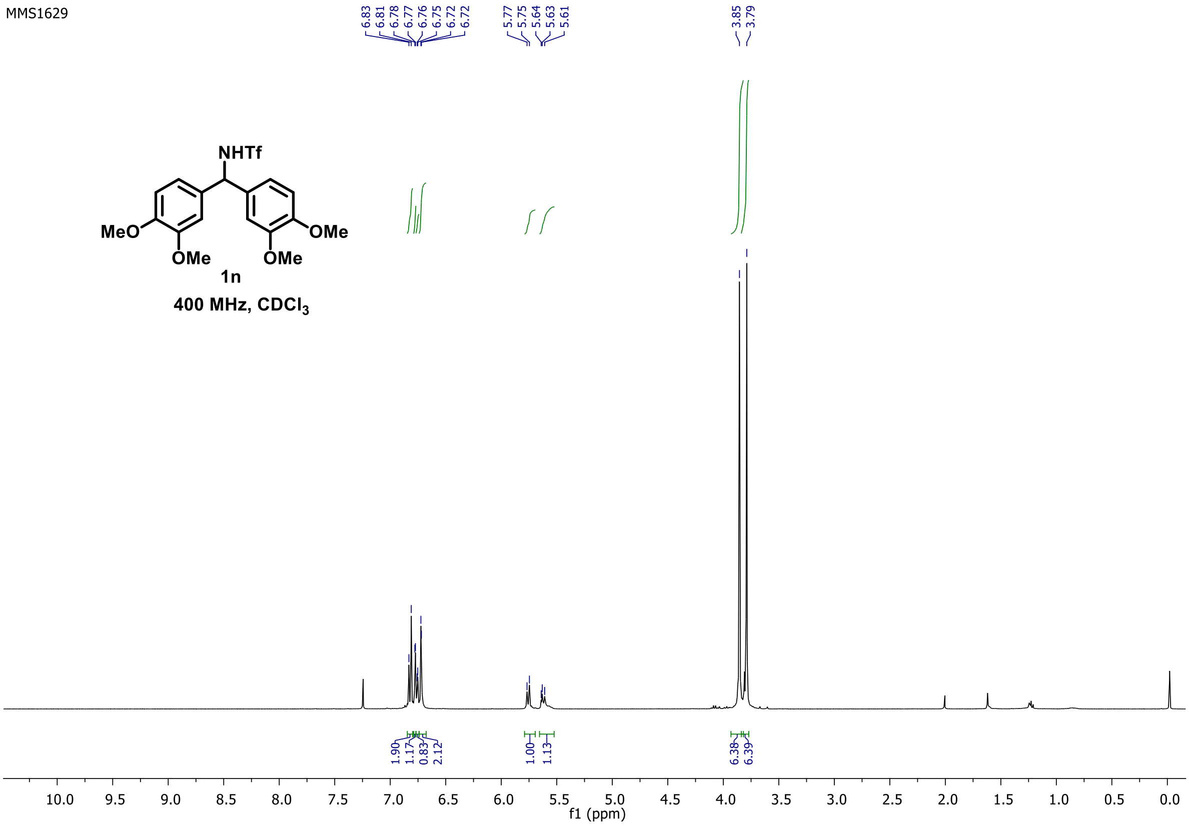
MMS1629
6.83
6.81
6.78
6.77
6.76
6.75
6.72
6.72
5.77
5.75
5.64
5.63
5.61
3.85
3.79
NHTf
MeO
OMe
OMe
OMe
1n
400 MHz, CDCl3
1.90
1.17
0.83
2.12
1.00
1.13
6.38
6.39
10.0 9.5 9.0 8.5 8.0 7.5 7.0 6.5 6.0 5.5 5.0 4.5 4.0 3.5 3.0 2.5 2.0 1.5 1.0 0.5 0.0
f1 (ppm)

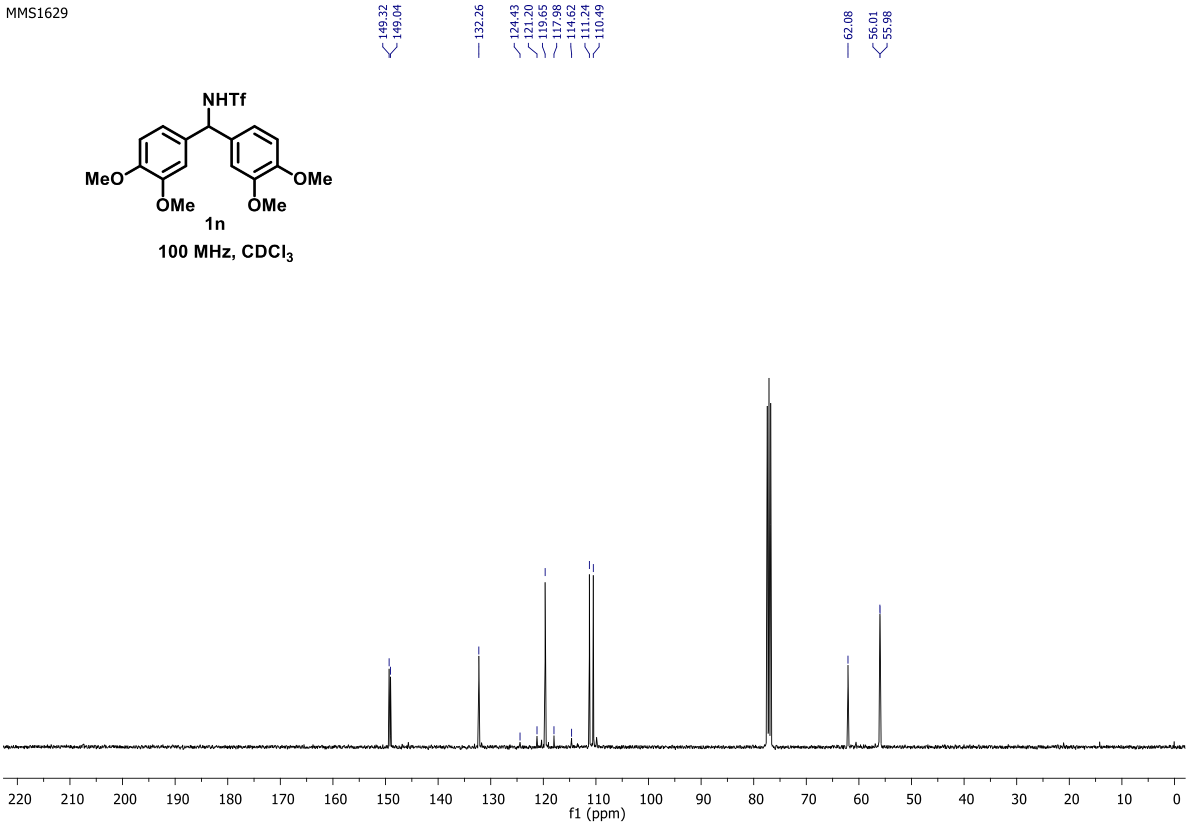
MMS1629
149.32
149.04
132.26
124.43
121.20
119.65
117.98
114.62
111.24
110.49
62.08
56.01
55.98
NHTf
MeO
OMe
OMe
OMe
1n
100 MHz, CDCl3
220 210 200 190 180 170 160 150 140 130 120 110 100 90 80 70 60 50 40 30 20 10 0
f1 (ppm)

5. NMR spectra of compound 3 and 4

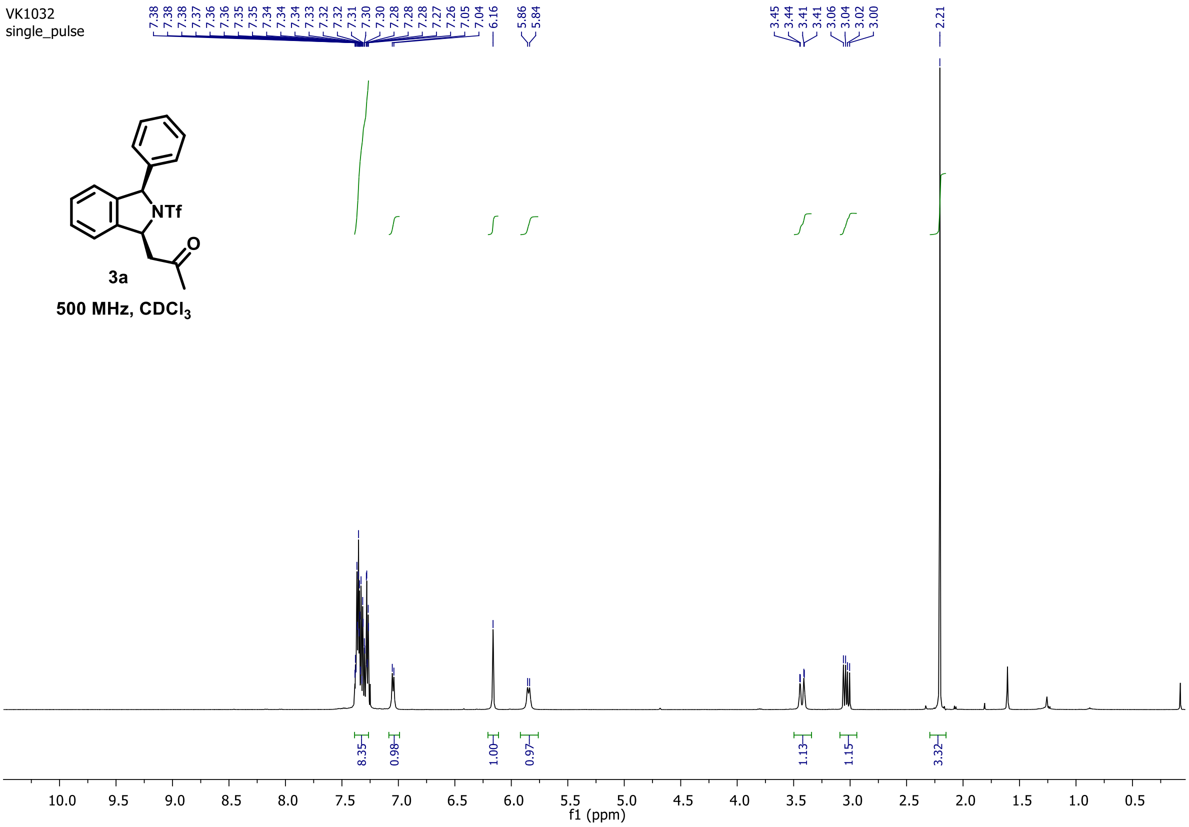

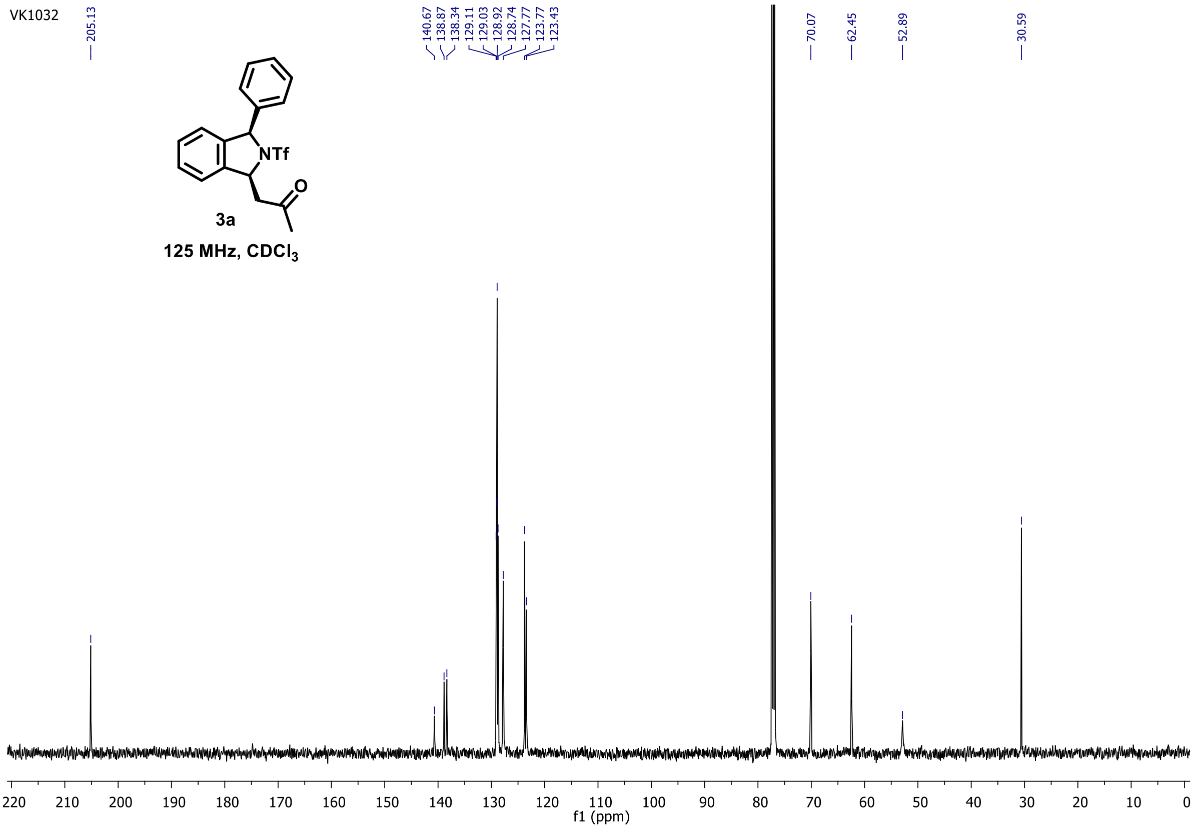

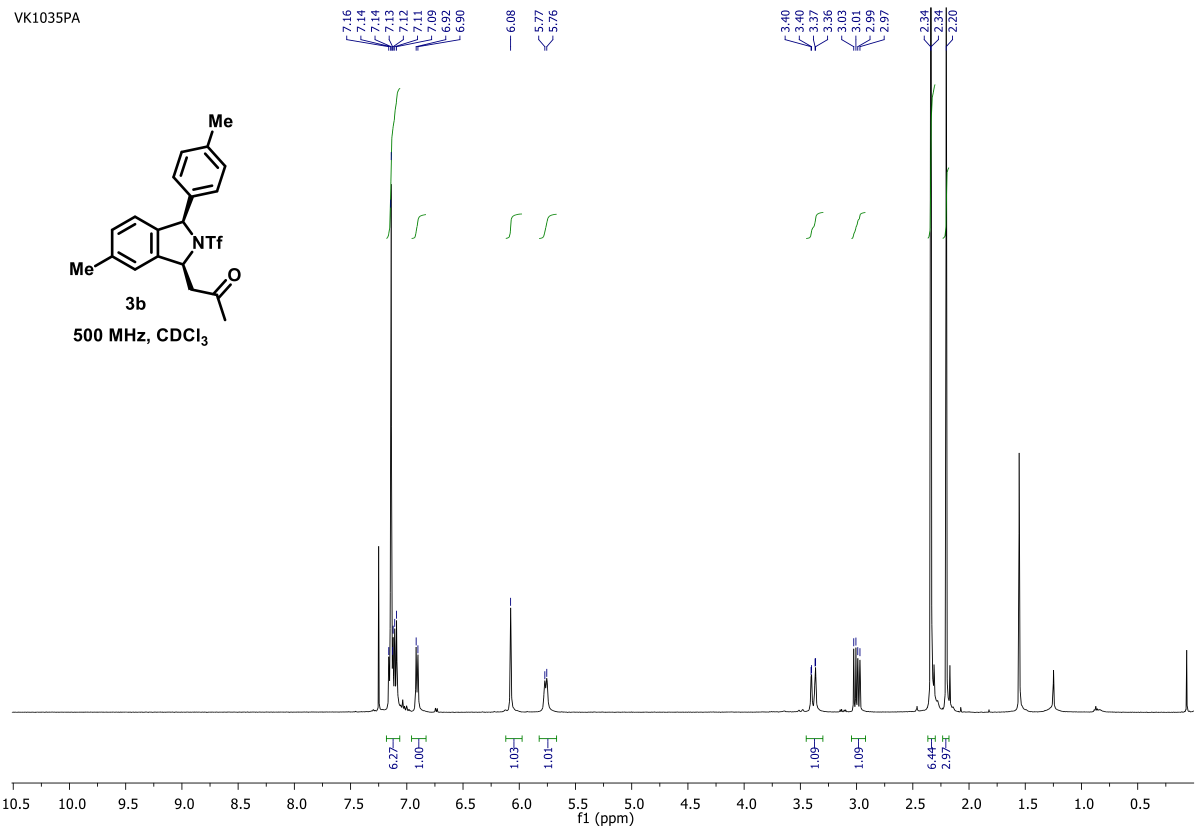
VK1035PA
7.16 7.14 7.14 7.13 7.12 7.11 7.09 6.92 6.90
6.08
5.77 5.76
3.40 3.40 3.37 3.36 3.03 3.01 2.99 2.97
2.34 2.34 2.20
Me
NTf
Me
O
3b
500 MHz, CDCl3
6.27 1.00 1.03 1.01 1.09 1.09 6.44 2.97
10.5 10.0 9.5 9.0 8.5 8.0 7.5 7.0 6.5 6.0 5.5 5.0 4.5 4.0 3.5 3.0 2.5 2.0 1.5 1.0 0.5
f1 (ppm)

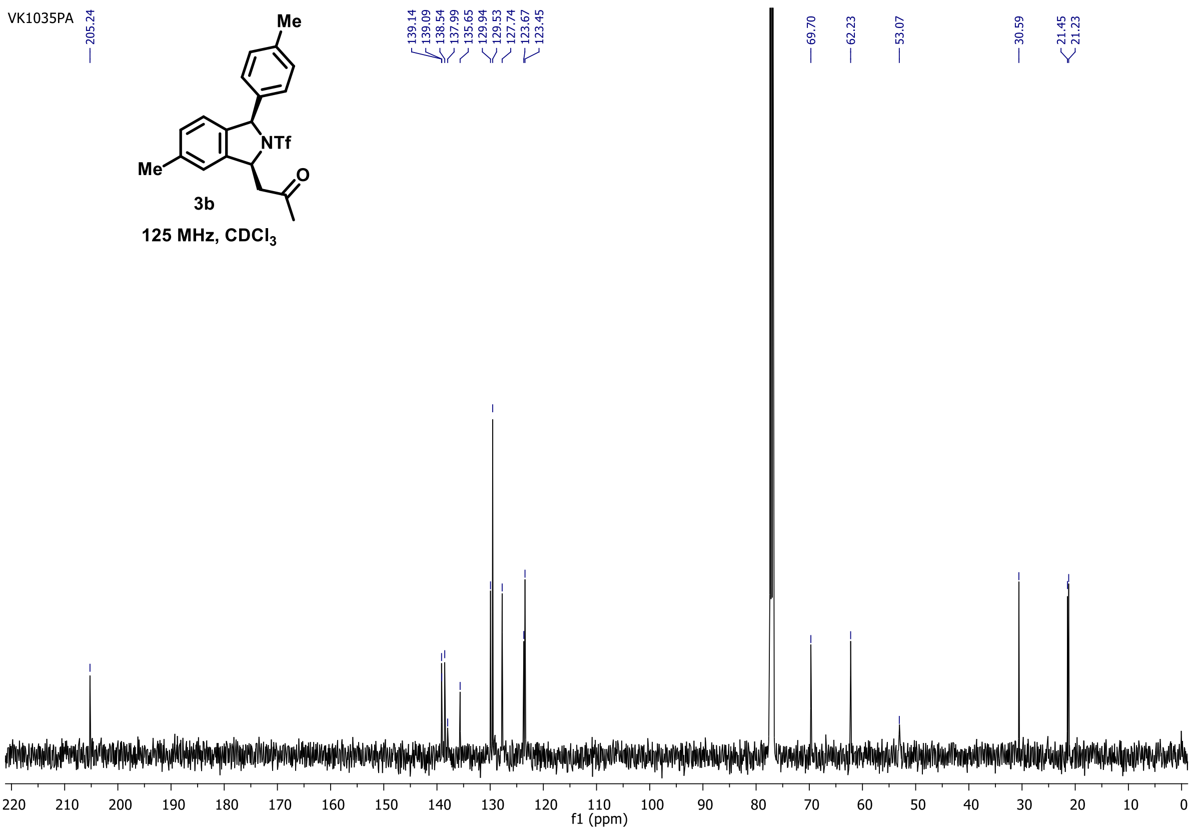
VK1035PA
205.24
139.14 139.09 138.54 137.99 135.65 129.94 129.53 127.74 123.67 123.45
69.70
62.23
53.07
30.59
21.45 21.23
Me
NTf
Me
O
3b
125 MHz, CDCl3
220 210 200 190 180 170 160 150 140 130 120 110 100 90 80 70 60 50 40 30 20 10 0
f1 (ppm)

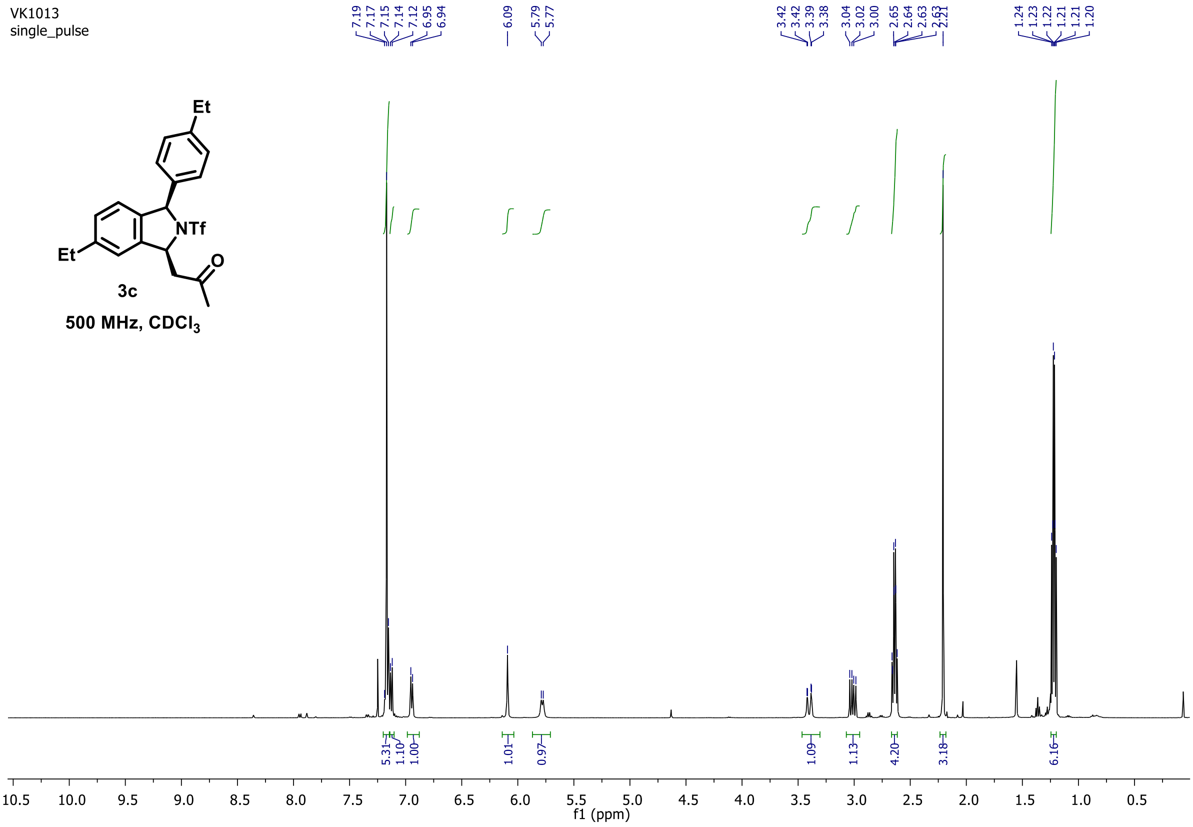
VK1013
single_pulse
7.19 7.17 7.15 7.14 7.12 6.95 6.94 6.09 5.79 5.77 3.42 3.42 3.39 3.38 3.04 3.02 3.00 2.65 2.64 2.63 2.63 2.21 1.24 1.23 1.22 1.21 1.21 1.20
Et
NTf
O
Et
3c
500 MHz, $CDCl_3$
5.31 1.10 1.00 1.01 0.97 1.09 1.13 4.20 3.18 6.16
10.5 10.0 9.5 9.0 8.5 8.0 7.5 7.0 6.5 6.0 5.5 5.0 4.5 4.0 3.5 3.0 2.5 2.0 1.5 1.0 0.5
f1 (ppm)

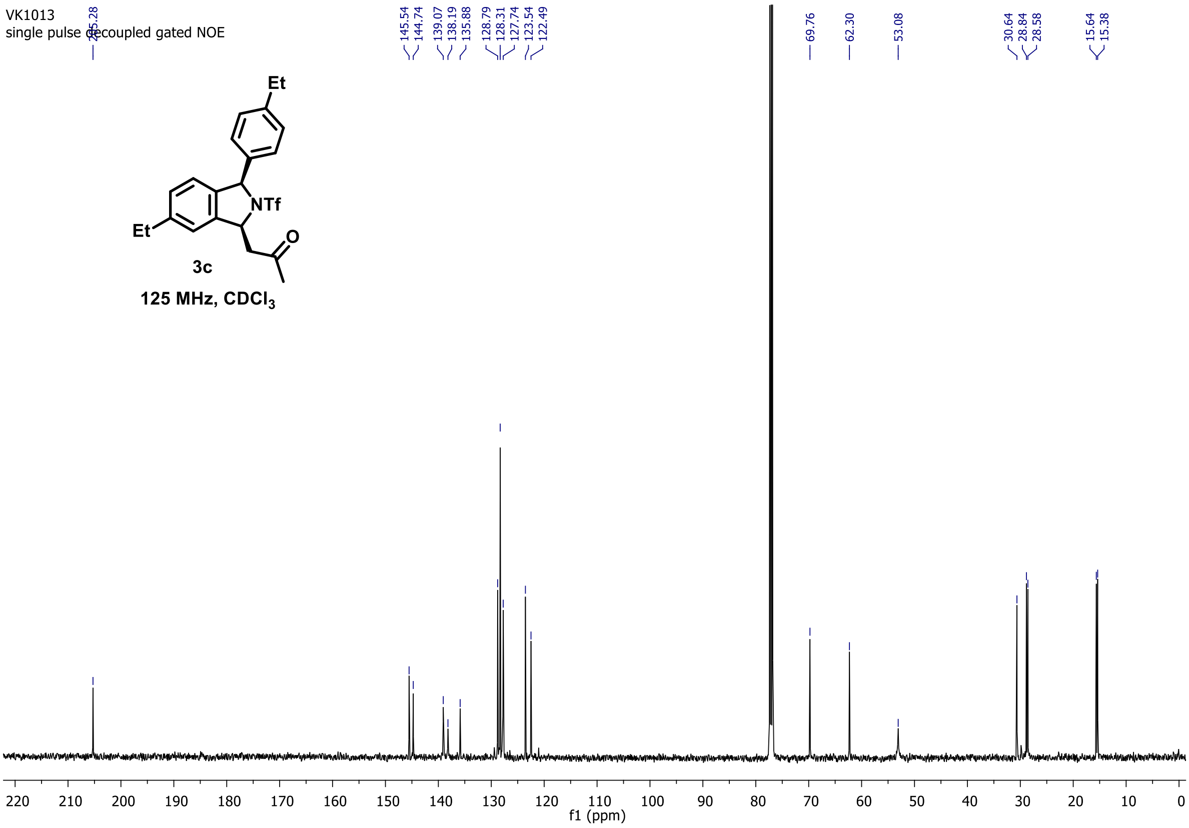
VK1013
single pulse decoupled gated NOE
205.28 145.54 144.74 139.07 138.19 135.88 128.79 128.31 127.74 123.54 122.49 69.76 62.30 53.08 30.64 28.84 28.58 15.64 15.38
Et
NTf
O
Et
3c
125 MHz, $CDCl_3$
220 210 200 190 180 170 160 150 140 130 120 110 100 90 80 70 60 50 40 30 20 10 0
f1 (ppm)

3d

400 MHz, $CDCl_3$

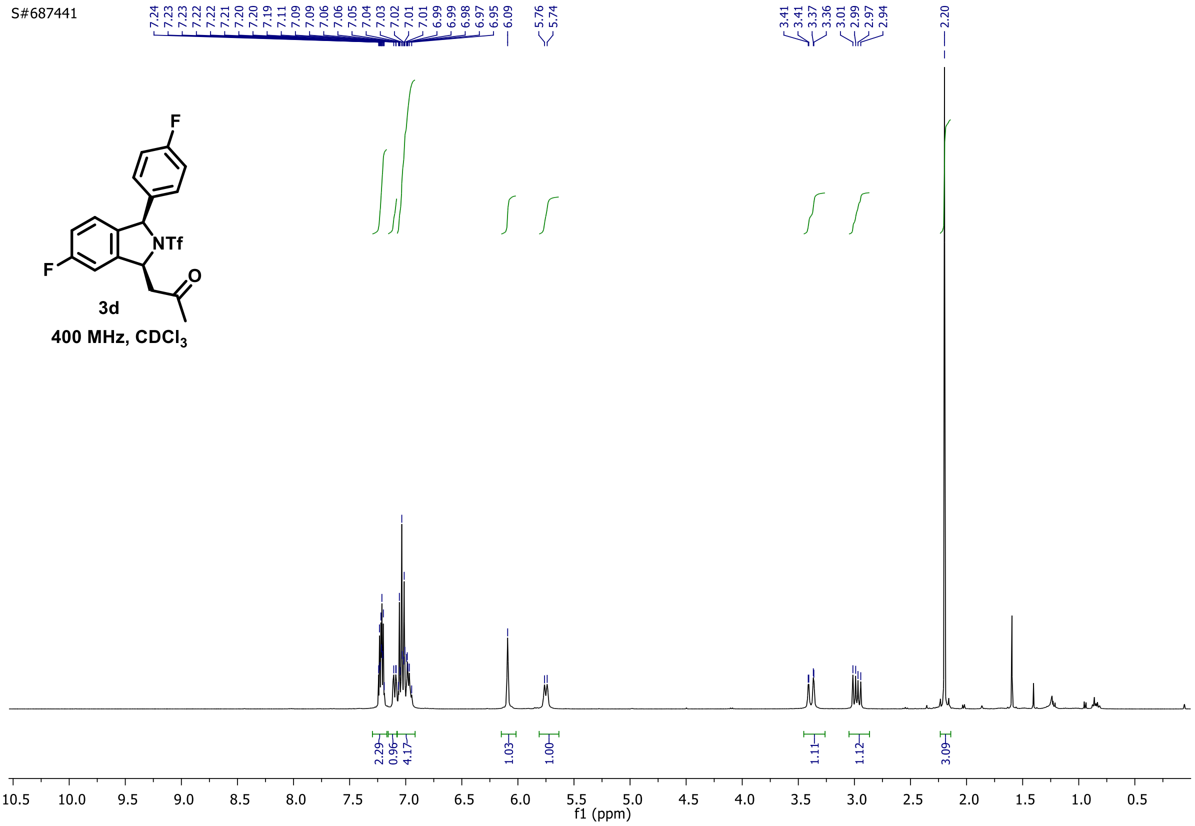

3d

125 MHz, $CDCl_3$

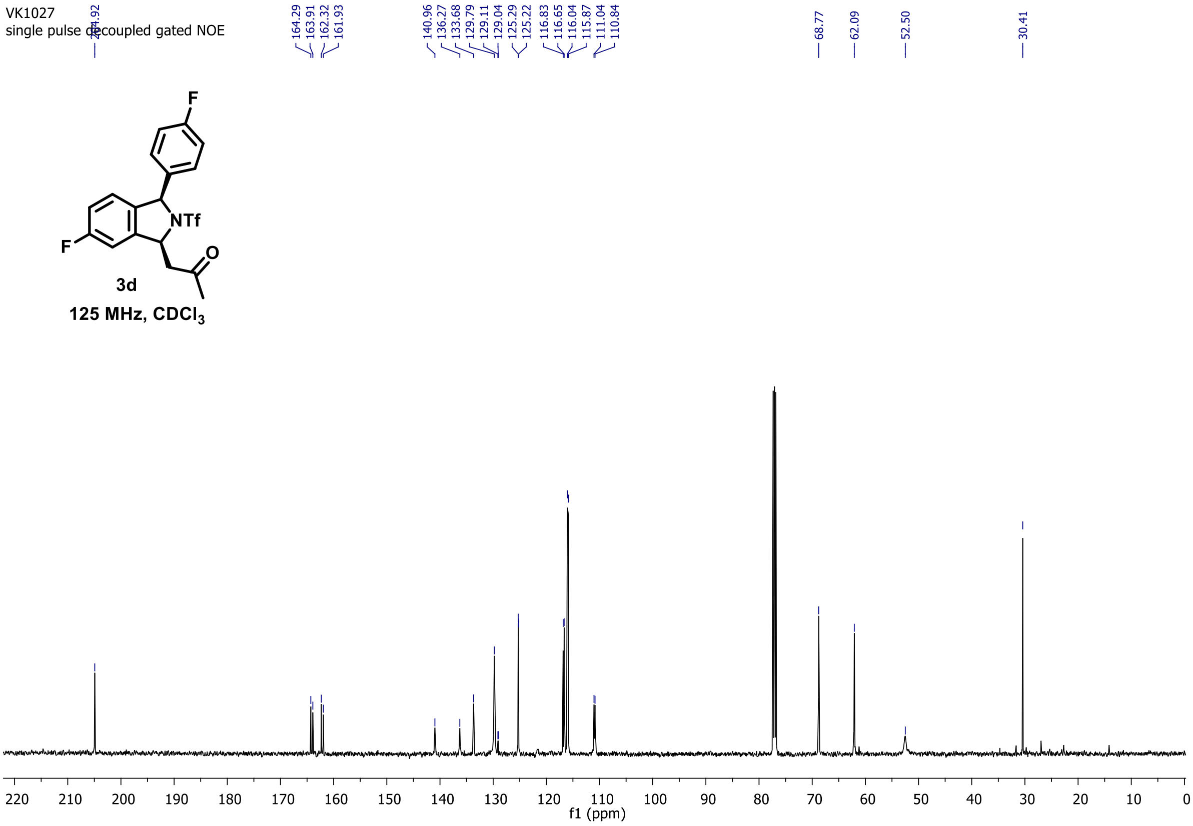

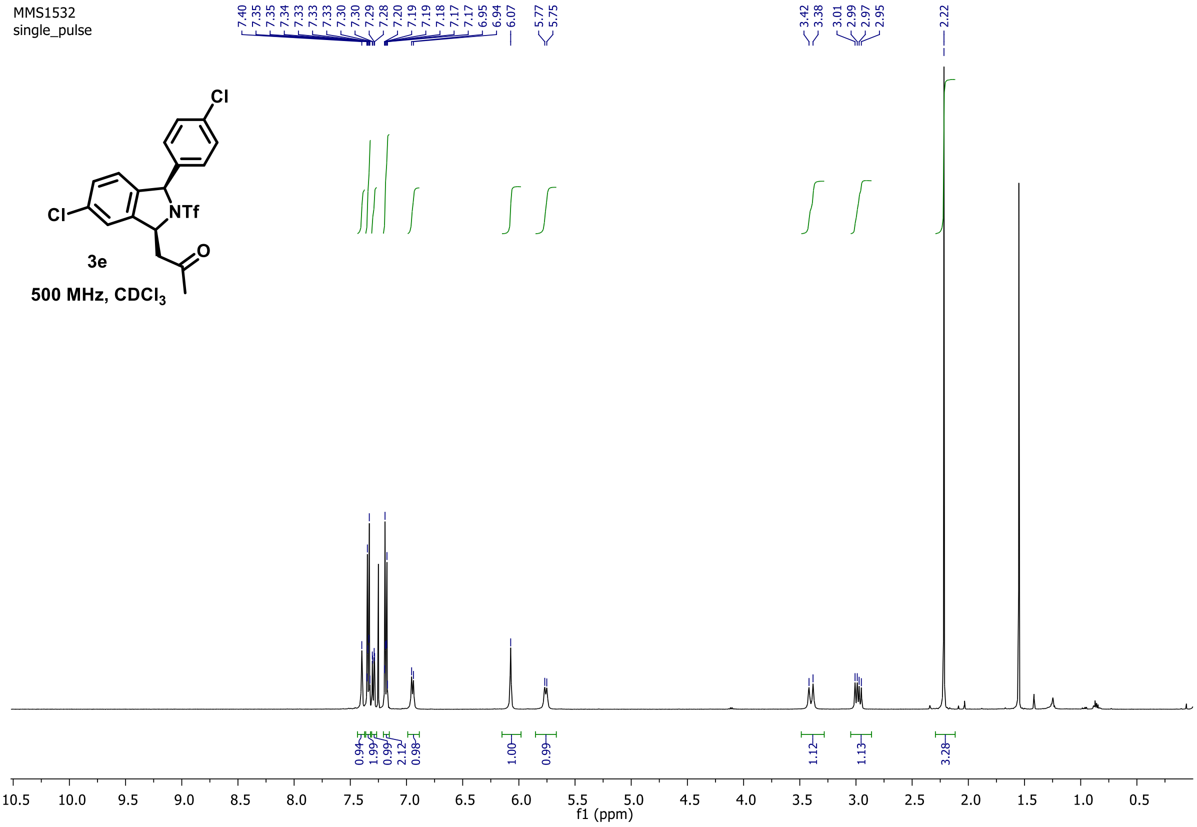
MMS1532
single_pulse
7.40 7.35 7.35 7.34 7.33 7.33 7.33 7.30 7.29 7.28 7.20 7.19 7.19 7.18 7.17 7.17 6.95 6.94 6.07 5.77 5.75
3.42 3.38 3.01 2.99 2.97 2.95 2.22
Cl
NTf
Cl
3e
500 MHz, CDCl3
0.94 1.99 0.99 2.12 0.98 1.00 0.99 1.12 1.13 3.28
10.5 10.0 9.5 9.0 8.5 8.0 7.5 7.0 6.5 6.0 5.5 5.0 4.5 4.0 3.5 3.0 2.5 2.0 1.5 1.0 0.5
f1 (ppm)

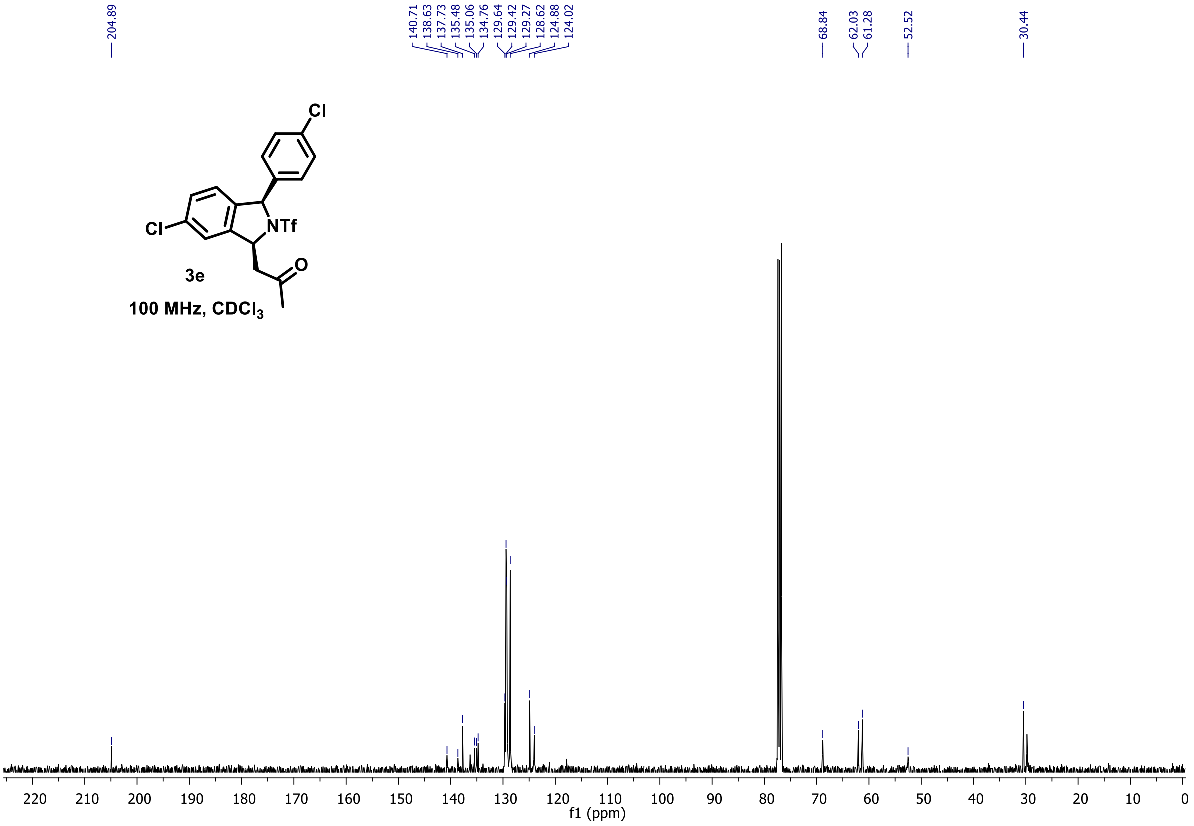
204.89
140.71 138.63 137.73 135.48 135.06 134.76 129.64 129.42 129.27 128.62 124.88 124.02
68.84 62.03 61.28 52.52 30.44
Cl
NTf
Cl
3e
100 MHz, CDCl3
220 210 200 190 180 170 160 150 140 130 120 110 100 90 80 70 60 50 40 30 20 10 0
f1 (ppm)

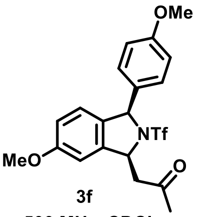

3f
500 MHz, $CDCl_3$

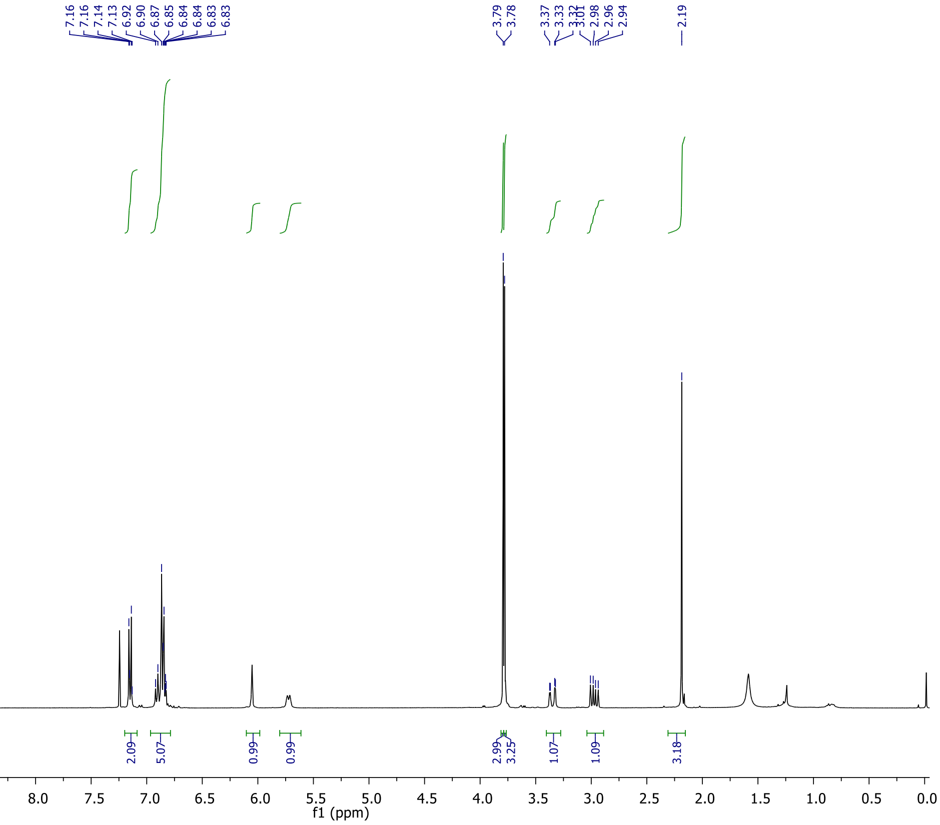

VK979PA1_5000

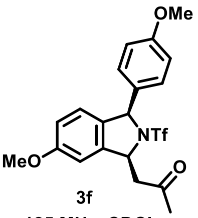

3f
125 MHz, $CDCl_3$

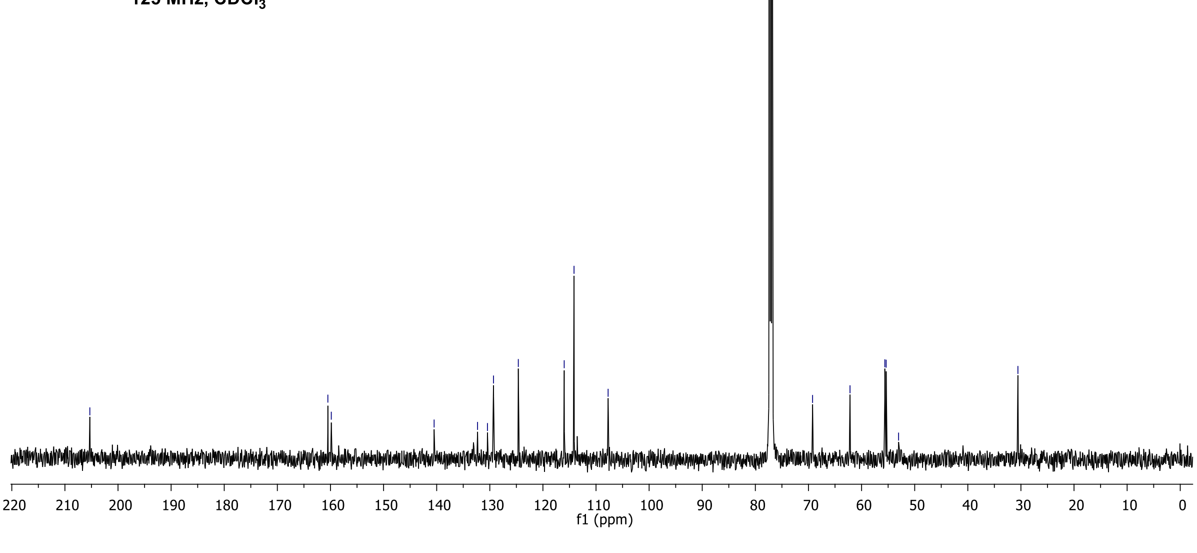

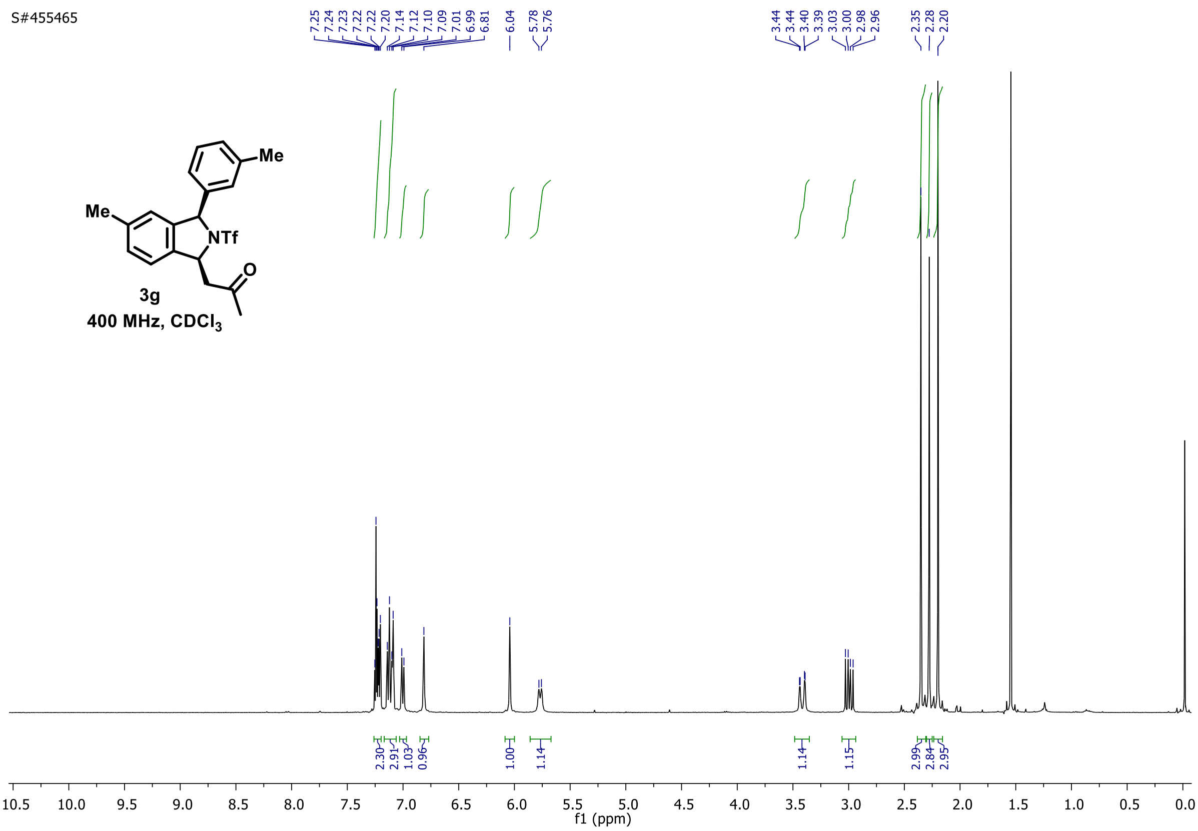
S#455465
7.25 7.24 7.23 7.22 7.22 7.20 7.14 7.12 7.10 7.09 7.01 6.99 6.81
6.04
5.78 5.76
3.44 3.44 3.40 3.39 3.03 3.00 2.98 2.96
2.35 2.28 2.20
Me
Me
NTf
O
3g
400 MHz, CDCl3
2.30 2.91 1.03 0.96 1.00 1.14 1.14 1.15 2.99 2.84 2.95
f1 (ppm)

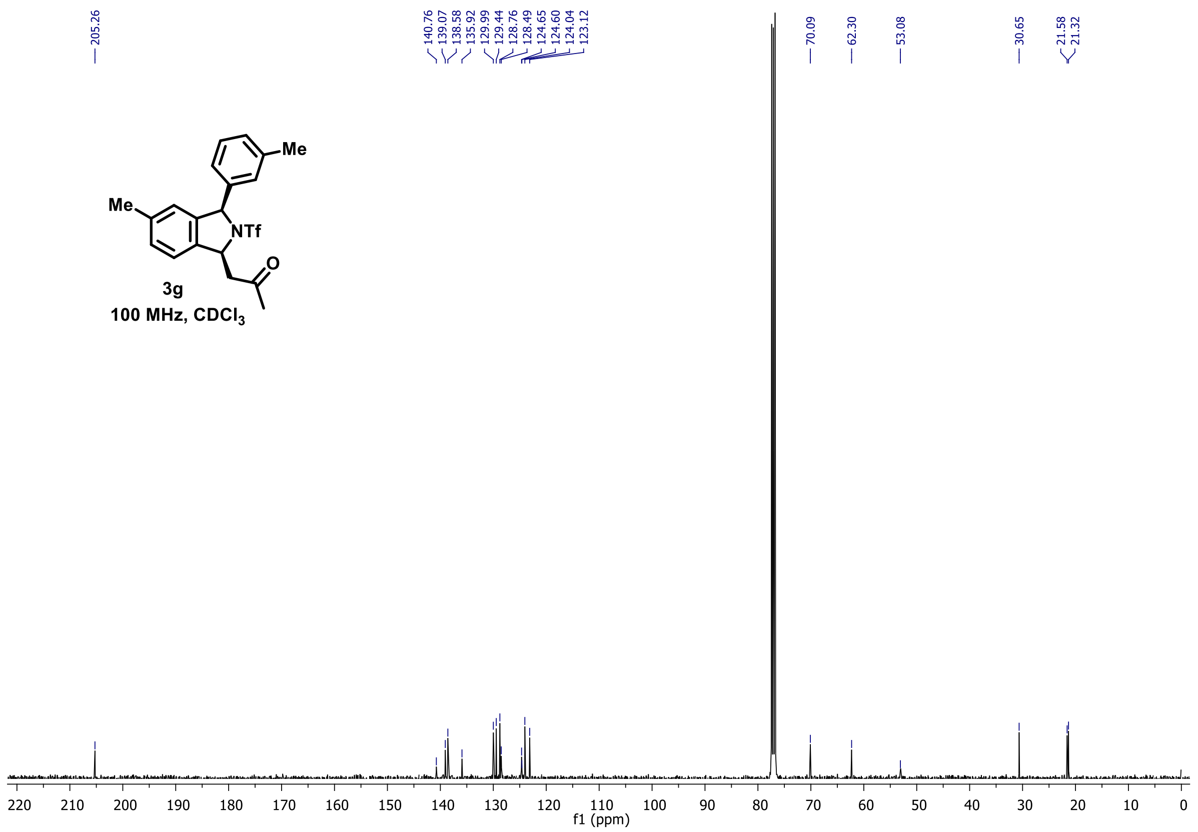
205.26
140.76 139.07 138.58 135.92 129.99 129.44 128.76 128.49 124.65 124.60 124.04 123.12
70.09
62.30
53.08
30.65
21.58 21.32
Me
Me
NTf
O
3g
100 MHz, CDCl3
f1 (ppm)

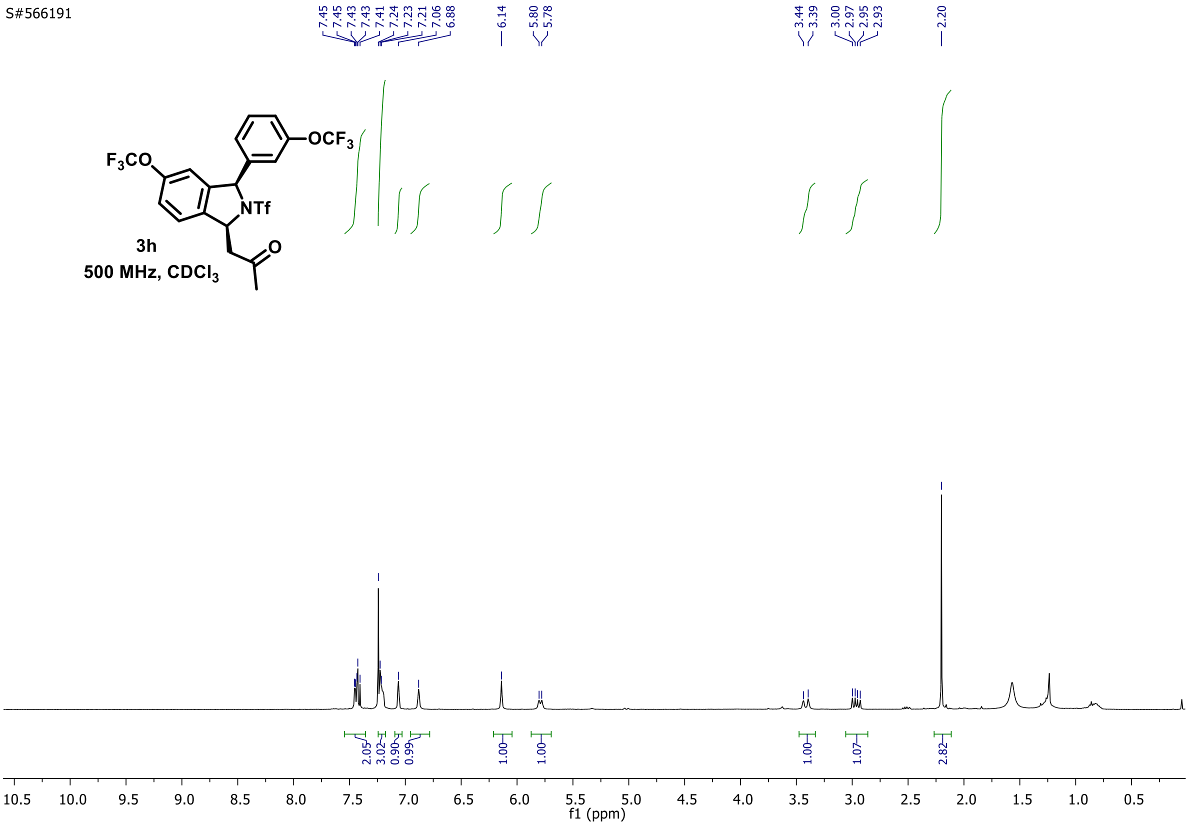
S#566191
7.45 7.45 7.43 7.43 7.41 7.24 7.23 7.21 7.06 6.88 6.14 5.80 5.78 3.44 3.39 3.00 2.97 2.95 2.93 2.20
F3CO
OCF3
NTf
O
3h
500 MHz, CDCl3
2.05 3.02 0.90 0.99 1.00 1.00 1.00 1.07 2.82
10.5 10.0 9.5 9.0 8.5 8.0 7.5 7.0 6.5 6.0 5.5 5.0 4.5 4.0 3.5 3.0 2.5 2.0 1.5 1.0 0.5
f1 (ppm)

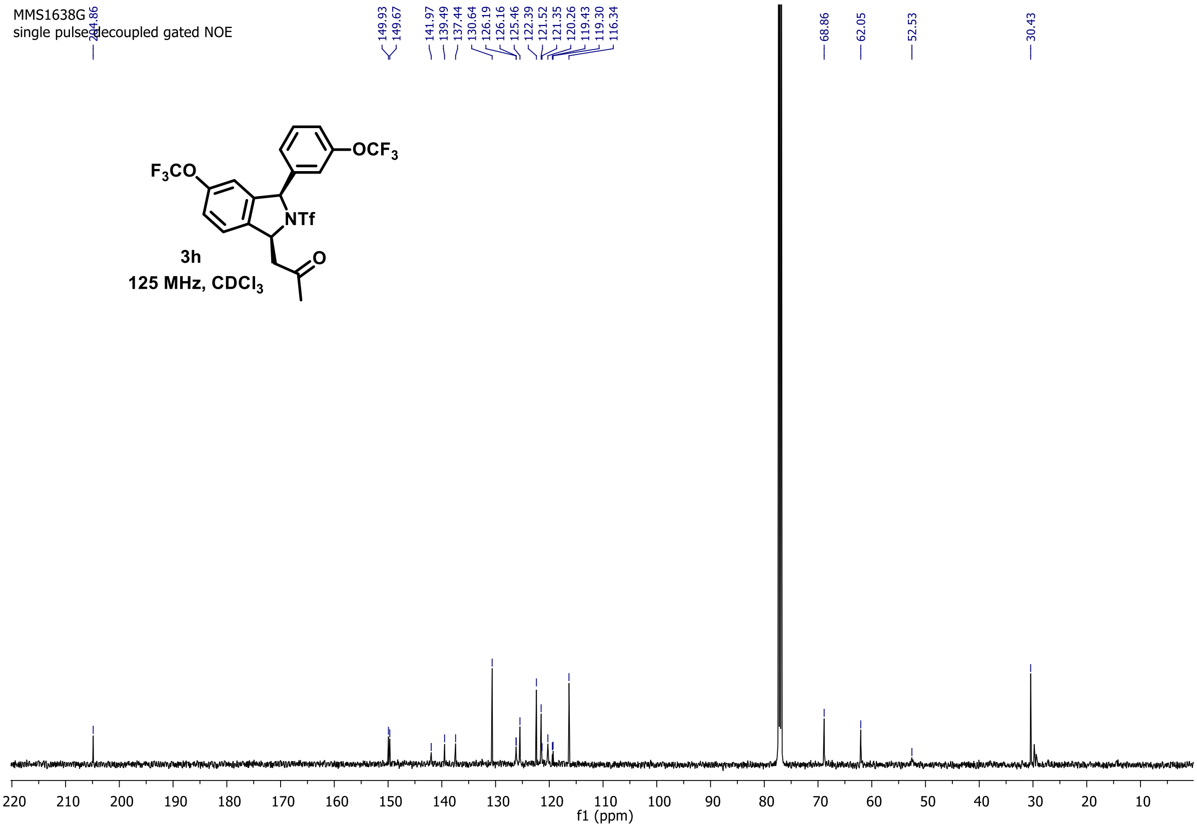
MMS1638G
single pulse decoupled gated NOE
204.86
149.93 149.67 141.97 139.49 137.44 130.64 126.19 126.16 125.46 122.39 121.52 121.35 120.26 119.43 119.30 116.34 68.86 62.05 52.53 30.43
F3CO
OCF3
NTf
O
3h
125 MHz, CDCl3
220 210 200 190 180 170 160 150 140 130 120 110 100 90 80 70 60 50 40 30 20 10
f1 (ppm)

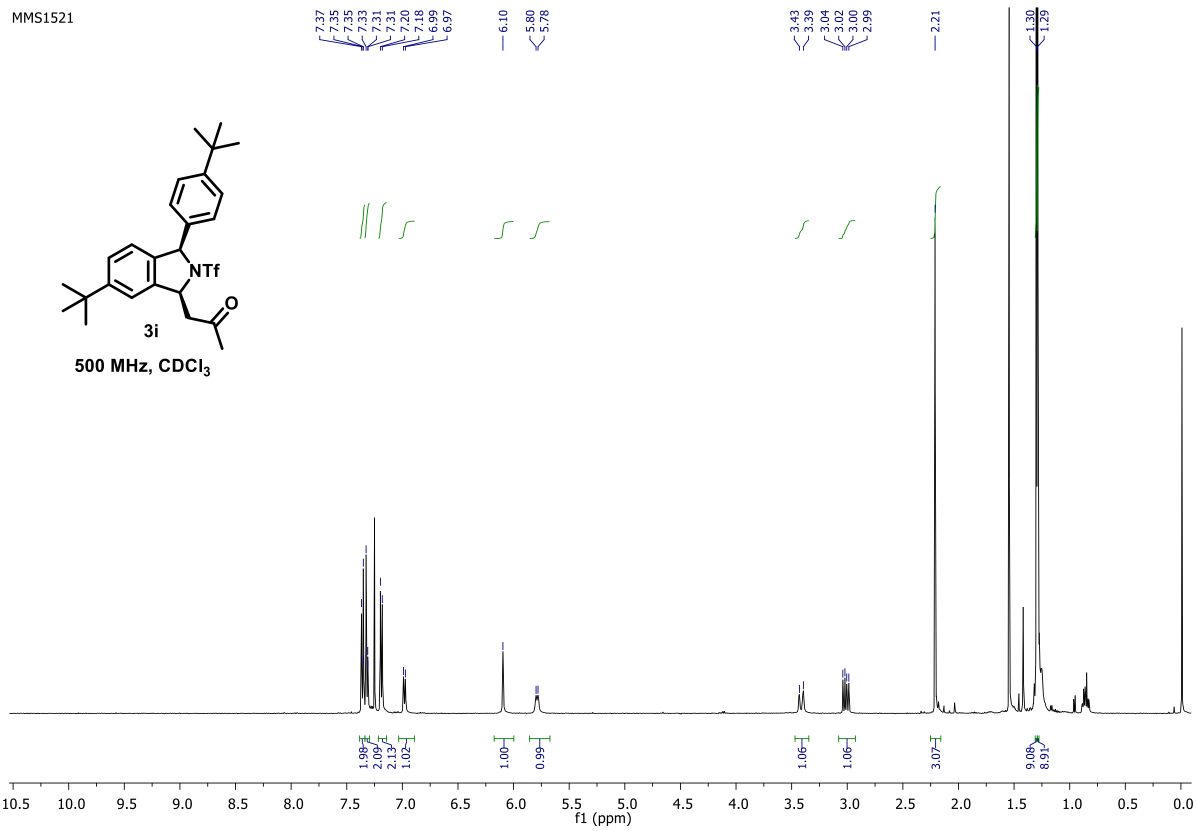
MMS1521
7.37 7.35 7.35 7.33 7.31 7.31 7.20 7.18 6.99 6.97
6.10
5.80 5.78
3.43 3.39 3.04 3.02 3.00 2.99
2.21
1.30 1.29
NTf
O
3i
500 MHz, $CDCl_3$
1.98 2.09 2.13 1.02
1.00
0.99
1.06
1.06
3.07
9.08 8.91
10.5 10.0 9.5 9.0 8.5 8.0 7.5 7.0 6.5 6.0 5.5 5.0 4.5 4.0 3.5 3.0 2.5 2.0 1.5 1.0 0.5 0.0
f1 (ppm)

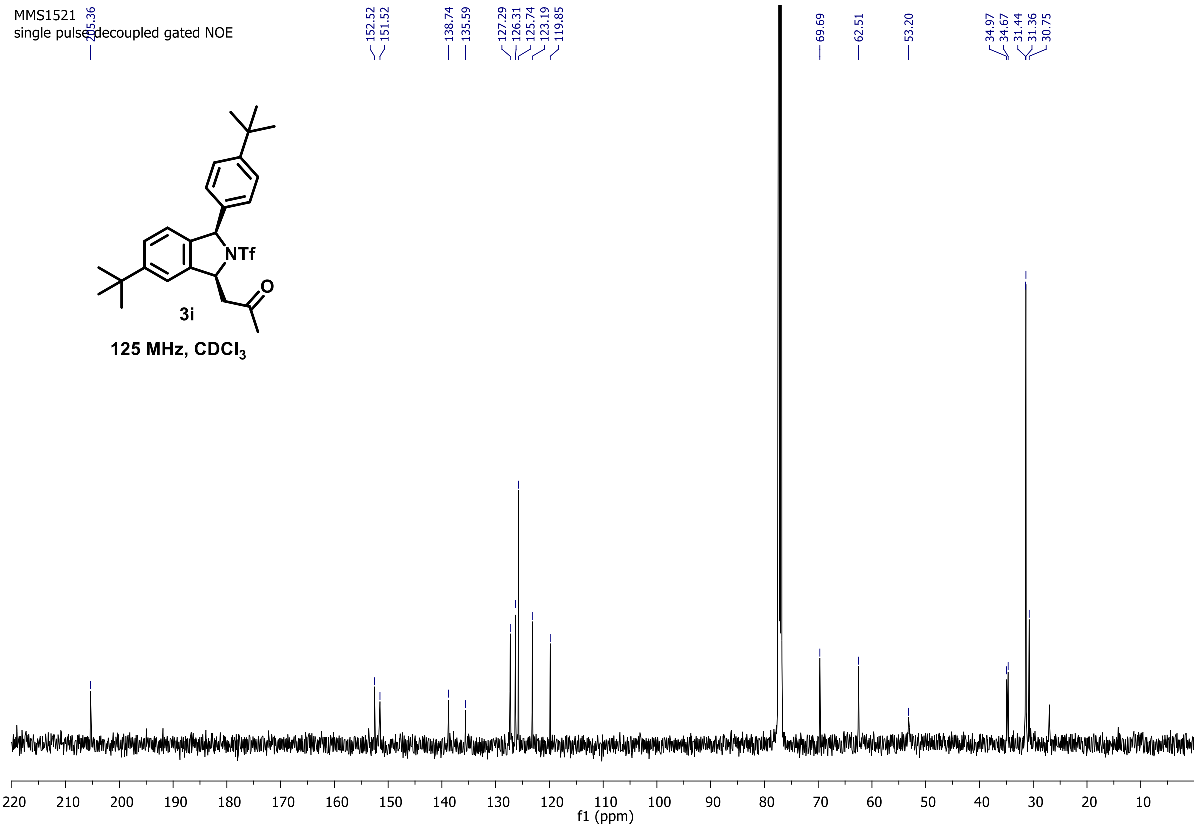
MMS1521
single pulse decoupled gated NOE
205.36
152.52 151.52
138.74 135.59
127.29 126.31 125.74 123.19 119.85
69.69
62.51
53.20
34.97 34.67 31.44 31.36 30.75
NTf
O
3i
125 MHz, $CDCl_3$
220 210 200 190 180 170 160 150 140 130 120 110 100 90 80 70 60 50 40 30 20 10
f1 (ppm)

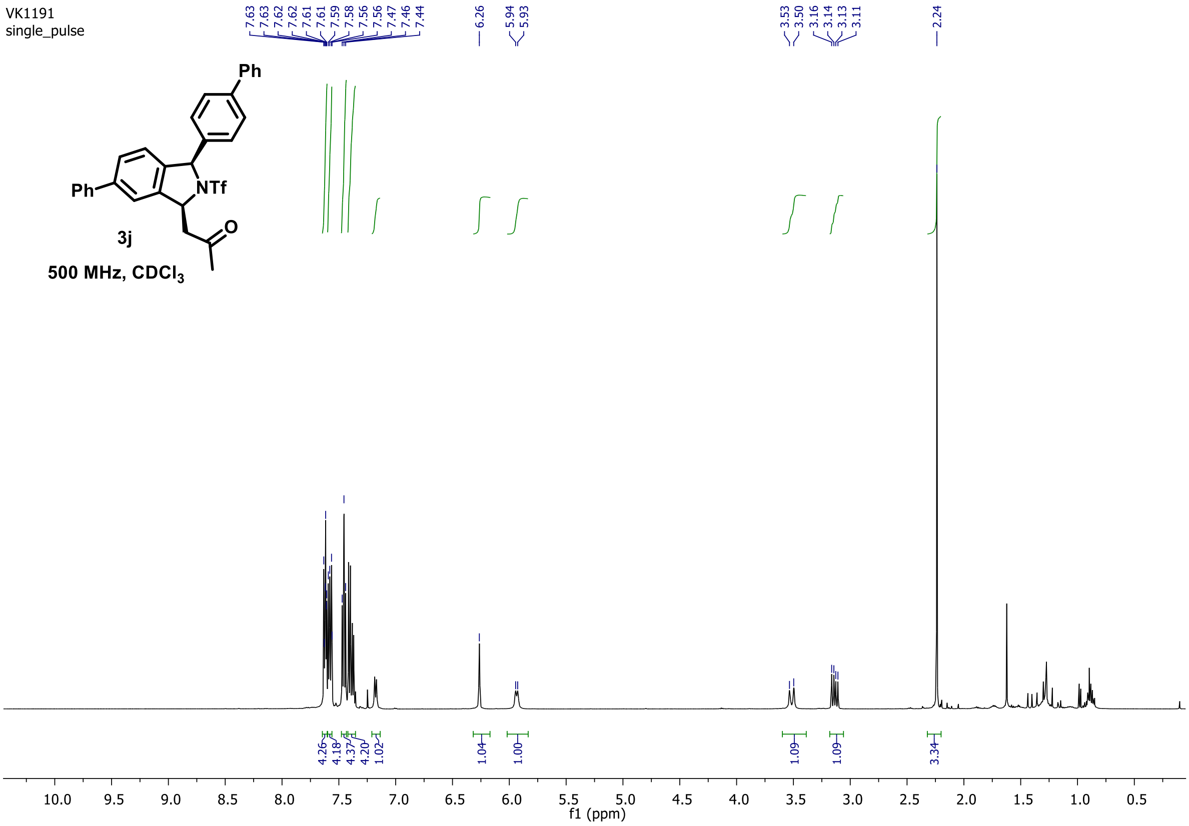
VK1191
single_pulse
7.63 7.63 7.62 7.62 7.61 7.61 7.59 7.58 7.56 7.56 7.47 7.46 7.44
6.26
5.94 5.93
3.53 3.50 3.16 3.14 3.13 3.11
2.24
Ph
Ph
NTf
O
3j
500 MHz, CDCl3
4.26 4.18 4.37 4.20 1.02
1.04
1.00
1.09
1.09
3.34
10.0 9.5 9.0 8.5 8.0 7.5 7.0 6.5 6.0 5.5 5.0 4.5 4.0 3.5 3.0 2.5 2.0 1.5 1.0 0.5
f1 (ppm)

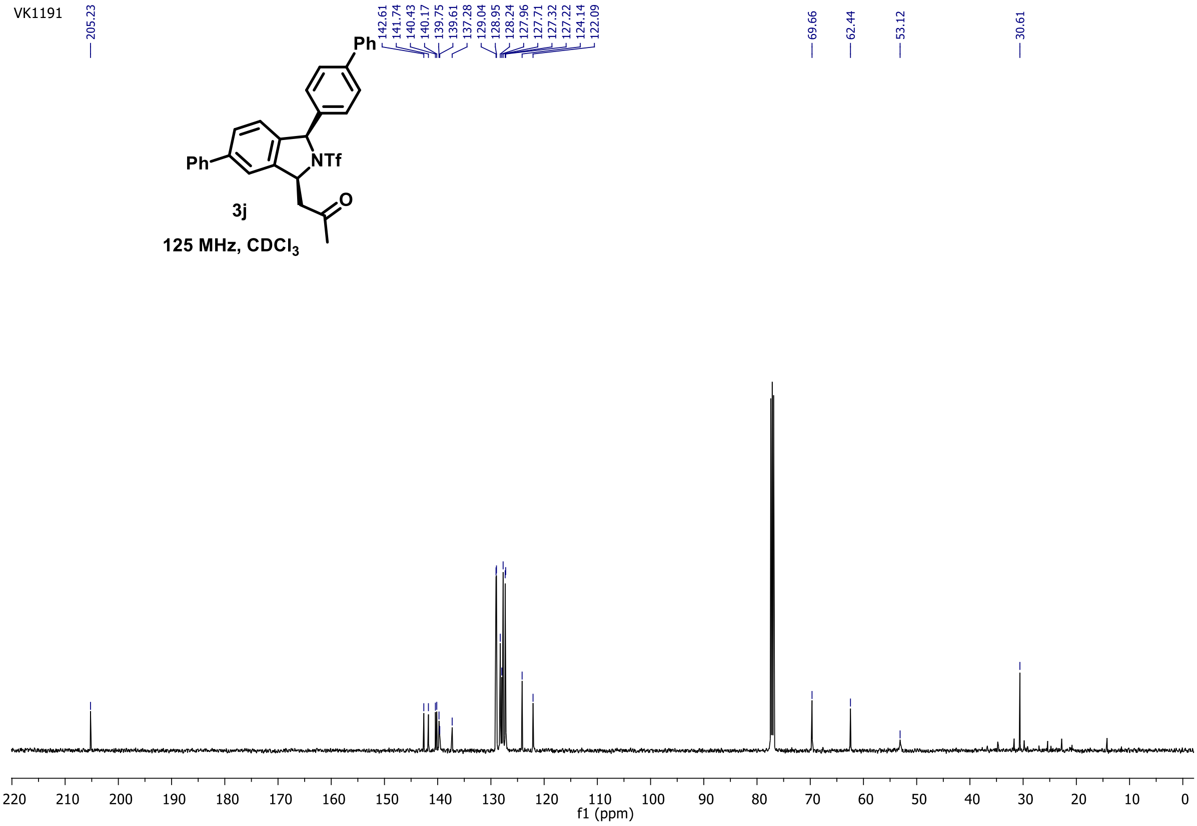
VK1191
205.23
142.61 141.74 140.43 140.17 139.75 139.61 137.28 129.04 128.95 128.24 127.96 127.71 127.32 127.22 124.14 122.09
69.66
62.44
53.12
30.61
Ph
Ph
NTf
O
3j
125 MHz, CDCl3
220 210 200 190 180 170 160 150 140 130 120 110 100 90 80 70 60 50 40 30 20 10 0
f1 (ppm)

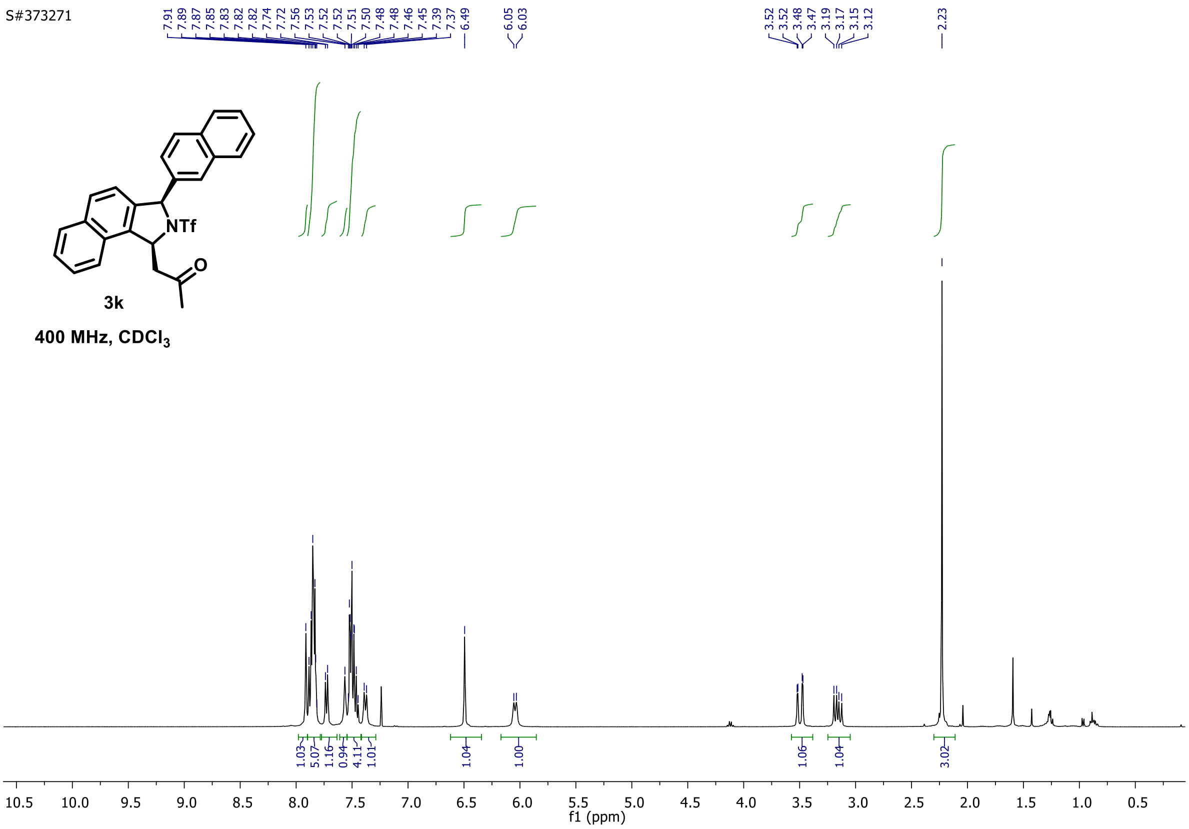
S#373271
7.91 7.89 7.87 7.85 7.83 7.82 7.82 7.74 7.72 7.56 7.53 7.52 7.52 7.51 7.50 7.48 7.48 7.46 7.45 7.39 7.37
6.49
6.05 6.03
3.52 3.52 3.48 3.47 3.19 3.17 3.15 3.12
2.23
NTf
O
3k
400 MHz, CDCl3
1.03 5.07 1.16 0.94 4.11 1.01
1.04
1.00
1.06
1.04
3.02
10.5 10.0 9.5 9.0 8.5 8.0 7.5 7.0 6.5 6.0 5.5 5.0 4.5 4.0 3.5 3.0 2.5 2.0 1.5 1.0 0.5
f1 (ppm)

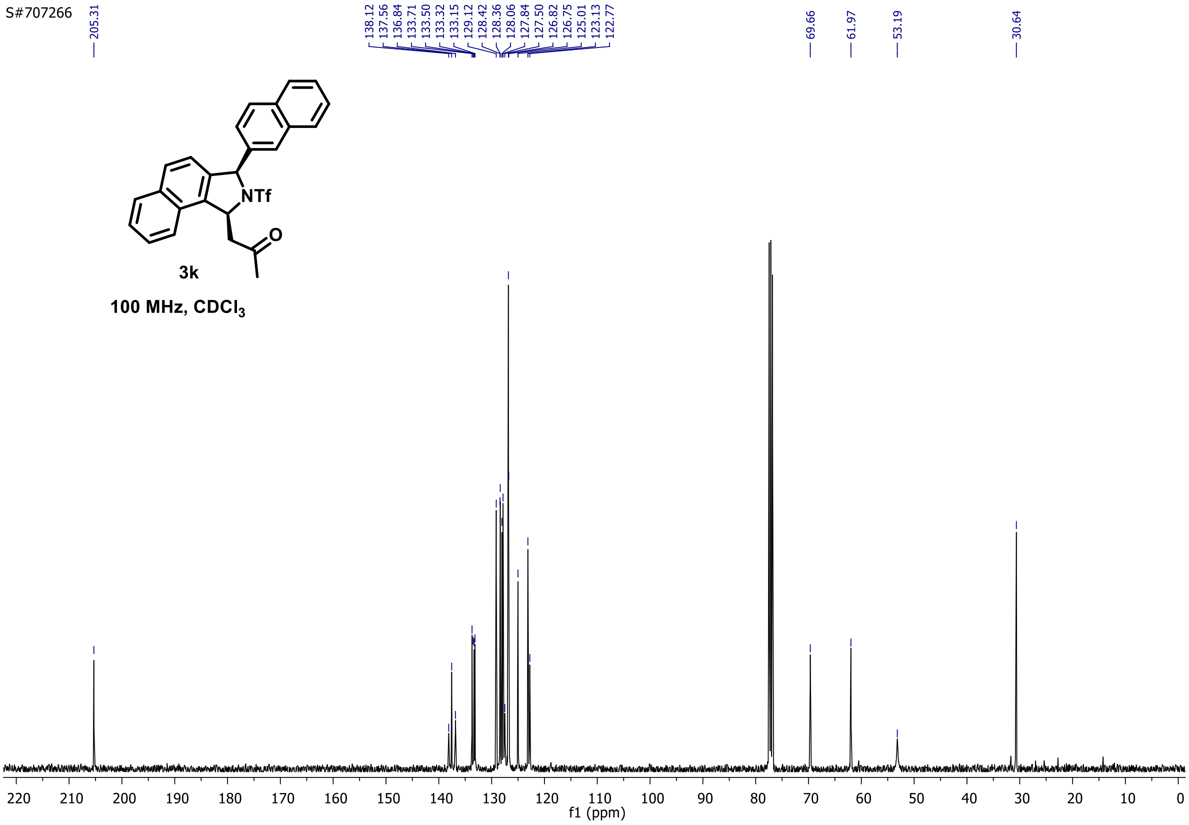
S#707266
205.31
138.12 137.56 136.84 133.71 133.50 133.32 133.15 129.12 128.42 128.36 128.06 127.84 127.50 126.82 126.75 125.01 123.13 122.77
69.66
61.97
53.19
30.64
NTf
O
3k
100 MHz, CDCl3
220 210 200 190 180 170 160 150 140 130 120 110 100 90 80 70 60 50 40 30 20 10 0
f1 (ppm)

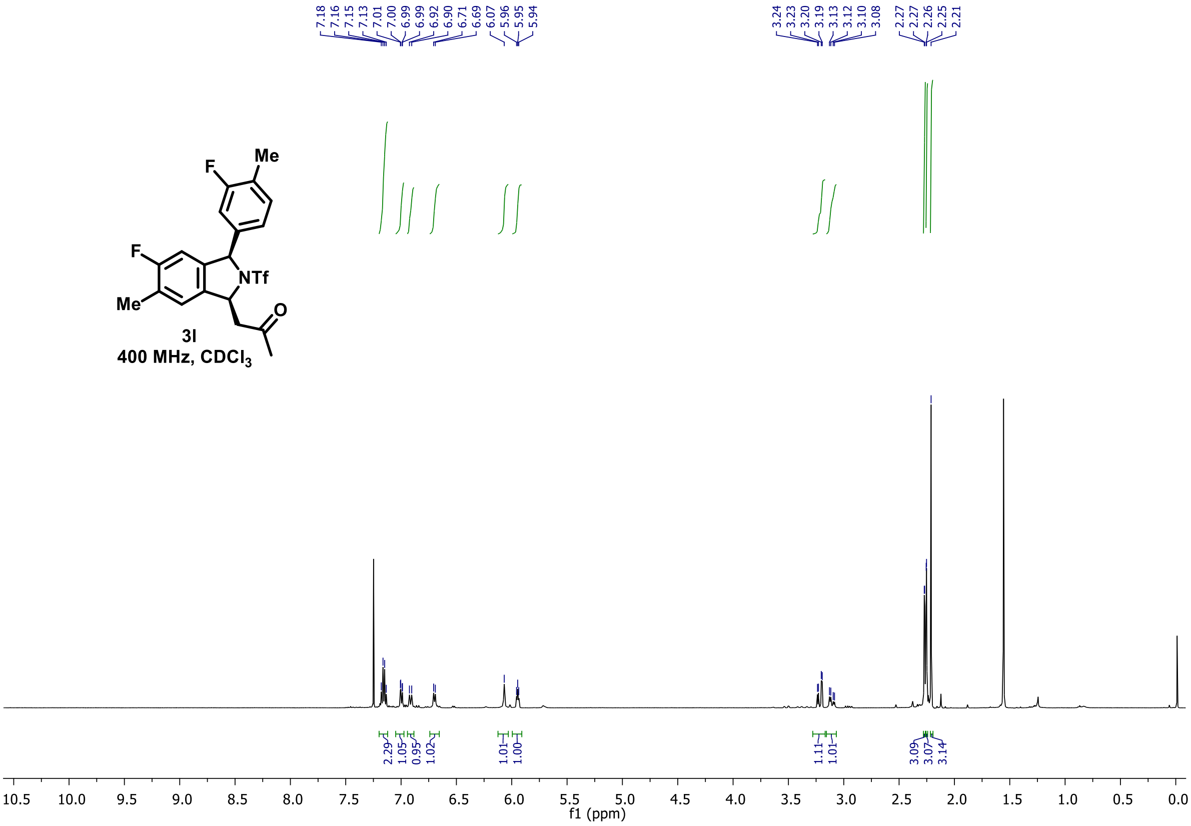
7.18
7.16
7.15
7.13
7.01
7.00
6.99
6.99
6.92
6.90
6.71
6.69
6.07
5.96
5.95
5.94
3.24
3.23
3.20
3.19
3.13
3.12
3.10
3.08
2.27
2.27
2.26
2.25
2.21
F
Me
F
NTf
Me
O
3l
400 MHz, CDCl3
2.29
1.05
0.95
1.02
1.01
1.00
1.11
1.01
3.09
3.07
3.14
f1 (ppm)

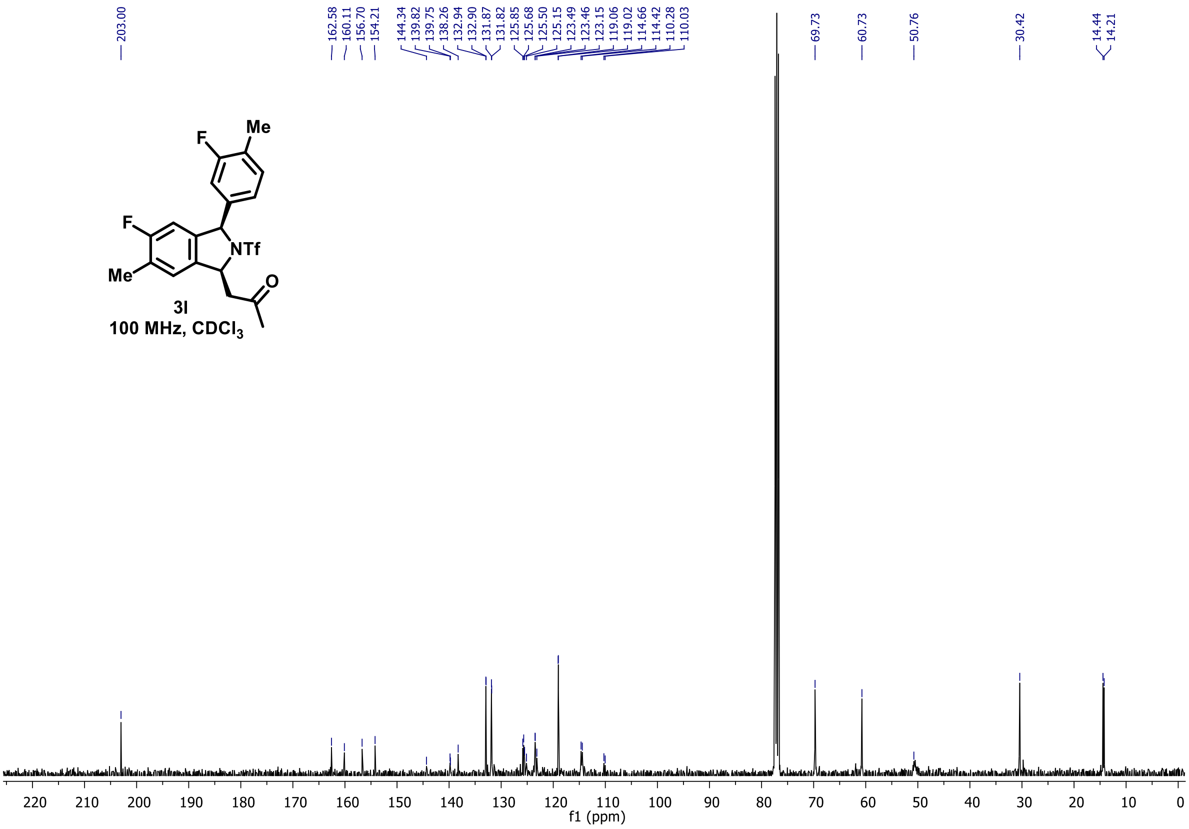
203.00
162.58
160.11
156.70
154.21
144.34
139.82
139.75
138.26
132.94
132.90
131.87
131.82
125.85
125.68
125.50
125.15
123.49
123.46
123.15
119.06
119.02
114.66
114.42
110.28
110.03
69.73
60.73
50.76
30.42
14.44
14.21
F
Me
F
NTf
Me
O
3l
100 MHz, CDCl3
f1 (ppm)

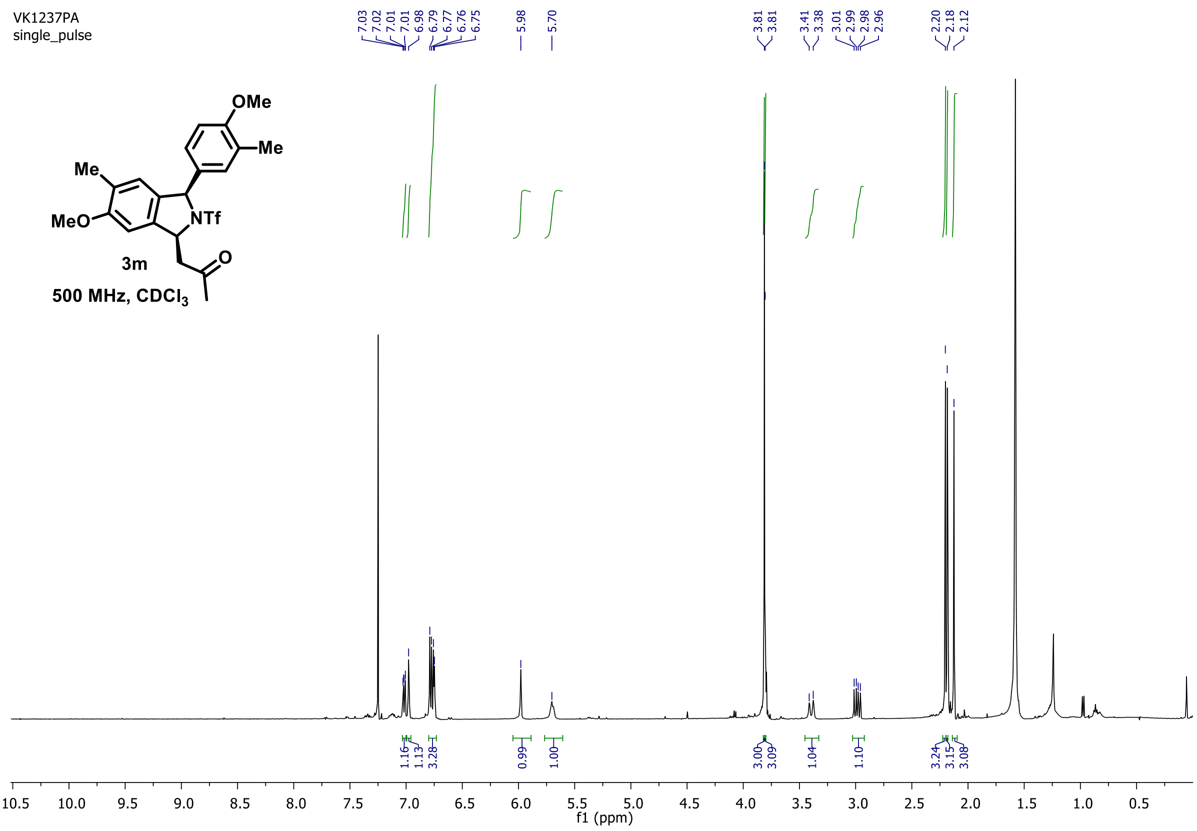
VK1237PA
single_pulse
7.03
7.02
7.01
6.98
6.79
6.77
6.76
6.75
5.98
5.70
3.81
3.81
3.41
3.38
3.01
2.99
2.98
2.96
2.20
2.18
2.12
OMe
Me
Me
MeO
NTf
O
3m
500 MHz, $CDCl_3$
1.16
1.13
3.28
0.99
1.00
3.00
3.09
1.04
1.10
3.24
3.15
3.08
10.5 10.0 9.5 9.0 8.5 8.0 7.5 7.0 6.5 6.0 5.5 5.0 4.5 4.0 3.5 3.0 2.5 2.0 1.5 1.0 0.5
f1 (ppm)

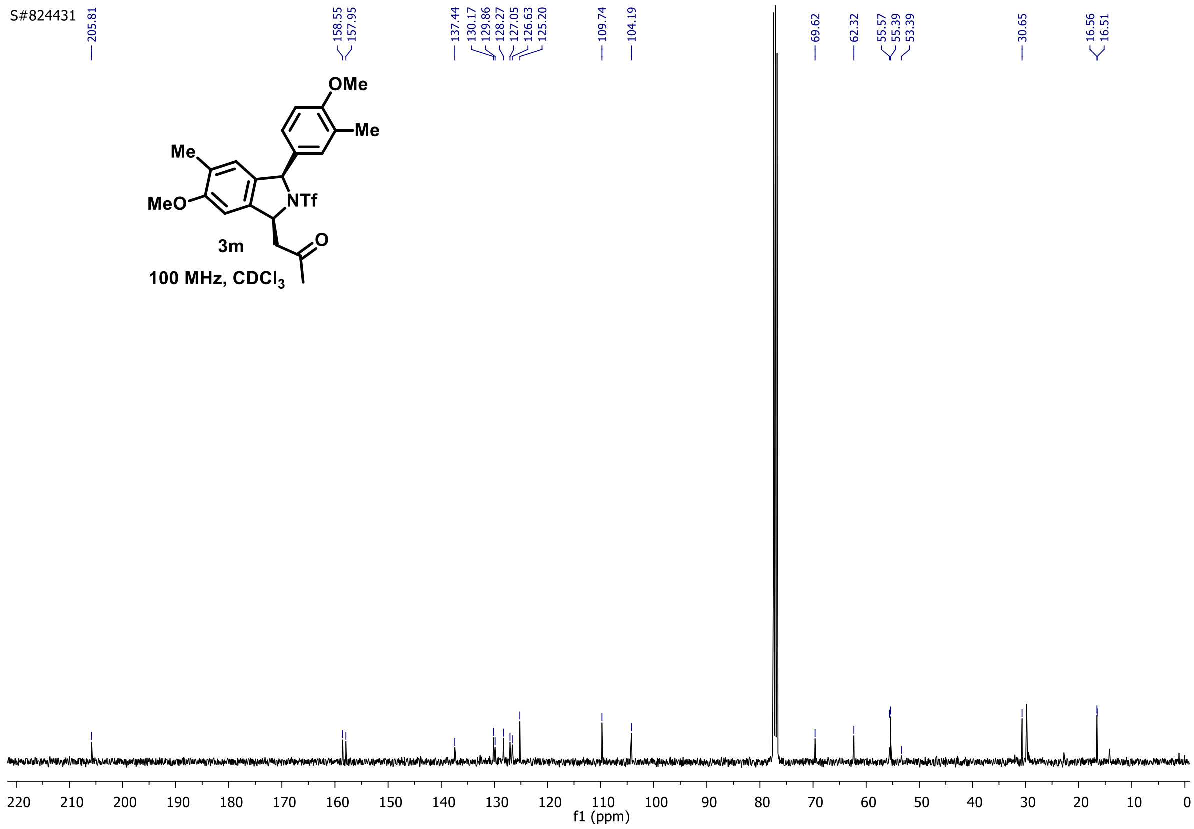
S#824431
205.81
158.55
157.95
137.44
130.17
129.86
128.27
127.05
126.63
125.20
109.74
104.19
69.62
62.32
55.57
55.39
53.39
30.65
16.56
16.51
OMe
Me
Me
MeO
NTf
O
3m
100 MHz, $CDCl_3$
220 210 200 190 180 170 160 150 140 130 120 110 100 90 80 70 60 50 40 30 20 10 0
f1 (ppm)

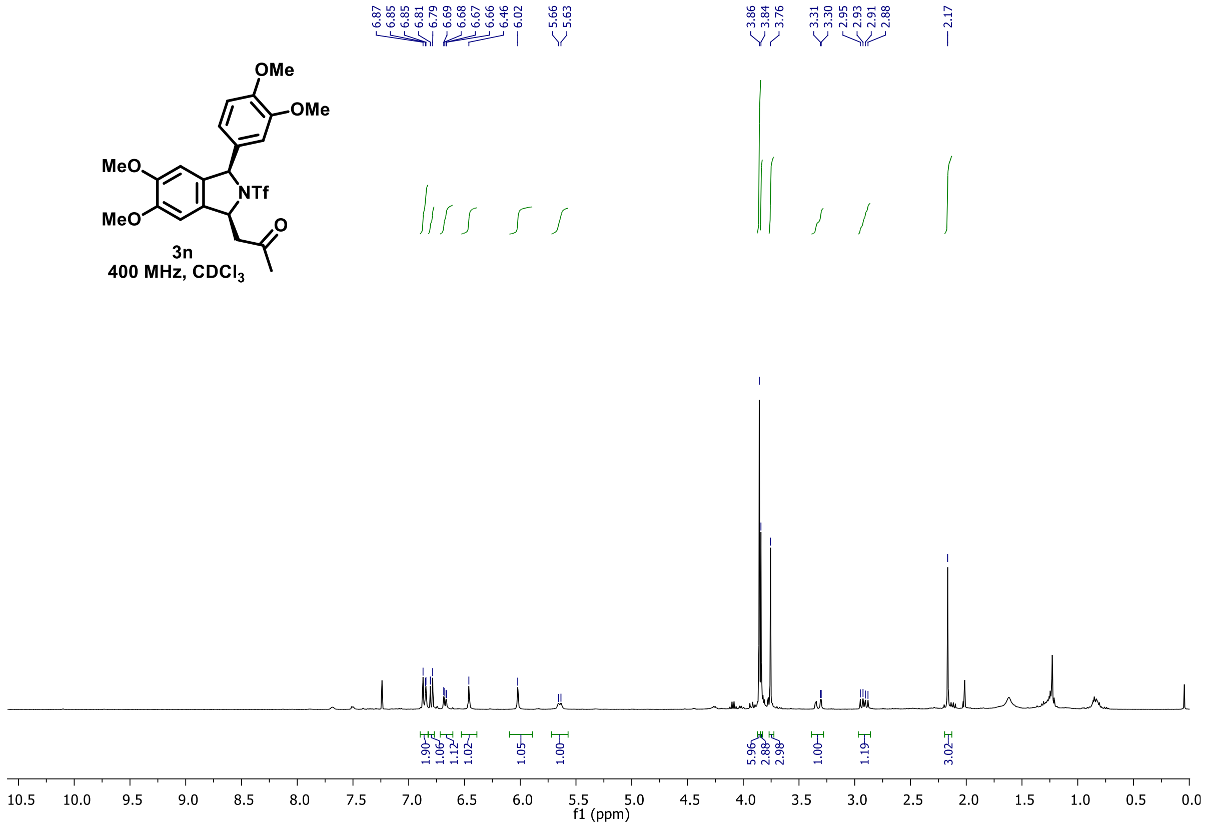
OMe
OMe
MeO
NTf
MeO
O
3n
400 MHz, CDCl3
6.87 6.85 6.85 6.81 6.79 6.69 6.68 6.67 6.66 6.46 6.02 5.66 5.63
3.86 3.84 3.76 3.31 3.30 2.95 2.93 2.91 2.88 2.17
1.90 1.06 1.12 1.02 1.05 1.00 5.96 2.88 2.98 1.00 1.19 3.02
10.5 10.0 9.5 9.0 8.5 8.0 7.5 7.0 6.5 6.0 5.5 5.0 4.5 4.0 3.5 3.0 2.5 2.0 1.5 1.0 0.5 0.0
f1 (ppm)

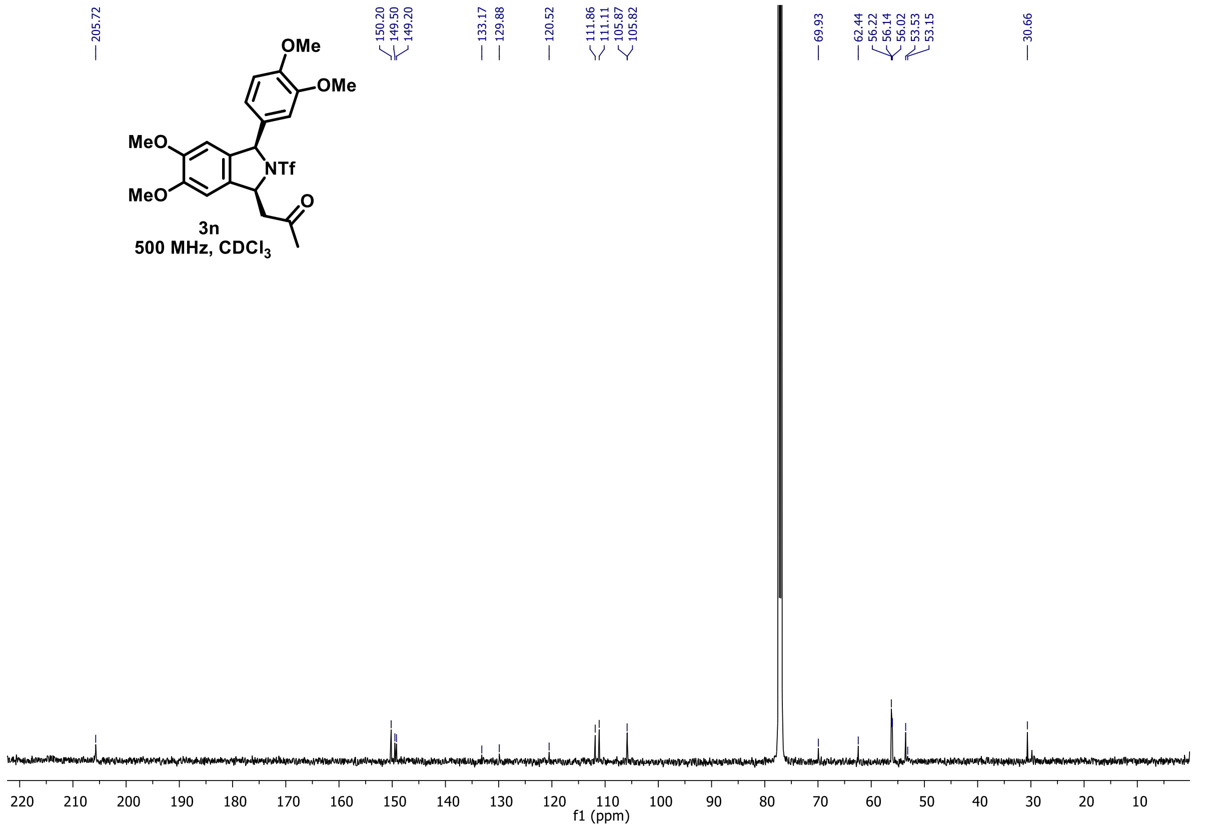
205.72
150.20 149.50 149.20
133.17 129.88
120.52
111.86 111.11 105.87 105.82
69.93
62.44 56.22 56.14 56.02 53.53 53.15
30.66
OMe
OMe
MeO
NTf
MeO
O
3n
500 MHz, CDCl3
220 210 200 190 180 170 160 150 140 130 120 110 100 90 80 70 60 50 40 30 20 10
f1 (ppm)

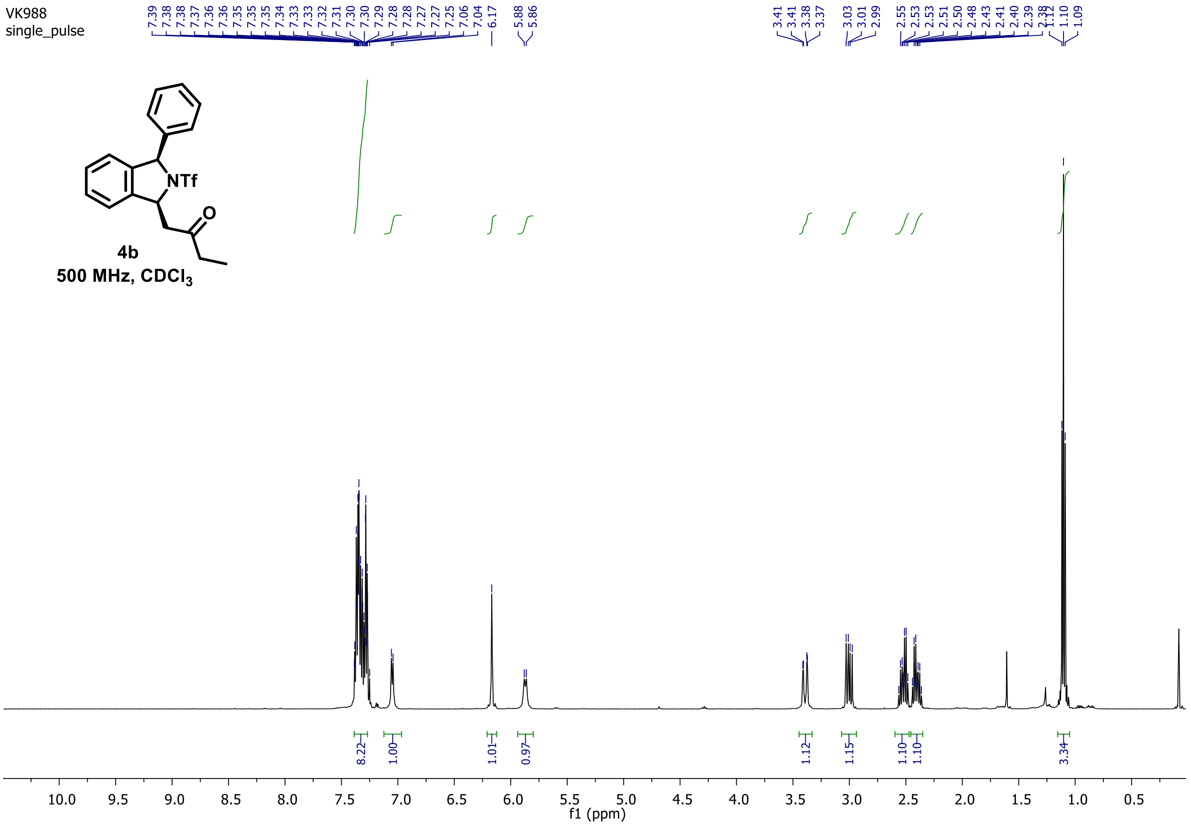
VK988
single_pulse
7.39 7.38 7.38 7.37 7.36 7.36 7.35 7.35 7.35 7.34 7.33 7.33 7.32 7.31 7.30 7.30 7.29 7.28 7.28 7.27 7.27 7.25 7.06 7.04 6.17 5.88 5.86
3.41 3.41 3.38 3.37 3.03 3.01 2.99 2.55 2.53 2.53 2.51 2.50 2.48 2.43 2.41 2.40 2.39 2.38 1.12 1.10 1.09
NTf
O
4b
500 MHz, $CDCl_3$
8.22 1.00 1.01 0.97 1.12 1.15 1.10 1.10 3.34
f1 (ppm)

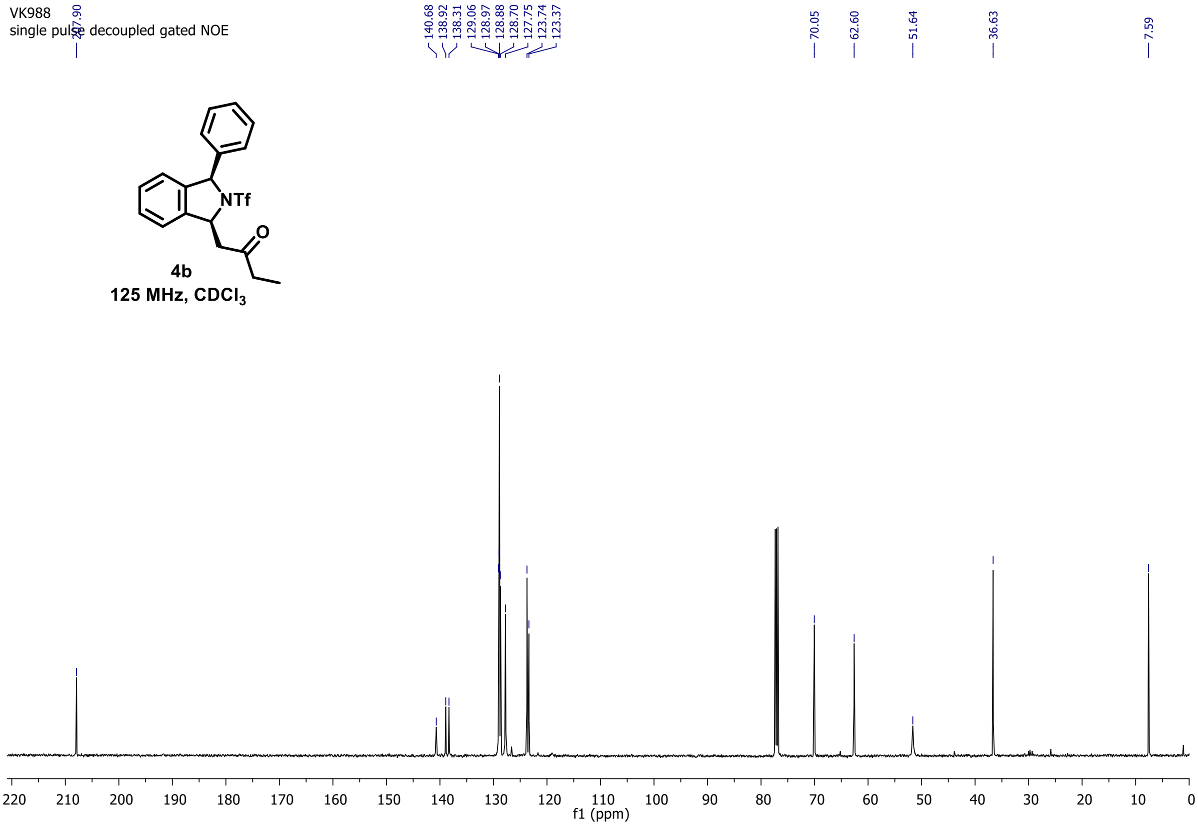
VK988
single pulse decoupled gated NOE
207.90
140.68 138.92 138.31 129.06 128.97 128.88 128.70 127.75 123.74 123.37
70.05 62.60 51.64 36.63 7.59
NTf
O
4b
125 MHz, $CDCl_3$
f1 (ppm)

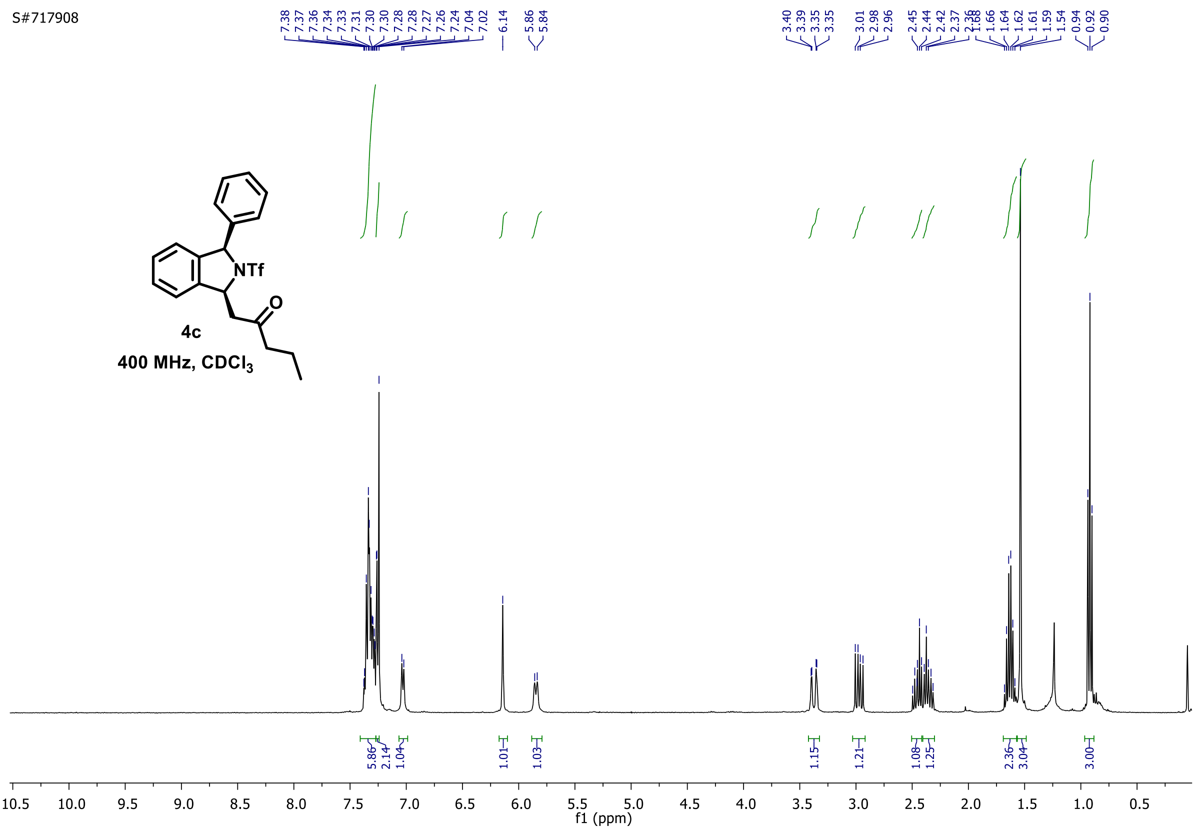
S#717908
4c
400 MHz, $CDCl_3$
f1 (ppm)

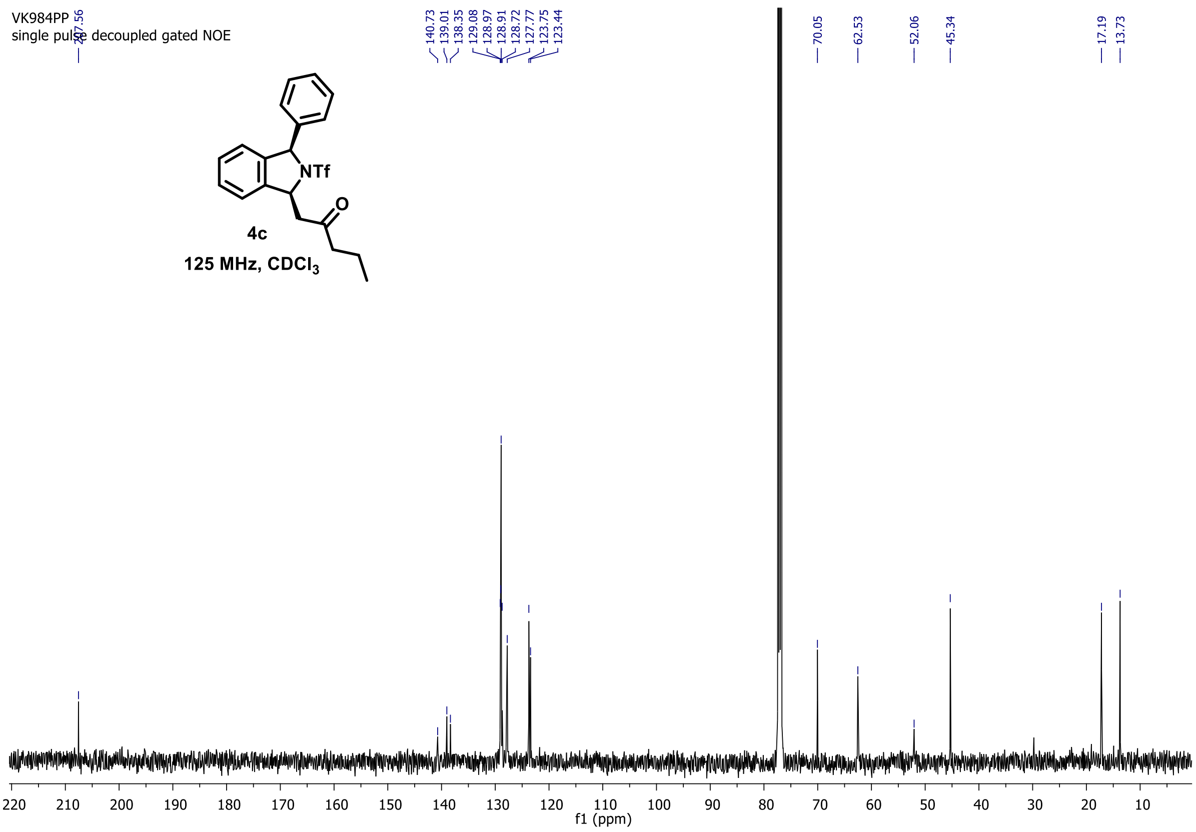
VK984PP
single pulse decoupled gated NOE
4c
125 MHz, $CDCl_3$
f1 (ppm)

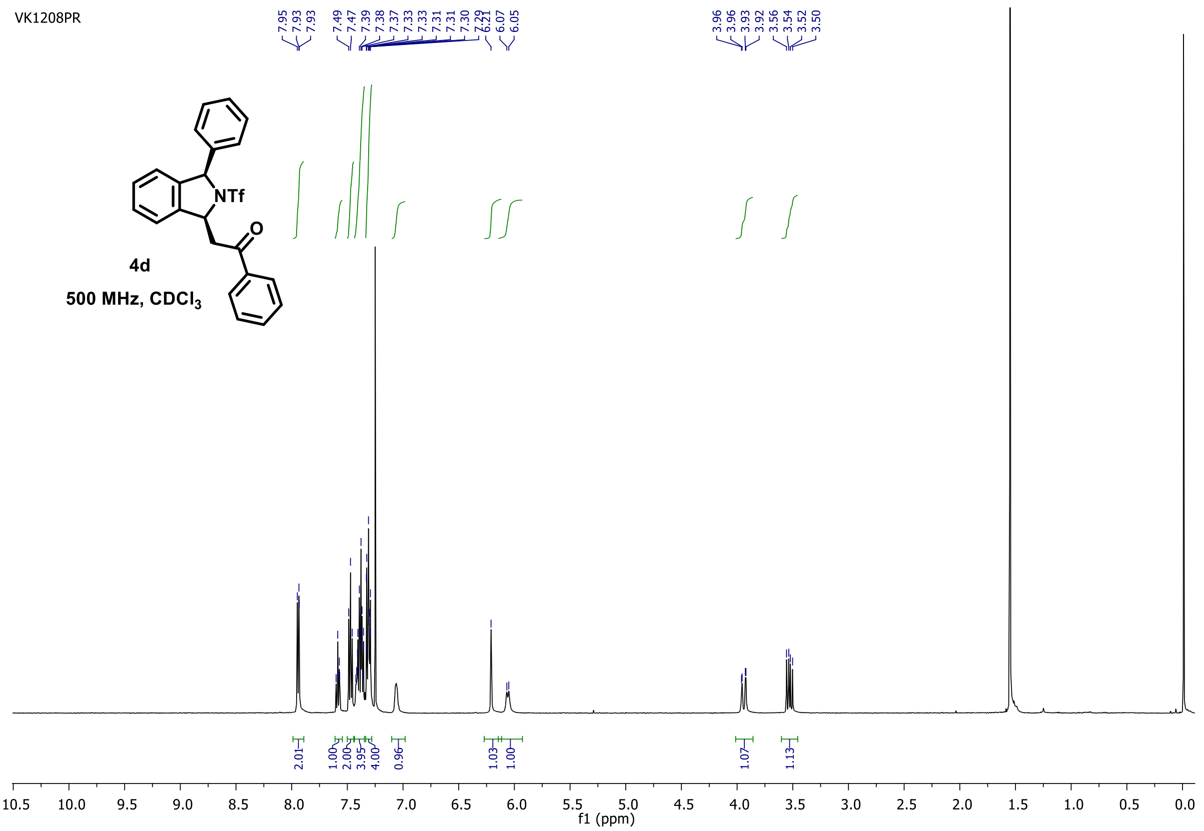
VK1208PR
4d
500 MHz, CDCl3
NTf
O
7.95
7.93
7.93
7.49
7.47
7.39
7.38
7.37
7.33
7.33
7.31
7.31
7.30
7.29
6.21
6.07
6.05
3.96
3.96
3.93
3.92
3.56
3.54
3.52
3.50
2.01
1.00
2.00
3.95
4.00
0.96
1.03
1.00
1.07
1.13
f1 (ppm)

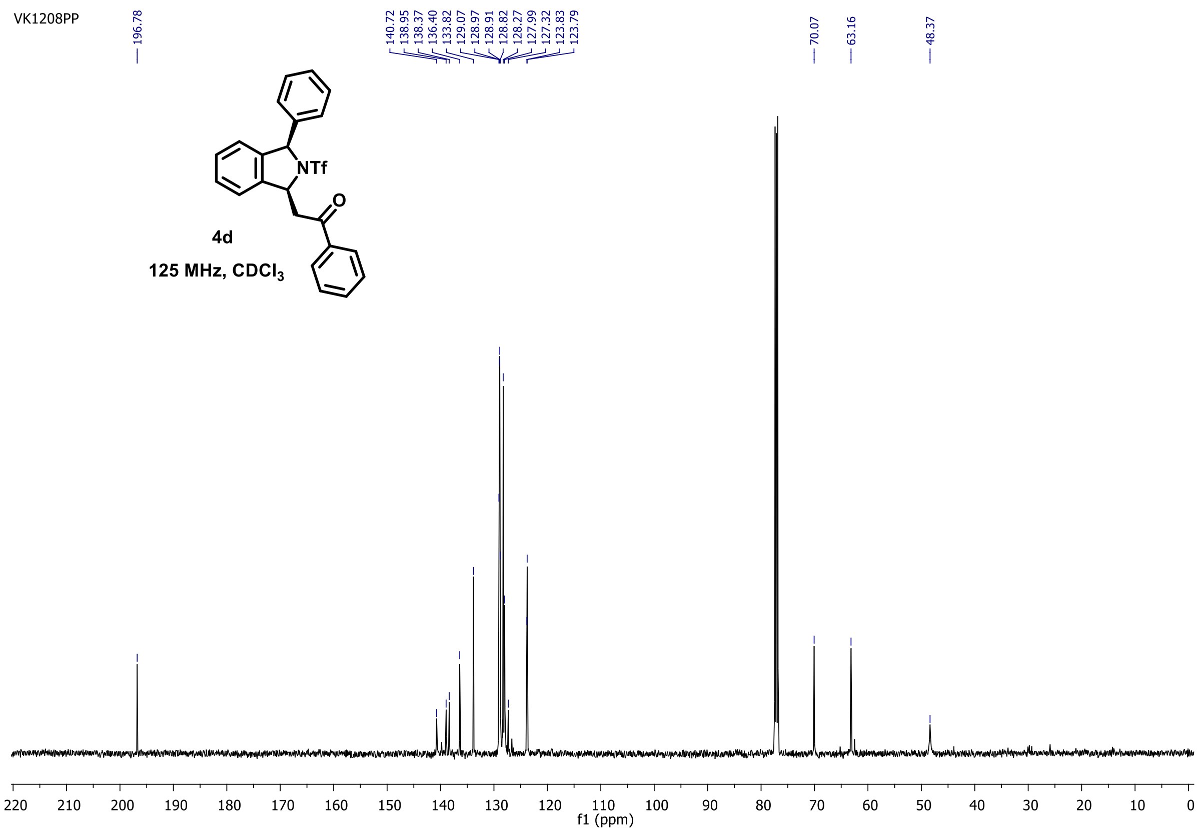
VK1208PP
4d
125 MHz, CDCl3
NTf
O
196.78
140.72
138.95
138.37
136.40
133.82
129.07
128.97
128.91
128.82
128.27
127.99
127.32
123.83
123.79
70.07
63.16
48.37
f1 (ppm)

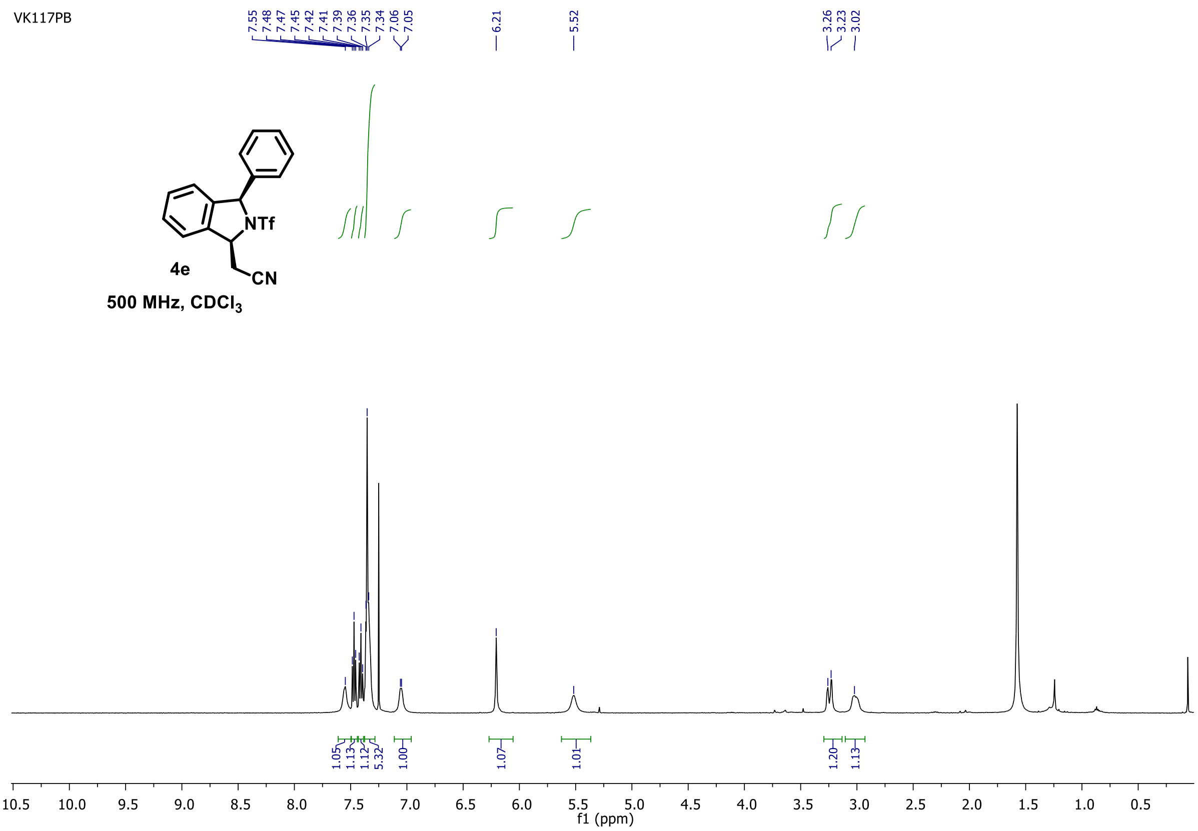
VK117PB
7.55
7.48
7.47
7.45
7.42
7.41
7.39
7.36
7.35
7.34
7.06
7.05
6.21
5.52
3.26
3.23
3.02
NTf
4e
CN
500 MHz, CDCl3
1.05
1.13
1.12
5.32
1.00
1.07
1.01
1.20
1.13
10.5 10.0 9.5 9.0 8.5 8.0 7.5 7.0 6.5 6.0 5.5 5.0 4.5 4.0 3.5 3.0 2.5 2.0 1.5 1.0 0.5
f1 (ppm)

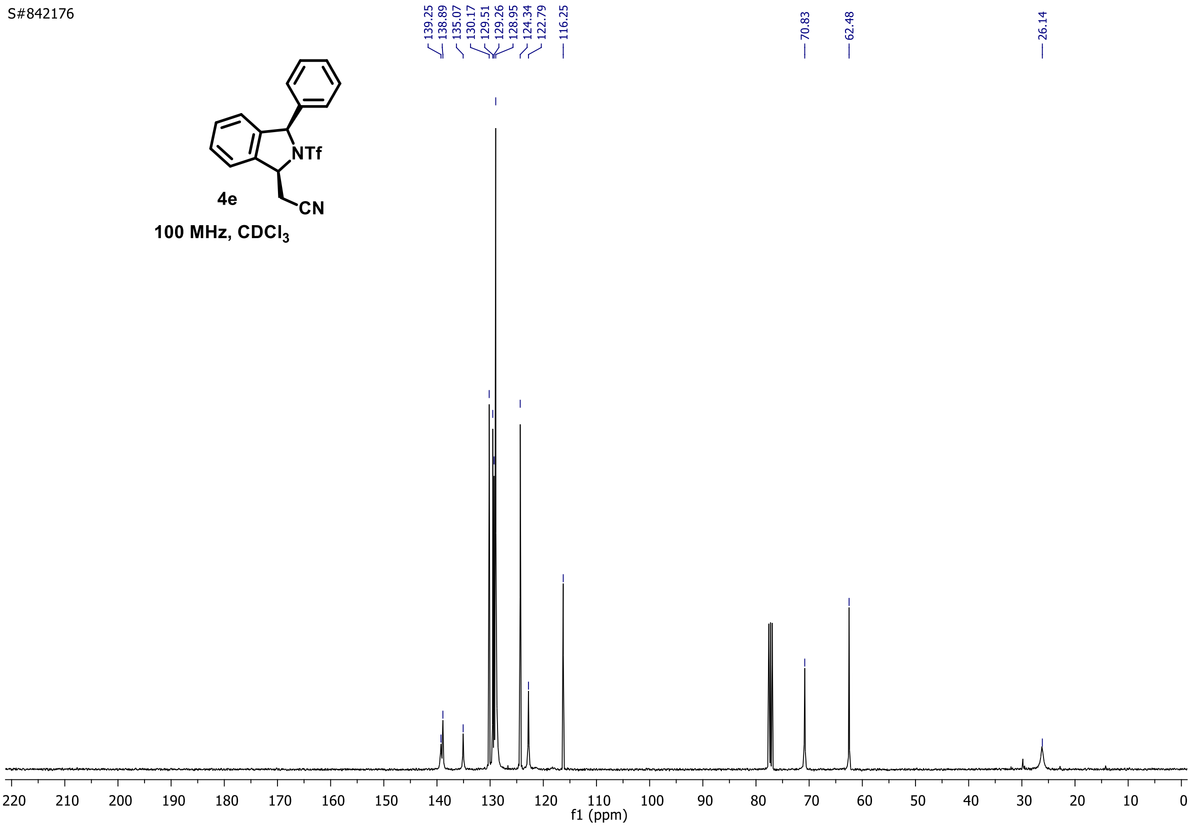
S#842176
139.25
138.89
135.07
130.17
129.51
129.26
128.95
124.34
122.79
116.25
70.83
62.48
26.14
NTf
4e
CN
100 MHz, CDCl3
220 210 200 190 180 170 160 150 140 130 120 110 100 90 80 70 60 50 40 30 20 10 0
f1 (ppm)

S#535337

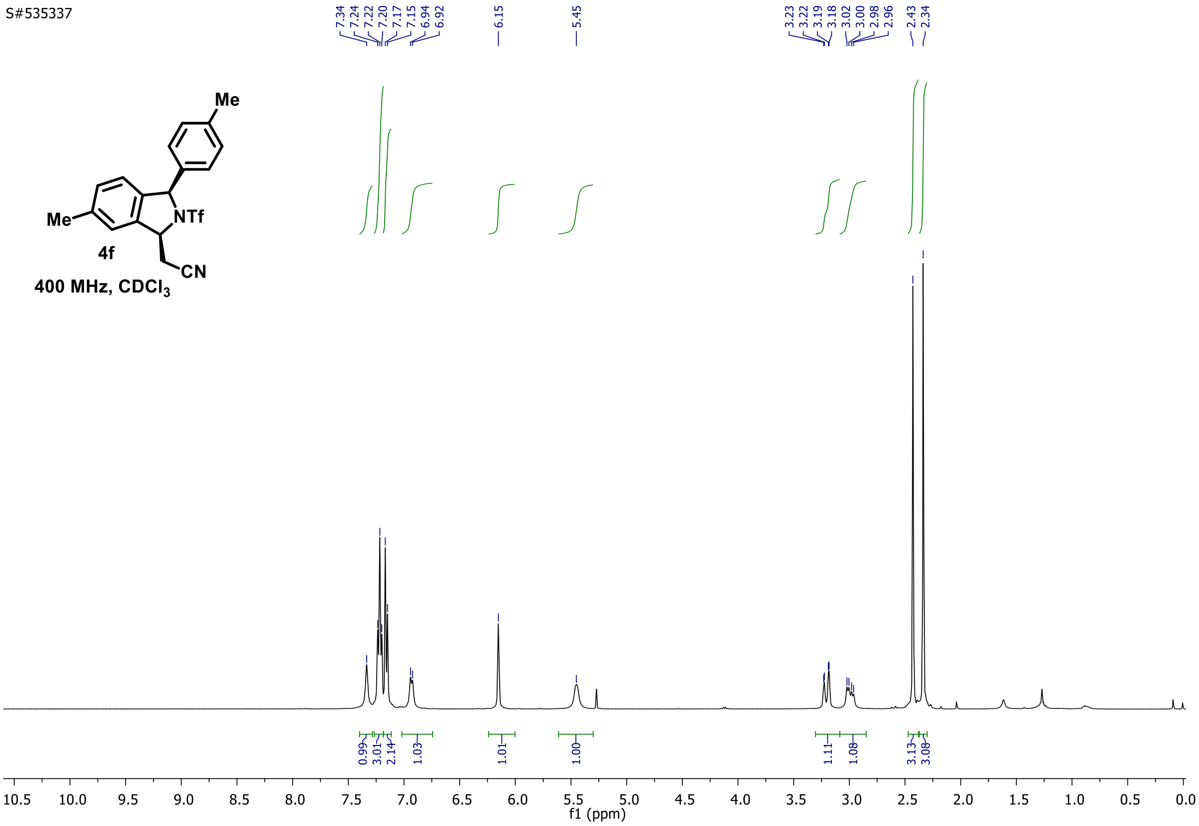

4f

400 MHz, $CDCl_3$

VK1455

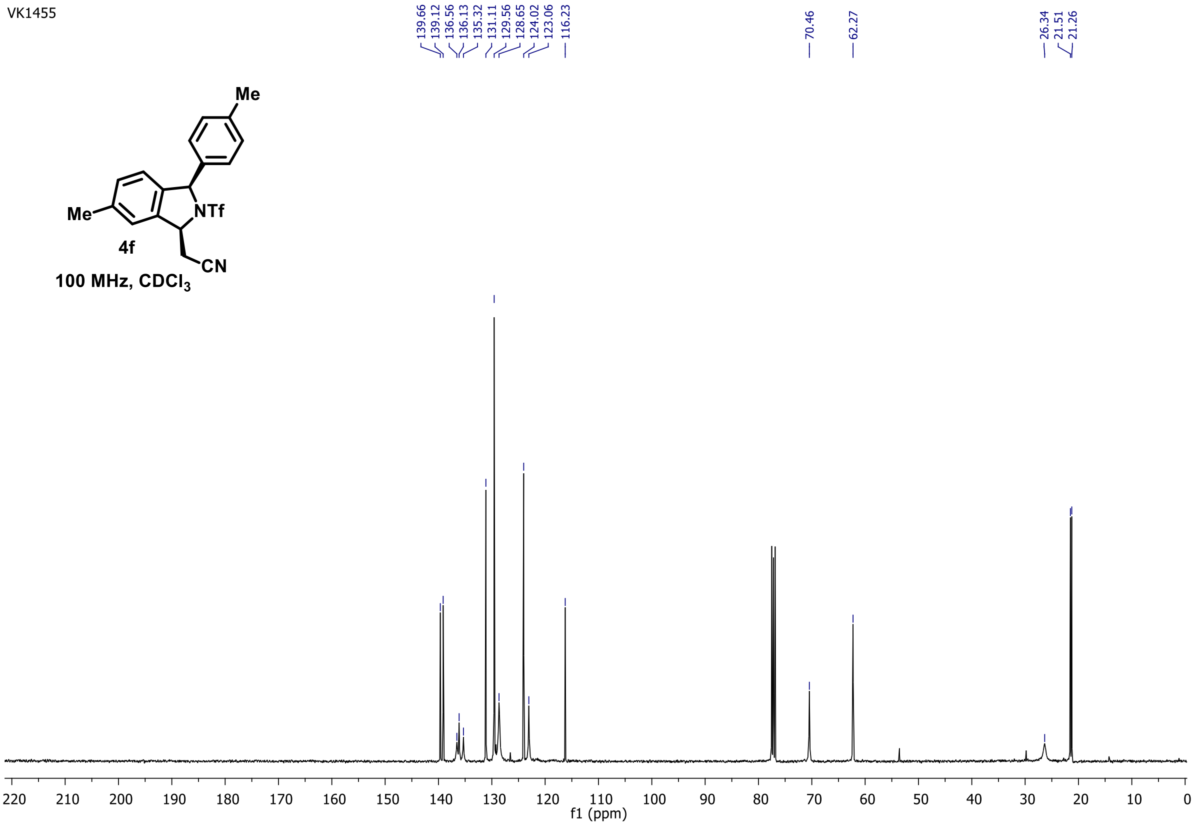

4f

100 MHz, $CDCl_3$

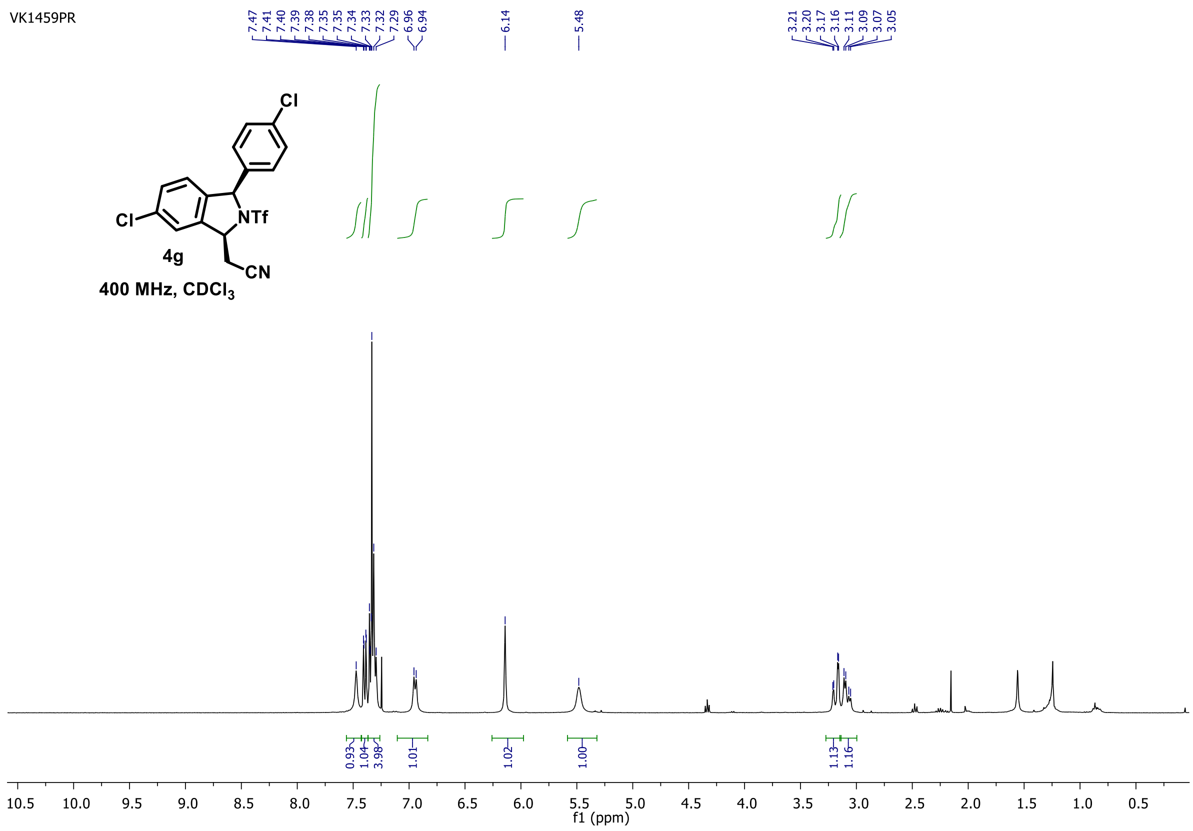
VK1459PR
7.47 7.41 7.40 7.39 7.38 7.35 7.35 7.34 7.33 7.32 7.29 6.96 6.94
6.14
5.48
3.21 3.20 3.17 3.16 3.11 3.09 3.07 3.05
Cl
Cl
NTf
4g
CN
400 MHz, $CDCl_3$
0.93 1.04 3.98 1.01 1.02 1.00 1.13 1.16
10.5 10.0 9.5 9.0 8.5 8.0 7.5 7.0 6.5 6.0 5.5 5.0 4.5 4.0 3.5 3.0 2.5 2.0 1.5 1.0 0.5
f1 (ppm)

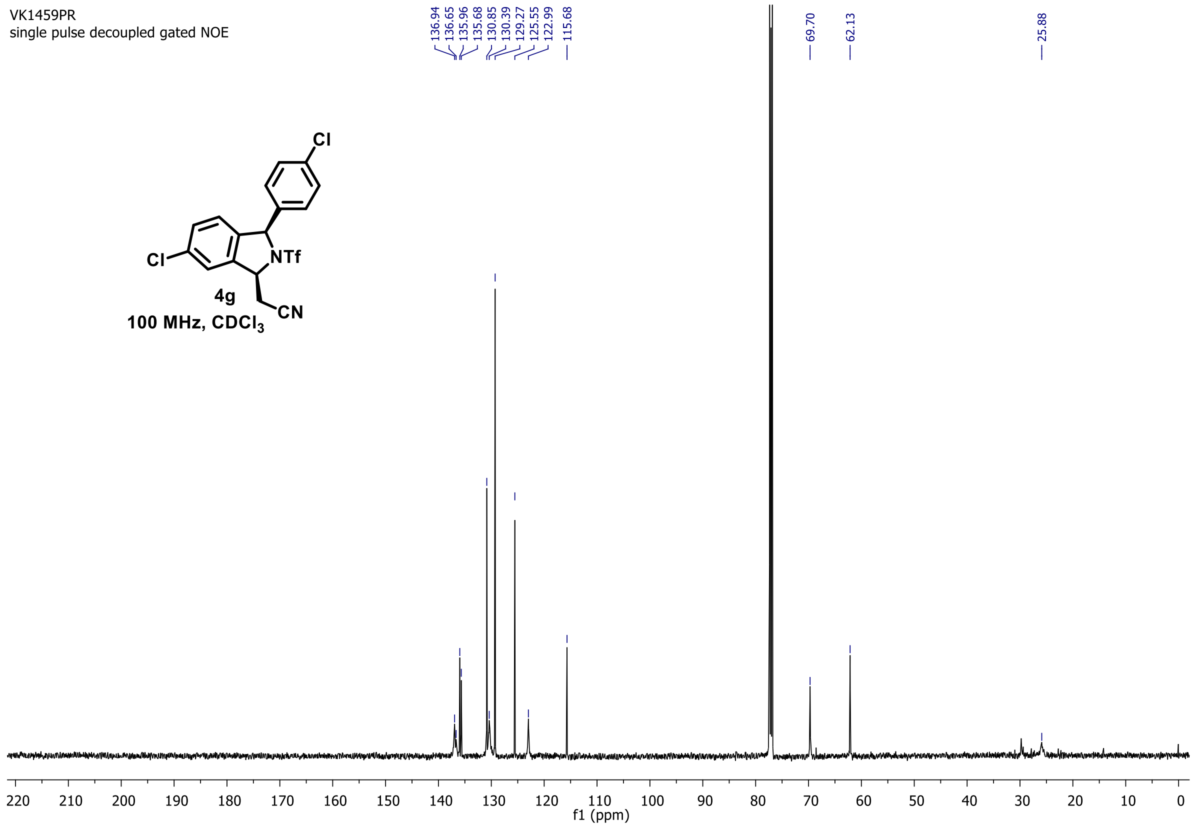
VK1459PR
single pulse decoupled gated NOE
136.94 136.65 135.96 135.68 130.85 130.39 129.27 125.55 122.99 115.68
69.70
62.13
25.88
Cl
Cl
NTf
4g
CN
100 MHz, $CDCl_3$
220 210 200 190 180 170 160 150 140 130 120 110 100 90 80 70 60 50 40 30 20 10 0
f1 (ppm)

S#695641

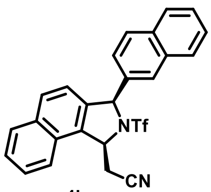

4h

400 MHz, $CDCl_3$

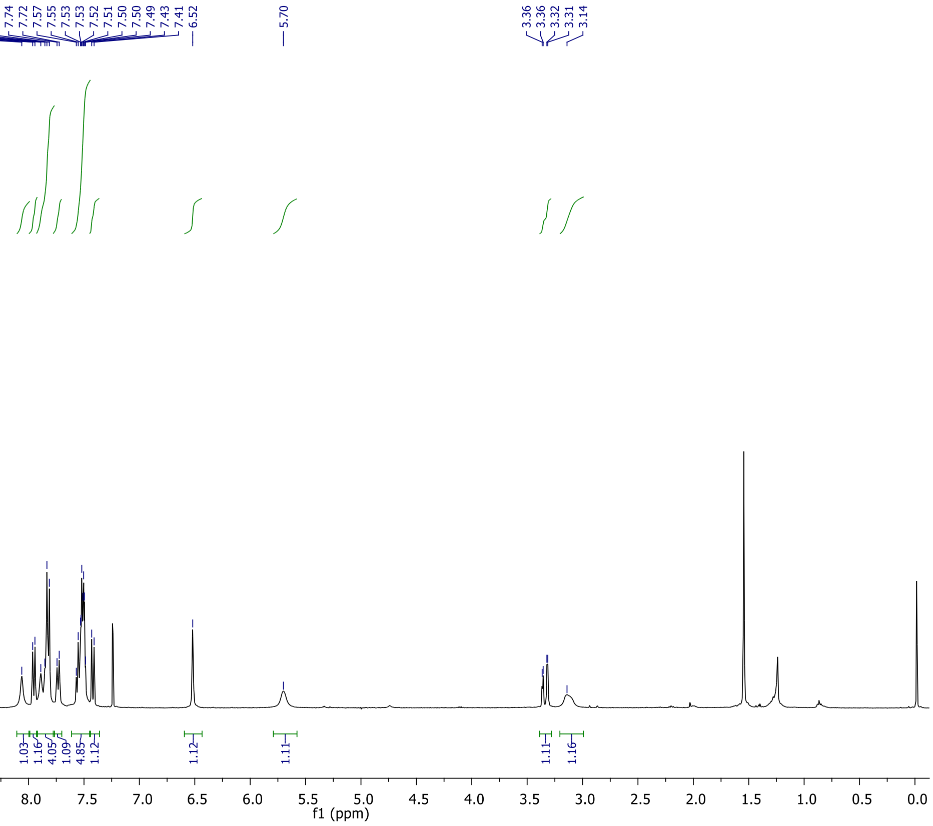

S#12026

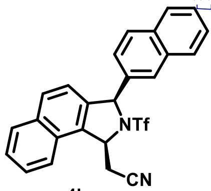

4h

100 MHz, $CDCl_3$

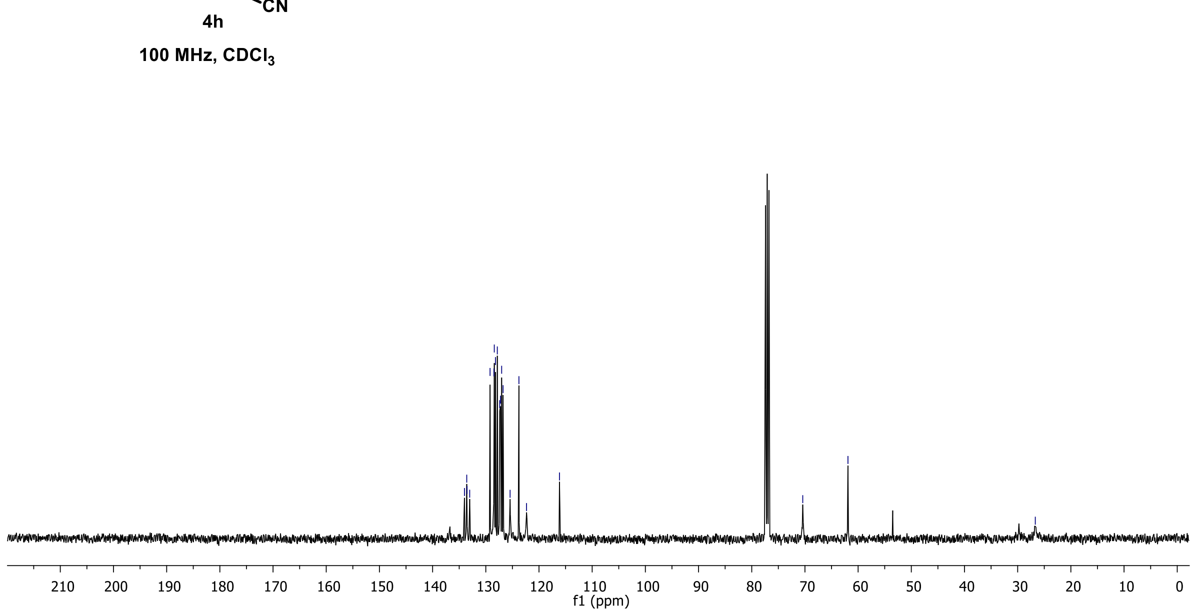

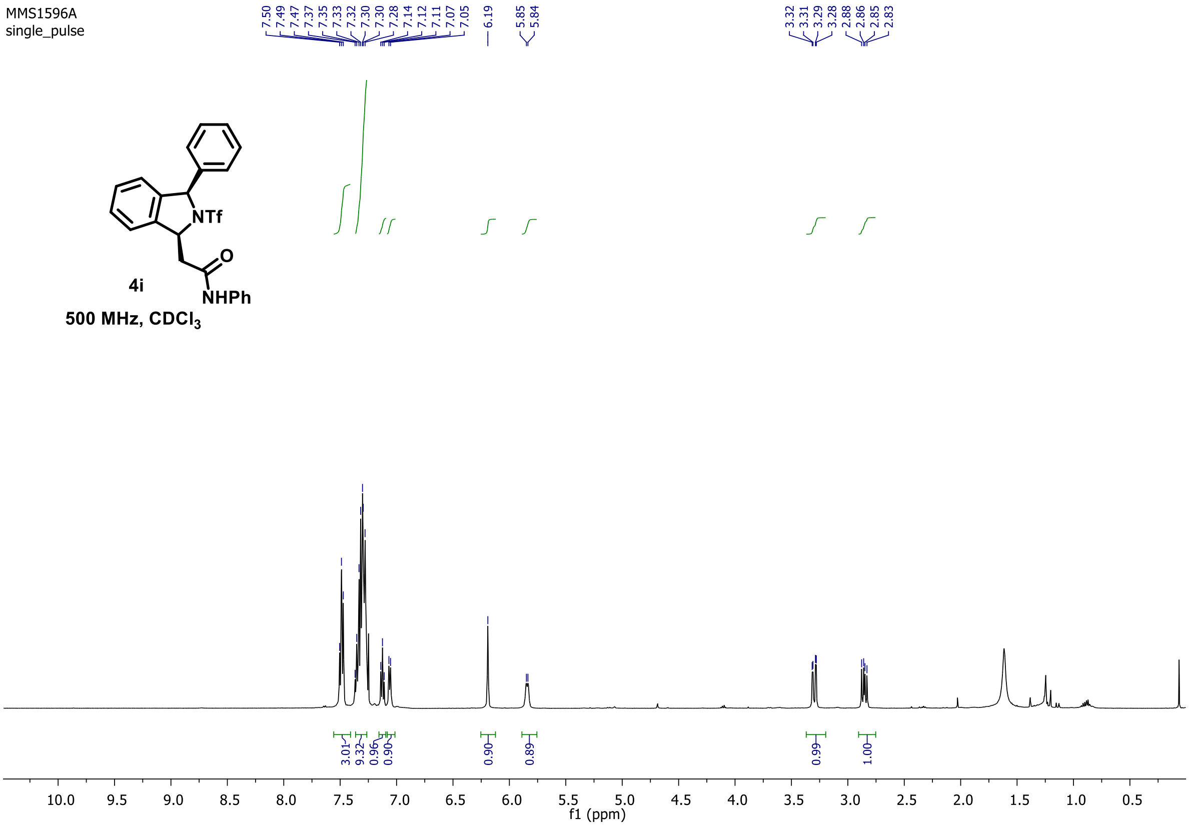
MMS1596A
single_pulse
7.50
7.49
7.47
7.37
7.35
7.33
7.32
7.30
7.30
7.28
7.14
7.12
7.11
7.07
7.05
6.19
5.85
5.84
3.32
3.31
3.29
3.28
2.88
2.86
2.85
2.83
NTf
O
NHPh
4i
500 MHz, CDCl3
3.01
9.32
0.96
0.90
0.90
0.89
0.99
1.00
10.0 9.5 9.0 8.5 8.0 7.5 7.0 6.5 6.0 5.5 5.0 4.5 4.0 3.5 3.0 2.5 2.0 1.5 1.0 0.5
f1 (ppm)

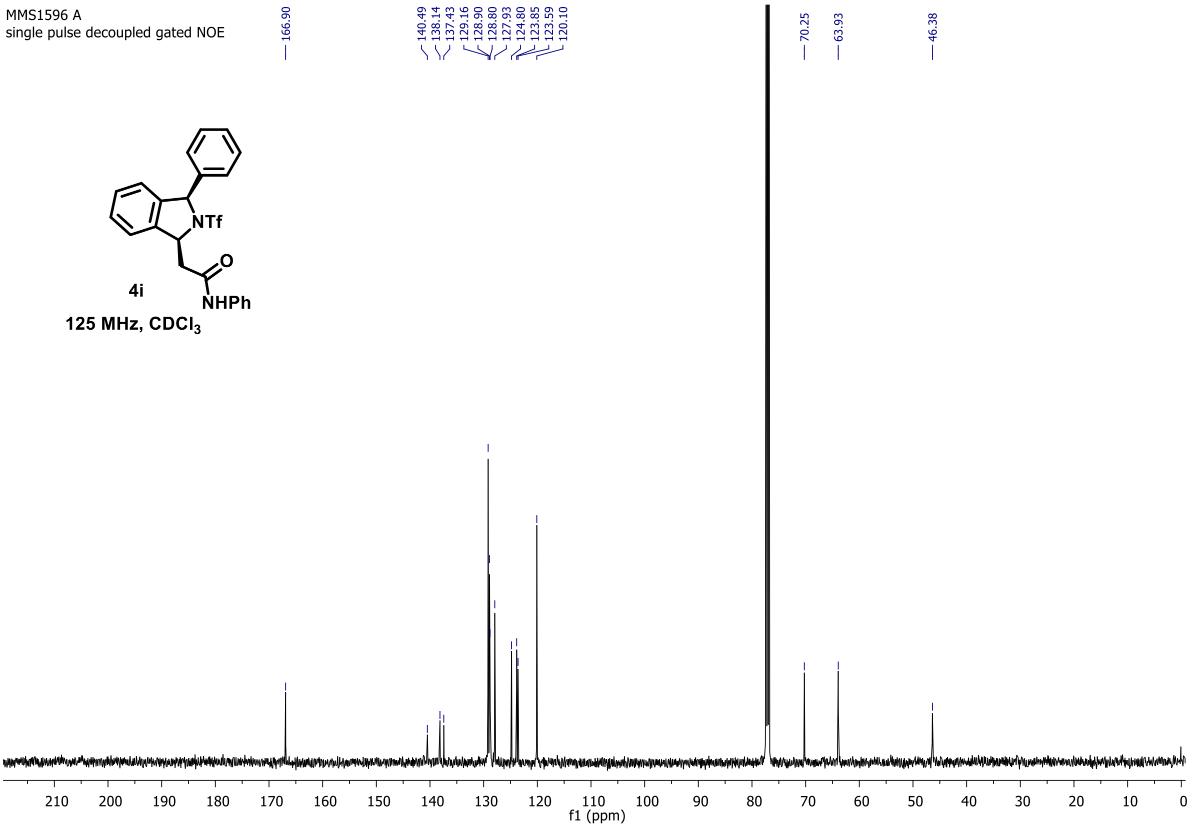
MMS1596 A
single pulse decoupled gated NOE
166.90
140.49
138.14
137.43
129.16
128.90
128.80
127.93
124.80
123.85
123.59
120.10
70.25
63.93
46.38
NTf
O
NHPh
4i
125 MHz, CDCl3
210 200 190 180 170 160 150 140 130 120 110 100 90 80 70 60 50 40 30 20 10 0
f1 (ppm)

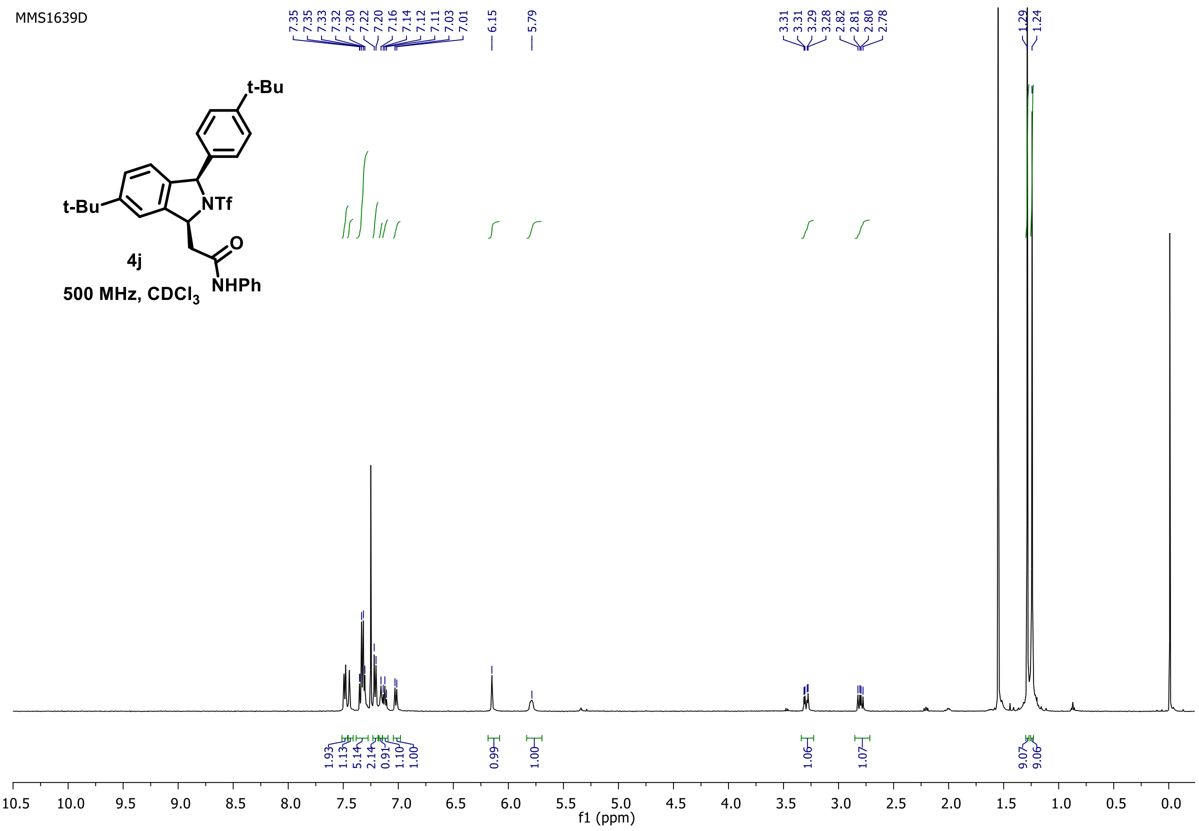
MMS1639D
t-Bu
t-Bu
NTf
O
4j
NHPh
500 MHz, CDCl3
7.35
7.35
7.33
7.32
7.30
7.22
7.20
7.16
7.14
7.12
7.11
7.03
7.01
6.15
5.79
3.31
3.31
3.29
3.28
2.82
2.81
2.80
2.78
1.29
1.24
1.93
1.13
5.14
2.14
0.91
1.10
1.00
0.99
1.00
1.06
1.07
9.07
9.06
10.5 10.0 9.5 9.0 8.5 8.0 7.5 7.0 6.5 6.0 5.5 5.0 4.5 4.0 3.5 3.0 2.5 2.0 1.5 1.0 0.5 0.0
f1 (ppm)

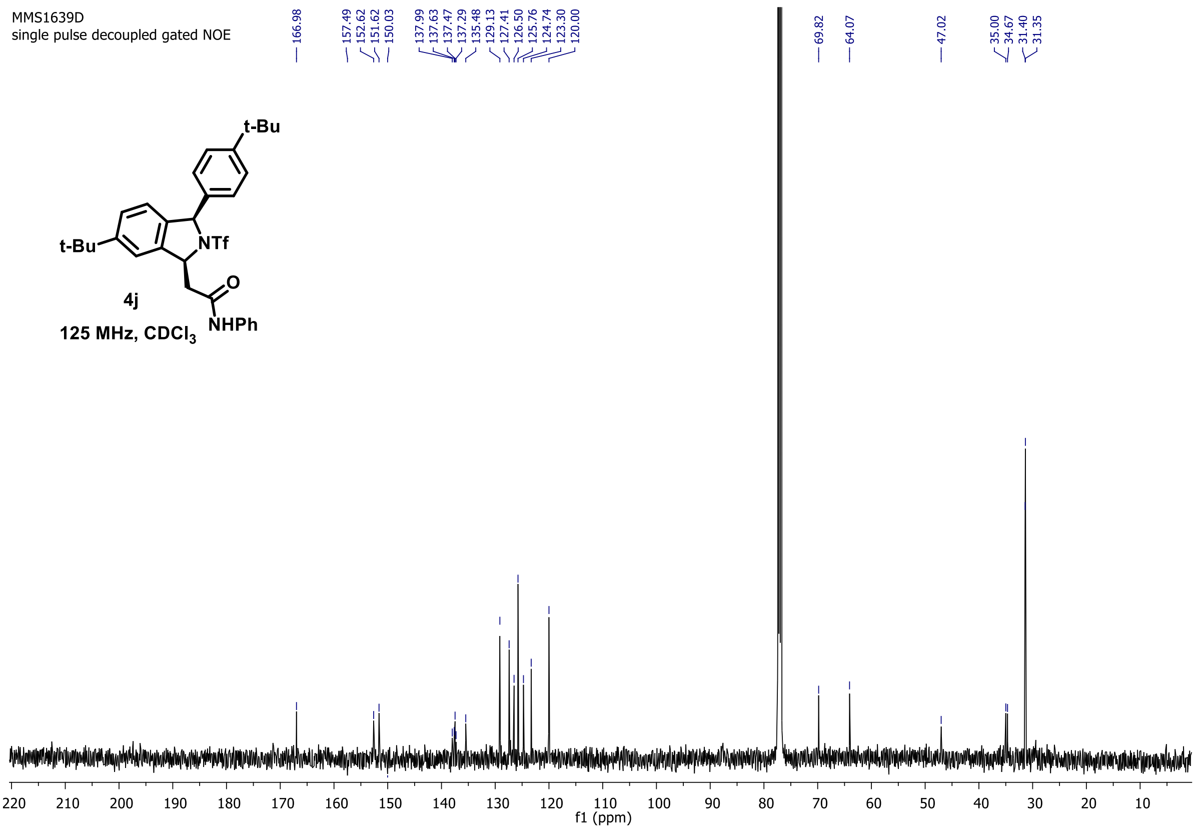
MMS1639D
single pulse decoupled gated NOE
t-Bu
t-Bu
NTf
O
4j
NHPh
125 MHz, CDCl3
166.98
157.49
152.62
151.62
150.03
137.99
137.63
137.47
137.29
135.48
129.13
127.41
126.50
125.76
124.74
123.30
120.00
69.82
64.07
47.02
35.00
34.67
31.40
31.35
220 210 200 190 180 170 160 150 140 130 120 110 100 90 80 70 60 50 40 30 20 10
f1 (ppm)

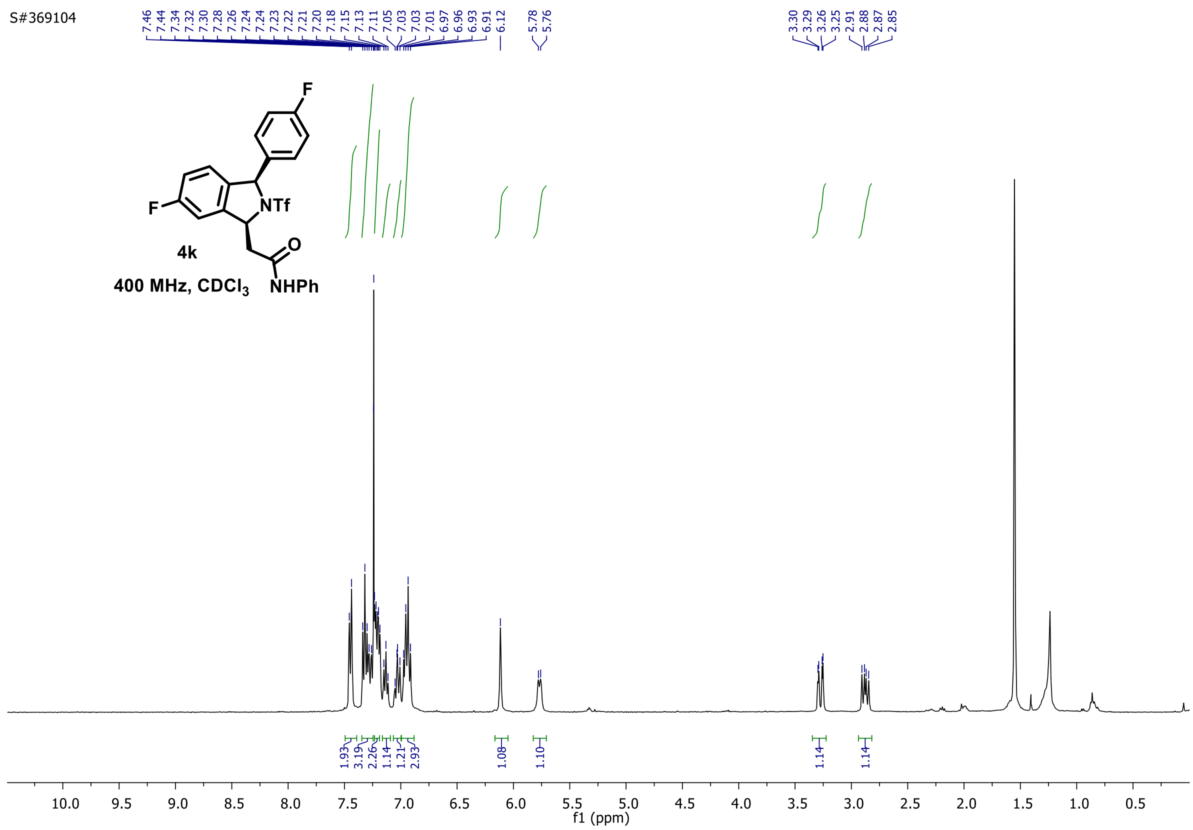
S#369104
7.46 7.44 7.34 7.32 7.30 7.28 7.26 7.24 7.24 7.23 7.22 7.21 7.20 7.18 7.15 7.13 7.11 7.05 7.03 7.03 7.01 6.97 6.96 6.93 6.91 6.12 5.78 5.76
3.30 3.29 3.26 3.25 2.91 2.88 2.87 2.85
F
F
NTf
O
4k
400 MHz, CDCl3
NHPh
1.93 3.19 2.26 1.14 1.21 2.93 1.08 1.10 1.14 1.14
f1 (ppm)

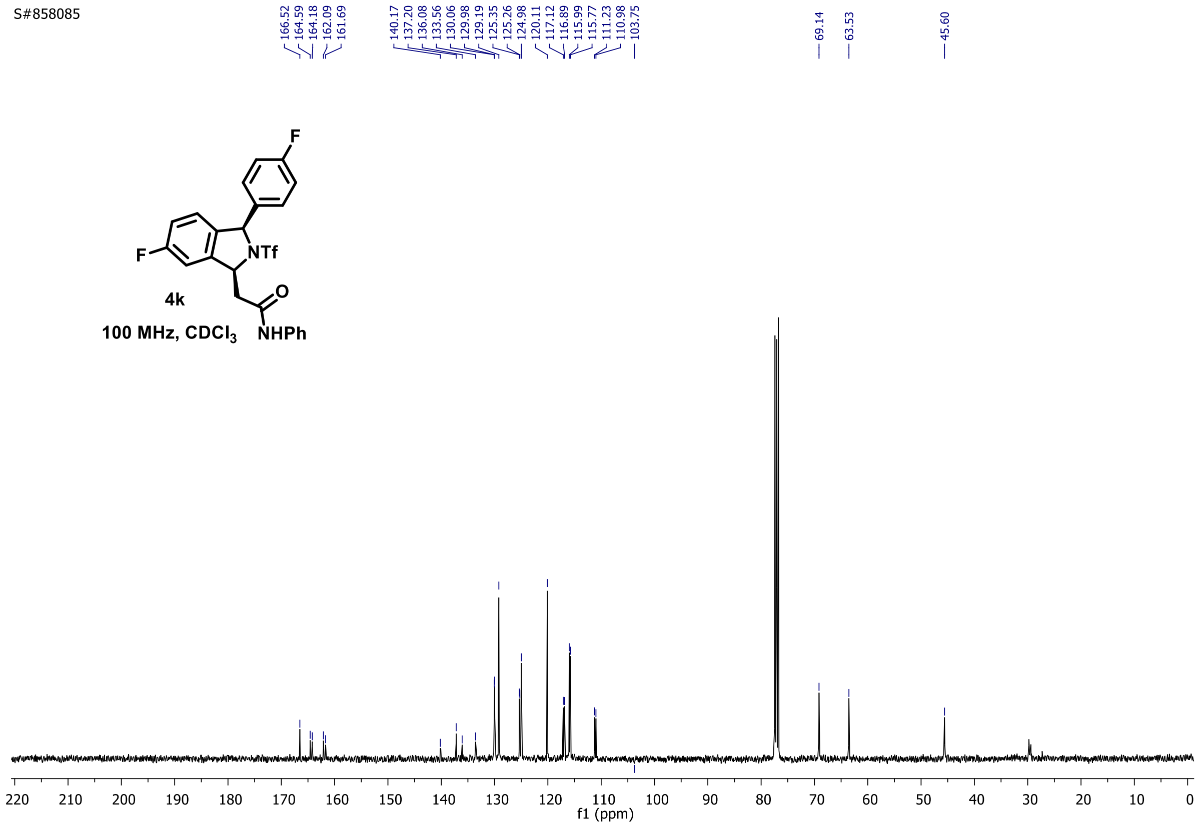
S#858085
166.52 164.59 164.18 162.09 161.69
140.17 137.20 136.08 133.56 130.06 129.98 129.19 125.35 125.26 124.98 120.11 117.12 116.89 115.99 115.77 111.23 110.98 103.75
69.14 63.53 45.60
F
F
NTf
O
4k
100 MHz, CDCl3
NHPh
f1 (ppm)

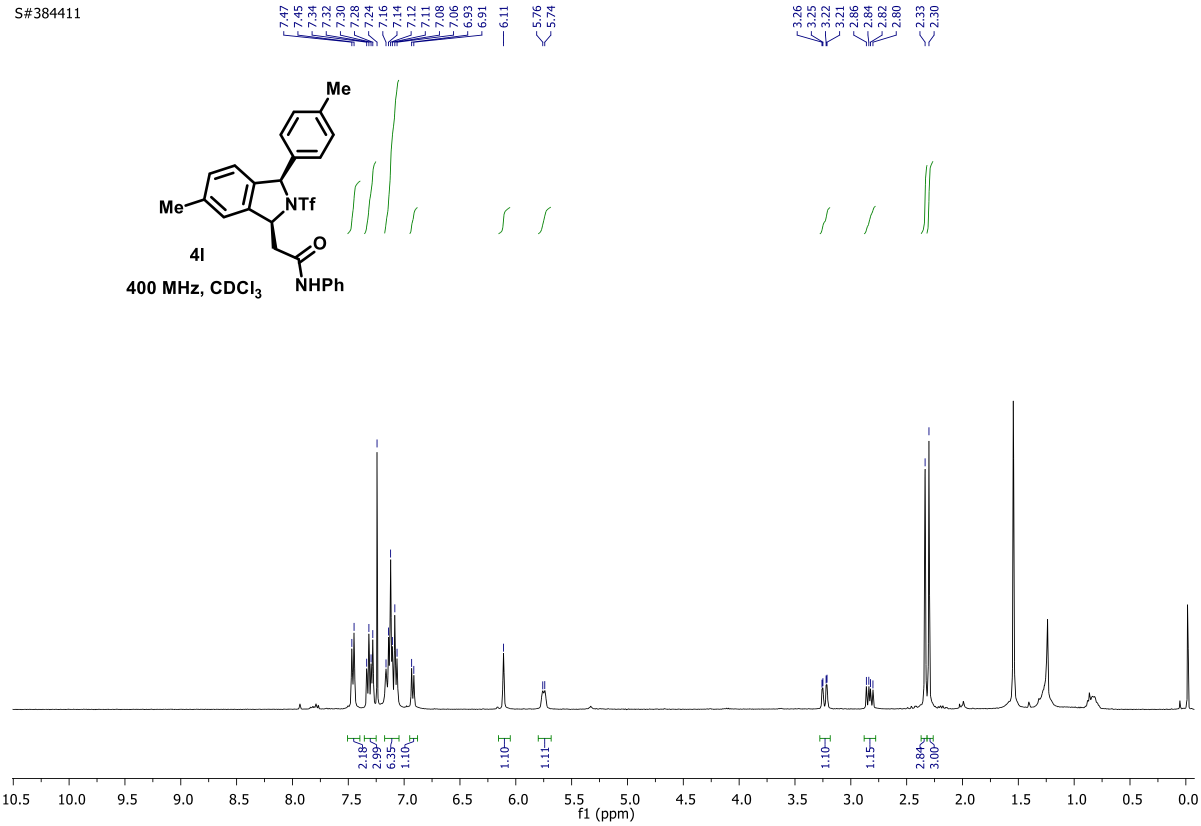
S#384411
7.47
7.45
7.34
7.32
7.30
7.28
7.24
7.16
7.14
7.12
7.11
7.08
7.06
6.93
6.91
6.11
5.76
5.74
3.26
3.25
3.22
3.21
2.86
2.84
2.82
2.80
2.33
2.30
Me
Me
NTf
O
4l
400 MHz, CDCl3
NHPh
2.18
2.99
6.35
1.10
1.10
1.11
1.10
1.15
2.84
3.00
f1 (ppm)

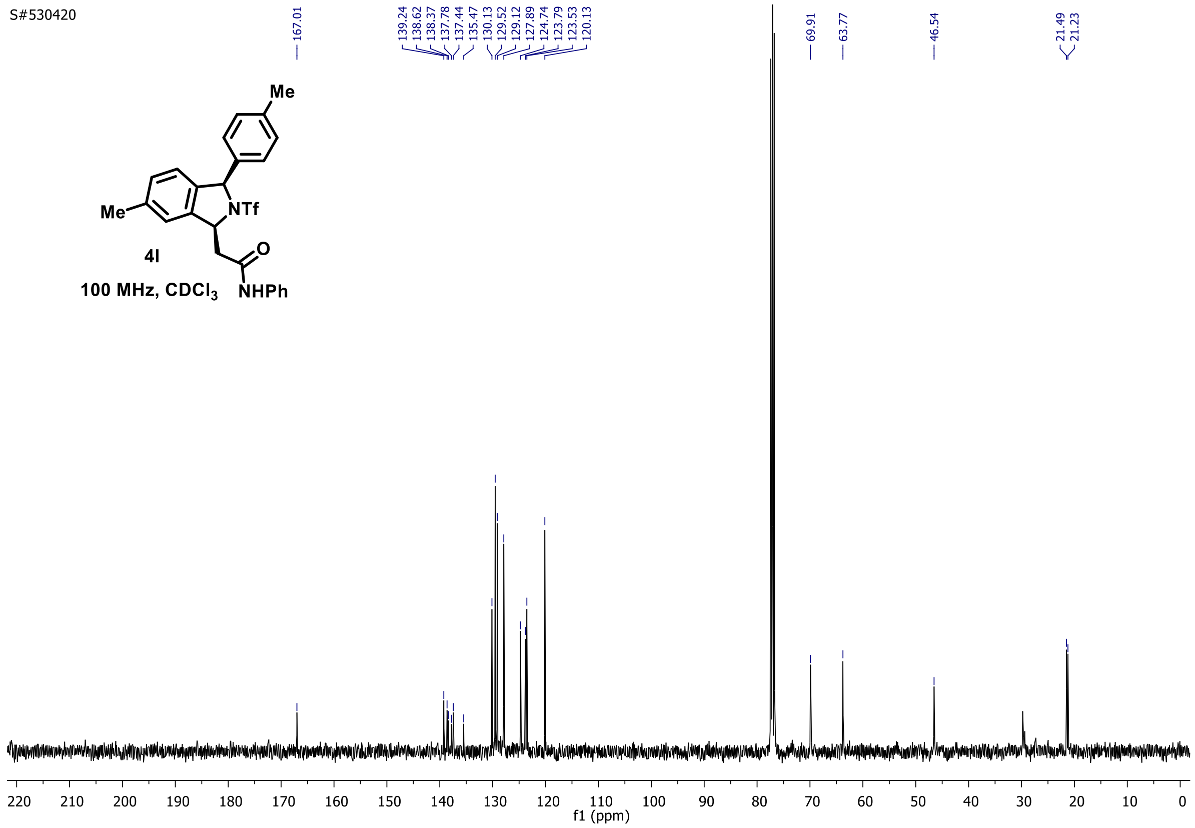
S#530420
167.01
139.24
138.62
138.37
137.78
137.44
135.47
130.13
129.52
129.12
127.89
124.74
123.79
123.53
120.13
69.91
63.77
46.54
21.49
21.23
Me
Me
NTf
O
4l
100 MHz, CDCl3
NHPh
f1 (ppm)

6. HPLC Spectra of Compounds 3 and 4

HPLC Chromatogram of Compound 3a (Racemic)

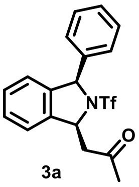

3a

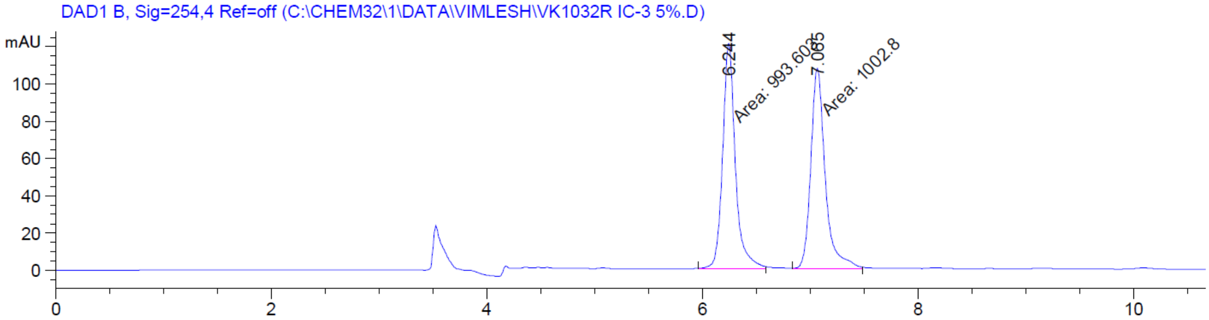

Peak #	RetTime [min]	Type	Width [min]	Area [mAU*s]	Height [mAU]	Area %
1	6.244	MM	0.1367	993.60272	121.16410	49.7697
2	7.065	MM	0.1552	1002.79694	107.65569	50.2303

HPLC Chromatogram of Compound 3a (Chiral)

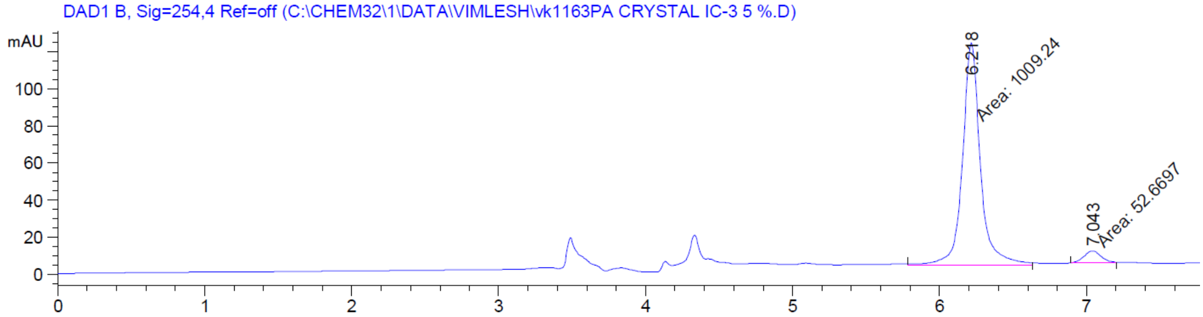

Peak #	RetTime [min]	Type	Width [min]	Area [mAU*s]	Height [mAU]	Area %
1	6.218	MM	0.1403	1009.24139	119.89330	95.0401
2	7.043	MM	0.1357	52.66973	6.47013	4.9599

HPLC Chromatogram of Compound 3b (Racemic)

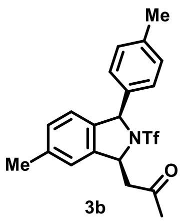

3b

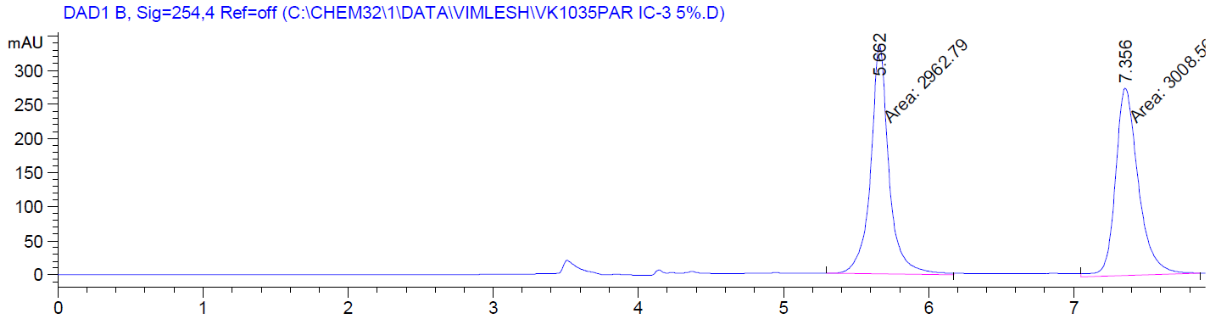

Peak #	RetTime [min]	Type	Width [min]	Area [mAU*s]	Height [mAU]	Area %
1	5.662	MM	0.1464	2962.78589	337.38437	49.6167
2	7.356	MM	0.1824	3008.55713	274.91068	50.3833

HPLC Chromatogram of Compound 3b (Chiral)

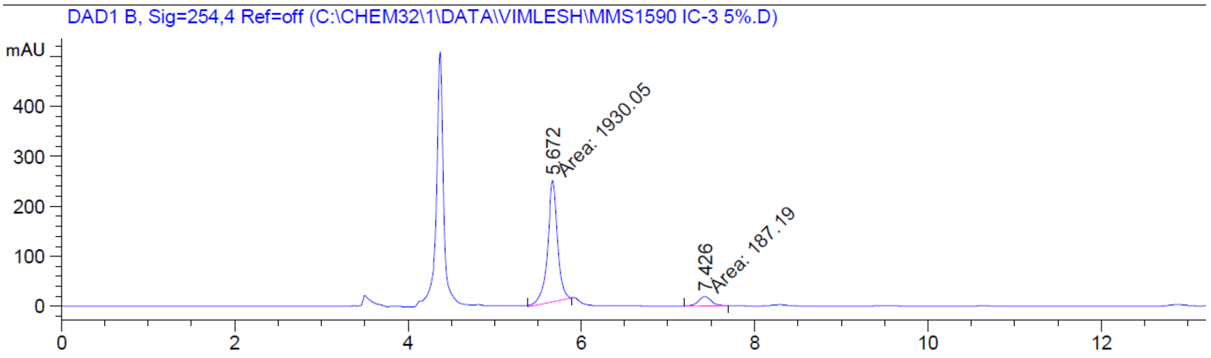

Peak #	RetTime [min]	Type	Width [min]	Area [mAU*s]	Height [mAU]	Area %
1	5.672	MM	0.1329	1930.05249	241.99738	91.1588
2	7.426	MM	0.1684	187.18967	18.52206	8.8412

HPLC Chromatogram of Compound 3c (Racemic)

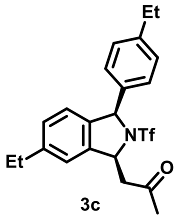

3c

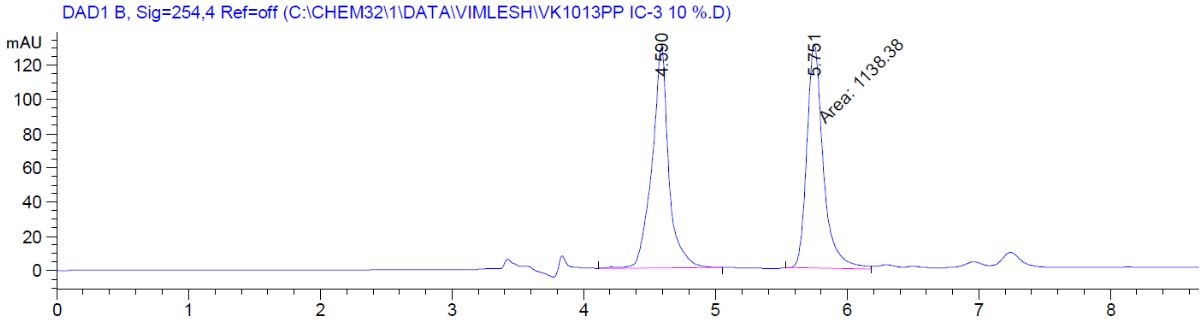

Peak #	RetTime [min]	Type	Width [min]	Area [mAU*s]	Height [mAU]	Area %
1	4.590	VB R	0.1230	1139.04150	128.99013	50.0144
2	5.751	MM	0.1447	1138.38367	131.12175	49.9856

HPLC Chromatogram of Compound 3c (Chiral)

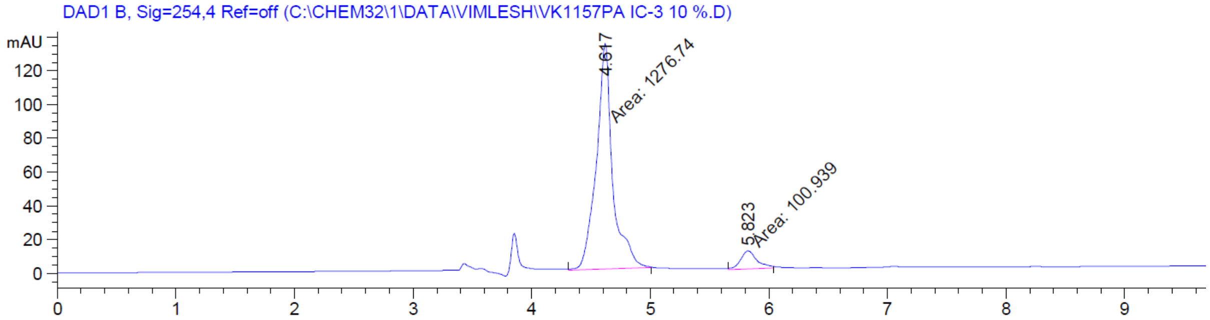

Peak #	RetTime [min]	Type	Width [min]	Area [mAU*s]	Height [mAU]	Area %
1	4.617	MM	0.1594	1276.74133	133.52121	92.6733
2	5.823	MM	0.1581	100.93903	10.64403	7.3267

HPLC Chromatogram of Compound 3d (Racemic)

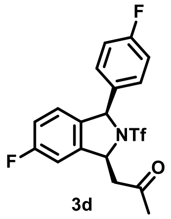

3d

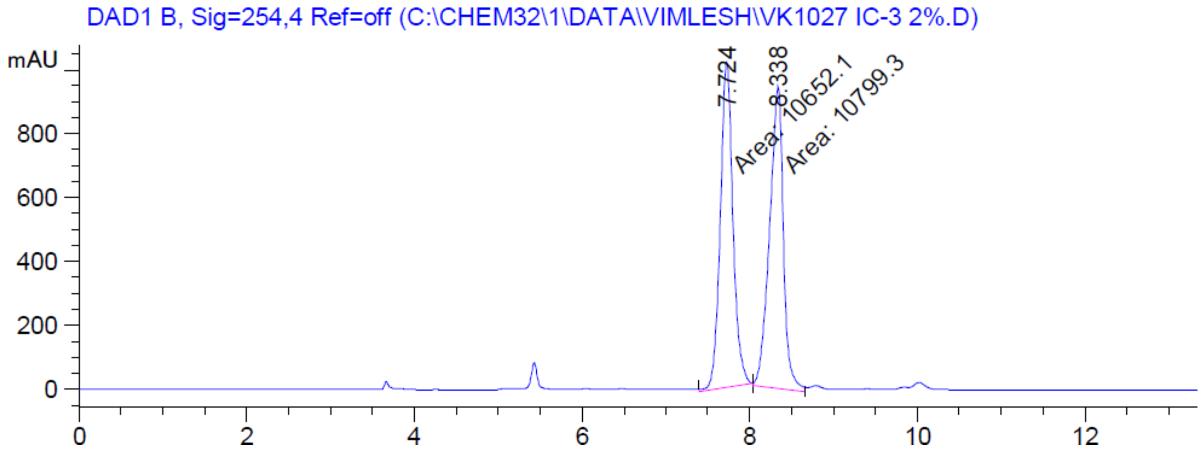

Peak #	RetTime [min]	Type	Width [min]	Area [mAU*s]	Height [mAU]	Area %
1	7.724	MM	0.1743	1.06521e4	1018.49365	49.6569
2	8.338	MM	0.1906	1.07993e4	944.29584	50.3431

HPLC Chromatogram of Compound 3d (Chiral)

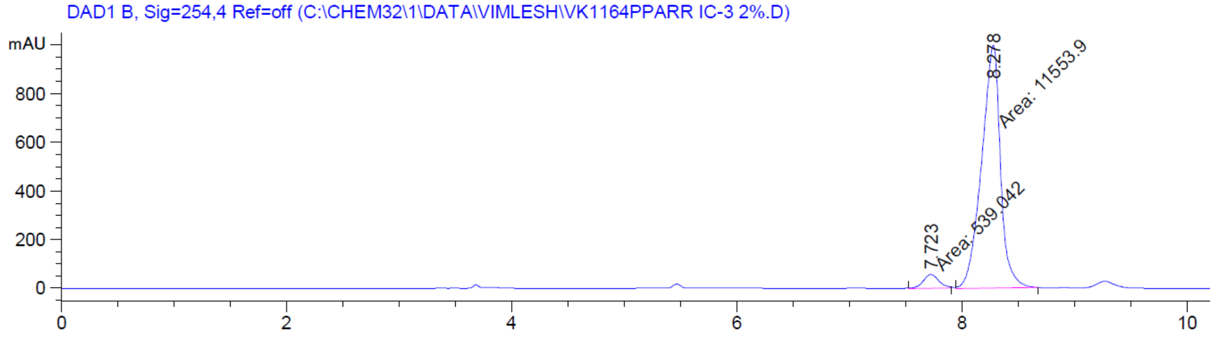

Peak #	RetTime [min]	Type	Width [min]	Area [mAU*s]	Height [mAU]	Area %
1	7.723	MM	0.1578	539.04181	56.94296	4.4575
2	8.278	MM	0.1929	1.15539e4	998.44409	95.5425

HPLC Chromatogram of Compound 3e (Racemic)

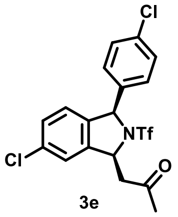

3e

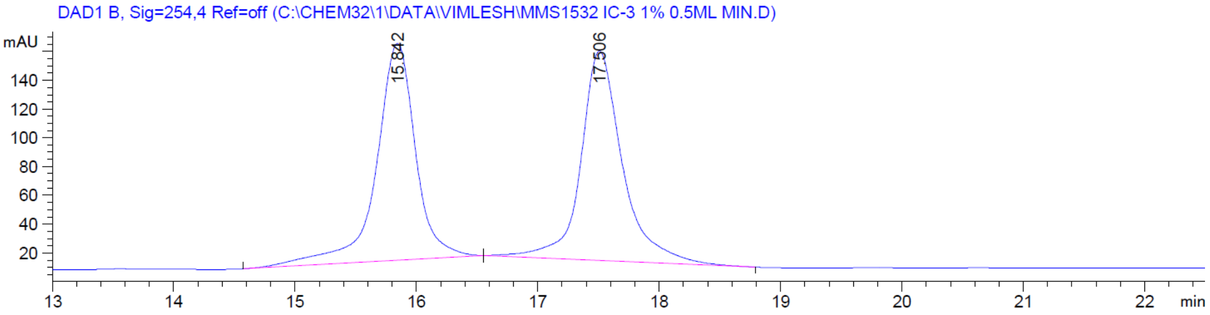

Peak #	RetTime [min]	Type	Width [min]	Area [mAU*s]	Height [mAU]	Area %
1	15.842	BB	0.3378	3408.47729	150.72656	49.4237
2	17.506	BB	0.3557	3487.96191	145.48553	50.5763

HPLC Chromatogram of Compound 3e (Chiral)

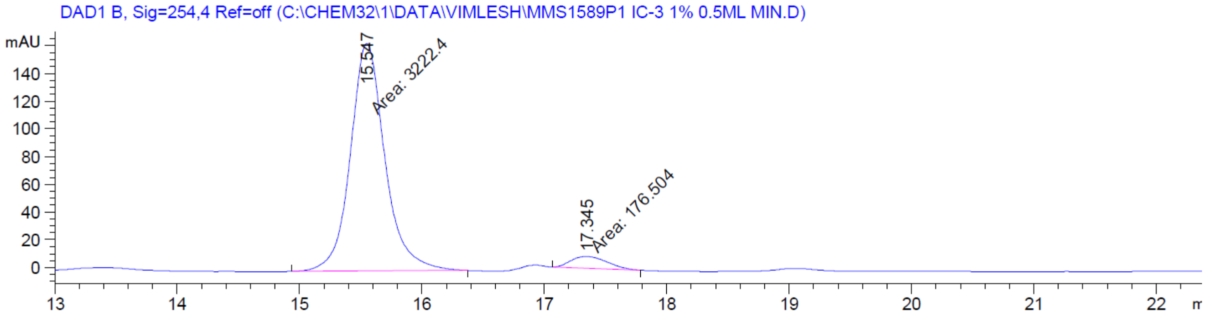

Peak #	RetTime [min]	Type	Width [min]	Area [mAU*s]	Height [mAU]	Area %
1	15.547	MM	0.3280	3239.69238	164.60991	94.9706
2	17.338	MM	0.3347	171.56528	8.54222	5.0294

HPLC Chromatogram of Compound 3f (Racemic)

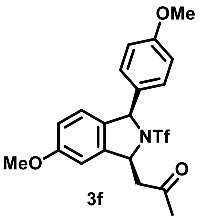

3f

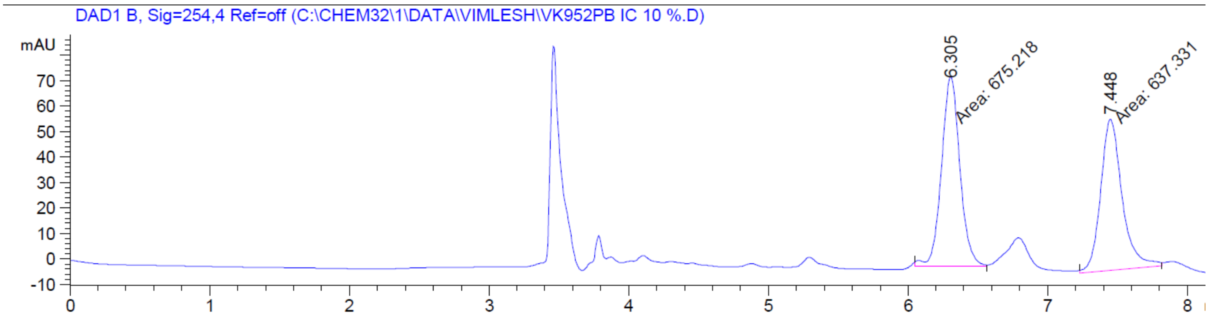

Peak #	RetTime [min]	Type	Width [min]	Area [mAU*s]	Height [mAU]	Area %
1	6.305	MM	0.1513	675.21777	74.38501	51.4433
2	7.448	MM	0.1788	637.33069	59.41415	48.5567

HPLC Chromatogram of Compound 3f (Chiral)

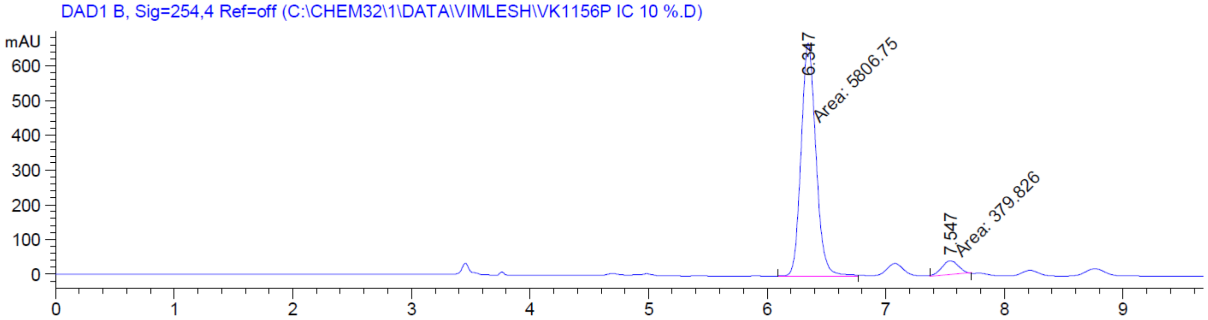

Peak #	RetTime [min]	Type	Width [min]	Area [mAU*s]	Height [mAU]	Area %
1	6.347	MM	0.1444	5806.75391	670.22614	93.8605
2	7.547	MM	0.1608	379.82629	39.37726	6.1395

HPLC Chromatogram of Compound 3g (Racemic)

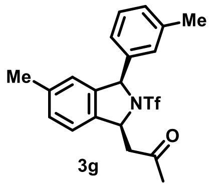

3g

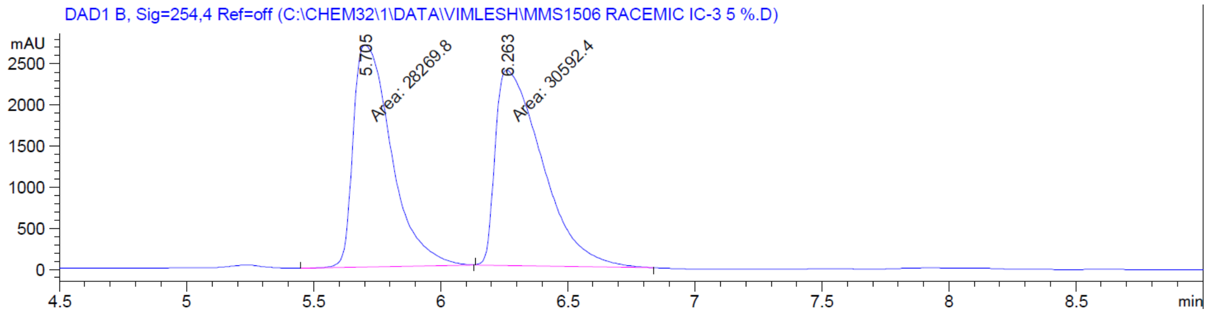

Peak #	RetTime [min]	Type	Width [min]	Area [mAU*s]	Height [mAU]	Area %
1	5.705	MM	0.1745	2.82698e4	2699.43188	48.0271
2	6.263	MM	0.2147	3.05924e4	2375.30981	51.9729

HPLC Chromatogram of Compound 3g (Chiral)

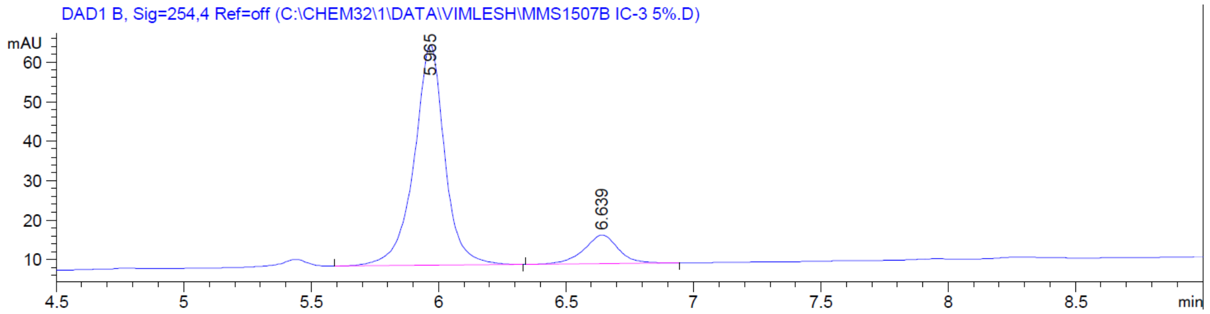

Peak #	RetTime [min]	Type	Width [min]	Area [mAU*s]	Height [mAU]	Area %
1	5.965	MM	0.1408	473.16995	56.02436	87.4266
2	6.639	MM	0.1557	68.04948	7.28279	12.5734

HPLC Chromatogram of Compound 3h (Racemic)

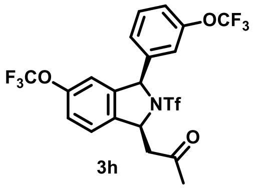

3h

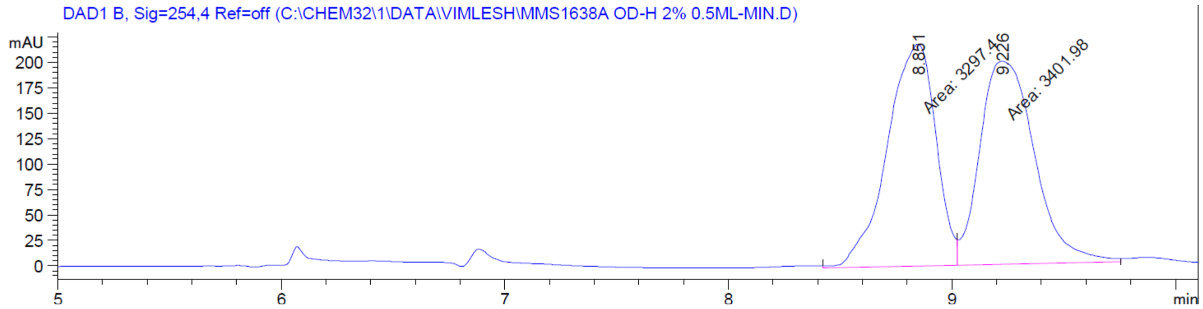

Peak #	RetTime [min]	Type	Width [min]	Area [mAU*s]	Height [mAU]	Area %
1	8.851	MF	0.2515	3297.40234	218.53894	49.2195
2	9.226	FM	0.2839	3401.98364	199.69458	50.7805

HPLC Chromatogram of Compound 3h (Racemic)

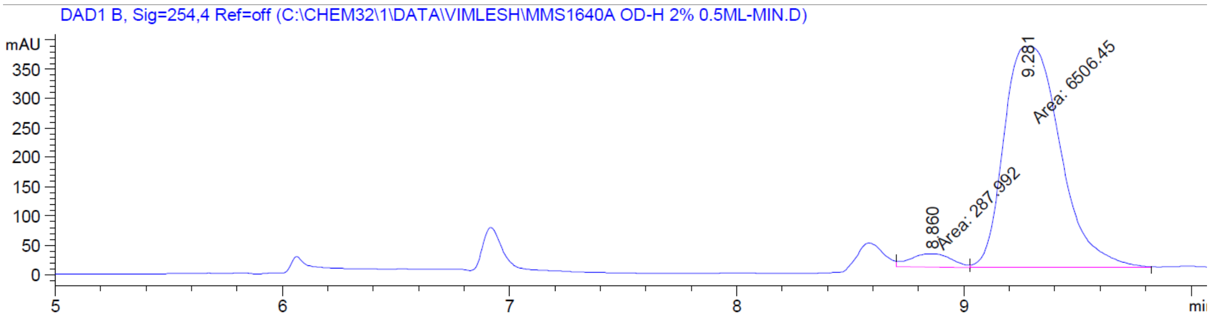

Peak #	RetTime [min]	Type	Width [min]	Area [mAU*s]	Height [mAU]	Area %
1	8.866	MM	0.2121	297.47137	23.37257	4.3662
2	9.281	MM	0.2880	6515.60742	377.09998	95.6338

HPLC Chromatogram of Compound 3i (Racemic)

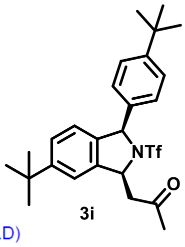

3i

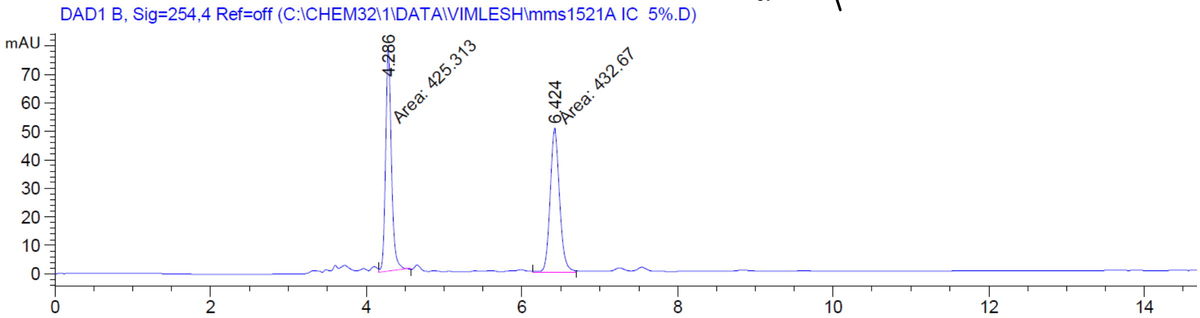

Peak #	RetTime [min]	Type	Width [min]	Area [mAU*s]	Height [mAU]	Area %
1	4.286	MM	0.0890	425.31323	79.64146	49.5713
2	6.424	MM	0.1427	432.67014	50.53651	50.4287

HPLC Chromatogram of Compound 3i (chiral)

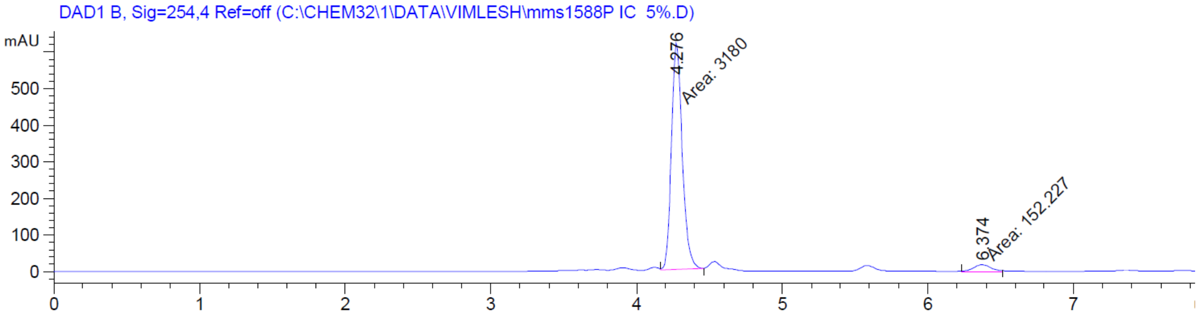

Peak #	RetTime [min]	Type	Width [min]	Area [mAU*s]	Height [mAU]	Area %
1	4.276	MM	0.0855	3179.99658	619.74030	95.4317
2	6.374	MM	0.1381	152.22742	18.36839	4.5683

HPLC Chromatogram of Compound 3j (Racemic)

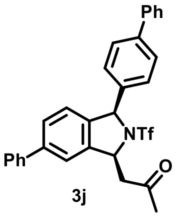

3j

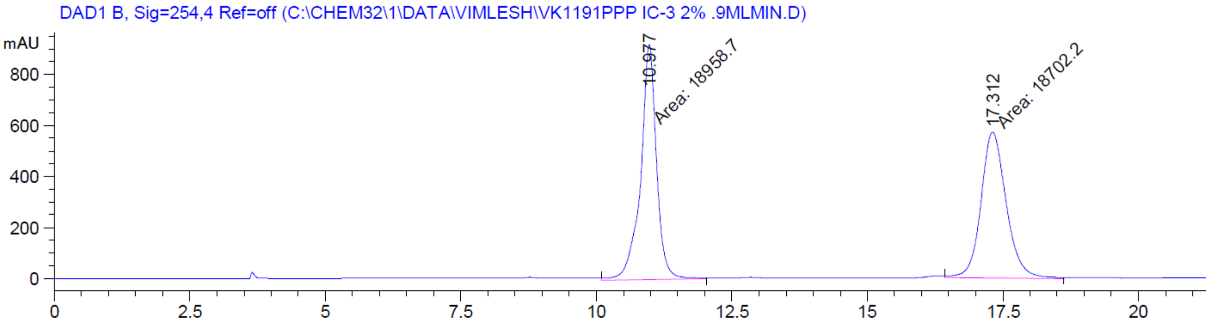

Peak #	RetTime [min]	Type	Width [min]	Area [mAU*s]	Height [mAU]	Area %
1	10.977	MM	0.3434	1.89587e4	920.15253	50.3405
2	17.312	MM	0.5447	1.87022e4	572.20557	49.6595

HPLC Chromatogram of Compound 3j (Chiral)

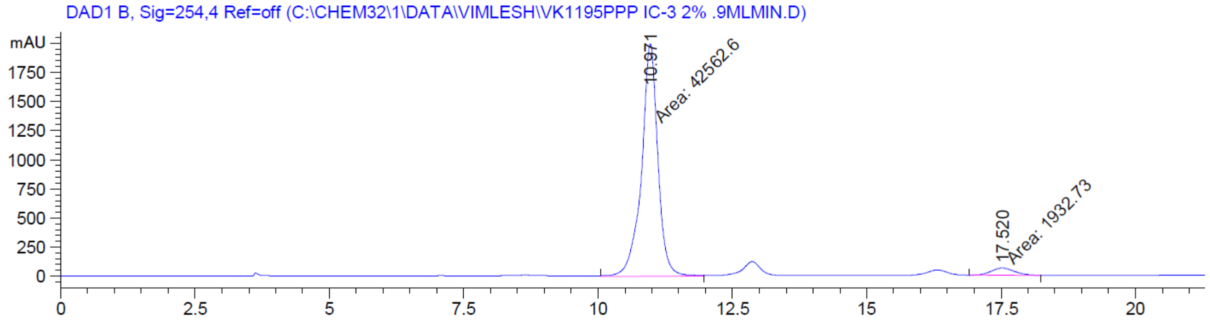

Peak #	RetTime [min]	Type	Width [min]	Area [mAU*s]	Height [mAU]	Area %
1	10.971	MM	0.3553	4.25626e4	1996.31531	95.6563
2	17.520	MM	0.5117	1932.72607	62.95689	4.3437

HPLC Chromatogram of Compound 3k (Racemic)

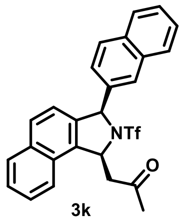

3k

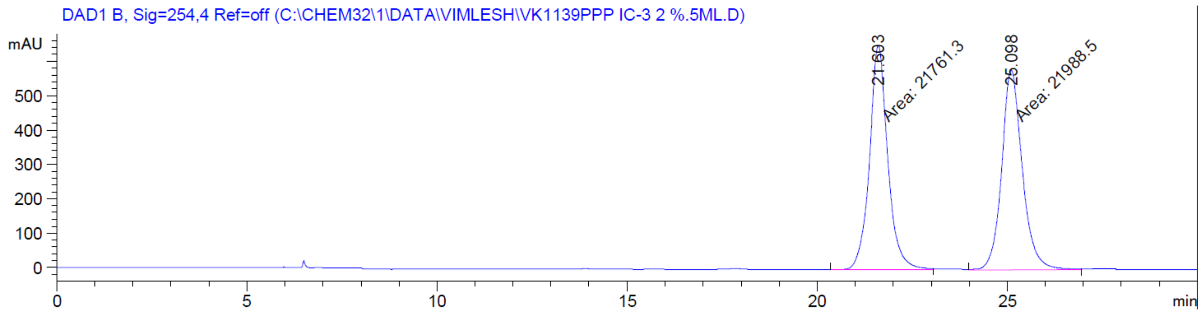

Peak #	RetTime [min]	Type	Width [min]	Area [mAU*s]	Height [mAU]	Area %
1	21.603	MM	0.5564	2.17613e4	651.86334	49.7403
2	25.098	MM	0.6298	2.19885e4	581.87134	50.2597

HPLC Chromatogram of Compound 3k (chiral)

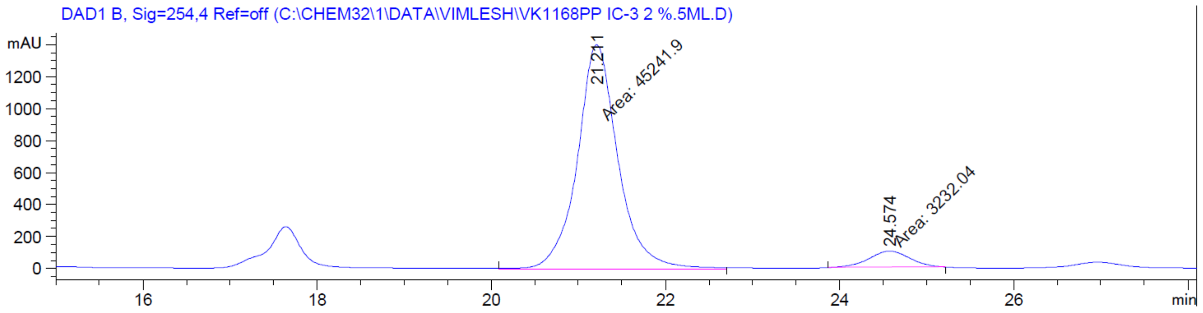

Peak #	RetTime [min]	Type	Width [min]	Area [mAU*s]	Height [mAU]	Area %
1	21.211	MM	0.5340	4.49417e4	1402.58838	92.3579
2	24.574	MM	0.5812	3718.68262	106.64301	7.6421

HPLC Chromatogram of Compound 3l (Racemic)

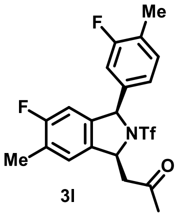

3l

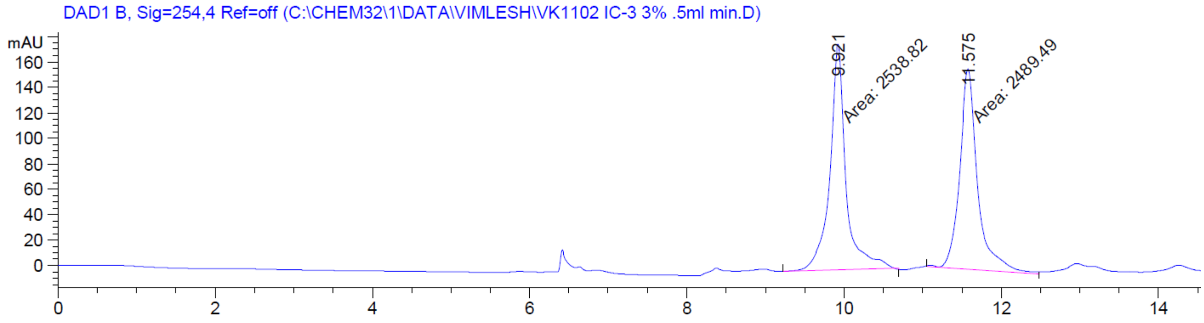

Peak #	RetTime [min]	Type	Width [min]	Area [mAU*s]	Height [mAU]	Area %
1	9.921	MM	0.2384	2538.82202	177.47005	50.4906
2	11.575	MM	0.2636	2489.48755	157.40889	49.5094

HPLC Chromatogram of Compound 3l (Chiral)

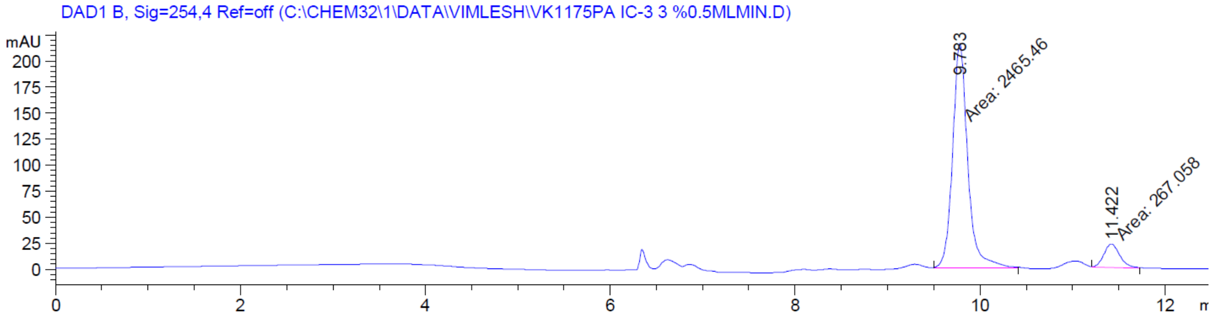

Peak #	RetTime [min]	Type	Width [min]	Area [mAU*s]	Height [mAU]	Area %
1	9.783	MM	0.1904	2465.45923	215.80150	90.2267
2	11.422	MM	0.1983	267.05786	22.45020	9.7733

HPLC Chromatogram of Compound 3m (Racemic)

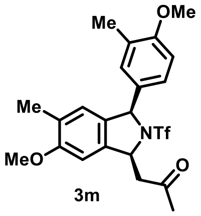

3m

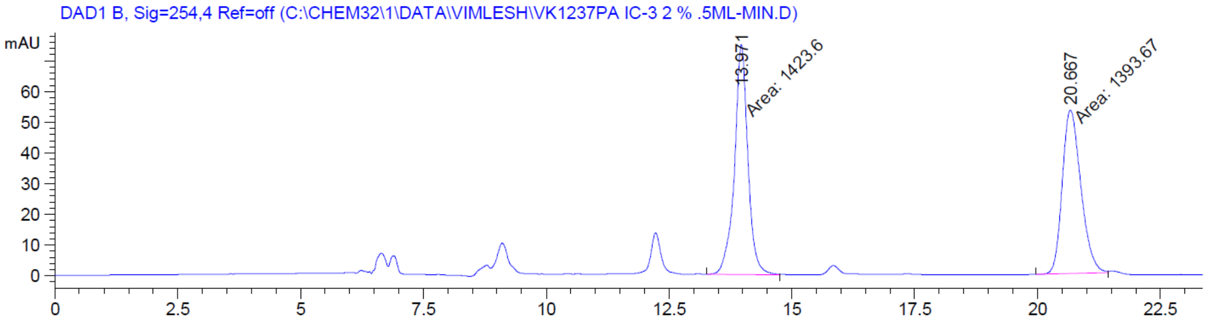

Peak #	RetTime [min]	Type	Width [min]	Area [mAU*s]	Height [mAU]	Area %
1	13.971	MM	0.3173	1423.60010	74.78687	50.5313
2	20.667	MM	0.4372	1393.66541	53.12901	49.4687

HPLC Chromatogram of Compound 3m (Chiral)

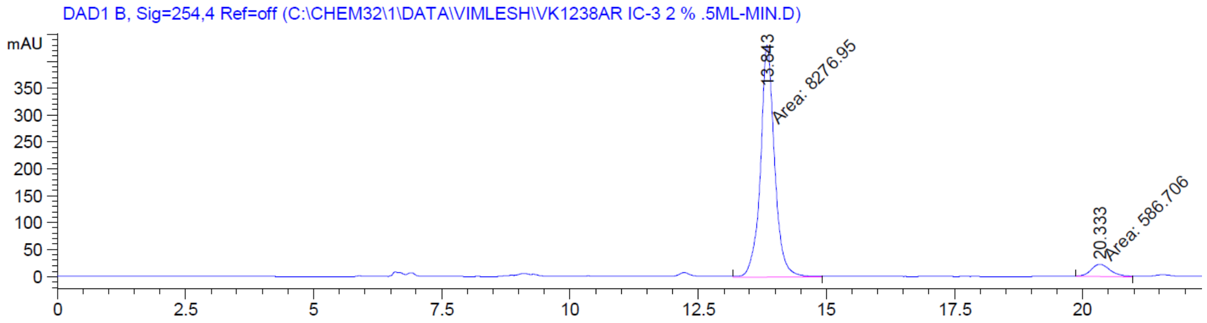

Peak #	RetTime [min]	Type	Width [min]	Area [mAU*s]	Height [mAU]	Area %
1	13.843	MM	0.3197	8276.94531	431.42938	93.3808
2	20.333	MM	0.4326	586.70599	22.60211	6.6192

HPLC Chromatogram of Compound 3n (Racemic)

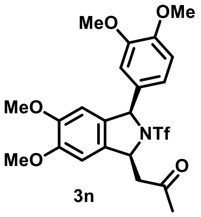

3n

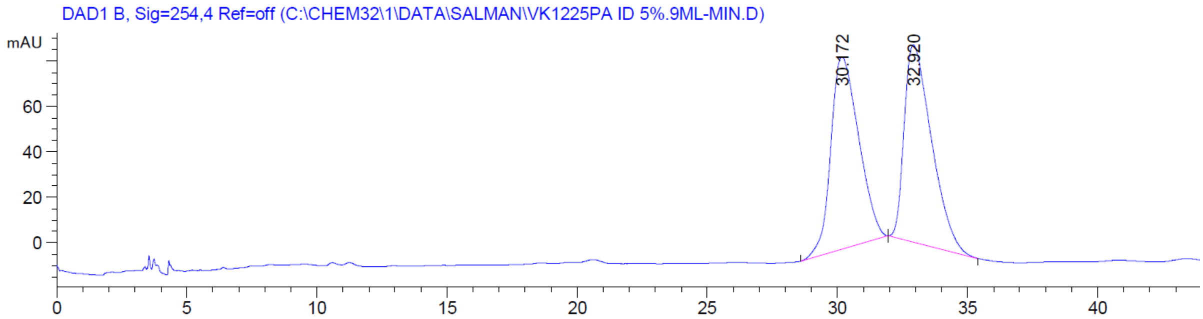

Peak #	RetTime [min]	Type	Width [min]	Area [mAU*s]	Height [mAU]	Area %
1	30.172	BB	1.0990	6306.24316	84.66151	49.7696
2	32.920	BB	1.0724	6364.63818	86.90709	50.2304

HPLC Chromatogram of Compound 3n (Chiral)

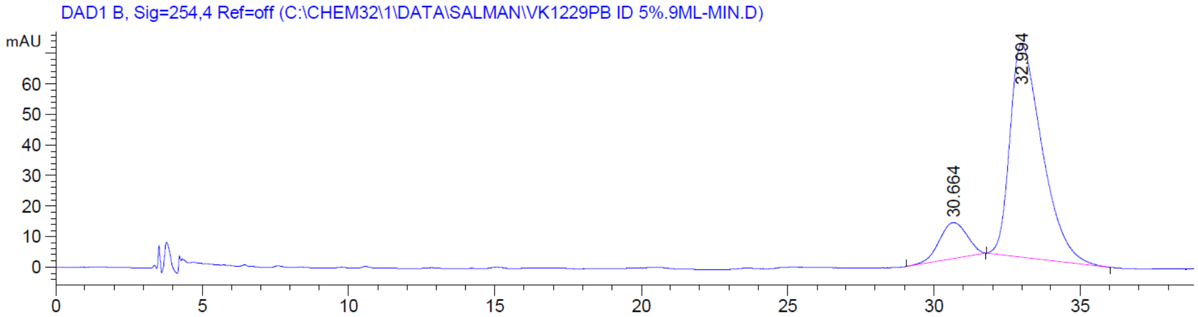

Peak #	RetTime [min]	Type	Width [min]	Area [mAU*s]	Height [mAU]	Area %
1	30.664	BB	0.7859	772.06158	11.79151	12.6554
2	32.994	BB	1.0851	5328.58838	69.91817	87.3446

HPLC Chromatogram of Compound 4b (Racemic)

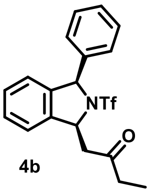

4b

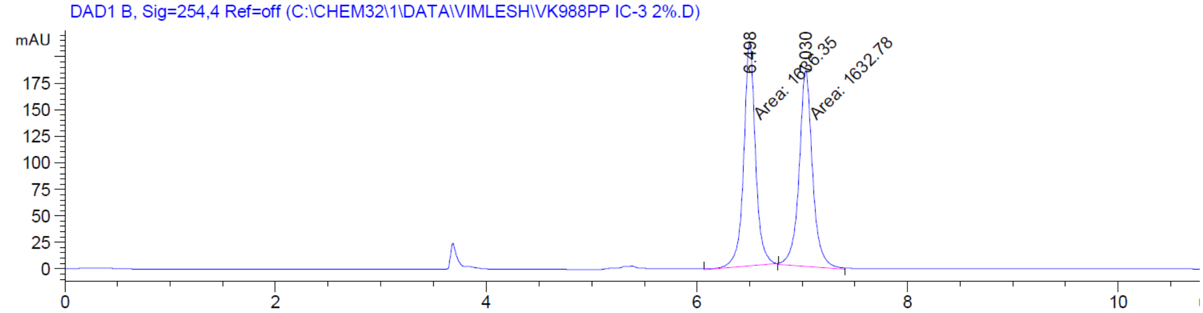

Peak #	RetTime [min]	Type	Width [min]	Area [mAU*s]	Height [mAU]	Area %
1	6.498	MM	0.1292	1636.35352	211.15944	50.0546
2	7.030	MM	0.1474	1632.78479	184.55968	49.9454

HPLC Chromatogram of Compound 4b (Chiral)

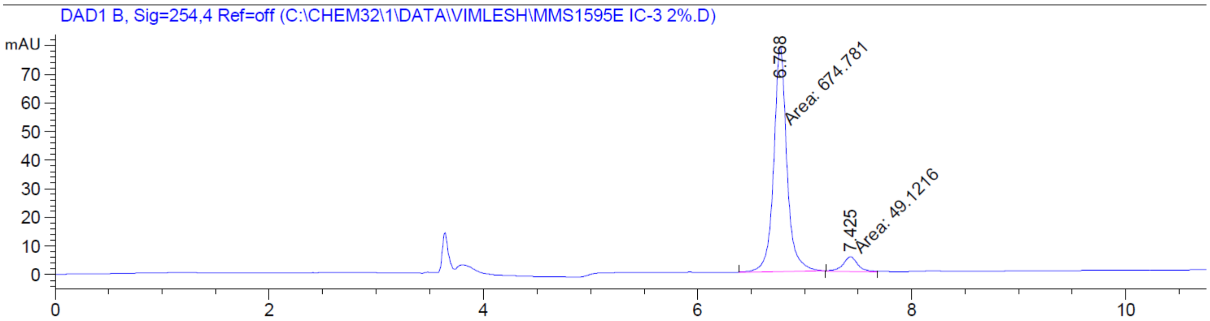

Peak #	RetTime [min]	Type	Width [min]	Area [mAU*s]	Height [mAU]	Area %
1	6.768	MM	0.1430	674.78076	78.66550	93.2143
2	7.425	MM	0.1593	49.12161	5.13943	6.7857

HPLC Chromatogram of Compound 4c (Racemic)

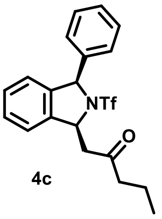

4c

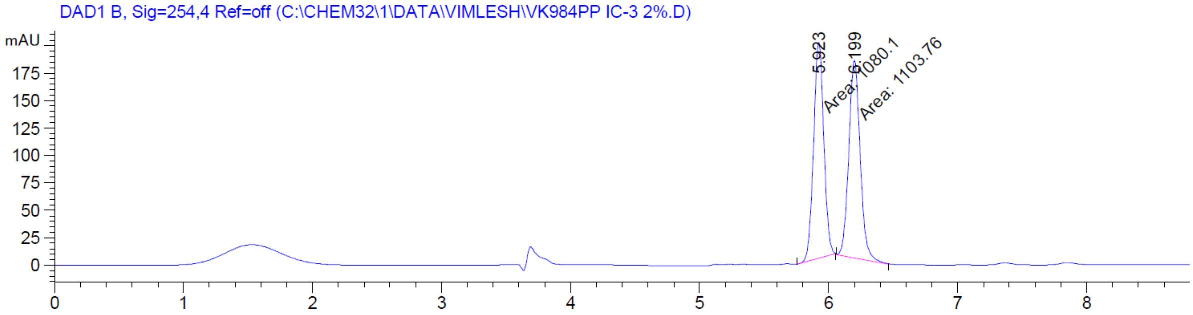

Peak #	RetTime [min]	Type	Width [min]	Area [mAU*s]	Height [mAU]	Area %
1	5.923	MM	0.0911	1080.09863	197.59573	49.4582
2	6.199	MM	0.1021	1103.76428	180.20326	50.5418

HPLC Chromatogram of Compound 4c (Chiral)

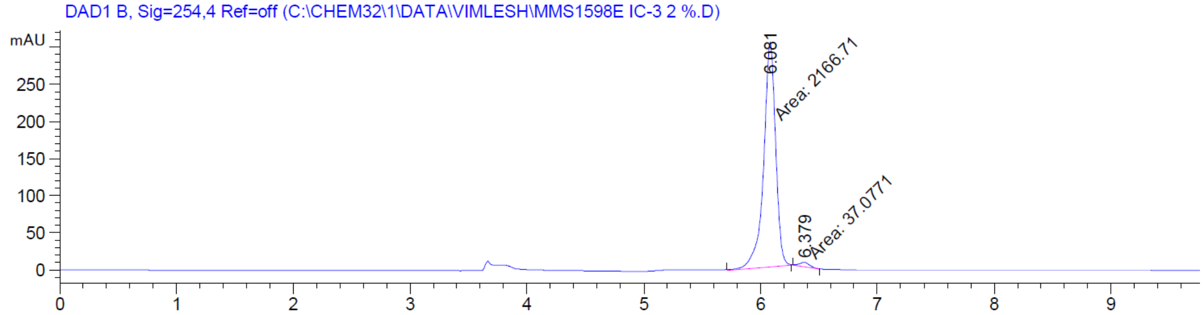

Peak #	RetTime [min]	Type	Width [min]	Area [mAU*s]	Height [mAU]	Area %
1	6.081	MM	0.1190	2166.70752	303.43588	98.3176
2	6.379	MM	0.1043	37.07708	5.92617	1.6824

HPLC Chromatogram of Compound 4d (Racemic)

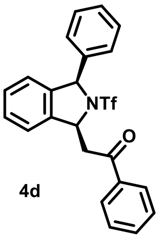

4d

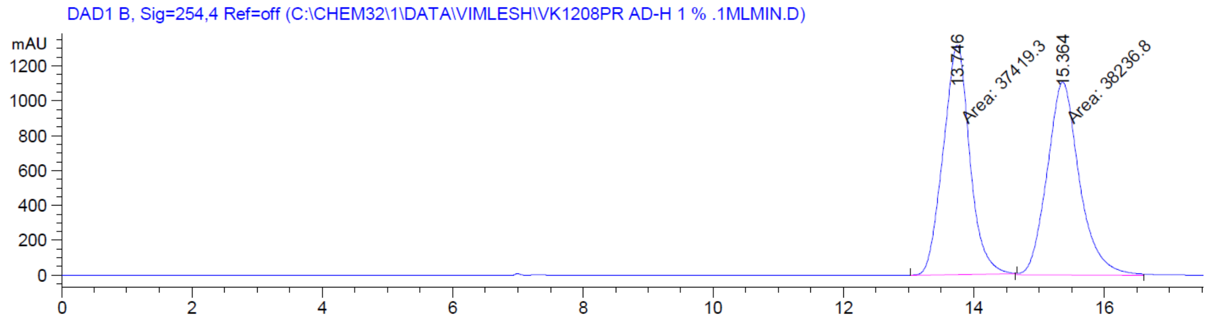

Peak #	RetTime [min]	Type	Width [min]	Area [mAU*s]	Height [mAU]	Area %
1	13.746	MM	0.4736	3.74193e4	1316.88147	49.4597
2	15.364	MM	0.5754	3.82368e4	1107.56274	50.5403

HPLC Chromatogram of Compound 4d (Chiral)

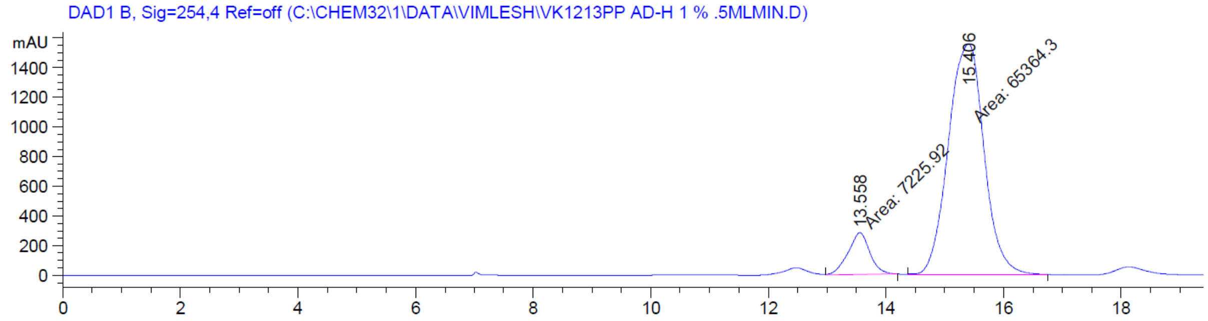

Peak #	RetTime [min]	Type	Width [min]	Area [mAU*s]	Height [mAU]	Area %
1	13.558	MM	0.4276	7225.92334	281.66183	9.9544
2	15.406	MM	0.7024	6.53643e4	1550.87988	90.0456

HPLC Chromatogram of Compound 4e (Racemic)

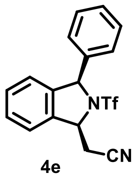

4e

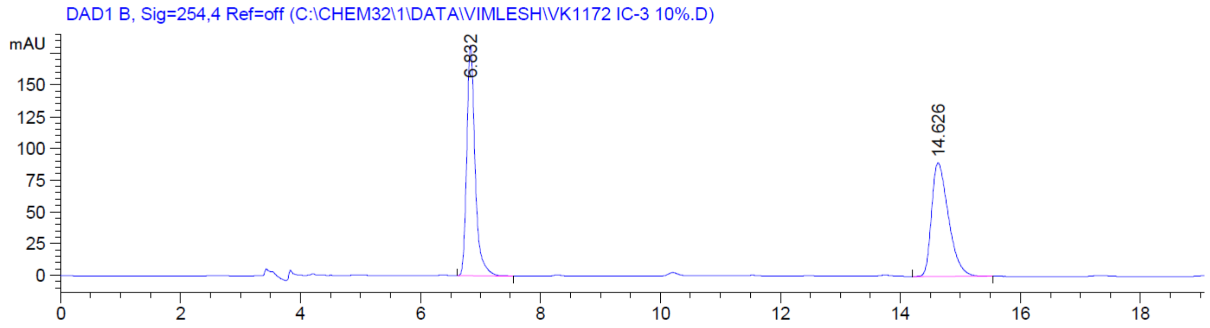

Peak #	RetTime [min]	Type	Width [min]	Area [mAU*s]	Height [mAU]	Area %
1	6.832	VB	0.1466	1752.72791	181.66571	50.0324
2	14.626	BB	0.3022	1750.46118	89.49341	49.9676

HPLC Chromatogram of Compound 4e (Chiral)

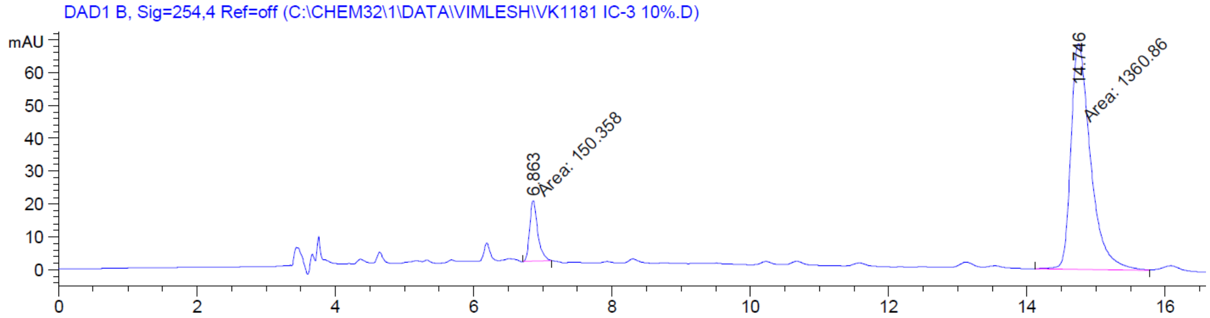

Peak #	RetTime [min]	Type	Width [min]	Area [mAU*s]	Height [mAU]	Area %
1	6.863	MM	0.1355	150.35840	18.49384	9.9495
2	14.746	MM	0.3297	1360.85681	68.79140	90.0505

HPLC Chromatogram of Compound 4f (Racemic)

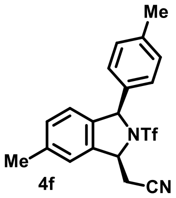

4f

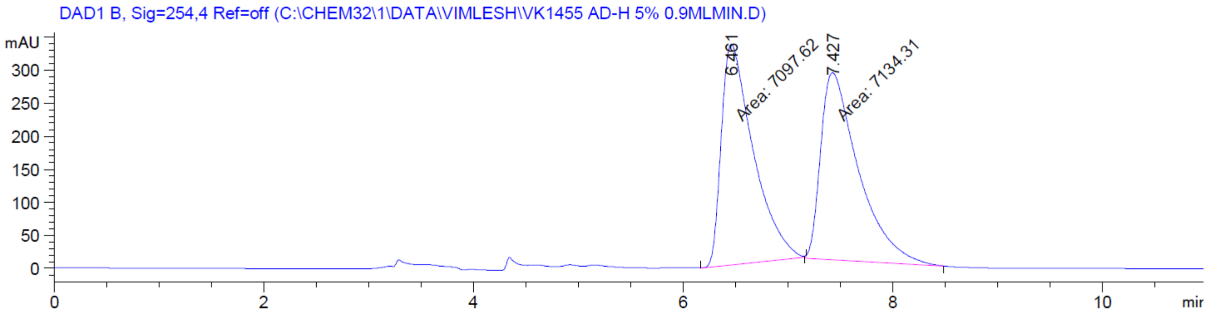

Peak #	RetTime [min]	Type	Width [min]	Area [mAU*s]	Height [mAU]	Area %
1	6.461	MM	0.3539	7097.61719	334.28638	49.8711
2	7.427	MM	0.4197	7134.30811	283.31888	50.1289

HPLC Chromatogram of Compound 4f (chiral)

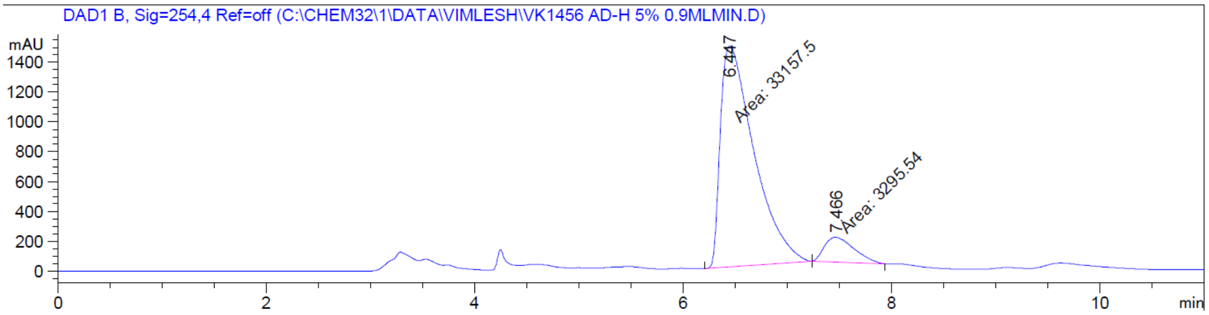

Peak #	RetTime [min]	Type	Width [min]	Area [mAU*s]	Height [mAU]	Area %
1	6.447	MM	0.3735	3.31575e4	1479.44995	90.9595
2	7.466	MM	0.3323	3295.53735	165.30466	9.0405

HPLC Chromatogram of Compound 4g (Racemic)

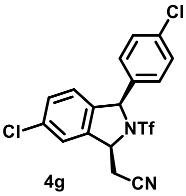

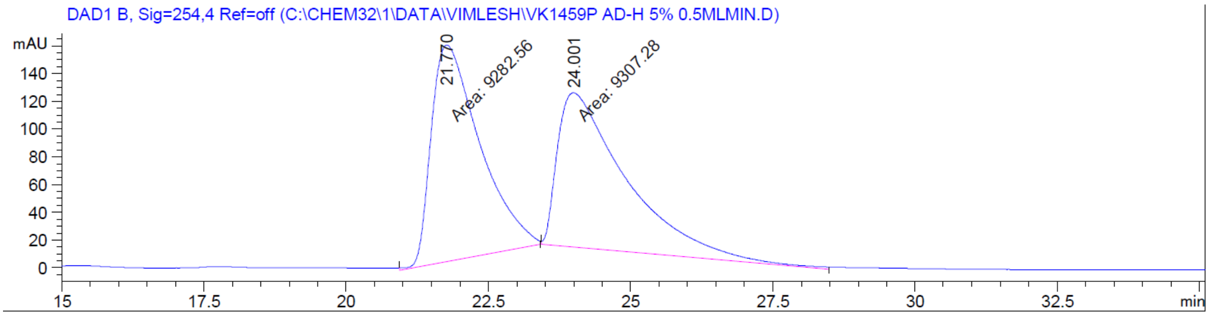

Peak #	RetTime [min]	Type	Width [min]	Area [mAU*s]	Height [mAU]	Area %
1	21.770	MM	0.9905	9282.55762	156.18779	49.9335
2	24.001	MM	1.3959	9307.27734	111.12739	50.0665

HPLC Chromatogram of Compound 4g (Chiral)

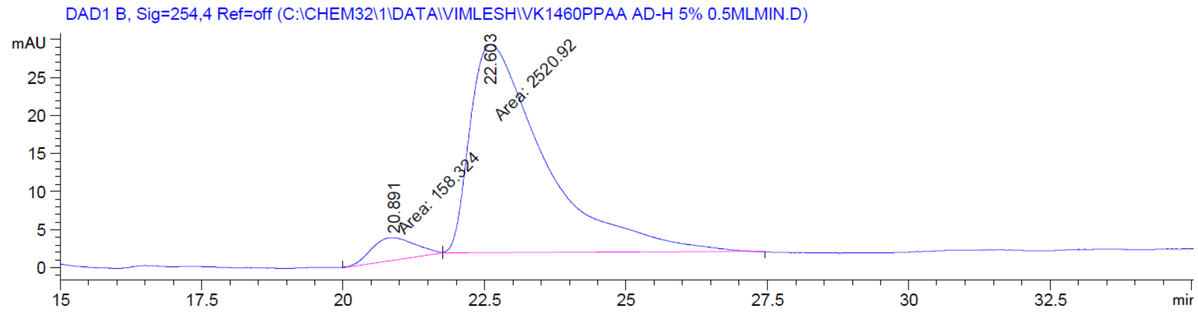

Peak #	RetTime [min]	Type	Width [min]	Area [mAU*s]	Height [mAU]	Area %
1	20.891	MM	0.8973	158.32405	2.94081	5.9093
2	22.603	MM	1.5356	2520.92065	27.36070	94.0907

HPLC Chromatogram of Compound 4h (Racemic)

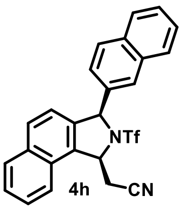

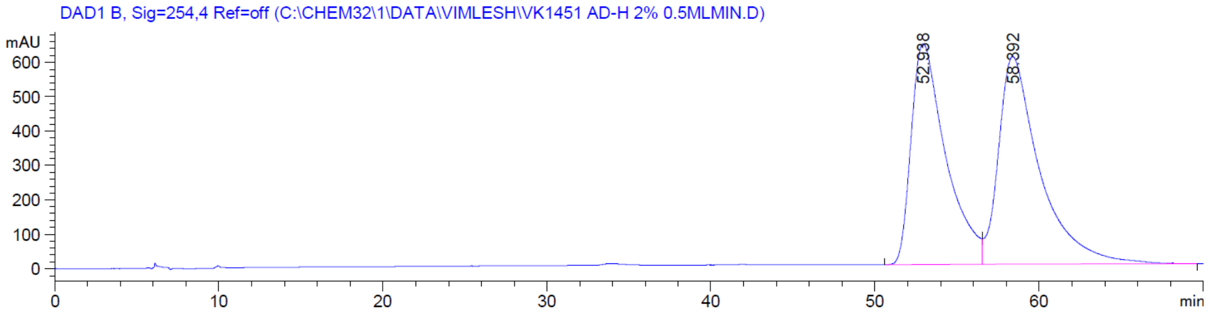

Signal 2: DAD1 B, Sig=254,4 Ref=off

Peak #	RetTime [min]	Type	Width [min]	Area [mAU*s]	Height [mAU]	Area %
1	52.938	MM	2.4574	9.46233e4	641.75934	47.6211
2	58.392	MM	2.8673	1.04077e5	604.96198	52.3789

HPLC Chromatogram of Compound 4h (Chiral)

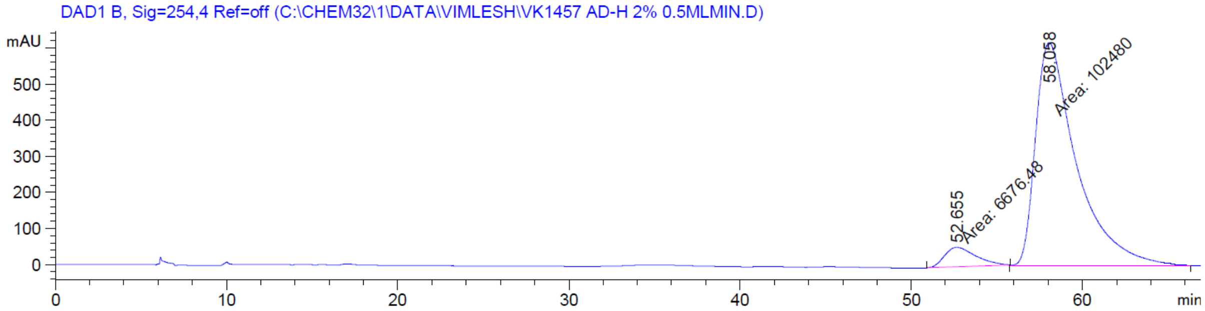

Peak #	RetTime [min]	Type	Width [min]	Area [mAU*s]	Height [mAU]	Area %
1	52.655	MM	2.0852	6676.47900	53.36424	6.1164
2	58.058	MM	2.7680	1.02480e5	617.04022	93.8836

HPLC Chromatogram of Compound 4i (Racemic)

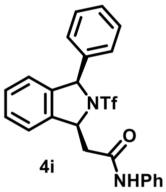

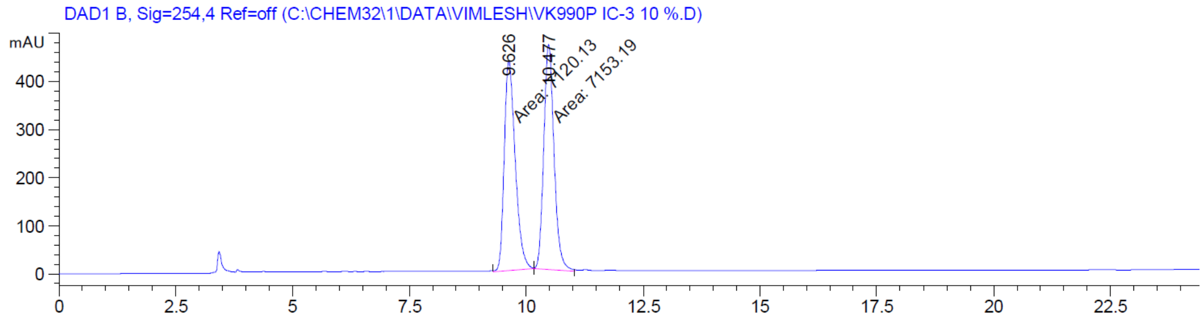

Peak #	RetTime [min]	Type	Width [min]	Area [mAU*s]	Height [mAU]	Area %
1	9.626	MM	0.2723	7120.13330	435.83905	49.8842
2	10.477	MM	0.2550	7153.18945	467.51151	50.1158

HPLC Chromatogram of Compound 4i (Chiral)

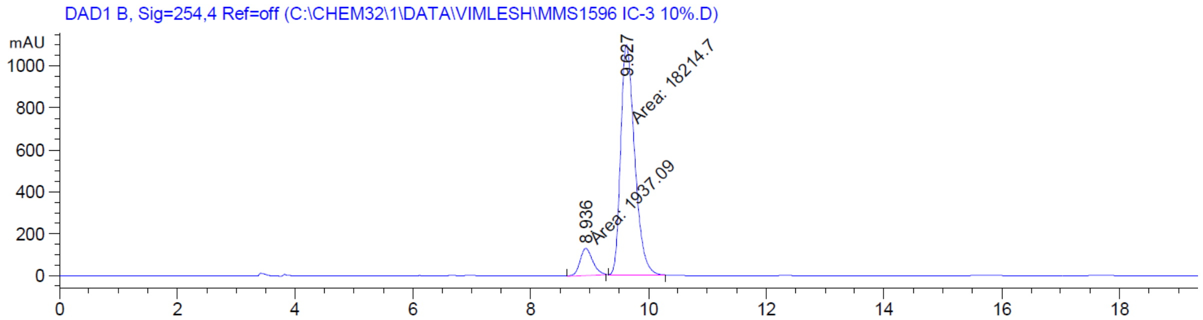

Peak #	RetTime [min]	Type	Width [min]	Area [mAU*s]	Height [mAU]	Area %
1	8.936	MM	0.2495	1937.09180	129.38841	9.6125
2	9.627	MM	0.2763	1.82147e4	1098.87183	90.3875

HPLC Chromatogram of Compound 4j (Racemic)

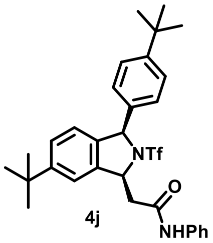

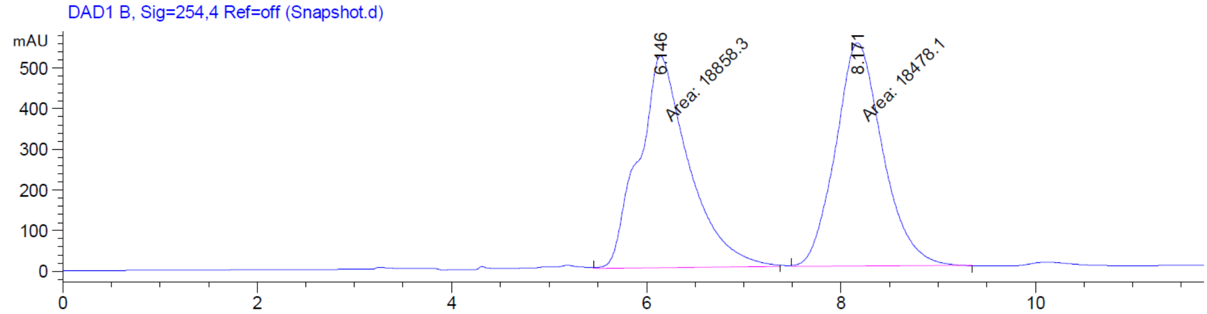

Peak #	RetTime [min]	Type	Width [min]	Area [mAU*s]	Height [mAU]	Area %
1	6.146	MM	0.6026	1.88583e4	521.60510	50.5093
2	8.171	MM	0.5612	1.84781e4	548.78101	49.4907

HPLC Chromatogram of Compound 4j (Chiral)

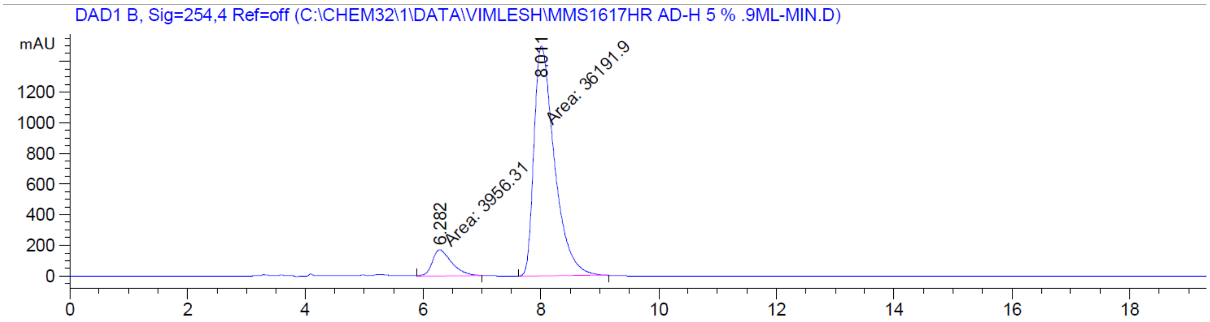

Peak #	RetTime [min]	Type	Width [min]	Area [mAU*s]	Height [mAU]	Area %
1	6.282	MM	0.3861	3956.31323	170.76566	9.8543
2	8.011	MM	0.4021	3.61919e4	1500.10315	90.1457

HPLC Chromatogram of Compound 4k (Racemic)

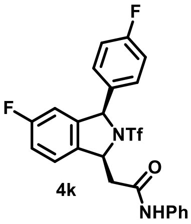

4k

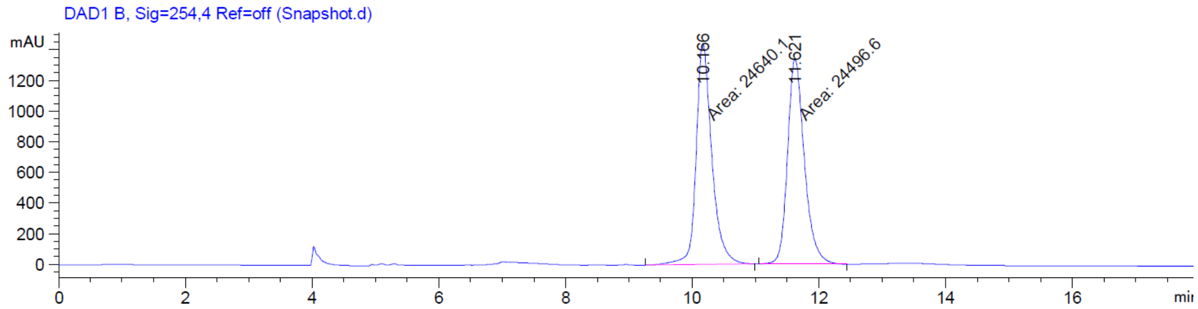

Peak #	RetTime [min]	Type	Width [min]	Area [mAU*s]	Height [mAU]	Area %
1	10.166	MM	0.2851	2.46401e4	1440.24182	50.1460
2	11.621	MM	0.3057	2.44966e4	1335.60474	49.8540

HPLC Chromatogram of Compound 4k (chiral)

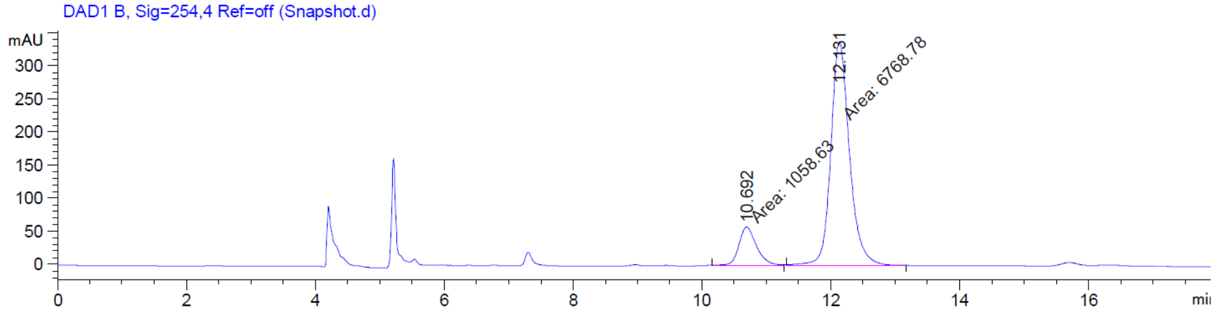

Peak #	RetTime [min]	Type	Width [min]	Area [mAU*s]	Height [mAU]	Area %
1	10.692	MM	0.3076	1058.63354	57.35867	13.5247
2	12.131	MM	0.3344	6768.78467	337.38974	86.4753

HPLC Chromatogram of Compound 4l (Racemic)

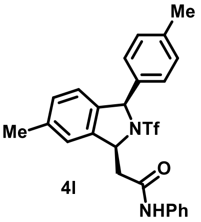

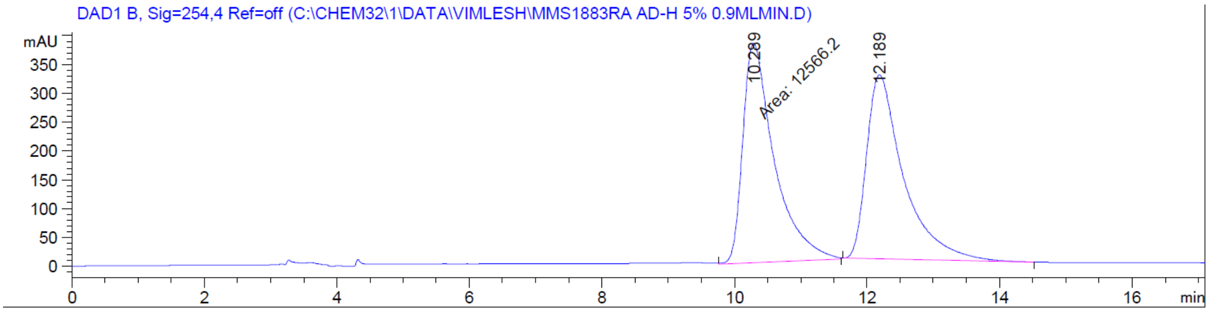

Peak #	RetTime [min]	Type	Width [min]	Area [mAU*s]	Height [mAU]	Area %
1	10.289	MM	0.5499	1.25662e4	380.89542	50.4109
2	12.189	BB	0.5659	1.23613e4	319.09689	49.5891

HPLC Chromatogram of Compound 4l (chiral)

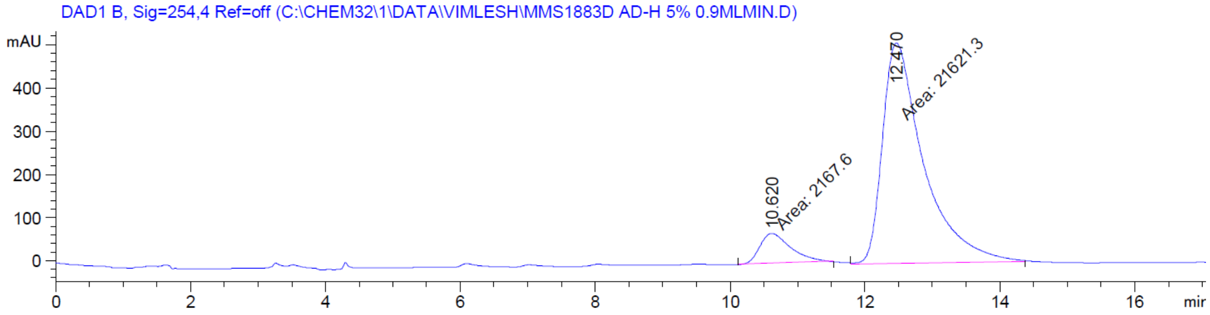

Peak #	RetTime [min]	Type	Width [min]	Area [mAU*s]	Height [mAU]	Area %
1	10.620	MM	0.5244	2167.60205	68.88584	9.1118
2	12.470	MM	0.7063	2.16213e4	510.22293	90.8882

7. Crystallographic Data of 3a

Table 1

Identification code	UPS_UK_1163_0m_a
Empirical formula	C18H16F3NO3S
Formula weight	383.38
Temperature	124 K
Crystal system	orthorhombic
Space group	P212121
Unit cell dimensions	a = 10.274(2) Å ;<br>α =90 º
	b = 11.716(4) Å ;<br>β = 90 º
	c = 14.422(4) Å;<br>γ = 90 º
Volume	1736.0(8) $Å^3$
Z	4
Density (calculated)	1.467 g/cm^3
Absorption coefficient	2.115 mm^{-1}
F(000)	792.0
Crystal size	0.343 × 0.32 × 0.12 mm^3
Radiation	Cu Kα (λ = 1.54178)
2Θ range for data collection	9.726 to 133.06
Index ranges	-12 ≤ h ≤ 12, -13 ≤ k ≤ 13, -17 ≤ l ≤ 17
Reflections collected	40412
Independent reflections	3009 [Rint = 0.0636, Rsigma = 0.0305]
Completeness of theta = 28.310º	98.00 %
Absorption correction	Semi-empirical from equivalents
Max. and min. transmission	0.508 and 0.776
Refinement method	Full-matrix least-square on F^2
Data/restraints/parameters	3009/0/237
Goodness-of-fit on F^2	1.063
Final R indexes [I>=2σ (I)]	R1 = 0.0317, wR2 = 0.0847
Final R indexes [all data]	R1 = 0.0318, wR2 = 0.0848
Largest diff. peak/hole	0.22/-0.30 $Å^{-3}$

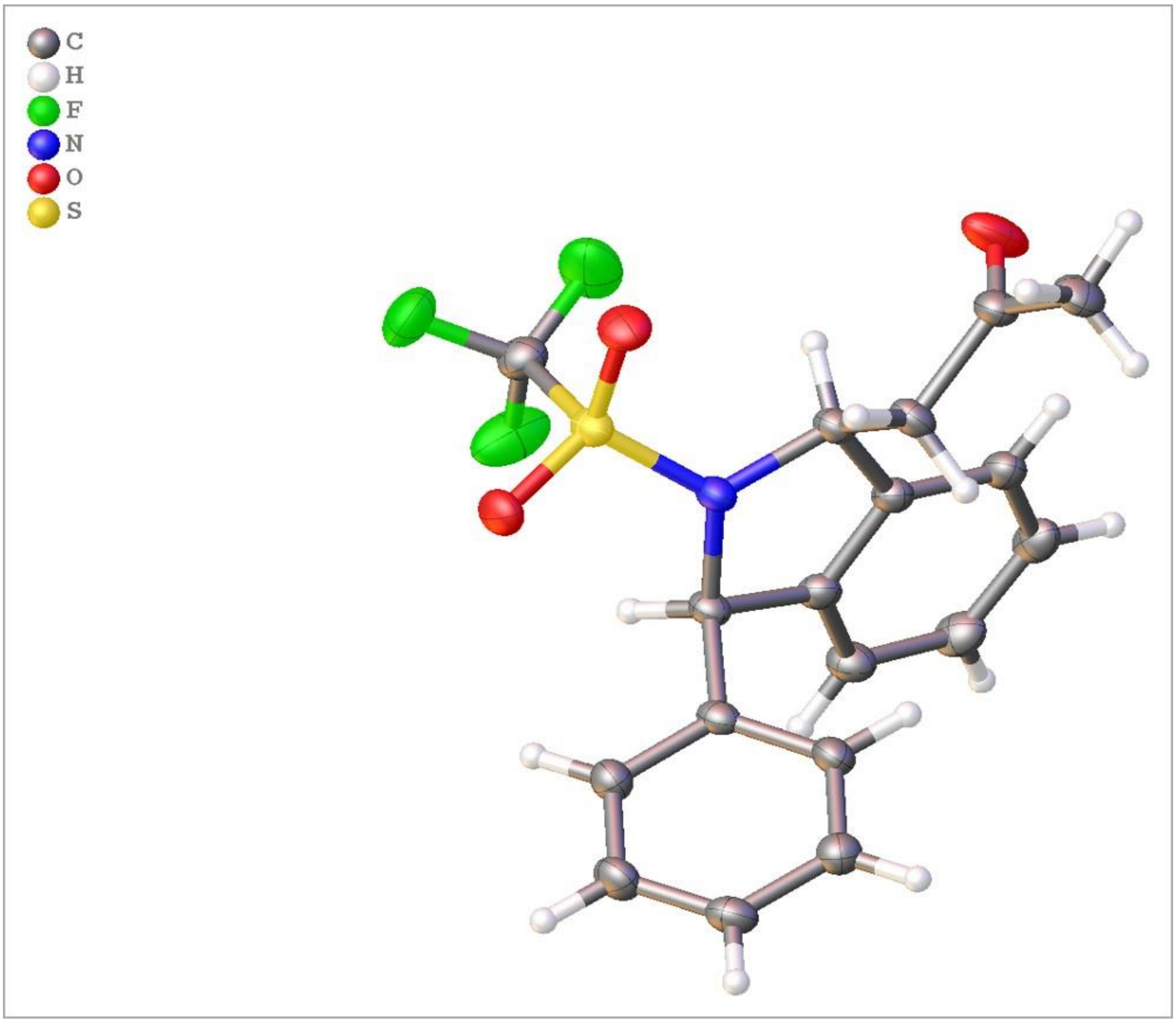
C
H
F
N
O
S

Table 2 Fractional Atomic Coordinates (×104) and Equivalent Isotropic Displacement Parameters (Å2×103) for UPS_UK_1163_0m_a. Ueq is defined as 1/3 of the trace of the orthogonalised UIJ tensor.

Atom	x	y	z	U(eq)
S11	9912.6(5)	5150.5(5)	5236.7(4)	23.3(2)
O13	10666.2(16)	5142.8(16)	4406.9(12)	31.0(4)
O12	10389.7(17)	5649.5(16)	6073.2(12)	32.6(4)
F17	10898.0(17)	3134.3(15)	5570.6(14)	47.2(5)
F15	9166(2)	3498.7(16)	6339.7(13)	54.1(5)
F16	9048(2)	3079.8(14)	4887.1(14)	54.0(5)
O9	7066(2)	6670.0(16)	7604.5(12)	43.4(5)
N2	8496.8(19)	5642.3(17)	5023.8(12)	22.1(4)
C3A	6415(2)	5243.1(19)	4489.6(15)	22.6(5)
C18	7998(2)	6234(2)	3386.7(14)	21.8(5)
C3	7797(2)	5333.7(19)	4138.4(14)	21.7(5)
C21	8428(2)	7885(2)	2018.1(16)	28.6(6)
C23	8888(2)	6035(2)	2680.7(17)	26.5(5)
C7A	6294(2)	5661.8(19)	5388.3(15)	22.1(5)
C22	9108(3)	6857(2)	1998.7(17)	30.6(6)
C9	7408(2)	7439(2)	7100.1(15)	25.6(5)
C19	7307(2)	7265(2)	3394.0(15)	25.7(5)
C4	5342(2)	4838(2)	4008.7(16)	27.5(5)
C6	3997(2)	5331(2)	5332.7(18)	30.5(6)
C7	5077(2)	5737(2)	5812.8(16)	26.6(5)
C20	7525(2)	8080(2)	2715.1(16)	27.7(5)
C1	7600(2)	5965(2)	5800.9(15)	22.6(5)
C8	7770(2)	7217(2)	6088.0(15)	25.4(5)
C5	4131(2)	4870(2)	4445.9(18)	30.4(5)
C10	7507(3)	8652(2)	7429.7(17)	31.4(6)
C14	9728(2)	3620(2)	5528.1(18)	30.8(6)

Table 3 Anisotropic Displacement Parameters (Å2×103) for UPS_UK_1163_0m_a. The Anisotropic displacement factor exponent takes the form:

-2π2[h2a*2U11+2hka*b*U12+…].

Atom	U11	U22	U33	U23	U13	U12
S11	17.5(3)	31.7(3)	20.8(3)	-0.1(2)	-0.1(2)	-1.4(2)
O13	22.2(8)	43.9(10)	26.9(8)	1.9(8)	6.3(7)	1.7(8)
O12	25.6(9)	43.3(10)	29.0(9)	-5.3(8)	-8.0(7)	-3.1(7)
F17	38.8(9)	45.8(9)	57.0(11)	7.7(8)	-5.0(8)	14.9(7)
F15	62.5(12)	50.9(10)	48.9(10)	18.6(8)	21.7(10)	3.2(9)
F16	65.6(12)	32.8(8)	63.6(12)	3.7(8)	-31.6(10)	-8.0(8)
O9	73.1(15)	35.8(10)	21.1(9)	0.8(8)	7.7(10)	-4.1(10)
N2	20.4(10)	29.7(10)	16.1(9)	-1.6(7)	0.2(8)	0.7(8)
C3A	22.6(11)	26.2(11)	19.1(10)	3.6(9)	-0.8(9)	-0.4(9)
C18	20.1(11)	29.7(11)	15.4(10)	-1.2(9)	-1.1(9)	-1.9(9)
C3	21.5(11)	27.7(11)	15.9(10)	-2.7(9)	-0.8(8)	-1.0(9)
C21	31.2(13)	35.6(13)	19.0(11)	2.6(9)	0.0(10)	-3.6(10)
C23	25.1(12)	31.5(12)	22.8(12)	-1.0(9)	2.1(10)	2.8(9)
C7A	21.3(11)	26.5(11)	18.6(11)	2.1(9)	-0.8(9)	1.0(9)
C22	28.4(13)	40.5(13)	22.8(12)	0.9(11)	6.5(10)	0.9(11)
C9	27.0(12)	32.6(12)	17.4(10)	-0.7(11)	-0.7(10)	2.1(10)
C19	26.0(12)	33.1(12)	17.8(10)	-2.0(9)	1.5(9)	2.5(10)
C4	26.7(12)	33.6(13)	22.4(11)	-1.3(9)	-2.5(9)	-3.3(10)
C6	20.1(11)	39.2(13)	32.1(13)	10.6(11)	2.6(9)	1.1(10)
C7	24.7(12)	34.1(12)	21.2(11)	4.9(9)	1.5(10)	3.0(10)
C20	27.7(13)	30.7(11)	24.8(12)	-0.2(10)	-1.4(11)	2.3(10)
C1	21.5(11)	29.7(11)	16.5(10)	-0.2(8)	1.0(9)	-0.5(9)
C8	27.8(12)	30.6(12)	17.8(10)	-2.0(9)	4.1(10)	-1.6(9)
C5	21.1(11)	37.6(13)	32.6(12)	5.1(11)	-4.1(10)	-3.7(10)
C10	38.2(15)	35.1(13)	20.8(11)	-6.8(11)	-0.8(10)	-0.5(11)
C14	26.0(13)	37.2(13)	29.1(12)	3.7(11)	-3.2(11)	1.1(10)

Table 4 Bond Lengths for UPS_UK_1163_0m_a.

Atom	Atom	Length/Å	Atom	Atom	Length/Å
S11	O13	1.4254(17)	C18	C23	1.388(3)
S11	O12	1.4274(18)	C18	C19	1.401(3)

S11	N2	1.594(2)	C21	C22	1.393(4)
S11	C14	1.851(3)	C21	C20	1.386(3)
F17	C14	1.331(3)	C23	C22	1.396(4)
F15	C14	1.313(3)	C7A	C7	1.395(3)
F16	C14	1.321(3)	C7A	C1	1.510(3)
O9	C9	1.210(3)	C9	C8	1.528(3)
N2	C3	1.510(3)	C9	C10	1.502(3)
N2	C1	1.499(3)	C19	C20	1.386(3)
C3A	C3	1.511(3)	C4	C5	1.395(3)
C3A	C7A	1.391(3)	C6	C7	1.392(4)
C3A	C4	1.386(3)	C6	C5	1.395(4)
C18	C3	1.527(3)	C1	C8	1.535(3)

Table 5 Bond Angles for UPS_UK_1163_0m_a.

Atom	Atom	Atom	Angle/˚	Atom	Atom	Atom	Angle/˚
O13	S11	O12	121.71(11)	C7	C7A	C1	127.49(19)
O13	S11	N2	109.64(10)	C21	C22	C23	120.1(2)
O13	S11	C14	103.89(11)	O9	C9	C8	121.2(2)
O12	S11	N2	109.15(10)	O9	C9	C10	122.3(2)
O12	S11	C14	103.89(12)	C10	C9	C8	116.5(2)
N2	S11	C14	107.48(11)	C20	C19	C18	120.4(2)
C3	N2	S11	120.73(15)	C3A	C4	C5	118.3(2)
C1	N2	S11	120.51(15)	C7	C6	C5	120.6(2)
C1	N2	C3	113.58(17)	C6	C7	C7A	118.3(2)
C7A	C3A	C3	111.8(2)	C19	C20	C21	120.5(2)
C4	C3A	C3	127.1(2)	N2	C1	C7A	101.07(17)
C4	C3A	C7A	121.1(2)	N2	C1	C8	111.90(19)
C23	C18	C3	119.6(2)	C7A	C1	C8	115.6(2)
C23	C18	C19	119.0(2)	C9	C8	C1	113.11(19)
C19	C18	C3	121.4(2)	C4	C5	C6	120.9(2)
N2	C3	C3A	100.43(16)	F17	C14	S11	109.40(18)
N2	C3	C18	111.74(18)	F15	C14	S11	110.62(18)

C3A	C3	C18	114.45(19)	F15	C14	F17	108.1(2)
C20	C21	C22	119.5(2)	F15	C14	F16	109.8(2)
C18	C23	C22	120.5(2)	F16	C14	S11	111.07(17)
C3A	C7A	C7	120.7(2)	F16	C14	F17	107.8(2)
C3A	C7A	C1	111.8(2)				

Table 6 Torsion Angles for UPS_UK_1163_0m_a.

A	B	C	D	Angle/˚	A	B	C	D	Angle/˚
S11	N2	C3	C3A	142.96(16)	C3	C3A	C4	C5	-177.3(2)
S11	N2	C3	C18	-95.3(2)	C3	C18	C23	C22	-178.5(2)
S11	N2	C1	C7A	-146.37(16)	C3	C18	C19	C20	178.7(2)
S11	N2	C1	C8	90.0(2)	C23	C18	C3	N2	101.7(2)
O13	S11	N2	C3	39.5(2)	C23	C18	C3	C3A	-145.0(2)
O13	S11	N2	C1	-167.55(17)	C23	C18	C19	C20	-1.1(3)
O13	S11	C14	F17	54.3(2)	C7A	C3A	C3	N2	10.8(2)
O13	S11	C14	F15	173.24(17)	C7A	C3A	C3	C18	-109.1(2)
O13	S11	C14	F16	-64.5(2)	C7A	C3A	C4	C5	0.6(4)
O12	S11	N2	C3	175.11(16)	C7A	C1	C8	C9	92.1(2)
O12	S11	N2	C1	-31.9(2)	C22	C21	C20	C19	0.6(4)
O12	S11	C14	F17	-73.9(2)	C19	C18	C3	N2	-78.0(3)
O12	S11	C14	F15	45.0(2)	C19	C18	C3	C3A	35.3(3)
O12	S11	C14	F16	167.23(19)	C19	C18	C23	C22	1.3(3)
O9	C9	C8	C1	2.1(3)	C4	C3A	C3	N2	-171.2(2)
N2	S11	C14	F17	170.48(17)	C4	C3A	C3	C18	69.0(3)
N2	S11	C14	F15	-70.60(19)	C4	C3A	C7A	C7	-3.2(3)
N2	S11	C14	F16	51.6(2)	C4	C3A	C7A	C1	175.4(2)
N2	C1	C8	C9	-152.93(19)	C7	C7A	C1	N2	177.4(2)
C3A	C7A	C7	C6	3.2(3)	C7	C7A	C1	C8	-61.6(3)
C3A	C7A	C1	N2	-1.1(2)	C7	C6	C5	C4	-2.0(4)
C3A	C7A	C1	C8	119.9(2)	C20	C21	C22	C23	-0.4(4)
C3A	C4	C5	C6	2.0(4)	C1	N2	C3	C3A	-11.7(2)
C18	C23	C22	C21	-0.5(4)	C1	N2	C3	C18	110.0(2)

C18	C19	C20	C21	0.2(4)	C1	C7A	C7	C6	-175.2(2)
C3	N2	C1	C7A	8.4(2)	C5	C6	C7	C7A	-0.6(3)
C3	N2	C1	C8	-115.2(2)	C10	C9	C8	C1	-178.2(2)
C3	C3A	C7A	C7	175.0(2)	C14	S11	N2	C3	-72.82(19)
C3	C3A	C7A	C1	-6.5(3)	C14	S11	N2	C1	80.14(19)

Table 7 Hydrogen Atom Coordinates (Å×104) and Isotropic Displacement Parameters (Å2×103) for UPS_UK_1163_0m_a.

Atom	x	y	z		U(eq)	
H3	8101		4573	3911	26	
H21	8580		8449	1557	34	
H23	9349		5333	2662	32	
H22	9724		6716	1521	37	
H19	6684		7406	3867	31	
H4	5429		4547	3397	33	
H6	3160		5368	5611	37	
H7	4987		6058	6414	32	
H20	7053		8777	2728	33	
H1	7774		5462	6347	27	
H8A	8688		7443	5988	31	
H8B	7220		7702	5685	31	
H5	3389		4574	4136	37	
H10A	6934		9137	7056	47	
H10B	8408		8917	7366	47	
H10C						
Experimental		7245		8695	8082	47
